# Supplementary material for: NiH-catalyzed asymmetric hydroarylation of N-acyl enamines to chiral benzylamines
Source: Nat Commun. 2021 Jan 27;12:638. doi: 10.1038/s41467-020-20888-5 (PMC7841163; doi:10.1038/s41467-020-20888-5)
Supplement: Supplementary file 1 — Supplementary Information [file 41467_2020_20888_MOESM1_ESM.pdf]

Supplementary Information

NiH-catalysed asymmetric hydroarylation of *N*-acyl enamines to chiral benzylamines

He et al.

I. General Information

Solvents were either purified and dried by passage through alumina and Q5 reactant-packed columns on a solvent purification system or bought from the commercial sources and transferred to the glovebox without exposure to air. Other commercial reagents were purchased from Sigma-Aldrich, Acros, Alfa Aesar, TCI, J&K, Energy Chemical, Bide Pharmatech Ltd. and were used as received. Flash chromatography was either performed using glass columns with *SiliaFlash*® P60 (SiliCycle, 230-400 mesh), or on pre-packed *Biotage*® SNAP columns using a Biotage Isolera Automated Flash Chromatography System.

NiI_2: (CAS 13462-90-3) and **$Ni(ClO_4)_2\cdot 6H_2O$**: (CAS 13520-61-1) were purchased from Strem Chemical;

$(MeO)_3SiH$: (CAS 2487-90-3) was purchased from Energy Chemical and stored under nitrogen at –20 °C in glove box;

NaF: (*white powder*, CAS 7681-49-4) was purchased from Acros (Stock No. 201290250);

DMAc: (*N*,*N*-Dimethylacetamide, CAS 127-19-5) was purchased from Acros (99.5%, Extra Dry, AcroSeal, Stock No.396350010) and stored under nitrogen in glove box;

All compounds (starting materials and products) were characterized by ^{1}H NMR, ^{13}C NMR, IR spectroscopy, melting point (where applicable), and high-resolution mass spectrometry. ^{1}H NMR spectra were recorded on Bruker 500 M MHz spectrometer and are referenced relative to residual $CDCl_3$ proton signals at δ 7.26 ppm. ^{19}F NMR spectra were recorded on a Bruker 500 MHz spectrometer and are referenced to $CFCl_3$ (δ 0.0 ppm). Data for ^{1}H and ^{19}F NMR are reported as follows: chemical shift (δ ppm), multiplicity (s = singlet, d = doublet, t = triplet, q = quartet, m = multiplet, ap = apparent), integration, and coupling constant (Hz). ^{13}C NMR spectra were recorded on a Bruker 500 MHz spectrometer and are referenced to $CDCl_3$ at δ 77.16 ppm. The ^{13}C NMR spectra were obtained with ^{1}H decoupling. Data for ^{13}C NMR are reported in terms of chemical shift and multiplicity where appropriate. IR spectra were obtained on a Bruker Alpha and was reported in terms of frequency of absorption (cm^{-1}). GC analyses were performed on an Agilent 8890 gas chromatograph with an FID detector using a J&W DB-1 column (10 m, 0.1 mm I.D.). High Resolution Mass spectra were obtained on a Bruker Daltonics, Inc. APEXIII 7.0 TESLA FTMS instrument (ESI). High pressure liquid chromatography (HPLC) was performed on Agilent 1260 Series chromatographs using Daicel Chiralcel columns (250 mm). Optical rotations were measured on a S3 Rudolph Research Analytical Autopol VI automatic polarimeter using a 50 mm pathlength cell at 589 nm with $[\alpha]_D$ values reported in degrees; concentration (c) is in g/100 mL. Melting points

(m.p.) were obtained on a Mel-Temp capillary melting point apparatus. The powder X-ray diffraction pattern (PXRD) measurements were carried out on a Philips X'pert MPD Pro *X*-ray diffractometer using Cu K*α* radiation (λ = 0.15418 nm), and the *X*-ray tube was operated at 40 kV and 40 mA at room temperature. Reactions were monitored by GC analysis and thin-layer chromatography (TLC) carried out on 0.25mm Jiang you silica gel plates (HSGF254) using UV light as a visualizing agent.

Safety note: MSDS indicates that $(MeO)_3SiH$ is a corrosive and flammable liquid. According to the literatures,[1-3] it may form pyrophoric gas (possibly SiH_4) during the storage or reaction. Although during our reaction, we used $(MeO)_3SiH$ without incident and SiH_4 was not observed, we urge the users of these procedures to be alert to the possibility of SiH_4 formation and possible exotherms and to take suitable precautions (suitable eye protection is also required).

Medium-sized screw-cap test tubes (8 mL) were used for all 0.20 mmol scale reactions:

Fisher 13 x 100 mm tubes (Cat. No. 14-959-35C)

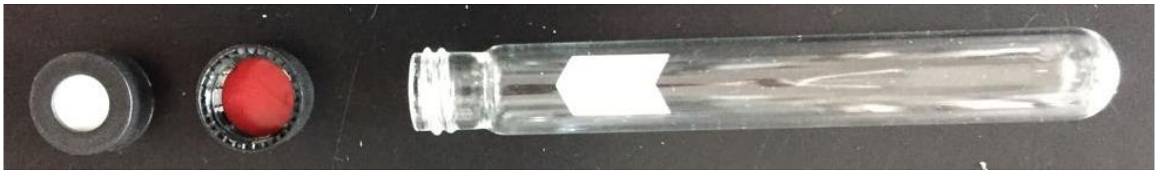

Cap with Septa: Thermo Scientific ASM PHN CAP w/PTFE/SIL (Cat. No. 03378316)

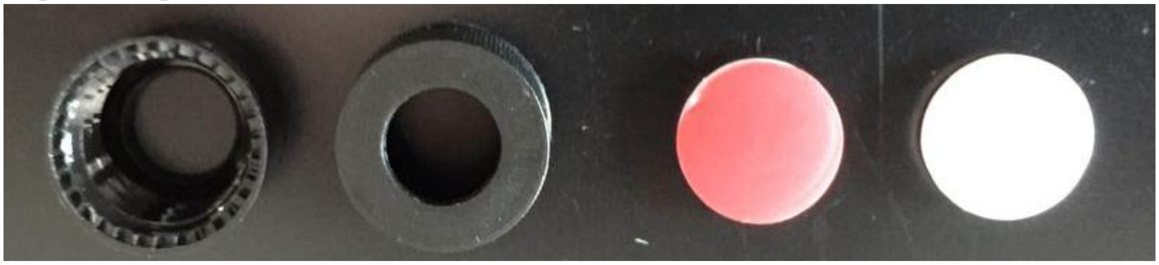

II. NiH-Catalyzed Asymmetric Hydroarylation of *N*-Acyl Enamines

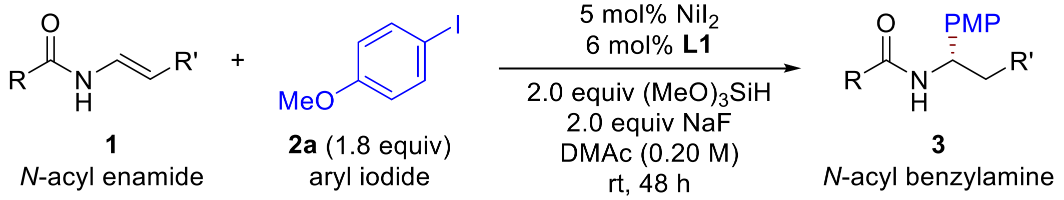

General Procedure (A) for the NiH-Catalyzed Asymmetric Hydroarylation of *N*-Acyl Enamines. In a nitrogen-filled glove box, to an oven-dried 8 mL screw-cap vial equipped with a magnetic stir bar was added NiI_2 (3.1 mg, 5.0 mol%), **L1** (7.2 mg, 6.0 mol%), NaF (16.8 mg, 2.0 equiv), and anhydrous DMAc (1.0 mL). The mixture was stirred for 20 min at room temperature, at which time (*E*)-*N*-(prop-1-en-1-yl)benzamide (**1a**) (32.2 mg, 0.20 mmol), 4-iodoanisole (84.0 mg, 0.36 mmol), and $(MeO)_3SiH$ (51.0 μL, 0.40 mmol) were added to the resulting mixture in this order. The tube was sealed with a teflon-lined screw cap, removed from the glove box and the reaction was stirred at rt (22~26 °C) for up to 48 h (the mixture was stirred at 750 rpm, ensuring that the base was uniformly suspended). After the reaction was complete, the reaction mixture was directly filtered through a short pad of silica gel [EtOAc in Petroleum ether (PE)] to give the crude product. 1,1,2,2-Tetrachloroethane (41.0 mg, 0.25 mmol) was added as internal standard for ^{1}H NMR analysis of the crude material. Dodecane (20 μL) was added as an internal standard for GC analysis. The product was purified by chromatography on silica gel for each substrate. The yields reported are the average of at least two experiments, unless otherwise indicated. The enantiomeric excesses (% ee) were determined by HPLC analysis using chiral stationary phases.

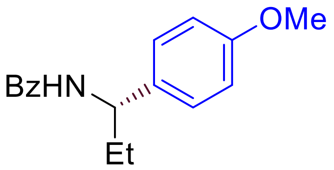

(*S*)-*N*-(1-(4-Methoxyphenyl)propyl)benzamide (Fig. 3, **3a**). From **4-Iodoanisole** (84.0 mg, 0.36 mmol, 1.8 equiv), the title compound was prepared following the general procedure **A** using NiI_2 (3.1 mg, 5.0 mol%), **L1** (7.2 mg, 6.0 mol%), NaF (16.8 mg, 2.0 equiv), (*E*)-*N*-(prop-1-en-1-yl)benzamide (**1a**) (32.2 mg, 0.20 mmol), $(MeO)_3SiH$ (51.0 μL, 0.40 mmol), anhydrous DMAc (1.0 mL). The reaction mixture was stirred for 48 h at

rt. The crude material was purified by flash column chromatography (0–10% EtOAc in PE) to provide the title compound as a white solid in 81% yield (43.1 mg).
Rf 0.4 (10% EtOAc in PE), UV;
^{1}H NMR (500 MHz, $CDCl_3$) δ 7.78 (d, *J* = 6.9 Hz, 2H), 7.49 (t, *J* = 7.4 Hz, 1H), 7.42 (t, *J* = 7.5 Hz, 2H), 7.30 (d, *J* = 8.6 Hz, 2H), 6.90 (d, *J* = 8.6 Hz, 2H), 6.44 (d, *J* = 8.2 Hz, 1H), 5.06 (q, *J* = 7.6 Hz, 1H), 3.81 (s, 3H), 2.03 – 1.85 (m, 2H), 0.96 (t, *J* = 7.4 Hz, 3H);
^{13}C NMR (126 MHz, $CDCl_3$) δ 166.8, 158.9, 134.8, 134.2, 131.4, 128.5, 127.9, 126.9, 114.1, 55.3, 54.9, 29.1, 10.9;
HRMS (ESI) calcd. for $C_{17}H_{20}NO_2$ $[M+H]^+$ *m/z* 270.1489, found 270.1488;
IR (neat, cm^{-1}) 3331, 1629, 1511, 1244, 691;
m.p. 135.3 – 136.7 °C;
$[\alpha]_D^{23}$ = –40.8 (*c* = 2.0, $CHCl_3$); 99% *ee*;
HPLC analysis CHIRALCEL OD-H column, 10% EtOH in hexane, 1.0 mL/min, 220 nm UV detector, t_R (minor) = 6.5 min, t_R (major) = 7.5 min.

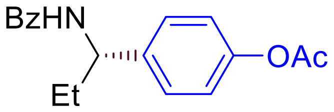

(*S*)-4-(1-Benzamidopropyl)phenyl acetate (Fig. 3, **3b**). From **4-Iodophenyl acetate** (94.3 mg, 0.36 mmol, 1.8 equiv), the title compound was prepared following the general procedure **A** using NiI_2 (3.1 mg, 5.0 mol%), **L1** (7.2 mg, 6.0 mol%), NaF (16.8 mg, 2.0 equiv), (*E*)-*N*-(prop-1-en-1-yl)benzamide (**1a**) (32.2 mg, 0.20 mmol), $(MeO)_3SiH$ (51.0 μL, 0.40 mmol), anhydrous DMAc (1.0 mL). The reaction mixture was stirred for 48 h at rt. The crude material was purified by flash column chromatography (0–20% EtOAc in PE) to provide the title compound as a white solid in 51% yield (30.1 mg).
Rf 0.4 (30% EtOAc in PE), UV;
^{1}H NMR (500 MHz, $CDCl_3$) δ 7.78 (d, *J* = 7.4 Hz, 2H), 7.51 (t, *J* = 7.3 Hz, 1H), 7.44 (t, *J* = 7.5 Hz, 2H), 7.38 (d, *J* = 8.5 Hz, 2H), 7.08 (d, *J* = 8.5 Hz, 2H), 6.41 (d, *J* = 8.2 Hz, 1H), 5.11 (q, *J* = 7.6 Hz, 1H), 2.31 (s, 3H), 2.01 – 1.89 (m, *J* = 7.1 Hz, 2H), 0.98 (t, *J* = 7.3 Hz, 3H);
^{13}C NMR (126 MHz, $CDCl_3$) δ 169.5, 166.8, 149.8, 139.7, 134.5, 131.5, 128.5, 127.8, 126.9, 121.7, 54.8, 29.1, 21.2, 10.9;
HRMS (ESI) calcd. for $C_{18}H_{20}NO_3$ $[M+H]^+$ *m/z* 298.1438, found 298.1437;
IR (neat, cm^{-1}) 2971, 1753, 1686, 1631, 1066;
m.p. 160.6 – 161.9 °C;
$[\alpha]_D^{23}$ = –29.5 (*c* = 1.5, $CHCl_3$); 98% *ee*;
HPLC analysis CHIRALCEL AD-H column, 20% ^{i}PrOH in hexane, 1.0 mL/min, 220 nm UV detector, t_R (minor) = 6.3 min, t_R (major) = 8.4 min.

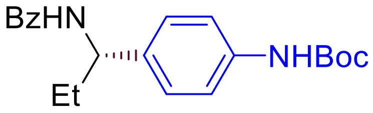

***tert*-Butyl (*S*)-(4-(1-benzamidopropyl)phenyl)carbamate** (Fig. 3, **3c**). From ***tert*-Butyl (4-iodophenyl)carbamate** (95.7 mg, 0.36 mmol, 1.8 equiv), the title compound was prepared following the general procedure **A** using NiI_2 (3.1 mg, 5.0 mol%), **L1** (7.2 mg, 6.0 mol%), NaF (16.8 mg, 2.0 equiv), (*E*)-*N*-(prop-1-en-1-yl)benzamide (**1a**) (32.2 mg,

0.20 mmol), $(MeO)_3SiH$ (51.0 μL, 0.40 mmol), anhydrous DMAc (1.0 mL). The reaction mixture was stirred for 48 h at rt. The crude material was purified by flash column chromatography (0–10% EtOAc in PE) to provide the title compound as a white solid in 99% yield (70.1 mg).

Rf 0.5 (20% EtOAc in PE), UV;

^{1}H NMR (500 MHz, Methanol-d_4) δ 7.83 (d, J = 7.3 Hz, 2H), 7.53 (t, J = 7.4 Hz, 1H), 7.46 (t, J = 7.5 Hz, 2H), 7.38 (d, J = 8.3 Hz, 2H), 7.31 (d, J = 8.5 Hz, 2H), 4.95 (q, J = 7.6 Hz, 1H), 2.00 – 1.83 (m, 2H), 1.52 (s, 9H), 0.99 (t, J = 7.3 Hz, 3H);

^{13}C NMR (126 MHz, Methanol-d_4) δ 168.6, 153.9, 138.1, 137.2, 134.6, 131.1, 128.1, 126.9, 126.8, 79.3, 55.3, 28.7, 27.3, 10.3;

HRMS (ESI) calcd. for $C_{21}H_{27}N_2O_3$ $[M+H]^+$ m/z 355.2016, found 355.2015;

IR (neat, cm^{-1}) 2986, 1688, 1632, 1419, 1067;

m.p. 183.7 – 183.9 °C;

$[\alpha]_D^{23}$= –30.8 (c = 2.0, MeOH); 97% *ee*;

HPLC analysis CHIRALCEL AD-H column, 20% ^{i}PrOH in hexane, 1.0 mL/min, 220 nm UV detector, t_R (minor) = 9.0 min, t_R (major) = 18.2 min.

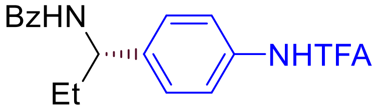

(*S*)-*N*-(1-(4-(2,2,2-Trifluoroacetamido)phenyl)propyl)benzamide (Fig. 3, **3d**). From **2,2,2-Trifluoro-*N*-(4-iodophenyl)acetamide** (94.5 mg, 0.30 mmol, 1.5 equiv), the title compound was prepared following the general procedure **A** using $Ni(ClO_4)_2·6H_2O$ (3.6 mg, 5.0 mol%), **L1** (7.2 mg, 6.0 mol%), NaF (16.8 mg, 2.0 equiv), (*E*)-*N*-(prop-1-en-1-yl)benzamide (**1a**) (32.2 mg, 0.20 mmol), $(MeO)_3SiH$ (51.0 μL, 0.40 mmol), anhydrous DMAc (2.0 mL). The reaction mixture was stirred for 48 h at rt. The crude material was purified by flash column chromatography (0–10% EtOAc in PE) to provide the title compound as a white solid in 69% yield (48.5 mg).

Rf 0.3 (10% EtOAc in PE), UV;

^{1}H NMR (500 MHz, Methanol-d_4) δ 7.84 (d, J = 7.2 Hz, 2H), 7.63 (d, J = 8.6 Hz, 2H), 7.54 (t, J = 7.4 Hz, 1H), 7.50 – 7.40 (m, 4H), 5.00 (q, J = 7.2, 6.2 Hz, 1H), 2.03 – 1.85 (m, 2H), 1.01 (t, J = 7.4 Hz, 3H);

^{13}C NMR (126 MHz, Methanol-d_4) δ 168.7 (d, J = 9.2 Hz), 155.3 (q, J = 37.4 Hz), 140.8 (d, J = 2.1 Hz), 135.1, 134.5 (d, J = 5.0 Hz), 131.2, 128.1, 127.1, 127.0, 120.8, 116.0 (q, J = 287.7 Hz), 55.4 (d, J = 12.8 Hz), 28.7 (d, J = 3.6 Hz), 10.3;

^{19}F NMR (471 MHz, Methanol-d_4) δ –77.0;

HRMS (ESI) calcd. for $C_{18}H_{18}F_3N_2O_2$ $[M+H]^+$ m/z 351.1315, found 351.1316;

IR (neat, cm^{-1}) 2987, 1695, 1626, 1066;

m.p. 201.3 – 201.9 °C;

$[\alpha]_D^{23}$ = –34.3 (c = 1.5, MeOH); 98% *ee*;

HPLC analysis CHIRALCEL AD-H column, 20% ^{i}PrOH in hexane, 1.0 mL/min, 220 nm UV detector, t_R (minor) = 6.9 min, t_R (major)= 11.3 min.

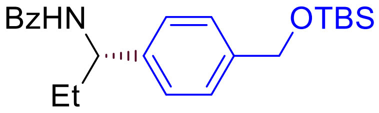

(*S*)-*N*-(1-(4-(((*tert*-Butyldimethylsilyl)oxy)methyl)phenyl)propyl)benzamide (Fig. 3, **3e**). From ***tert*-Butyl((4-iodobenzyl)oxy)dimethylsilane** (104.5 mg, 0.36 mmol, 1.8 equiv), the title compound was prepared following the general procedure **A** using NiI_2 (3.1 mg, 5.0 mol%), **L1** (7.2 mg, 6.0 mol%), NaF (16.8 mg, 2.0 equiv), (*E*)-*N*-(prop-1-en-1-yl)benzamide (**1a**) (32.2 mg, 0.20 mmol), $(MeO)_3SiH$ (51.0 µL, 0.40 mmol), anhydrous DMAc (1.0 mL). The reaction mixture was stirred for 48 h at rt. The crude material was purified by flash column chromatography (0–10% EtOAc in PE) to provide the title compound as a white solid in 66% yield (50.7 mg).
Rf 0.2 (10% EtOAc in PE), UV;
^{1}H NMR (500 MHz, $CDCl_3$) δ 7.79 (d, *J* = 7.2 Hz, 2H), 7.50 (t, *J* = 7.4 Hz, 1H), 7.43 (t, *J* = 7.5 Hz, 2H), 7.37 – 7.31 (m, 4H), 6.44 (d, *J* = 8.0 Hz, 1H), 5.11 (q, *J* = 7.5 Hz, 1H), 4.75 (s, 2H), 2.03 – 1.90 (m, 2H), 0.97 (s, 12H), 0.12 (s, 6H);
^{13}C NMR (126 MHz, $CDCl_3$) δ 166.8, 140.6, 140.6, 134.7, 131.4, 128.6, 126.9, 126.6, 126.3, 64.6, 55.2, 29.1 (contain **2 C**), 25.9, 10.8, –5.2;
HRMS (ESI) calcd. for $C_{23}H_{34}NO_2Si$ $[M+H]^+$ *m/z* 384.2353, found 384.2351;
IR (neat, cm^{-1}) 3318, 2959, 1634, 1527, 693;
m.p. 58.1 – 60.2 ^oC;
$[\alpha]_D^{23}$ = –22.8 (*c* = 1.0, $CHCl_3$); 98% *ee*;
HPLC analysis 1) CHIRALCEL AD-H column, 20% ^{i}PrOH in hexane, 1.0 mL/min, 220 nm UV detector, t_R (minor) = 3.8 min, t_R (major) = 4.4 min. 2) CHIRALCEL OD-H column, 20% ^{i}PrOH in hexane, 1.0 mL/min, 220 nm UV detector, t_R (minor) = 4.8 min, t_R (major) = 5.5 min.

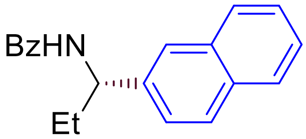

(*S*)-*N*-(1-(Naphthalen-2-yl)propyl)benzamide (Fig. 3, **3f**). From **2-Iodonaphthalene** (76.2 mg, 0.36 mmol, 1.8 equiv), the title compound was prepared following the general procedure **A** using NiI_2 (3.1 mg, 5.0 mol%), **L1** (7.2 mg, 6.0 mol%), NaF (16.8 mg, 2.0 equiv), (*E*)-*N*-(prop-1-en-1-yl)benzamide (**1a**) (32.2 mg, 0.20 mmol), $(MeO)_3SiH$ (51.0 µL, 0.40 mmol), anhydrous DMAc (1.0 mL). The reaction mixture was stirred for 48 h at rt. The crude material was purified by flash column chromatography (0–10% EtOAc in PE) to provide the title compound as a yellow solid in 86% yield (50.3 mg).
Rf 0.2 (10% EtOAc in PE), UV;
^{1}H NMR (500 MHz, $CDCl_3$) δ 7.88 – 7.80 (m, 6H), 7.56 – 7.38 (m, 6H), 6.70 (d, *J* = 8.2 Hz, 1H), 5.29 (q, *J* = 7.4 Hz, 1H), 2.13 – 1.99 (m, 2H), 1.01 (t, *J* = 7.3 Hz, 3H);
^{13}C NMR (126 MHz, $CDCl_3$) δ 166.9, 139.5, 134.6, 133.4, 132.8, 131.5, 128.5 (contain **2 C**), 127.9, 127.6, 127.0, 126.2, 125.8, 125.5, 124.5, 55.5, 29.0, 10.9;
HRMS (ESI) calcd. for $C_{20}H_{20}NO$ $[M+H]^+$ *m/z* 290.1539, found 290.1538;
IR (neat, cm^{-1}) 2987, 1688, 1631, 1066;
m.p. 134.7 – 135.2 ^oC;
$[\alpha]_D^{23}$ = –21.3 (*c* = 2.0, $CHCl_3$); >99% *ee*;
HPLC analysis CHIRALCEL AD-H column, 20% ^{i}PrOH in hexane, 1.0 mL/min, 220 nm UV detector, t_R (minor) = 6.3 min, t_R (major) = 9.9 min.

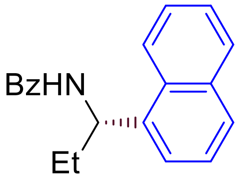

(*S*)-*N*-(1-(Naphthalen-1-yl)propyl)benzamide (Fig. 3, **3g**). From **1-Iodonaphthalene** (91.4 mg, 0.36 mmol, 1.8 equiv), the title compound was prepared following the general procedure **A** using $Ni(ClO_4)_2·6H_2O$ (3.6 mg, 5.0 mol%), **L1** (7.2 mg, 6.0 mol%), NaF (16.8 mg, 2.0 equiv), (*E*)-*N*-(prop-1-en-1-yl)benzamide (**1a**) (32.2 mg, 0.20 mmol), $(MeO)_3SiH$ (51.0 μL, 0.40 mmol), anhydrous DMAc (2.0 mL). The reaction mixture was stirred for 48 h at rt. The crude material was purified by flash column chromatography (0–10% EtOAc in PE) to provide the title compound as a white solid in 65% yield (37.5 mg).
Rf 0.2 (10% EtOAc in PE), UV;
^{1}H NMR (500 MHz, $CDCl_3$) δ 8.28 (d, *J* = 8.4 Hz, 1H), 7.90 (d, *J* = 8.1 Hz, 1H), 7.83 (d, *J* = 8.1 Hz, 1H), 7.77 (d, *J* = 7.5 Hz, 2H), 7.60 – 7.55 (m, 2H), 7.54 – 7.46 (m, 3H), 7.40 (t, *J* = 7.5 Hz, 2H), 6.50 (d, *J* = 8.5 Hz, 1H), 5.98 (q, *J* = 7.5 Hz, 1H), 2.24 – 2.12 (m, *J* = 7.0 Hz, 2H), 1.09 (t, *J* = 7.3 Hz, 3H);
^{13}C NMR (126 MHz, $CDCl_3$) δ 166.8, 137.5, 134.5, 134.1, 131.6, 131.5, 128.8, 128.5, 128.3, 126.9, 126.6, 125.9, 125.2, 123.4, 122.9, 50.9, 28.5, 11.2;
HRMS (ESI) calcd. for $C_{20}H_{20}NO$ $[M+H]^+$ *m/z* 290.1539, found 290.1538;
IR (neat, cm^{-1}) 3304, 1969, 1631, 693;
m.p. 167.6 – 168.2 °C;
$[\alpha]_D^{23}$ = +35.8 (*c* = 1.0, $CHCl_3$); >99% *ee*;
HPLC analysis CHIRALCEL AD-H column, 20% ^{i}PrOH in hexane, 1.0 mL/min, 220 nm UV detector, t_R (minor) = 5.8 min, t_R (major) = 8.4 min.

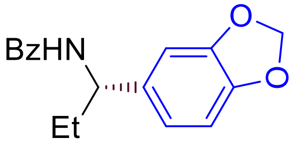

(*S*)-*N*-(1-(Benzo[*d*][1,3]dioxol-5-yl)propyl)benzamide (Fig. 3, **3h**). From **5-Iodobenzo[*d*][1,3]dioxole** (84.0 mg, 0.36 mmol, 1.8 equiv), the title compound was prepared following the general procedure **A** using NiI_2 (3.1 mg, 5.0 mol%), **L1** (7.2 mg, 6.0 mol%), NaF (16.8 mg, 2.0 equiv), (*E*)-*N*-(prop-1-en-1-yl)benzamide (**1a**) (32.2 mg, 0.20 mmol), $(MeO)_3SiH$ (51.0 μL, 0.40 mmol), anhydrous DMAc (1.0 mL). The reaction mixture was stirred for 48 h at rt. The crude material was purified by flash column chromatography (0–10% EtOAc in PE) to provide the title compound as a white solid in 51% yield (28.8 mg).
Rf 0.4 (20% EtOAc in PE), UV;
^{1}H NMR (500 MHz, $CDCl_3$) δ 7.78 (d, *J* = 7.2 Hz, 2H), 7.50 (t, *J* = 7.4 Hz, 1H), 7.43 (t, *J* = 7.5 Hz, 2H), 6.88 – 6.82 (m, 2H), 6.79 (d, *J* = 7.9 Hz, 1H), 6.39 (d, *J* = 9.1 Hz, 1H), 5.96 (s, 2H), 5.00 (q, *J* = 7.6 Hz, 1H), 1.98 – 1.93 (m, 1H), 1.91 – 1.84 (m, 1H), 0.96 (t, *J* = 7.4 Hz, 3H);
^{13}C NMR (126 MHz, $CDCl_3$) δ 166.8, 147.9, 146.8, 136.1, 134.6, 131.4, 128.5, 126.9, 120.1, 108.3, 107.1, 101.0, 55.3, 29.2, 10.8;
HRMS (ESI) calcd. for $C_{17}H_{18}NO_3$ $[M+H]^+$ *m/z* 284.1281, found 284.1280;

IR (neat, cm^{-1}) 3334, 2987, 1633, 1503, 934;
m.p. 129.2 – 129.9 °C;
$[\alpha]_D^{23}$ = –26.0 (*c* = 1.0, $CHCl_3$); 99% *ee*;
HPLC analysis CHIRALCEL AD-H column, 20% ^{i}PrOH in hexane, 1.0 mL/min, 220 nm UV detector, t_R (minor) = 7.3 min, t_R (major) = 11.8 min.

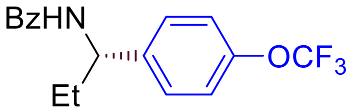

(*S*)-*N*-(1-(4-(Trifluoromethoxy)phenyl)propyl)benzamide (Fig. 3, **3i**). From **1-Iodo-4-(trifluoromethoxy)benzene** (86.4 mg, 0.30 mmol, 1.5 equiv), the title compound was prepared following the general procedure **A** using $Ni(ClO_4)_2{\cdot}6H_2O$ (3.6 mg, 5.0 mol%), **L1** (7.2 mg, 6.0 mol%), NaF (16.8 mg, 2.0 equiv), (*E*)-*N*-(prop-1-en-1-yl)benzamide (**1a**) (32.2 mg, 0.20 mmol), $(MeO)_3SiH$ (51.0 µL, 0.40 mmol), anhydrous DMAc (2.0 mL). The reaction mixture was stirred for 48 h at rt. The crude material was purified by flash column chromatography (0–10% EtOAc in PE) to provide the title compound as a white solid in 69% yield (44.8 mg).
Rf 0.2 (10% EtOAc in PE), UV;
^{1}H NMR (500 MHz, $CDCl_3$) δ 7.79 (d, *J* = 7.1 Hz, 2H), 7.51 (t, *J* = 7.4 Hz, 1H), 7.45 – 7.35 (m, 4H), 7.19 (d, *J* = 8.2 Hz, 2H), 6.63 (d, *J* = 8.0 Hz, 1H), 5.09 (q, *J* = 7.5 Hz, 1H), 1.97 – 1.88 (m, 2H), 0.97 (t, *J* = 7.3 Hz, 3H);
^{13}C NMR (126 MHz, $CDCl_3$) δ 167.0, 148.3, 141.0, 134.4, 131.6, 128.6, 128.0, 126.9, 121.1, 120.5 (q, *J* = 257.1 Hz), 54.7, 29.1, 10.8;
^{19}F NMR (471 MHz, $CDCl_3$) δ –57.9;
HRMS (ESI) calcd. for $C_{17}H_{17}F_3NO_2$ $[M+H]^+$ *m/z* 324.1206, found 324.1205;
IR (neat, cm^{-1}) 3675, 2971, 1633, 1522, 1066;
m.p. 109.2 – 110.4 °C;
$[\alpha]_D^{23}$ = –4.9 (*c* = 2.5, $CHCl_3$); >99% *ee*;
HPLC analysis CHIRALCEL AD-H column, 20% ^{i}PrOH in hexane, 1.0 mL/min, 220 nm UV detector, t_R (minor) = 4.3 min, t_R (major) = 5.6 min.

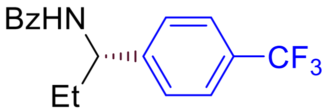

(*S*)-*N*-(1-(4-(Trifluoromethyl)phenyl)propyl)benzamide (Fig. 3, **3j**). From **1-Iodo-4-(trifluoromethyl)benzene** (81.6 mg, 0.30 mmol, 1.5 equiv), the title compound was prepared following the general procedure **A** using $Ni(ClO_4)_2{\cdot}6H_2O$ (3.6 mg, 5.0 mol%), **L1** (7.2 mg, 6.0 mol%), NaF (16.8 mg, 2.0 equiv), (*E*)-*N*-(prop-1-en-1-yl)benzamide (**1a**) (32.2 mg, 0.20 mmol), $(MeO)_3SiH$ (51.0 µL, 0.40 mmol), anhydrous DMAc (2.0 mL). The reaction mixture was stirred for 48 h at rt. The crude material was purified by flash column chromatography (0–10% EtOAc in PE) to provide the title compound as a white solid in 75% yield (46.1 mg).
Rf 0.3 (10% EtOAc in PE), UV;
^{1}H NMR (500 MHz, $CDCl_3$) δ 7.79 (d, *J* = 7.1 Hz, 2H), 7.59 (d, *J* = 8.0 Hz, 2H), 7.51 (t, *J* = 7.4 Hz, 1H), 7.46 (d, *J* = 8.0 Hz, 2H), 7.42 (t, *J* = 7.5 Hz, 2H), 6.71 (d, *J* = 7.8 Hz, 1H), 5.11 (q, *J* = 7.4 Hz, 1H), 1.99 – 1.86 (m, 2H), 0.98 (t, *J* = 7.4 Hz, 3H);

^{13}C NMR (126 MHz, $CDCl_3$) δ 167.1, 146.5, 134.3, 131.7, 129.5 (q, J = 32.5 Hz), 128.6, 127.0, 126.9, 125.6 (q, J = 3.9 Hz), 124.1 (q, J = 272.2 Hz), 55.2, 29.1, 10.8;
^{19}F NMR (471 MHz, $CDCl_3$) δ –62.5;
HRMS (ESI) calc. for $C_{17}H_{17}F_3NO$ $[M+H]^+$ *m/z* 308.1257, found 308.1256;
IR (neat, cm^{-1}) 3313, 2967, 1635, 1330, 1067;
m.p. 134.5 – 135.7 °C;
$[\alpha]_D^{23}$ = +1.3 (c = 2.0, $CHCl_3$); >99% *ee*;
HPLC analysis CHIRALCEL AD-H column, 20% ^{i}PrOH in hexane, 1.0 mL/min, 220 nm UV detector, t_R (minor) = 4.5 min, t_R (major) = 6.2 min.

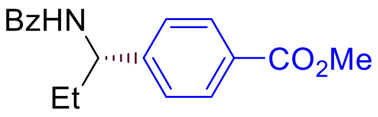

Methyl (*S*)-4-(1-benzamidopropyl)benzoate (Fig. 3, **3k**). From **Methyl 4-iodobenzoate** (79.0 mg, 0.30 mmol, 1.5 equiv), the title compound was prepared following the general procedure **A** using $Ni(ClO_4)_2·6H_2O$ (3.6 mg, 5.0 mol%), **L1** (7.2 mg, 6.0 mol%), NaF (16.8 mg, 2.0 equiv), (*E*)-*N*-(prop-1-en-1-yl)benzamide (**1a**) (32.2 mg, 0.20 mmol), $(MeO)_3SiH$ (51.0 μL, 0.40 mmol), anhydrous DMAc (2.0 mL). The reaction mixture was stirred for 48 h at rt. The crude material was purified by flash column chromatography (0–10% EtOAc in PE) to provide the title compound as a white solid in 76% yield (43.0 mg).
Rf 0.3 (20% EtOAc in PE), UV;
^{1}H NMR (500 MHz, $CDCl_3$) δ 8.00 (d, J = 8.3 Hz, 2H), 7.79 (d, J = 7.1 Hz, 2H), 7.50 (t, J = 7.3 Hz, 1H), 7.42 (d, J = 8.5 Hz, 4H), 6.73 (d, J = 17.4 Hz, 1H), 5.12 (q, J = 7.5 Hz, 1H), 3.91 (s, 3H), 2.00 – 1.88 (m, 2H), 0.97 (t, J = 7.3 Hz, 3H);
^{13}C NMR (126 MHz, $CDCl_3$) δ 167.0, 166.8, 147.6, 147.6, 134.4, 131.6, 129.9, 128.6, 127.0, 126.7, 55.2, 52.1, 29.1, 10.8;
HRMS (ESI) calcd. for $C_{18}H_{20}NO_3$ $[M+H]^+$ *m/z* 298.1438, found 298.1439;
IR (neat, cm^{-1}) 3317, 2930, 1717, 1632, 1264, 706;
m.p. 144.9 – 146.0 °C;
$[\alpha]_D^{23}$ = –3.2 (c = 1.5, $CHCl_3$); 99% *ee*;
HPLC analysis CHIRALCEL AD-H column, 20% ^{i}PrOH in hexane, 1.0 mL/min, 220 nm UV detector, t_R (minor) = 7.6 min, t_R (major) = 8.7 min.

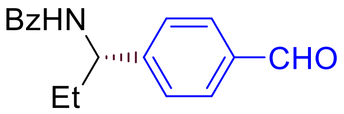

(*S*)-*N*-(1-(4-Formylphenyl)propyl)benzamide (Fig. 3, **3l**). From **4-Iodobenzaldehyde** (69.6 mg, 0.30 mmol, 1.5 equiv), the title compound was prepared following the general procedure **A** using $Ni(ClO_4)_2·6H_2O$ (3.6 mg, 5.0 mol%), **L1** (7.2 mg, 6.0 mol%), NaF (16.8 mg, 2.0 equiv), (*E*)-*N*-(prop-1-en-1-yl)benzamide (**1a**) (32.2 mg, 0.20 mmol), $(MeO)_3SiH$ (51.0 μL, 0.40 mmol), anhydrous DMAc (2.0 mL). The reaction mixture was stirred for 48 h at rt. The crude material was purified by flash column chromatography (0–10% EtOAc in PE) to provide the title compound as a white solid in 82% yield (43.8 mg).
Rf 0.2 (2% EtOAc in PE), UV;

^{1}H NMR (500 MHz, $CDCl_3$) δ 9.96 (s, 1H), 7.85 – 7.77 (m, 4H), 7.53 – 7.46 (m, 3H), 7.39 (t, *J* = 7.7 Hz, 2H), 6.96 (d, *J* = 7.8 Hz, 1H), 5.11 (q, *J* = 7.5 Hz, 1H), 1.99 – 1.86 (m, 2H), 0.97 (t, *J* = 7.4 Hz, 3H);
^{13}C NMR (126 MHz, $CDCl_3$) δ 192.0, 167.2, 149.6, 135.5, 134.3, 131.7, 130.2, 128.6, 127.3, 127.0, 55.5, 29.1, 10.9;
HRMS (ESI) calcd. for $C_{17}H_{18}NO_2$ $[M+H]^+$ *m/z* 268.1332, found 268.1331;
IR (neat, cm^{-1}) 3300, 2965, 1700, 1632, 690;
m.p. 131.2 – 131.6 °C;
$[\alpha]_D^{23}$ = –2.4 (*c* = 2.0, $CHCl_3$); >99% *ee*;
HPLC analysis CHIRALCEL AD-H column, 20% ^{i}PrOH in hexane, 1.0 mL/min, 220 nm UV detector, t_R (major) = 13.7 min.

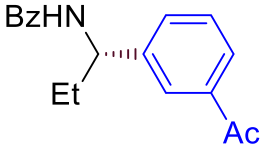

(*S*)-*N*-(1-(3-Acetylphenyl)propyl)benzamide (Fig. 3, **3m**). From **1-(3-Iodophenyl)ethan-1-one** (74.0 mg, 0.30 mmol, 1.5 equiv), the title compound was prepared following the general procedure **A** using $Ni(ClO_4)_2·6H_2O$ (3.6 mg, 5.0 mol%), **L1** (7.2 mg, 6.0 mol%), NaF (16.8 mg, 2.0 equiv), (*E*)-*N*-(prop-1-en-1-yl)benzamide (**1a**) (32.2 mg, 0.20 mmol), $(MeO)_3SiH$ (51.0 μL, 0.40 mmol), anhydrous DMAc (2.0 mL). The reaction mixture was stirred for 48 h at rt. The crude material was purified by flash column chromatography (0–15% EtOAc in PE) to provide the title compound as a yellow oil in 84% yield (47.4 mg).
Rf 0.3 (20% EtOAc in PE), UV;
^{1}H NMR (500 MHz, $CDCl_3$) δ 7.99 (s, 1H), 7.84 (d, *J* = 7.7 Hz, 1H), 7.81 – 7.77 (m, 2H), 7.58 (d, *J* = 7.7 Hz, 1H), 7.48 (t, *J* = 7.4 Hz, 1H), 7.45 – 7.38 (m, 3H), 6.79 (d, *J* = 8.0 Hz, 1H), 5.13 (q, *J* = 7.6 Hz, 1H), 2.59 (s, 3H), 2.04 – 1.87 (m, 2H), 0.97 (t, *J* = 7.4 Hz, 3H);
^{13}C NMR (126 MHz, $CDCl_3$) δ 198.2, 167.1, 143.2, 137.5, 134.4, 131.9, 131.6, 128.6, 127.5, 127.4, 127.0, 126.0, 55.3, 29.2, 26.7, 10.9;
HRMS (ESI) calcd. for $C_{18}H_{20}NO_2$ $[M+H]^+$ *m/z* 282.1489, found 282.1492;
IR (neat, cm^{-1}) 3306, 2965, 1680, 1633, 692;
$[\alpha]_D^{23}$ = –10.4 (*c* = 2.0, $CHCl_3$); 98% *ee*;
HPLC analysis CHIRALCEL AD-H column, 20% ^{i}PrOH in hexane, 1.0 mL/min, 220 nm UV detector, t_R (major) = 6.5 min, t_R (minor) = 10.0 min.

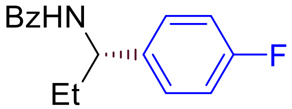

(*S*)-*N*-(1-(4-Fluorophenyl)propyl)benzamide (Fig. 3, **3n**). From **1-Fluoro-4-iodobenzene** (66.6 mg, 0.30 mmol, 1.5 equiv), the title compound was prepared following the general procedure **A** using $Ni(ClO_4)_2·6H_2O$ (3.6 mg, 5.0 mol%), **L1** (7.2 mg, 6.0 mol%), NaF (16.8 mg, 2.0 equiv), (*E*)-*N*-(prop-1-en-1-yl)benzamide (**1a**) (32.2 mg, 0.20 mmol), $(MeO)_3SiH$ (51.0 μL, 0.40 mmol), anhydrous DMAc (2.0 mL). The reaction mixture was stirred for 48 h at rt. The crude material was purified by flash

column chromatography (0–10% EtOAc in PE) to provide the title compound as a white solid in 78% yield (40.3 mg).
Rf 0.2 (10% EtOAc in PE), UV;
^{1}H NMR (500 MHz, $CDCl_3$) δ 7.78 (d, *J* = 7.6 Hz, 2H), 7.51 (t, *J* = 7.3 Hz, 1H), 7.43 (t, *J* = 7.5 Hz, 2H), 7.37 – 7.30 (m, 2H), 7.04 (t, *J* = 8.5 Hz, 2H), 6.48 (d, *J* = 8.3 Hz, 1H), 5.07 (q, *J* = 7.5 Hz, 1H), 2.01 – 1.86 (m, 2H), 0.97 (t, *J* = 7.3 Hz, 3H);
^{13}C NMR (126 MHz, $CDCl_3$) δ 166.8, 162.0 (d, *J* = 245.3 Hz), 138.0 (d, *J* = 3.3 Hz), 134.5, 131.6, 128.6, 128.3(d, *J* = 8.0 Hz), 126.9, 115.5 (d, *J* = 21.3 Hz), 54.8, 29.2, 10.9;
^{19}F NMR (471 MHz, $CDCl_3$) δ –115.3;
HRMS (ESI) calcd. for $C_{16}H_{17}FNO$ $[M+H]^+$ *m/z* 258.1289, found 258.1290;
IR (neat, cm^{-1}) 3321, 2969, 1634, 1513 ,1027;
m.p. 127.3 – 128.3 °C;
$[\alpha]_D^{23}$ = –12.5 (*c* = 1.2, $CHCl_3$); >99% *ee*;
HPLC analysis CHIRALCEL AD-H column, 20% ^{i}PrOH in hexane, 1.0 mL/min, 220 nm UV detector, t_R (major) = 7.7 min.

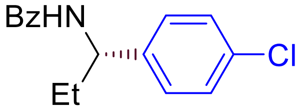

(*S*)-*N*-(1-(4-Chlorophenyl)propyl)benzamide (Fig. 3, **3o**). From **1-Chloro-4-iodobenzene** (71.5 mg, 0.30 mmol, 1.5 equiv), the title compound was prepared following the general procedure **A** using $Ni(ClO_4)_2{\cdot}6H_2O$ (3.6 mg, 5.0 mol%), **L1** (7.2 mg, 6.0 mol%), NaF (16.8 mg, 2.0 equiv), (*E*)-*N*-(prop-1-en-1-yl)benzamide (**1a**) (32.2 mg, 0.20 mmol), $(MeO)_3SiH$ (51.0 μL, 0.40 mmol), anhydrous DMAc (2.0 mL). The reaction mixture was stirred for 48 h at rt. The crude material was purified by flash column chromatography (0–10% EtOAc in PE) to provide the title compound as a white solid in 72% yield (39.3 mg).
Rf 0.2 (10% EtOAc in PE), UV;
^{1}H NMR (500 MHz, $CDCl_3$) δ 7.78 (d, *J* = 7.2 Hz, 2H), 7.52 (t, *J* = 7.4 Hz, 1H), 7.43 (t, *J* = 7.6 Hz, 2H), 7.35 – 7.27 (m, 4H), 6.46 (d, *J* = 7.8 Hz, 1H), 5.06 (q, *J* = 7.5 Hz, 1H), 2.01 – 1.84 (m, 2H), 0.97 (t, *J* = 7.4 Hz, 3H);
^{13}C NMR (126 MHz, $CDCl_3$) δ 166.9, 140.7, 134.4, 133.1, 131.6, 128.8, 128.6, 128.1, 126.9, 54.8, 29.1, 10.8;
HRMS (ESI) calcd. for $C_{16}H_{17}ClNO$ $[M+H]^+$ *m/z* 274.0993, found 274.0992;
IR (neat, cm^{-1}) 3294, 2968, 1631, 689;
m.p. 158.8 – 159.4 °C;
$[\alpha]_D^{23}$ = –6.5 (*c* = 2.0, $CHCl_3$); >99% *ee*;
HPLC analysis CHIRALCEL AD-H column, 20% ^{i}PrOH in hexane, 1.0 mL/min, 220 nm UV detector, t_R (major) = 7.9 min.

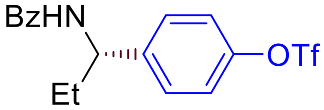

(*S*)-4-(1-Benzamidopropyl)phenyl trifluoromethanesulfonate (Fig. 3, **3p**). From **4-Iodophenyl trifluoromethanesulfonate** (105.6 mg, 0.30 mmol, 1.5 equiv), the title compound was prepared following the general procedure **A** using $Ni(ClO_4)_2{\cdot}6H_2O$ (3.6

mg, 5.0 mol%), **L1** (7.2 mg, 6.0 mol%), NaF (16.8 mg, 2.0 equiv), (*E*)-*N*-(prop-1-en-1-yl)benzamide (**1a**) (32.2 mg, 0.20 mmol), $(MeO)_3SiH$ (51.0 μL, 0.40 mmol), anhydrous DMAc (2.0 mL). The reaction mixture was stirred for 48 h at rt. The crude material was purified by flash column chromatography (0–10% EtOAc in PE) to provide the title compound as a white solid in 86% yield (66.3 mg).

Rf 0.2 (10% EtOAc in PE), UV;

^{1}H NMR (500 MHz, $CDCl_3$) δ 7.79 (d, *J* = 7.0 Hz, 2H), 7.51 (t, *J* = 7.4 Hz, 1H), 7.42 (d, *J* = 8.1 Hz, 4H), 7.26 – 7.20 (m, 2H), 6.73 (d, *J* = 8.0 Hz, 1H), 5.08 (q, *J* = 7.5 Hz, 1H), 1.95 – 1.86 (m, 2H), 0.97 (t, *J* = 7.3 Hz, 3H);

^{13}C NMR (126 MHz, $CDCl_3$) δ 167.1, 148.5, 143.0, 134.2, 131.7, 128.6, 128.5, 127.0, 121.5, 118.7 (q, *J* = 320.7 Hz). 54.7, 29.1, 10.8;

^{19}F NMR (471 MHz, $CDCl_3$) δ –72.9;

HRMS (ESI) calcd. for $C_{17}H_{17}F_3NO_4S$ $[M+H]^+$ *m/z* 388.0825, found 388.0824;

IR (neat, cm^{-1}) 3343, 2987, 1635, 1075, 694;

m.p. 106.5 – 107.5 °C;

$[\alpha]_D^{23}$ = –2.8 (*c* = 2.5, $CHCl_3$); >99% *ee*;

HPLC analysis CHIRALCEL AD-H column, 20% ^{i}PrOH in hexane, 1.0 mL/min, 220 nm UV detector, t_R (major) = 8.3 min.

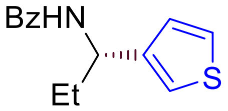

(*S*)-*N*-(1-(Thiophen-3-yl)propyl)benzamide (Fig. 3, **3q**). From **3-Iodothiophene** (76.0 mg, 0.36 mmol, 1.8 equiv), the title compound was prepared following the general procedure **A** using NiI_2 (3.1 mg, 5.0 mol%), **L1** (7.2 mg, 6.0 mol%), NaF (16.8 mg, 2.0 equiv), (*E*)-*N*-(prop-1-en-1-yl)benzamide (**1a**) (32.2 mg, 0.20 mmol), $(MeO)_3SiH$ (51.0 μL, 0.40 mmol), anhydrous DMAc (1.0 mL). The reaction mixture was stirred for 48 h at rt. The crude material was purified by flash column chromatography (0–10% EtOAc in PE) to provide the title compound as a white solid in 67% yield (32.9 mg).

Rf 0.4 (10% EtOAc in PE), UV;

^{1}H NMR (500 MHz, $CDCl_3$) δ 7.79 (d, *J* = 7.3 Hz, 2H), 7.55 – 7.47 (m, 1H), 7.42 (t, *J* = 7.5 Hz, 2H), 7.34 – 7.29 (m, 1H), 7.20 (d, *J* = 2.9 Hz, 1H), 7.10 (d, *J* = 5.0 Hz, 1H), 6.45 (d, *J* = 8.6 Hz, 1H), 5.26 (q, *J* = 7.6 Hz, 1H), 2.05 – 1.92 (m, 2H), 1.00 (t, *J* = 7.3 Hz, 3H);

^{13}C NMR (126 MHz, $CDCl_3$) δ 166.8, 143.2, 134.6, 131.5, 128.6, 126.9, 126.4, 126.2, 121.2, 50.9, 28.7, 10.7;

HRMS (ESI) calcd. for $C_{14}H_{16}NOS$ $[M+H]^+$ *m/z* 246.0947, found 246.0946;

IR (neat, cm^{-1}) 3339, 2966, 1632, 1523, 1075;

m.p. 95.9 – 96.5 °C;

$[\alpha]_D^{23}$ = –44.0 (*c* = 1.2, $CHCl_3$); 97% *ee*;

HPLC analysis CHIRALCEL AD-H column, 20% ^{i}PrOH in hexane, 1.0 mL/min, 220 nm UV detector, t_R (minor) = 5.9 min, t_R (major) = 8.4 min.

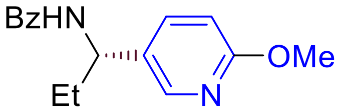

(*S*)-*N*-(1-(6-Methoxypyridin-3-yl)propyl)benzamide (Fig. 3, **3r**). From **5-Iodo-2-methoxypyridine** (84.6 mg, 0.36 mmol, 1.8 equiv), the title compound was prepared following the general procedure **A** using NiI_2 (3.1 mg, 5.0 mol%), **L1** (7.2 mg, 6.0 mol%), NaF (16.8 mg, 2.0 equiv), (*E*)-*N*-(prop-1-en-1-yl)benzamide (**1a**) (32.2 mg, 0.20 mmol), $(MeO)_3SiH$ (51.0 μL, 0.40 mmol), anhydrous DMAc (1.0 mL). The reaction mixture was stirred for 48 h at rt. The crude material was purified by flash column chromatography (0–20% EtOAc in PE) to provide the title compound as a white solid in 45% yield (24.2 mg).
Rf 0.4 (30% EtOAc in PE), UV;
^{1}H NMR (500 MHz, $CDCl_3$) δ 8.19 (s, 1H), 7.76 (d, *J* = 7.3 Hz, 2H), 7.58 (d, *J* = 8.5 Hz, 1H), 7.49 (t, *J* = 7.3 Hz, 1H), 7.41 (t, *J* = 7.6 Hz, 2H), 6.73 (d, *J* = 8.5 Hz, 1H), 6.54 (d, *J* = 8.2 Hz, 1H), 5.04 (q, *J* = 7.5 Hz, 1H), 3.93 (d, *J* = 1.4 Hz, 3H), 2.01 – 1.86 (m, 2H), 0.97 (t, *J* = 7.3 Hz, 3H);
^{13}C NMR (126 MHz, $CDCl_3$) δ 166.9, 163.5, 145.1, 137.5, 134.4, 131.6, 130.4, 128.6, 126.9, 110.9, 53.5, 52.8, 28.8, 10.8;
HRMS (ESI) calcd. for $C_{16}H_{19}N_2O_2$ $[M+H]^+$ *m/z* 271.1441, found 271.1439;
IR (neat, cm^{-1}) 3327, 2971, 1634, 1055;
m.p. 117.7 – 118.2 °C;
$[\alpha]_D^{23}$ = –22.2 (*c* = 1.0, $CHCl_3$); 98% *ee*;
HPLC analysis CHIRALCEL AD-H column, 20% ^{i}PrOH in hexane, 1.0 mL/min, 220 nm UV detector, t_R (minor) = 6.0 min, t_R (major) = 7.4 min.

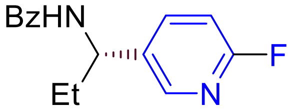

(*S*)-*N*-(1-(6-Fluoropyridin-3-yl)propyl)benzamide (Fig. 3, **3s**). From **2-Fluoro-5-iodopyridine** (66.9 mg, 0.30 mmol, 1.5 equiv), the title compound was prepared following the general procedure **A** using $Ni(ClO_4)_2·6H_2O$ (3.6 mg, 5.0 mol%), **L1** (7.2 mg, 6.0 mol%), NaF (16.8 mg, 2.0 equiv), (*E*)-*N*-(prop-1-en-1-yl)benzamide (**1a**) (32.2 mg, 0.20 mmol), $(MeO)_3SiH$ (51.0 μL, 0.40 mmol), anhydrous DMAc (2.0 mL). The reaction mixture was stirred for 48 h at rt. The crude material was purified by flash column chromatography (0–10% EtOAc in PE) to provide the title compound as a white solid in 72% yield (37.3 mg).
Rf 0.2 (20% EtOAc in PE), UV;
^{1}H NMR (500 MHz, $CDCl_3$) δ 8.20 (s, 1H), 7.81 – 7.73 (m, 3H), 7.49 (t, *J* = 7.4 Hz, 1H), 7.39 (t, *J* = 7.7 Hz, 2H), 6.90 – 6.83 (m, 2H), 5.05 (q, *J* = 7.6 Hz, 1H), 2.01 – 1.83 (m, 2H), 0.97 (t, *J* = 7.4 Hz, 3H);
^{13}C NMR (126 MHz, $CDCl_3$) δ 167.5, 162.8 (d, *J* = 238.6 Hz), 145.9 (d, *J* = 14.9 Hz), 139.8 (d, *J* = 7.9 Hz), 135.7 (d, *J* = 4.6 Hz), 134.1, 131.7, 128.6, 127.0, 109.4 (d, *J* = 37.5 Hz), 52.7 (d, *J* = 1.3 Hz), 28.8, 10.8;
^{19}F NMR (471 MHz, $CDCl_3$) δ –70.1;
HRMS (ESI) calcd. for $C_{15}H_{16}FN_2O$ $[M+H]^+$ *m/z* 259.1241, found 259.1240;
IR (neat, cm^{-1}) 3313, 2970, 1632, 1295;
m.p. 99.0 – 99.4 °C;
$[\alpha]_D^{23}$ = +0.8 (*c* = 1.5, $CHCl_3$); 98% *ee*;

HPLC analysis CHIRALCEL AD-H column, 20% ^{i}PrOH in hexane, 1.0 mL/min, 220 nm UV detector, t_R (minor) = 4.7 min, t_R (major) = 7.2 min.

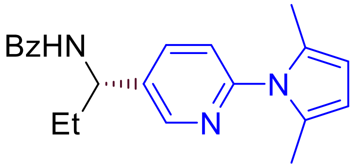

(*S*)-*N*-(1-(6-(2,5-Dimethyl-1H-pyrrol-1-yl)pyridin-3-yl)propyl)benzamide (Fig. 3, **3t**). From **2-(2,5-Dimethyl-1H-pyrrol-1-yl)-5-iodopyridine** (89.4 mg, 0.30 mmol, 1.5 equiv), the title compound was prepared following the general procedure **A** using $Ni(ClO_4)_2·6H_2O$ (3.6 mg, 5.0 mol%), **L1** (7.2 mg, 6.0 mol%), NaF (16.8 mg, 2.0 equiv), (*E*)-*N*-(prop-1-en-1-yl)benzamide (**1a**) (32.2 mg, 0.20 mmol), $(MeO)_3SiH$ (51.0 µL, 0.40 mmol), anhydrous DMAc (2.0 mL). The reaction mixture was stirred for 48 h at rt. The crude material was purified by flash column chromatography (0–20% EtOAc in PE) to provide the title compound as a white solid in 67% yield (44.5 mg).
Rf 0.4 (30% EtOAc in PE), UV;
^{1}H NMR (500 MHz, $CDCl_3$) δ 8.63 (d, *J* = 2.5 Hz, 1H), 7.83 (d, *J* = 8.4 Hz, 3H), 7.53 (t, *J* = 7.4 Hz, 1H), 7.45 (t, *J* = 7.6 Hz, 2H), 7.20 (d, *J* = 8.1 Hz, 1H), 6.76 (d, *J* = 8.0 Hz, 1H), 5.91 (s, 2H), 5.20 (q, *J* = 7.6 Hz, 1H), 2.13 (s, 6H), 2.04 – 1.97 (m, 2H), 1.04 (t, *J* = 7.3 Hz, 3H);
^{13}C NMR (126 MHz, $CDCl_3$) δ 167.2, 151.1, 147.7, 136.6, 136.6, 134.1, 131.8, 128.7, 127.0, 121.7, 106.9, 52.9, 29.0, 13.3, 10.9;
HRMS (ESI) calcd. for $C_{21}H_{24}N_3O$ $[M+H]^+$ *m/z* 334.1914, found 334.1916;
IR (neat, cm^{-1}) 2987, 1653, 1636, 1074;
m.p. 132.1 – 133.2 °C;
$[\alpha]_D^{23}$ = –18.0 (*c* = 2.0, $CHCl_3$); 99% *ee*;
HPLC analysis CHIRALCEL AD-H column, 20% ^{i}PrOH in hexane, 1.0 mL/min, 220 nm UV detector, t_R (minor) = 4.2 min, t_R (major) = 4.8 min.

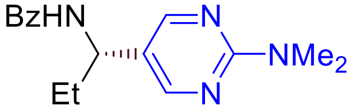

(*S*)-*N*-(1-(2-(Dimethylamino)pyrimidin-5-yl)propyl)benzamide (Fig. 3, **3u**). From **5-Iodo-*N*,*N*-dimethylpyrimidin-2-amine** (74.4 mg, 0.30 mmol, 1.5 equiv), the title compound was prepared following the general procedure **A** using $Ni(ClO_4)_2·6H_2O$ (3.6 mg, 5.0 mol%), **L1** (7.2 mg, 6.0 mol%), NaF (16.8 mg, 2.0 equiv), (*E*)-*N*-(prop-1-en-1-yl)benzamide (**1a**) (32.2 mg, 0.20 mmol), $(MeO)_3SiH$ (51.0 µL, 0.40 mmol), anhydrous DMAc (2.0 mL). The reaction mixture was stirred for 48 h at rt. The crude material was purified by flash column chromatography (0–25% EtOAc in PE) to provide the title compound as a white solid in 46% yield (25.9 mg).
Rf 0.3 (50% EtOAc in PE), UV;
^{1}H NMR (500 MHz, $CDCl_3$) δ 8.35 (s, 2H), 7.75 (d, *J* = 7.6 Hz, 2H), 7.49 (t, *J* = 7.4 Hz, 1H), 7.41 (t, *J* = 7.6 Hz, 2H), 6.42 (d, *J* = 7.8 Hz, 1H), 4.93 (q, *J* = 7.6 Hz, 1H), 3.19 (s, 6H), 2.01 – 1.85 (m, 2H), 0.99 (t, *J* = 7.3 Hz, 3H);
^{13}C NMR (126 MHz, $CDCl_3$) δ 166.9, 161.6, 156.5, 134.4, 131.6, 128.6, 126.9, 121.9, 51.2, 37.2, 28.3, 10.8;

HRMS (ESI) calcd. for $C_{16}H_{21}N_4O$ $[M+H]^+$ *m/z* 285.1710, found 285.1708;
IR (neat, cm^{-1}) 3294, 2971, 1631, 1407, 1066;
m.p. 122.2 – 122.6 °C;
$[\alpha]_D^{23}$ = –31.8 (*c* = 1.0, $CHCl_3$); 98% *ee*;
HPLC analysis CHIRALCEL AD-H column, 20% ^{i}PrOH in hexane, 1.0 mL/min, 220 nm UV detector, t_R (major) = 6.2 min, t_R (minor) = 7.8 min.

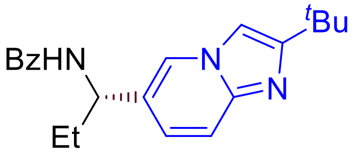

(*S*)-*N*-(1-(2-(*tert*-Butyl)imidazo[1,2-*a*]pyridin-6-yl)propyl)benzamide (Fig. 3, **3v**). From **2-(*tert*-Butyl)-6-iodoimidazo[1,2-*a*]pyridine** (94.5 mg, 0.30 mmol, 1.5 equiv), the title compound was prepared following the general procedure **A** using $Ni(ClO_4)_2 \cdot 6H_2O$ (3.6 mg, 5.0 mol%), **L1** (7.2 mg, 6.0 mol%), NaF (16.8 mg, 2.0 equiv), (*E*)-*N*-(prop-1-en-1-yl)benzamide (**1a**) (32.2 mg, 0.20 mmol), $(MeO)_3SiH$ (51.0 µL, 0.40 mmol), anhydrous DMAc (2.0 mL). The reaction mixture was stirred for 48 h at rt. The crude material was purified by flash column chromatography (0–15% EtOAc in PE) to provide the title compound as a yellow oil in 54% yield (36.4 mg).
Rf 0.3 (30% EtOAc in PE), UV;
^{1}H NMR (500 MHz, $CDCl_3$) δ 8.10 (s, 1H), 7.77 (d, *J* = 7.2 Hz, 2H), 7.53 (d, *J* = 9.3 Hz, 1H), 7.48 (t, *J* = 6.8 Hz, 1H), 7.39 (t, *J* = 7.6 Hz, 2H), 7.28 (s, 1H), 7.14 (d, *J* = 9.3 Hz, 1H), 6.74 (d, *J* = 7.9 Hz, 1H), 5.01 (q, *J* = 7.6 Hz, 1H), 2.03 – 1.89 (m, 2H), 1.39 (s, 9H), 0.97 (t, *J* = 7.4 Hz, 3H);
^{13}C NMR (126 MHz, $CDCl_3$) δ 167.0, 157.2, 144.1, 134.2, 131.7, 128.6, 126.9, 126.0, 124.0, 123.0, 117.2, 107.2, 53.1, 32.3, 30.2, 28.1, 10.9;
HRMS (ESI) calcd. for $C_{21}H_{26}N_3O$ $[M+H]^+$ *m/z* 336.2070, found 336.2070;
IR (neat, cm^{-1}) 2962, 1633, 1509, 692;
$[\alpha]_D^{23}$ = +61.6 (*c* = 1.0, $CHCl_3$); 98% *ee*;
HPLC analysis CHIRALCEL AD-H column, 20% ^{i}PrOH in hexane, 1.0 mL/min, 220 nm UV detector, t_R (minor) = 6.8 min, t_R (major) = 8.9 min.

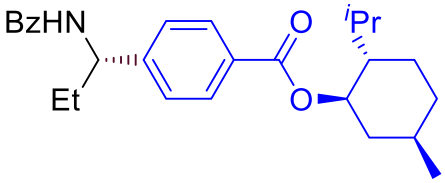

(*1R,2S,5R*)-2-Isopropyl-5-methylcyclohexyl 4-((*S*)-1-benzamidopropyl)benzoate (Fig. 3, **3w**). From **(*1R,2S,5R*)-2-Isopropyl-5-methylcyclohexyl 4-iodobenzoate** (115.9 mg, 0.30 mmol, 1.5 equiv), the title compound was prepared following the general procedure **A** using $Ni(ClO_4)_2 \cdot 6H_2O$ (3.6 mg, 5.0 mol%), **L1** (7.2 mg, 6.0 mol%), NaF (16.8 mg, 2.0 equiv), (*E*)-*N*-(prop-1-en-1-yl)benzamide (**1a**) (32.2 mg, 0.20 mmol), $(MeO)_3SiH$ (51.0 µL, 0.40 mmol), anhydrous DMAc (2.0 mL). The reaction mixture was stirred for 48 h at rt. The crude material was purified by flash column chromatography (0–15% EtOAc in PE) to provide the title compound as a yellow oil in 82% yield (69.4 mg).
Rf 0.3 (20% EtOAc in PE), UV;

^{1}H NMR (500 MHz, $CDCl_3$) δ 8.03 (d, *J* = 8.3 Hz, 2H), 7.79 (d, *J* = 7.2 Hz, 2H), 7.50 (t, *J* = 7.4 Hz, 1H), 7.46 – 7.38 (m, 4H), 6.69 (d, *J* = 7.5 Hz, 1H), 5.12 (q, *J* = 7.5 Hz, 1H), 4.98 – 4.89 (m, 1H), 2.12 (d, *J* = 11.9 Hz, 1H), 2.00 – 1.89 (m, 3H), 1.74 (d, *J* = 11.5 Hz, 2H), 1.56 (t, *J* = 11.6 Hz, 2H), 1.17 – 1.07 (m, 2H), 0.99 – 0.91 (m, 10H), 0.80 (d, *J* = 7.0 Hz, 3H);
^{13}C NMR (126 MHz, $CDCl_3$) δ 167.0, 165.8, 147.4, 134.4, 131.6, 130.0, 129.8, 128.6, 127.0, 126.6, 74.8, 55.3, 47.2, 40.9, 34.3, 31.5, 29.1, 26.5, 23.6, 22.1, 20.8, 16.5, 10.8;
HRMS (ESI) calcd. for $C_{27}H_{36}O_3$ $[M+H]^+$ *m/z* 422.2690, found 422.2689;
IR (neat, cm^{-1}) 3304, 2957, 1711, 1635, 1271;
$[\alpha]_D^{23}$ = –41.1 (*c* = 1.5, $CHCl_3$); >20:1 dr;

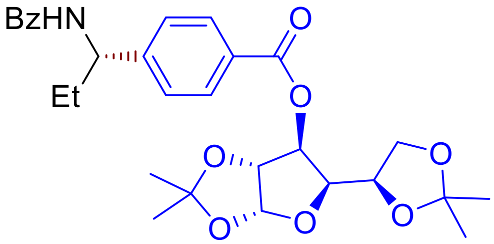

(*3aR,5R,6S,6aR*)-5-((*R*)-2,2-Dimethyl-1,3-dioxolan-4-yl)-2,2-dimethyltetrahydrofuro[2,3-*d*][1,3]dioxol-6-yl 4-((*S*)-1-benzamidopropyl)benzoate (Fig. 3, **3x**). From **(*3aR,5R,6S,6aR*)-5-((*R*)-2,2-Dimethyl-1,3-dioxolan-4-yl)-2,2-dimethyltetrahydrofuro[2,3-*d*][1,3]dioxol-6-yl 4-iodobenzoate** (147.1 mg, 0.30 mmol, 1.5 equiv), the title compound was prepared following the general procedure **A** using $Ni(ClO_4)_2{\cdot}6H_2O$ (3.6 mg, 5.0 mol%), **L1** (7.2 mg, 6.0 mol%), NaF (16.8 mg, 2.0 equiv), (*E*)-*N*-(prop-1-en-1-yl)benzamide (**1a**) (32.2 mg, 0.20 mmol), $(MeO)_3SiH$ (51.0 µL, 0.40 mmol), anhydrous DMAc (2.0 mL). The reaction mixture was stirred for 48 h at rt. The crude material was purified by flash column chromatography (0–15% EtOAc in PE) to provide the title compound as a yellow oil in 86% yield (90.2 mg).
Rf 0.2 (30% EtOAc in PE), UV;
^{1}H NMR (500 MHz, $CDCl_3$) δ 7.99 (d, *J* = 8.3 Hz, 2H), 7.78 (d, *J* = 7.2 Hz, 2H), 7.49 (t, *J* = 7.4 Hz, 1H), 7.46 – 7.38 (m, 4H), 6.75 (d, *J* = 7.8 Hz, 1H), 5.94 (d, *J* = 3.7 Hz, 1H), 5.49 (d, *J* = 2.8 Hz, 1H), 5.09 (q, *J* = 7.5 Hz, 1H), 4.61 (d, *J* = 3.6 Hz, 1H), 4.38 – 4.31 (m, 2H), 4.14 – 4.07 (m, 2H), 1.99 – 1.86 (m, 2H), 1.56 (s, 3H), 1.42 (s, 3H), 1.32 (s, 3H), 1.27 (s, 3H), 0.97 (t, *J* = 7.4 Hz, 3H);
^{13}C NMR (126 MHz, $CDCl_3$) δ 167.1, 164.9, 148.4, 134.3, 131.7, 130.1, 128.6, 128.5, 127.0, 126.8, 112.3, 109.4, 105.1, 83.4, 79.9, 76.6, 72.6, 67.2, 55.4, 29.1, 26.8, 26.7, 26.2, 25.2, 10.8;
HRMS (ESI) calcd. for $C_{29}H_{36}NO_8$ $[M+H]^+$ *m/z* 526.2435, found 526.2433;
IR (neat, cm^{-1}) 2987, 1722, 1636, 1073, 728;
$[\alpha]_D^{23}$ = –26.8 (*c* = 4.5, $CHCl_3$); >20:1 dr;

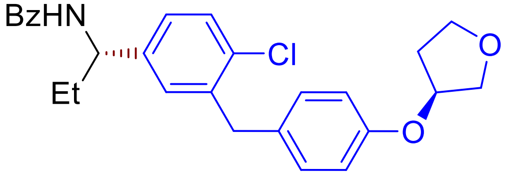

***N*-((*S*)-1-(4-Chloro-3-(4-(((*S*)-tetrahydrofuran-3 yl)oxy)benzyl)phenyl)propyl)benzamide** (Fig. 3, **3y**). From **(*S*)-3-(4-(2-Chloro-5-iodobenzyl)phenoxy)tetrahydrofuran** (124.4 mg, 0.30 mmol, 1.5 equiv), the title compound was prepared following the general procedure **A** using $Ni(ClO_4)_2 \cdot 6H_2O$ (3.6 mg, 5.0 mol%), **L1** (7.2 mg, 6.0 mol%), NaF (16.8 mg, 2.0 equiv), (*E*)-*N*-(prop-1-en-1-yl)benzamide (**1a**) (32.2 mg, 0.20 mmol), $(MeO)_3SiH$ (51.0 µL, 0.40 mmol), anhydrous DMAc (2.0 mL). The reaction mixture was stirred for 48 h at rt. The crude material was purified by flash column chromatography (0–20% EtOAc in PE) to provide the title compound as a white solid in 68% yield (61.2 mg).

Rf 0.2 (40% EtOAc in PE), UV;

^{1}H NMR (500 MHz, $CDCl_3$) δ 7.74 (d, *J* = 7.5 Hz, 2H), 7.51 (t, *J* = 7.3 Hz, 1H), 7.42 (t, *J* = 7.4 Hz, 2H), 7.34 (d, *J* = 7.9 Hz, 1H), 7.16 (d, *J* = 11.5 Hz, 2H), 7.10 (d, *J* = 8.2 Hz, 2H), 6.79 (d, *J* = 8.2 Hz, 2H), 6.47 (d, *J* = 7.9 Hz, 1H), 5.02 (d, *J* = 7.4 Hz, 1H), 4.88 (s, 1H), 4.04 (s, 2H), 4.02 – 3.96 (m, 3H), 3.94 – 3.86 (m, 1H), 2.25 – 2.12 (m, *J* = 7.6, 6.9 Hz, 2H), 1.92 – 1.82 (m, 2H), 0.94 (t, *J* = 7.2 Hz, 3H);

^{13}C NMR (126 MHz, $CDCl_3$) δ 166.9, 155.9, 141.2, 139.1, 134.5, 132.9, 131.1, 131.6, 130.1, 129.7, 129.3, 128.6, 126.9, 125.7, 115.4, 77.3, 73.2, 67.2, 54.8, 38.4, 33.0, 29.2, 10.8;

HRMS (ESI) calcd. for $C_{27}H_{29}ClNO_3$ $[M+H]^+$ *m/z* 450.1830, found 450.1829;

IR (neat, cm^{-1}) 2987, 1632, 1066;

m.p. 142.1 – 143.9 °C;

$[\alpha]_D^{23}$ = –1.3 (*c* = 2.0, $CHCl_3$); >20:1 dr;

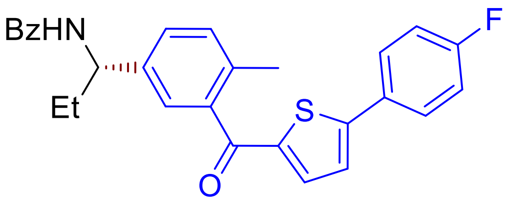

(*S*)-*N*-(1-(3-(5-(4-Fluorophenyl)thiophene-2-carbonyl)-4-methylphenyl)propyl)benzamide (Fig. 3, **3z**). From **(5-(4-Fluorophenyl)thiophen-2-yl)(5-iodo-2-methylphenyl)methanone** (126.7 mg, 0.30 mmol, 1.5 equiv), the title compound was prepared following the general procedure **A** using $Ni(ClO_4)_2 \cdot 6H_2O$ (3.6 mg, 5.0 mol%), **L1** (7.2 mg, 6.0 mol%), NaF (16.8 mg, 2.0 equiv), (*E*)-*N*-(prop-1-en-1-yl)benzamide (**1a**) (32.2 mg, 0.20 mmol), $(MeO)_3SiH$ (51.0 µL, 0.40 mmol), anhydrous DMAc (2.0 mL). The reaction mixture was stirred for 48 h at rt. The crude material was purified by flash column chromatography (0–15% EtOAc in PE) to provide the title compound as a white solid in 73% yield (67.2 mg).

Rf 0.3 (20% EtOAc in PE), UV;

^{1}H NMR (500 MHz, $CDCl_3$) δ 7.81 (d, *J* = 7.4 Hz, 2H), 7.61 – 7.55 (m, 2H), 7.48 (s, 2H), 7.44 – 7.37 (m, 3H), 7.35 (s, 1H), 7.26 (d, *J* = 7.4 Hz, 1H), 7.16 (s, 1H), 7.10 (t, *J* = 8.3 Hz, 2H), 6.79 (d, *J* = 7.4 Hz, 1H), 5.09 (q, *J* = 7.1 Hz, 1H), 2.38 (s, 3H), 2.00 – 1.84 (m, 2H), 0.97 (t, *J* = 7.0 Hz, 3H);

^{13}C NMR (126 MHz, $CDCl_3$) δ 190.1, 167.0, 163.3 (d, *J* = 250.2 Hz), 152.8, 143.2, 139.7, 138.2, 136.8, 135.5, 134.5, 131.5 (d, *J* = 10.8 Hz), 129.6 (d, *J* = 3.5 Hz), 128.9, 128.7, 128.2 (d, *J* = 8.3 Hz), 127.0, 126.2, 124.1, 116.3, 116.2, 55.0, 29.2, 19.3, 10.9;

^{19}F NMR (471 MHz, $CDCl_3$) δ –111.5;

HRMS (ESI) calcd. for $C_{28}H_{25}FNO_2S$ $[M+H]^+$ *m/z* 458.1585, found 458.1582;
IR (neat, cm^{-1}) 3293, 2971, 1635, 1057;
m.p. 132.8 – 133.1 °C;
$[\alpha]_D^{23}$ = +15.5 (*c* = 3.0, $CHCl_3$); 99% *ee*;
HPLC analysis CHIRALCEL AD-H column, 30% ^{i}PrOH in hexane, 1.0 mL/min, 220 nm UV detector, t_R (minor) = 7.3 min, t_R (major) = 13.4 min.

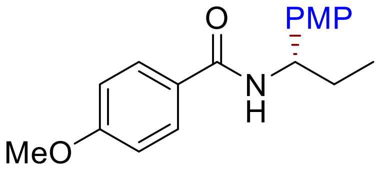

(*S*)-4-Methoxy-*N*-(1-(4-methoxyphenyl)propyl)benzamide (Fig. 4, **4b**). From **(*Z*)-4-Methoxy-*N*-(prop-1-en-1-yl)benzamide** (38.2 mg, 0.20 mmol), the title compound was prepared following the general procedure **A** using NiI_2 (3.1 mg, 5.0 mol%), **L1** (7.2 mg, 6.0 mol%), NaF (16.8 mg, 2.0 equiv), 4-iodoanisole (**2a**) (84.0 mg, 0.36 mmol, 1.8 equiv), $(MeO)_3SiH$ (51.0 μL, 0.40 mmol), anhydrous DMAc (1.0 mL). The reaction mixture was stirred for 48 h at rt. The crude material was purified by flash column chromatography (0–10% EtOAc in PE) to provide the title compound as a white solid in 63% yield (37.5 mg).
Rf 0.2 (20% EtOAc in PE), UV;
^{1}H NMR (500 MHz, $CDCl_3$) δ 7.75 (d, *J* = 8.7 Hz, 2H), 7.29 (d, *J* = 8.8 Hz, 2H), 6.94 – 6.86 (m, 4H), 6.33 (d, *J* = 7.9 Hz, 1H), 5.04 (q, *J* = 7.5 Hz, 1H), 3.85 (s, 3H), 3.81 (s, 3H), 2.01 – 1.86 (m, 2H), 0.95 (t, *J* = 7.4 Hz, 3H);
^{13}C NMR (126 MHz, $CDCl_3$) δ 166.2, 162.1, 158.8, 134.4, 128.7, 127.9, 127.0, 114.0, 113.6, 55.4, 55.3, 54.7, 29.1, 10.9;
HRMS (ESI) calcd. for $C_{18}H_{22}NO_3$ $[M+H]^+$ *m/z* 300.1594, found 300.1593;
IR (neat, cm^{-1}) 3331, 2956, 1624, 1502, 1238;
m.p. 173.4 – 173.9 °C;
$[\alpha]_D^{23}$ = –1.2 (*c* = 1.0, $CHCl_3$); 99% *ee*;
HPLC analysis CHIRALCEL AD-H column, 20% EtOH in hexane, 1.0 mL/min, 220 nm UV detector, t_R (minor) = 4.8 min, t_R (major) = 5.4 min.

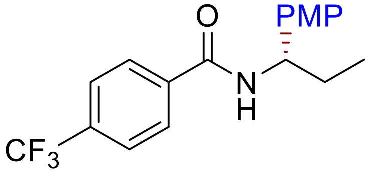

(*S*)-*N*-(1-(4-Methoxyphenyl)propyl)-4-(trifluoromethyl)benzamide (Fig. 4, **4c**). From ***N*-(Prop-1-en-1-yl)-4-(trifluoromethyl)benzamide** (*Z:E* 6.6:1, 45.8 mg, 0.20 mmol), the title compound was prepared following the general procedure **A** using NiI_2 (3.1 mg, 5.0 mol%), **L1** (7.2 mg, 6.0 mol%), NaF (16.8 mg, 2.0 equiv), 4-iodoanisole (**2a**) (84.0 mg, 0.36 mmol, 1.8 equiv), $(MeO)_3SiH$ (51.0 μL, 0.40 mmol), anhydrous DMAc (1.0 mL). The reaction mixture was stirred for 48 h at rt. The crude material was purified by flash column chromatography (0–7% EtOAc in PE) to provide the title compound as a white solid in 88% yield (59.7 mg).
Rf 0.2 (10% EtOAc in PE), UV;

^{1}H NMR (500 MHz, $CDCl_3$) δ 7.84 (d, *J* = 8.0 Hz, 2H), 7.62 (d, *J* = 8.0 Hz, 2H), 7.28 (d, *J* = 8.6 Hz, 2H), 6.88 (d, *J* = 8.6 Hz, 2H), 6.73 (d, *J* = 8.0 Hz, 1H), 5.02 (q, *J* = 7.5 Hz, 1H), 3.80 (s, 3H), 2.03 – 1.94 (m, 1H), 1.94 – 1.84 (m, 1H), 0.95 (t, *J* = 7.4 Hz, 3H);
^{13}C NMR (126 MHz, $CDCl_3$) δ 165.6, 158.9, 138.0, 133.8, 133.0 (q, *J* = 32.8 Hz), 127.9, 127.5, 125.5 (q, *J* = 3.6 Hz), 123.7 (q, *J* = 272.4 Hz), 114.1, 55.3, 55.2, 28.9, 10.9;
^{19}F NMR (471 MHz, $CDCl_3$) δ – 62.9;
HRMS (ESI) calcd. for $C_{18}H_{19}F_3NO_2$ $[M+H]^+$ *m/z* 338.1362, found 338.1361;
IR (neat, cm^{-1}) 3330, 2930, 1624, 1513, 1027;
m.p. 143.5 – 144.2 °C;
$[\alpha]_D^{23}$ = –21.7 (*c* = 2.0, $CHCl_3$); 98% *ee*;
HPLC analysis CHIRALCEL AD-H column, 10% EtOH in hexane, 1.0 mL/min, 220 nm UV detector, t_R (minor) = 9.7 min, t_R (major) = 15.1 min.

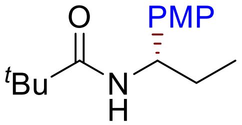

(*S*)-*N*-(1-(4-Methoxyphenyl)propyl)pivalamide (Fig. 4, **4d**). From **(*Z*)-*N*-(Prop-1-en-1-yl)pivalamide** (28.2 mg, 0.20 mmol), the title compound was prepared following the general procedure **A** using NiI_2 (3.1 mg, 5.0 mol%), **L1** (7.2 mg, 6.0 mol%), NaF (16.8 mg, 2.0 equiv), 4-iodoanisole (**2a**) (84.0 mg, 0.36 mmol, 1.8 equiv), $(MeO)_3SiH$ (51.0 µL, 0.40 mmol), anhydrous DMAc (1.0 mL). The reaction mixture was stirred for 48 h at rt. The crude material was purified by flash column chromatography (0–10% EtOAc in PE) to provide the title compound as a white solid in 55% yield (27.5 mg).
Rf 0.2 (20% EtOAc in PE), UV;
^{1}H NMR (500 MHz, $CDCl_3$) δ 7.20 (d, *J* = 8.6 Hz, 2H), 6.88 (d, *J* = 8.6 Hz, 2H), 5.80 (d, *J* = 8.0 Hz, 1H), 4.83 (q, *J* = 7.5 Hz, 1H), 3.81 (s, 3H), 1.86 – 1.77 (m, 2H), 1.21 (s, 9H), 0.89 (t, *J* = 7.4 Hz, 3H);
^{13}C NMR (126 MHz, $CDCl_3$) δ 177.5, 158.6, 134.5, 127.6, 113.9, 55.2, 54.1, 38.7, 29.1, 27.6, 10.7;
HRMS (ESI) calcd. for $C_{15}H_{24}O_2$ $[M+H]^+$ *m/z* 250.1802, found 250.1801;
IR (neat, cm^{-1}) 3309, 2928, 1635, 1512, 691;
m.p. 166.2 – 167.4 °C;
$[\alpha]_D^{23}$ = –116.6 (*c* = 1.0, $CHCl_3$); 95% *ee*;
HPLC analysis CHIRALCEL AD-H column, 10% ^{i}PrOH in hexane, 1.0 mL/min, 220 nm UV detector, t_R (minor) = 5.5 min, t_R (major) = 7.5 min.

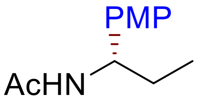

(*S*)-*N*-(1-(4-Methoxyphenyl)propyl)acetamide (Fig. 4, **4e**). From **(*Z*)-*N*-(Prop-1-en-1-yl)acetamide** (19.8 mg, 0.20 mmol), the title compound was prepared following the general procedure **A** using NiI_2 (3.1 mg, 5.0 mol%), **L1** (7.2 mg, 6.0 mol%), NaF (16.8 mg, 2.0 equiv), 4-iodoanisole (**2a**) (84.0 mg, 0.36 mmol, 1.8 equiv), $(MeO)_3SiH$ (51.0 µL, 0.40 mmol), anhydrous DMAc (1.0 mL). The reaction mixture was stirred for 48 h at rt. The crude material was purified by flash column chromatography (0–20% EtOAc in PE) to provide the title compound as a white solid in 81% yield (33.7 mg).

Rf 0.4 (50% EtOAc in PE), UV;
^{1}H NMR (500 MHz, $CDCl_3$) δ 7.21 (d, *J* = 8.6 Hz, 2H), 6.87 (d, *J* = 8.7 Hz, 2H), 5.97 (d, *J* = 7.9 Hz, 1H), 4.83 (q, *J* = 7.7 Hz, 1H), 3.80 (s, 3H), 1.98 (s, 3H), 1.89 – 1.72 (m, 2H), 0.88 (t, *J* = 7.4 Hz, 3H);
^{13}C NMR (126 MHz, $CDCl_3$) δ 169.3, 158.8, 134.2, 127.8, 113.9, 55.3, 54.5, 28.9, 23.4, 10.8;
HRMS (ESI) calcd. for $C_{12}H_{18}NO_2$ $[M+H]^+$ *m/z* 208.1332, found 208.1331;
IR (neat, cm^{-1}) 3308, 2927, 1637, 1512, 1246;
m.p. 100.2 – 101.4 °C;
$[\alpha]_D^{23}$ = –184.5 (*c* = 0.8, $CHCl_3$); 97% *ee*;
HPLC analysis CHIRALCEL AD-H column, 10% ^{i}PrOH in hexane, 0.8 mL/min, 220 nm UV detector, t_R (minor) = 8.2 min, t_R (major) = 10.4 min.

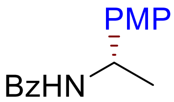

(*S*)-*N*-(1-(4-Methoxyphenyl)ethyl)benzamide (Fig. 4, **4f**). From ***N*-Vinylbenzamide** (29.4 mg, 0.20 mmol), the title compound was prepared following the general procedure **A** using NiI_2 (3.1 mg, 5.0 mol%), **L1** (7.2 mg, 6.0 mol%), NaF (16.8 mg, 2.0 equiv), 4-iodoanisole (**2a**) (84.0 mg, 0.36 mmol, 1.8 equiv), $(MeO)_3SiH$ (51.0 µL, 0.40 mmol), anhydrous DMAc (1.0 mL). The reaction mixture was stirred for 48 h at rt. The crude material was purified by flash column chromatography (0–10% EtOAc in PE) to provide the title compound as a white solid in 61% yield (31.0 mg).
Rf 0.2 (10% EtOAc in PE), UV;
^{1}H NMR (500 MHz, $CDCl_3$) δ 7.78 (d, *J* = 7.3 Hz, 2H), 7.50 (t, *J* = 7.4 Hz, 1H), 7.42 (t, *J* = 7.5 Hz, 2H), 7.34 (d, *J* = 8.5 Hz, 2H), 6.90 (d, *J* = 8.7 Hz, 2H), 6.45 (d, *J* = 7.9 Hz, 1H), 5.31 (p, *J* = 7.0 Hz, 1H), 3.82 (s, 3H), 1.60 (d, *J* = 6.9 Hz, 3H);
^{13}C NMR (126 MHz, $CDCl_3$) δ 166.6, 158.9, 135.3, 134.6, 131.4, 128.5, 127.5, 126.9, 114.1, 55.3, 48.7, 21.6;
HRMS (ESI) calcd. for $C_{16}H_{18}NO_2$ $[M+H]^+$ *m/z* 256.1332, found 256.1333;
IR (neat, cm^{-1}) 3349, 1632, 1515, 1251, 692;
m.p. 136.5 – 137.7 °C;
$[\alpha]_D^{23}$ = –32.0 (*c* = 1.6, $CHCl_3$); 97% *ee*;
HPLC analysis CHIRALCEL OD-H column, 10% EtOH in hexane, 1.0 mL/min, 220 nm UV detector, t_R (minor) = 7.4 min, t_R (major) = 9.8 min.

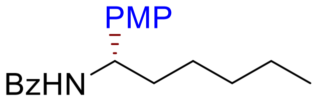

(*S*)-*N*-(1-(4-Methoxyphenyl)hexyl)benzamide (Fig. 4, **4g**). From **(*E*)-*N*-(Hex-1-en-1-yl)benzamide** (40.6 mg, 0.20 mmol), the title compound was prepared following the general procedure **A** using NiI_2 (3.1 mg, 5.0 mol%), **L1** (7.2 mg, 6.0 mol%), NaF (16.8 mg, 2.0 equiv), 4-iodoanisole (**2a**) (84.0 mg, 0.36 mmol, 1.8 equiv), $(MeO)_3SiH$ (51.0 µL, 0.40 mmol), anhydrous DMAc (1.0 mL). The reaction mixture was stirred for 48 h at rt. The crude material was purified by flash column chromatography (0–7% EtOAc in PE) to provide the title compound as a white solid in 88% yield (54.5 mg).
Rf 0.2 (10% EtOAc in PE), UV;

^{1}H NMR (500 MHz, $CDCl_3$) δ 7.78 (d, *J* = 7.5 Hz, 2H), 7.49 (t, *J* = 7.3 Hz, 1H), 7.41 (t, *J* = 7.6 Hz, 2H), 7.33 – 7.28 (m, 2H), 6.89 (d, *J* = 8.6 Hz, 2H), 6.48 (d, *J* = 8.1 Hz, 1H), 5.13 (q, *J* = 7.6 Hz, 1H), 3.81 (s, 3H), 2.00 – 1.82 (m, 2H), 1.38 – 1.25 (m, 6H), 0.89 (t, *J* = 7.4 Hz, 3H);
^{13}C NMR (126 MHz, $CDCl_3$) δ 166.7, 158.8, 134.7, 134.6, 131.3, 128.5, 127.8, 126.9, 114.1, 55.2, 53.4, 36.1, 31.6, 26.1, 22.5, 14.0;
HRMS (ESI) calcd. for $C_{20}H_{26}NO_2$ $[M+H]^+$ *m/z* 312.1958, found 312.1959;
IR (neat, cm^{-1}) 3337, 2928, 1630, 1514, 1250, 690;
m.p. 100.2 – 101.3 °C;
$[\alpha]_D^{23}$ = –26.7 (*c* = 2.5, $CHCl_3$); 99% *ee*;
HPLC analysis CHIRALCEL AD-H column, 8% EtOH in hexane, 1.0 mL/min, 220 nm UV detector, t_R (minor) = 13.5 min, t_R (major) = 19.3 min.

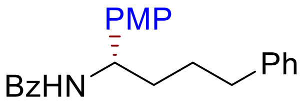

(*S*)-*N*-(1-(4-Methoxyphenyl)-4-phenylbutyl)benzamide (Fig. 4, **4h**). From **(*E*)-*N*-(4-Phenylbut-1-en-1-yl)benzamide** (50.2 mg, 0.20 mmol), the title compound was prepared following the general procedure **A** using NiI_2 (3.1 mg, 5.0 mol%), **L1** (7.2 mg, 6.0 mol%), NaF (16.8 mg, 2.0 equiv), 4-iodoanisole (**2a**) (84.0 mg, 0.36 mmol, 1.8 equiv), $(MeO)_3SiH$ (51.0 µL, 0.40 mmol), anhydrous DMAc (1.0 mL). The reaction mixture was stirred for 48 h at rt. The crude material was purified by flash column chromatography (0–1% EtOAc in PE) to provide the title compound as a white solid in 73% yield (59.7 mg). ***Note:*** from **(*Z*)-*N*-(4-Phenylbut-1-en-1-yl)benzamide**, the yield of the title compound was 88% (63.1 mg).
Rf 0.4 (20% EtOAc in PE), UV;
^{1}H NMR (500 MHz, $CDCl_3$) δ 7.78 (d, *J* = 7.4 Hz, 2H), 7.50 (t, *J* = 7.3 Hz, 1H), 7.41 (t, *J* = 7.6 Hz, 2H), 7.33 – 7.26 (m, 4H), 7.26 – 7.15 (m, 3H), 6.90 (d, *J* = 8.6 Hz, 2H), 6.51 (d, *J* = 8.2 Hz, 1H), 5.19 (q, *J* = 7.6 Hz, 1H), 3.81 (s, 3H), 2.68 (t, *J* = 7.6 Hz, 2H), 2.06 – 1.87 (m, 2H), 1.80 – 1.60 (m, 2H);
^{13}C NMR (126 MHz, $CDCl_3$) δ 166.7, 158.9, 142.0, 134.7, 134.3, 131.4, 128.5, 128.5, 128.4, 127.9, 126.9, 125.8, 114.1, 55.1, 53.3, 35.6, 28.1;
HRMS (ESI) calcd. for $C_{24}H_{26}NO_2$ $[M+H]^+$ *m/z* 360.1958, found 360.1957;
IR (neat, cm^{-1}) 2932, 1632, 1512, 693;
m.p. 143.6 – 145.0 °C;
$[\alpha]_D^{23}$ = –27.5 (*c* = 3.0, $CHCl_3$); 98% *ee*;
HPLC analysis CHIRALCEL AD-H column, 20% EtOH in hexane, 1.0 mL/min, 220 nm UV detector, t_R (minor) = 17.1 min, t_R (major) = 27.1 min.

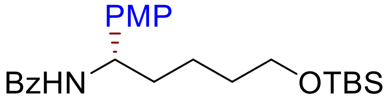

(*S*)-*N*-(5-((*tert*-Butyldimethylsilyl)oxy)-1-(4-methoxyphenyl)pentyl)benzamide (Fig. 4, **4i**). From **(*E*)-*N*-(5-((*tert*-Butyldimethylsilyl)oxy)pent-1-en-1-yl)benzamide** (63.8 mg, 0.20 mmol), the title compound was prepared following the general procedure **A** using NiI_2 (3.1 mg, 5.0 mol%), **L1** (7.2 mg, 6.0 mol%), NaF (16.8 mg, 2.0 equiv), 4-iodoanisole (**2a**) (84.0 mg, 0.36 mmol, 1.8 equiv), $(MeO)_3SiH$ (51.0 µL, 0.40 mmol),

anhydrous DMAc (1.0 mL). The reaction mixture was stirred for 48 h at rt. The crude material was purified by flash column chromatography (0–10% EtOAc in PE) to provide the title compound as a white solid in 72% yield (61.9 mg).

Rf 0.2 (10% EtOAc in PE), UV;

^{1}H NMR (500 MHz, $CDCl_3$) δ 7.77 (d, *J* = 7.3 Hz, 2H), 7.49 (t, *J* = 7.4 Hz, 1H), 7.42 (t, *J* = 7.6 Hz, 2H), 7.30 (d, *J* = 8.6 Hz, 2H), 6.89 (d, *J* = 8.6 Hz, 2H), 6.39 (d, *J* = 8.0 Hz, 1H), 5.13 (q, *J* = 7.6 Hz, 1H), 3.81 (s, 3H), 3.60 (t, *J* = 6.4 Hz, 2H), 2.02 – 1.94 (m, 1H), 1.93 – 1.86 (m, 1H), 1.62 – 1.53 (m, 2H), 1.52 – 1.41 (m, 1H), 1.41 – 1.32 (m, 1H), 0.88 (s, 9H), 0.04 (s, 6H);

^{13}C NMR (126 MHz, $CDCl_3$) δ 166.6, 158.8, 134.7, 134.4, 131.4, 128.5, 127.8, 126.9, 114.1, 62.9, 55.3, 35.9, 32.5, 25.9, 22.7, 18.3, –5.2, –5.3;

HRMS (ESI) calcd. for $C_{25}H_{38}NO_3Si$ $[M+H]^+$ *m/z* 428.2615, found 428.2614;

IR (neat, cm^{-1}) 3326, 1512, 1244, 692;

m.p. 110.7 – 111.4 °C;

$[\alpha]_D^{23}$ = –23.1 (*c* = 2.0, $CHCl_3$); 98% *ee*;

HPLC analysis CHIRALCEL AD-H column, 10% ^{i}PrOH in hexane, 1.0 mL/min, 220 nm UV detector, t_R (minor) = 7.0 min, t_R (major) = 11.7 min.

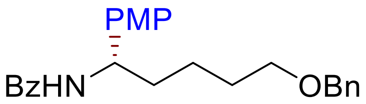

(*S*)-*N*-(5-(Benzyloxy)-1-(4-methoxyphenyl)pentyl)benzamide (Fig. 4, **4j**). From **(*E*)-*N*-(5-(Benzyloxy)pent-1-en-1-yl)benzamide** (59.0 mg, 0.20 mmol), the title compound was prepared following the general procedure **A** using NiI_2 (3.1 mg, 5.0 mol%), **L1** (7.2 mg, 6.0 mol%), NaF (16.8 mg, 2.0 equiv), 4-iodoanisole (**2a**) (84.0 mg, 0.36 mmol, 1.8 equiv), $(MeO)_3SiH$ (51.0 μL, 0.40 mmol), anhydrous DMAc (1.0 mL). The reaction mixture was stirred for 48 h at rt. The crude material was purified by flash column chromatography (0–15% EtOAc in PE) to provide the title compound as a white solid in 82% yield (65.8 mg).

Rf 0.3 (20% EtOAc in PE), UV;

^{1}H NMR (500 MHz, $CDCl_3$) δ 7.78 (d, *J* = 7.6 Hz, 2H), 7.49 (t, *J* = 7.4 Hz, 1H), 7.41 (t, *J* = 7.5 Hz, 2H), 7.37 – 7.29 (m, 7H), 6.89 (d, *J* = 8.3 Hz, 2H), 6.54 (d, *J* = 8.0 Hz, 1H), 5.14 (q, *J* = 7.6 Hz, 1H), 4.50 (s, 2H), 3.81 (s, 3H), 3.48 (t, *J* = 6.4 Hz, 2H), 2.03 – 1.86 (m, 2H), 1.70 (q, *J* = 7.4 Hz, 2H), 1.57 – 1.47 (m, 1H), 1.47 – 1.37 (m, 1H);

^{13}C NMR (126 MHz, $CDCl_3$) δ 166.7, 158.8, 138.6, 134.7, 134.5, 131.4, 128.5, 128.4, 127.8, 127.7, 127.6, 126.9, 114.1, 72.9, 70.1, 55.3, 53.4, 35.9, 29.4, 23.2;

HRMS (ESI) calcd. for $C_{26}H_{30}NO_3$ $[M+H]^+$ *m/z* 404.2220, found 404.2221;

IR (neat, cm^{-1}) 3336, 2930, 1629, 1514, 1251, 692;

m.p. 102.3 – 103.8 °C;

$[\alpha]_D^{23}$ = –18.4 (*c* = 3.0, $CHCl_3$); 99% *ee*;

HPLC analysis CHIRALCEL AD-H column, 20% EtOH in hexane, 1.0 mL/min, 220 nm UV detector, t_R (minor) = 10.4 min, t_R (major) = 13.5 min.

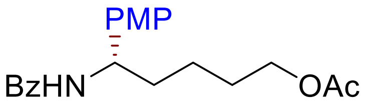

(*S*)-5-Benzamido-5-(4-methoxyphenyl)pentyl acetate (Fig. 4, **4k**). From **(*E*)-5-Benzamidopent-4-en-1-yl acetate** (49.4 mg, 0.20 mmol), the title compound was prepared following the general procedure **A** using NiI_2 (3.1 mg, 5.0 mol%), **L1** (7.2 mg, 6.0 mol%), NaF (16.8 mg, 2.0 equiv), 4-iodoanisole (**2a**) (84.0 mg, 0.36 mmol, 1.8 equiv), $(MeO)_3SiH$ (51.0 μL, 0.40 mmol), anhydrous DMAc (1.0 mL). The reaction mixture was stirred for 48 h at rt. The crude material was purified by flash column chromatography (0–30% EtOAc in PE) to provide the title compound as a white solid in 58% yield (41.1 mg).

Rf 0.5 (50% EtOAc in PE), UV;

^{1}H NMR (500 MHz, $CDCl_3$) δ 7.78 (d, *J* = 7.3 Hz, 2H), 7.50 (t, *J* = 7.3 Hz, 1H), 7.43 (t, *J* = 7.5 Hz, 2H), 7.30 (d, *J* = 8.6 Hz, 2H), 6.90 (d, *J* = 8.6 Hz, 2H), 6.38 (d, *J* = 8.0 Hz, 1H), 5.14 (q, *J* = 7.6 Hz, 1H), 4.11 – 3.99 (m, 2H), 3.81 (s, 3H), 2.02 (s, 4H), 1.95 – 1.86 (m, 1H), 1.69 (p, *J* = 7.7, 7.1 Hz, 2H), 1.50 – 1.34 (m, 2H);

^{13}C NMR (126 MHz, $CDCl_3$) δ 171.3, 166.7, 158.9, 134.6, 134.1, 131.5, 128.6, 127.8, 126.9, 114.1, 64.0, 55.3, 53.2, 35.6, 28.3, 22.7, 21.0;

HRMS (ESI) calcd. for $C_{21}H_{26}NO_4$ $[M+H]^+$ *m/z* 356.1856, found 356.1857;

IR (neat, cm^{-1}) 3336, 2930, 1735, 1632, 1518, 691;

m.p. 119.6 – 120.4 °C;

$[\alpha]_D^{23}$ = –23.8 (*c* = 1.0, $CHCl_3$); 94% *ee*;

HPLC analysis CHIRALCEL AD-H column, 20% EtOH in hexane, 1.0 mL/min, 220 nm UV detector, t_R (minor) = 5.5 min, t_R (major) = 6.3 min.

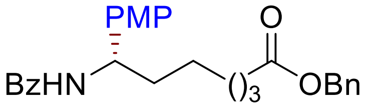

Benzyl (*S*)-7-benzamido-7-(4-methoxyphenyl)heptanoate (Fig. 4, **4l**). From **Benzyl (*E*)-7-benzamidohept-6-enoate** (67.4 mg, 0.20 mmol), the title compound was prepared following the general procedure **A** using NiI_2 (3.1 mg, 5.0 mol%), **L1** (7.2 mg, 6.0 mol%), NaF (16.8 mg, 2.0 equiv), 4-iodoanisole (**2a**) (84.0 mg, 0.36 mmol, 1.8 equiv), $(MeO)_3SiH$ (51.0 μL, 0.40 mmol), anhydrous DMAc (1.0 mL). The reaction mixture was stirred for 48 h at rt. The crude material was purified by flash column chromatography (0–20% EtOAc in PE) to provide the title compound as a white solid in 42% yield (37.4 mg).

Rf 0.5 (50% EtOAc in PE), UV;

^{1}H NMR (500 MHz, $CDCl_3$) δ 7.77 (d, *J* = 7.6 Hz, 2H), 7.50 (t, *J* = 7.1 Hz, 1H), 7.43 (t, *J* = 7.5 Hz, 2H), 7.40 – 7.34 (m, 5H), 7.29 (d, *J* = 8.1 Hz, 2H), 6.90 (d, *J* = 8.4 Hz, 2H), 6.33 (d, *J* = 8.1 Hz, 1H), 5.16 – 5.08 (m, 3H), 3.82 (d, *J* = 1.1 Hz, 3H), 2.35 (t, *J* = 7.5 Hz, 2H), 1.98 – 1.91 (m, 1H), 1.89 – 1.84 (m, 1H), 1.64 (q, *J* = 7.3 Hz, 2H), 1.42 – 1.36 (m, 3H), 1.33 – 1.27 (m, 1H);

^{13}C NMR (126 MHz, $CDCl_3$) δ 173.5, 166.6, 158.9, 136.1, 134.7, 134.2, 131.5, 128.6, 128.2, 127.8, 126.9, 114.1, 66.1, 55.3, 53.3, 35.9, 34.2, 28.9, 26.0, 24.7;

HRMS (ESI) calcd. for $C_{28}H_{32}NO_4$ $[M+H]^+$ *m/z* 446.2326, found 446.2327;

IR (neat, cm^{-1}) 3362, 1722, 1634, 1515, 1244, 692;

m.p. 67.2 – 67.9 °C;

$[\alpha]_D^{23}$ = –17.5 (*c* = 0.8, $CHCl_3$); 98% *ee*;

HPLC analysis CHIRALCEL OD-H column, 10% EtOH in hexane, 1.0 mL/min, 220 nm UV detector, t_R (minor) = 14.7 min, t_R (major) = 22.1 min.

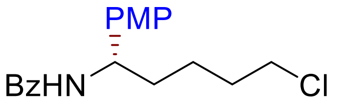

(*S*)-*N*-(5-Chloro-1-(4-methoxyphenyl)pentyl)benzamide (Fig. 4, **4m**). From **(*E*)-*N*-(5-Chloropent-1-en-1-yl)benzamide** (44.6 mg, 0.20 mmol), the title compound was prepared following the general procedure **A** using NiI_2 (3.1 mg, 5.0 mol%), **L1** (7.2 mg, 6.0 mol%), NaF (16.8 mg, 2.0 equiv), 4-iodoanisole (**2a**) (84.0 mg, 0.36 mmol, 1.8 equiv), $(MeO)_3SiH$ (51.0 µL, 0.40 mmol), anhydrous DMAc (1.0 mL). The reaction mixture was stirred for 48 h at rt. The crude material was purified by flash column chromatography (0–15% EtOAc in PE) to provide the title compound as a white solid in 67% yield (44.2 mg).

Rf 0.3 (20% EtOAc in PE), UV;

^{1}H NMR (500 MHz, $CDCl_3$) δ 7.77 (d, *J* = 7.3 Hz, 2H), 7.50 (t, *J* = 7.4 Hz, 1H), 7.42 (t, *J* = 7.6 Hz, 2H), 7.30 (d, *J* = 8.6 Hz, 2H), 6.93 – 6.87 (m, 2H), 6.47 (d, *J* = 7.3 Hz, 1H), 5.13 (q, *J* = 7.6 Hz, 1H), 3.81 (s, 3H), 3.58 – 3.48 (m, 2H), 2.05 – 1.95 (m, 1H), 1.95 – 1.76 (m, 3H), 1.61 – 1.51 (m, 1H), 1.51 – 1.38 (m, 1H);

^{13}C NMR (126 MHz, $CDCl_3$) δ 166.7, 158.9, 134.6, 133.9, 131.5, 128.6, 127.8, 126.9, 114.2, 55.3, 53.2, 44.7, 35.2, 32.2, 23.7;

HRMS (ESI) calcd. for $C_{19}H_{23}ClNO_2$ $[M+H]^+$ *m/z* 332.1412, found 332.1413;

IR (neat, cm^{-1}) 3323, 1633, 1513, 692;

m.p. 104.3 – 105.1 °C;

$[\alpha]_D^{23}$ = –23.8 (*c* = 2.0, $CHCl_3$); 98% *ee*;

HPLC analysis CHIRALCEL AD-H column, 20% EtOH in hexane, 1.0 mL/min, 220 nm UV detector, t_R (minor) = 9.1 min, t_R (major) = 11.6 min.

III. Synthesis Application

a) Gram scale and benchtop set up experiment

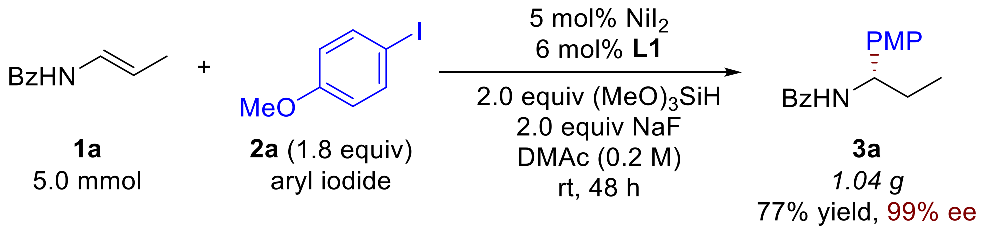

(*S*)-*N*-(1-(4-Methoxyphenyl)propyl)benzamide (Fig. 5, **3a**). The title compound was prepared following the general procedure **B** (out of glovebox): In air, NiI_2 (77.5 mg, 5.0 mol%; ***Note: hydroscopic, stored under nitrogen. Exposure to air should be less than 5 minutes***),), **L1** (190.0 mg, 6.0 mol%), NaF (420.0 mg, 2.0 equiv) (*E*)-*N*-(prop-1-en-1-yl)benzamide (**1a**) (806.0 mg, 5.00 mmol) and 4-iodoanisole (**2a**) (2.1 g, 9.0 mmol, 1.8 equiv) were added to an oven-dried 100 mL round-bottom flask equipped with a stir bar, The flask was evacuated and backfilled with nitrogen five times. Under nitrogen, anhydrous DMAc (25 mL) was added to the 100 mL round-bottom flask via a syringe, and the mixture was stirred vigorously for over 20 min at rt, $(MeO)_3SiH$ (1.27 mL, 10.0

mmol, ***Note: air-sensitive, stored under nitrogen. Exposure to air should be less than 5 minutes.***) was added to the resulting mixture in this order. The reaction mixture was stirred for 48 h at rt. After the reaction was complete, the reaction mixture was directly filtered through a short pad of silica gel (25% EtOAc in PE) to give the crude product. The crude material was purified by flash column chromatography (0–10% EtOAc in PE) to provide the title compound as a white solid in 77% yield (1.0382 g).

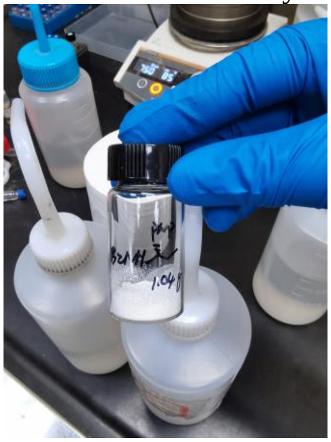

b) Further transformation

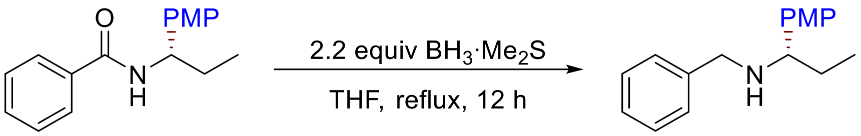

3a
0.5 mmol, 99% ee

5a
95% yield, 99% ee

(*S*)-*N*-Benzyl-1-(4-methoxyphenyl)propan-1-amine (Fig. 5, **5a**). To a solution of compound **3a** (134.6 mg, 0.5 mmol) in THF (1.0 mL), stirred in atmosphere of Argon, borane-dimethyl sulfide (0.55 mL, 1.1 mol) was added dropwise (evolution of hydrogen) and the reaction mixture was heated to reflux for 12 h. MeOH (5.0 mL) was added carefully and then the mixture was evaporated to a third of volume by distillation under ordinary pressure, to the distillation residue methanol hydrochloride, obtained by dissolution of acetyl chloride (0.5 mL) in MeOH (1.0 mL), was added and solvent was distilled under ordinary pressure. The distillation residue, which was triturated in ether, provided a white solid which was splitted in aqueous NaOH (20.0 mg of NaOH in 2 mL of water) and dichloromethane (20 mL). The organic phase dried on Na_2CO_3 and evaporated under reduced pressure, gave the title compound as a colorless oil in 95% yield (120.9 mg).

^{1}H NMR (500 MHz, $CDCl_3$) δ 7.36 – 7.26 (m, 7H), 6.92 (d, J = 8.7 Hz, 2H), 3.85 (s, 3H), 3.68 (d, J = 13.2 Hz, 1H), 3.56 (d, J = 13.2 Hz, 1H), 3.52 (dd, J = 8.0, 5.7 Hz, 1H), 1.83 – 1.75 (m, 1H), 1.70 – 1.62 (m, 1H), 0.82 (t, J = 7.4 Hz, 3H);

^{13}C NMR (126 MHz, $CDCl_3$) δ 158.6, 128.5, 128.4, 128.3, 126.8, 113.7, 63.5, 55.3, 51.4, 31.1, 10.8;

HRMS (ESI) calcd. for $C_{17}H_{22}NO$ $[M+H]^+$ *m/z* 256.1696, found 256.1695;

$[\alpha]_D^{23}$ = –40.6 (c = 1.0, $CHCl_3$); 99% *ee*;

HPLC analysis CHIRALCEL AD-H column, 10% ^{i}PrOH in hexane, 1.0 mL/min, 220 nm UV detector, t_R (minor) = 4.5 min, t_R (major) = 4.9 min.

IV. Isotopic Labelling Experiments

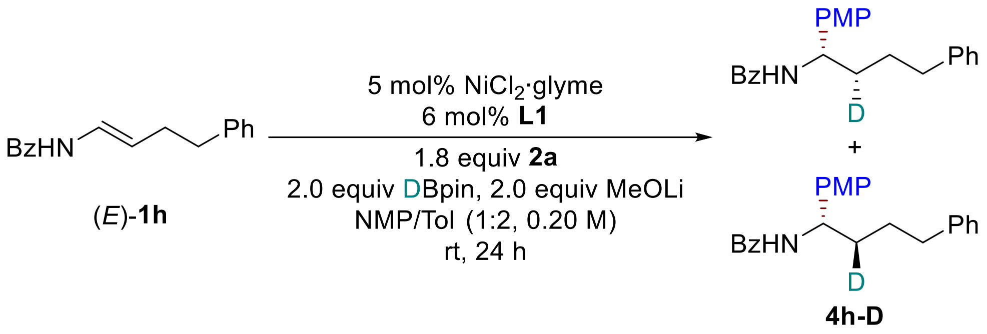

***N*-((1*S*,2*S*)-1-(4-Methoxyphenyl)-4-phenylbutyl-2-*d*)benzamide** (Fig. 5, **4h-D**). From **(*E*)-*N*-(4-Phenylbut-1-en-1-yl)benzamide** (25.1 mg, 0.10 mmol), the title compound was prepared following the general procedure **C** using $NiCl_2$·glyme (1.1 mg, 5.0 mol%), **L8** (3.6 mg, 6.0 mol%), LiOMe (7.6 mg, 2.0 equiv), 4-iodoanisole (**2a**) (42.0 mg, 0.18 mmol, 1.8 equiv), DBpin (29 μL, 0.20 mmol), anhydrous toluene (0.33 mL) and NMP (0.17 mL). The reaction mixture was stirred for 24 h at rt. The crude material was purified by flash column chromatography (0–10% EtOAc in PE) to provide the title compound as a white solid in 22% yield (7.8 mg) with 2.4:1 dr.

^{1}H NMR (500 MHz, $CDCl_3$) δ 7.76 (d, J = 7.4 Hz, 2H), 7.51 (t, J = 7.3 Hz, 1H), 7.43 (t, J = 7.5 Hz, 2H), 7.32 – 7.24 (m, 1H), 7.24 – 7.12 (m, 1H), 7.17 (d, J = 7.4 Hz, 2H), 6.90 (d, J = 8.5 Hz, 2H), 6.27 (d, J = 8.2 Hz, 1H), 5.17 (t, J = 7.7 Hz, 1H), 3.82 (s, 3H), 2.68 (t, J = 7.6 Hz, 2H), 2.01 – 1.95 (m, 0.8H), 1.92 – 1.87 (m, 0.34H), 1.77 – 1.71 (m, 1H), 1.67 – 1.62 (m, 1H);

^{13}C NMR (126 MHz, $CDCl_3$) δ 166.6, 158.9, 142.0, 134.6, 134.2, 131.5, 128.6, 128.5, 128.3, 127.8, 127.8, 126.9, 125.8, 114.1, 55.3, 53.2, 35.5, 27.9;

HRMS (ESI) calcd. for $C_{24}H_{25}DNO_2$ $[M+H]^+$ *m/z* 361.2021, found 361.2020;

IR (neat, cm^{-1}) 2932, 1632, 1512, 692;

m.p. 143.6 – 145.0 °C;

$[\alpha]_D^{23}$ = –36.0 (c = 0.25, $CHCl_3$); 96% *ee*;

HPLC analysis CHIRALCEL AD-H column, 20% ^{i}PrOH in hexane, 1.0 mL/min, 220 nm UV detector, t_R (minor) = 9.1 min, t_R (major) = 13.5 min.

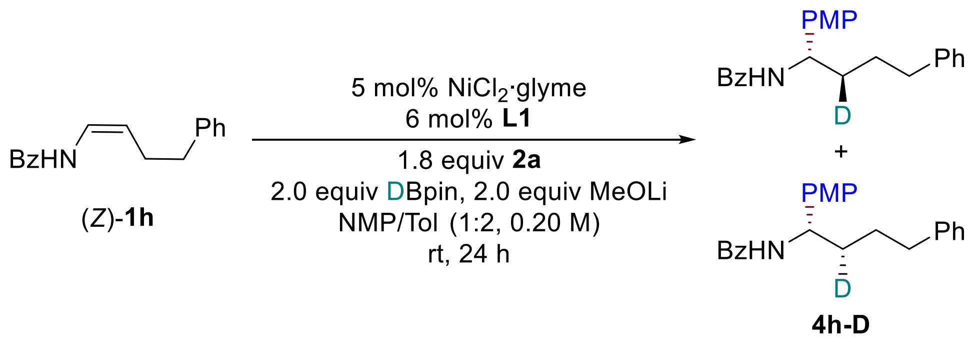

***N*-((1*S*,2*S*)-1-(4-Methoxyphenyl)-4-phenylbutyl-2-*d*)benzamide** (Fig. 5, **4h-D**). From **(*Z*)-*N*-(4-Phenylbut-1-en-1-yl)benzamide** (25.1 mg, 0.10 mmol), the title compound was prepared following the general procedure **C** using $NiCl_2$·glyme (1.1 mg, 5.0 mol%), **L8** (3.6 mg, 6.0 mol%), LiOMe (7.6 mg, 2.0 equiv), 4-iodoanisole (**2a**) (42.0 mg, 0.18 mmol, 1.8 equiv), DBpin (29 μL, 0.20 mmol), anhydrous toluene (0.33 mL) and NMP (0.17 mL). The reaction mixture was stirred for 24 h at rt. The crude material was purified by flash column chromatography (0–10% EtOAc in PE) to provide the title compound as a white solid in 25% yield (8.9 mg) with 1:2.3 dr.

^{1}H NMR (500 MHz, $CDCl_3$) δ 7.76 (d, *J* = 7.2 Hz, 2H), 7.51 (t, *J* = 7.4 Hz, 1H), 7.43 (t, *J* = 7.5 Hz, 2H), 7.30 – 7.25 (m, 4H), 7.22 – 7.15 (m, 3H), 6.90 (d, *J* = 8.7 Hz, 2H), 6.27 (d, *J* = 8.1 Hz, 1H), 5.17 (t, *J* = 7.7 Hz, 1H), 3.82 (s, 3H), 2.68 (t, *J* = 7.6 Hz, 2H), 2.01 – 1.95 (m, 0.34H), 1.92 – 1.87 (m, 0.78H), 1.77 – 1.71 (m, 1H), 1.68 – 1.62 (m, 1H).

^{13}C NMR (126 MHz, $CDCl_3$) δ 166.6, 158.9, 142.0, 134.6, 134.1, 131.4, 128.5, 128.5, 128.3, 127.8, 127.8, 126.9, 125.8, 114.1, 55.1, 53.2, 35.5, 27.9;

HRMS (ESI) calcd. for $C_{24}H_{25}DNO_2$ $[M+H]^+$ *m/z* 361.2021, found 361.2020;

IR (neat, cm^{-1}) 2932, 1632, 1512, 692;

m.p. 143.6 – 145.0 °C;

$[\alpha]_D^{23}$ = –32.7 (*c* = 0.3, $CHCl_3$); 96% *ee*;

HPLC analysis CHIRALCEL AD-H column, 20% ^{i}PrOH in hexane, 1.0 mL/min, 220 nm UV detector, t_R (minor) = 9.1 min, t_R (major) = 13.5 min.

V. Detailed Conditions Optimization

Supplementary Table 1. The effect of *N*-protected functional groups for NiH-catalysed asymmetric hydroarylation of *N*-acyl enamines

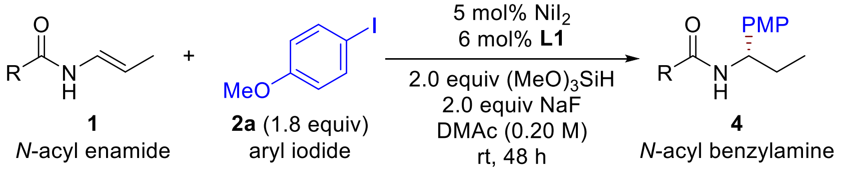

entry	R	yield of **4** (%)^a	rr^a	ee^b
1	Bz	80	>95:5	99
2	Ac	85	>95:5	97
3	Piv	62	>95:5	95
4	Boc	14	3:1	n.d.
5	Cbz	22	2.5:1	n.d.
6	Bn	trace	n.d.	n.d.
7	H	NR	n.d.	n.d.

[a] Yields and regioselectivities (rr) determined by crude ^{1}H NMR using 1,1,2,2-tetrachloroethane as the internal standard. [b] Enantioselectivity was determined by chiral HPLC analysis.

Supplementary Table 2. The effect of Nickel salts and base for NiH-catalysed asymmetric hydroarylation of *N*-acyl enamines

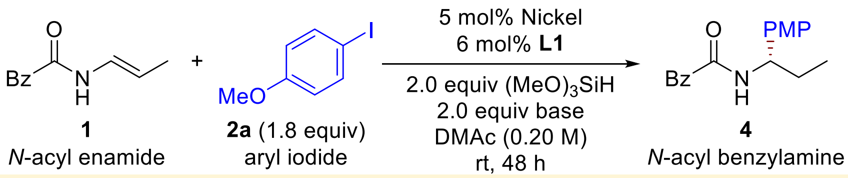

entry	Base	Nickel	yield[a]	ee[b]
1	Na_2CO_3	NiI_2	9	n.d.
2	K_2CO_3	NiI_2	trace	n.d.
3	$NaHCO_3$	NiI_2	11	99
4	$KHCO_3$	NiI_2	trace	n.d.
5	Na_3PO_4	NiI_2	9	n.d.
6	K_3PO_4	NiI_2	trace	n.d.
7	CsF	NiI_2	trace	n.d.
8	KF	NiI_2	8	n.d.
9	KF	$NiBr_2$	37	98
10	KF	$NiCl_2$	62	98
11	KF	$NiCl_2·6H_2O$	69	98
12	NaF	NiI_2	80	99
13	NaF	$NiBr_2$	65	98
14	NaF	$NiCl_2$	22	98
15	NaF	$NiCl_2·6H_2O$	38	98

[a] Yields and regioselectivities (rr) determined by crude ^{1}H NMR using 1,1,2,2-tetrachloroethane as the internal standard. [b] Enantioselectivity was determined by chiral HPLC analysis.

VI. Competition Experiment (difference steric environment of aryl iodide)

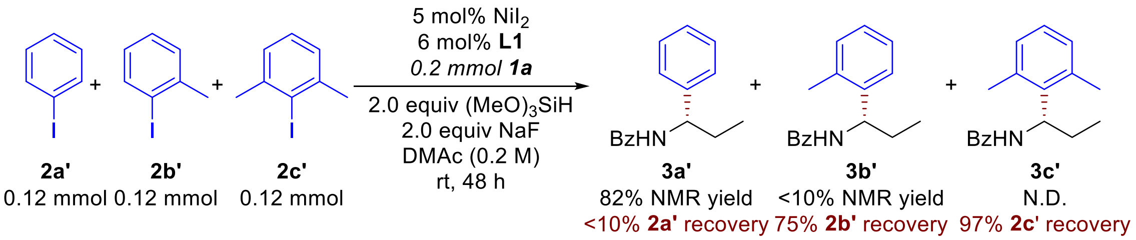

From **Iodobenzene** (**2a**') (13.4 μL, 0.12 mmol), **1-Iodo-2-methylbenzene** (**2b**') (15.2 μL, 0.12 mmol) and **2-Iodo-1,3-dimethylbenzene** (**2c**') (17.4 μL, 0.12 mmol). the title compound was prepared following the general procedure **A** using NiI_2 (3.1 mg, 5.0 mol%), **L1** (7.2 mg, 6.0 mol%), NaF (16.8 mg, 2.0 equiv), (*E*)-*N*-(prop-1-en-1-yl)benzamide (**1a**) (32.2 mg, 0.20 mmol), $(MeO)_3SiH$ (51.0 μL, 0.40 mmol), anhydrous DMAc (1.0 mL). The reaction mixture was stirred for 48 h at rt. After the reaction was complete, the reaction mixture was directly filtered through a short pad of silica gel (20% EtOAc in PE) to give the crude product. Dodecane (20 μL) was added as an internal standard for GC and GC-MS analysis.

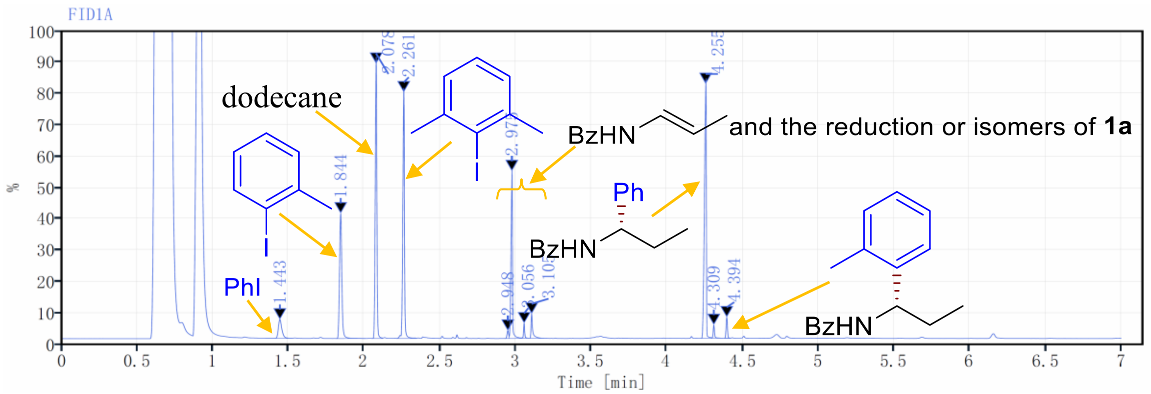

Signal: FID1A

RetTime [min]	Type	Width [min]	Area	Height	Area%	Name
1.443	BV	0.11	12.39	7.72	3.19	
1.844	BB	0.19	54.67	51.16	14.06	
2.078	BV	0.07	85.49	112.33	21.99	
2.261	BV	0.13	76.56	100.65	19.69	
2.948	BV	0.03	2.28	3.18	0.59	
2.975	VV	0.08	45.72	68.43	11.76	
3.056	VB	0.05	3.35	6.15	0.86	
3.105	BV	0.10	7.63	10.03	1.96	
4.255	BV	0.07	91.92	104.04	23.64	
4.309	VB	0.07	3.42	5.45	0.88	
4.394	BV	0.04	5.35	8.59	1.38	

Supplementary Fig. 1. GC analysis for competition experiment.

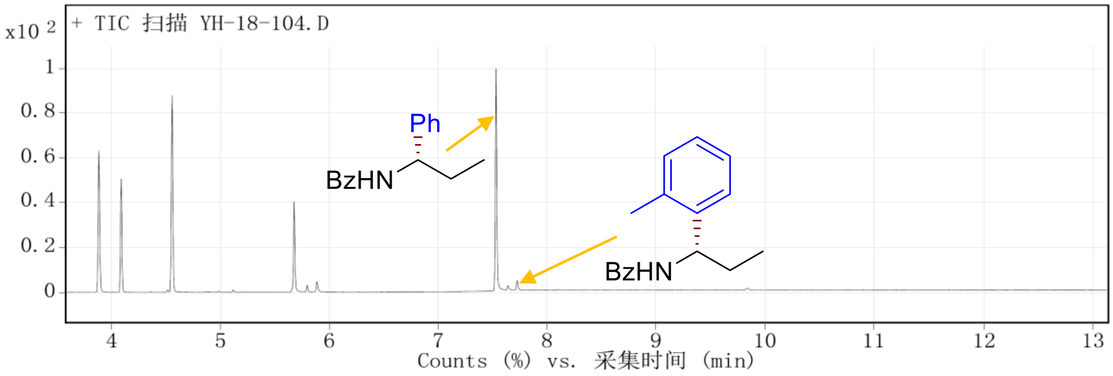

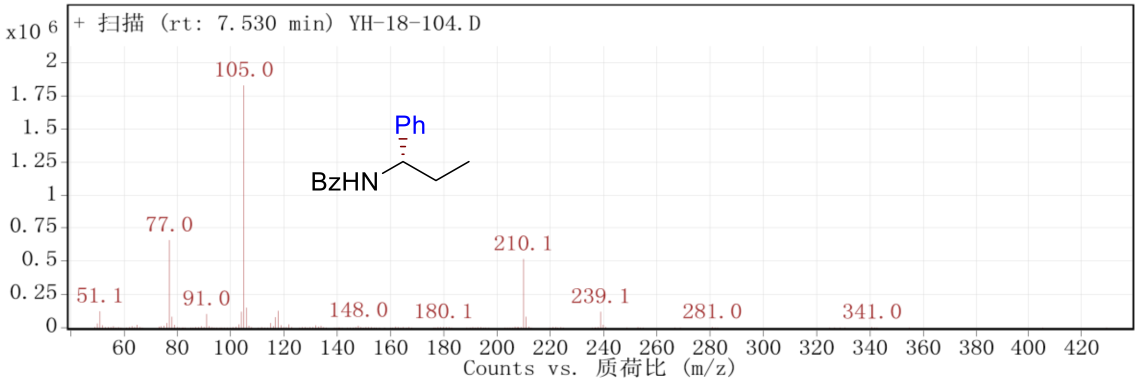

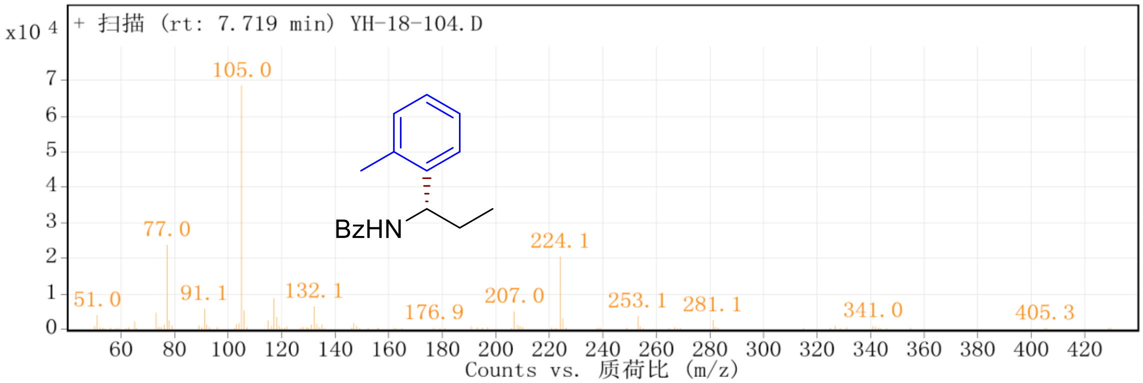

Supplementary Fig. 2. GC-MS analysis for competition experiment.

VII. Crystal Data and Structure Refinement for 4d

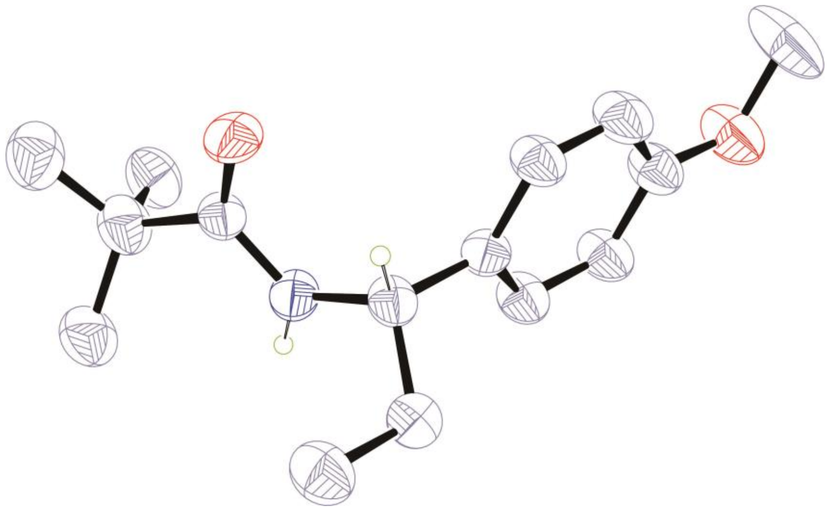

Supplementary Fig. 3. Crystal data and structure refinement for **4d**

Identification code	ZSL_a
Empirical formula	$C_{30}H_{46}N_2O_4$
Formula weight	498.69
Temperature/K	193.0
Crystal system	tetragonal
Space group	P43
a/Å	12.6082(11)
b/Å	12.6082(11)
c/Å	19.399(2)
α/°	90
β/°	90
γ/°	90
Volume/Å3	3083.9(7)
Z	4

ρcalcg/cm3 1.074
μ/mm-1 0.358
F(000) 1088.0
Crystal size/mm3 0.17 × 0.13 × 0.11
Radiation GaKα (λ = 1.34139)
2Θ range for data collection/° 6.098 to 107.84
Index ranges -15 ≤ h ≤ 15, -15 ≤ k ≤ 15, -23 ≤ l ≤ 22
Reflections collected 24428
Independent reflections 5522 [Rint = 0.0453, Rsigma = 0.0304]
Data/restraints/parameters 5522/49/335
Goodness-of-fit on F2 1.075
Final R indexes [I>=2σ (I)] R1 = 0.0758, wR2 = 0.2158
Final R indexes [all data] R1 = 0.0909, wR2 = 0.2387
Largest diff. peak/hole / e Å-3 0.51/-0.41
Flack parameter -0.03(12)

VIII. The Isomerization Processes of 1h

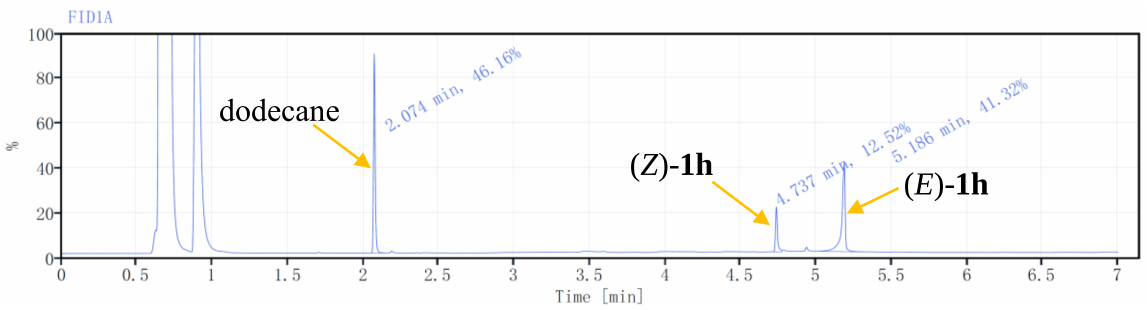

RetTime (min)	Signal	Width (min)	Area	Height	Area%
2.074	FID1A	0.099	23.3	29.8	46.16
4.737	FID1A	0.060	6.3	6.7	12.52
5.186	FID1A	0.292	20.9	13.2	41.32

Supplementary Fig. 4. GC analysis for (*E*)-**1h** under standard condition without ArI (quenched after 24h)

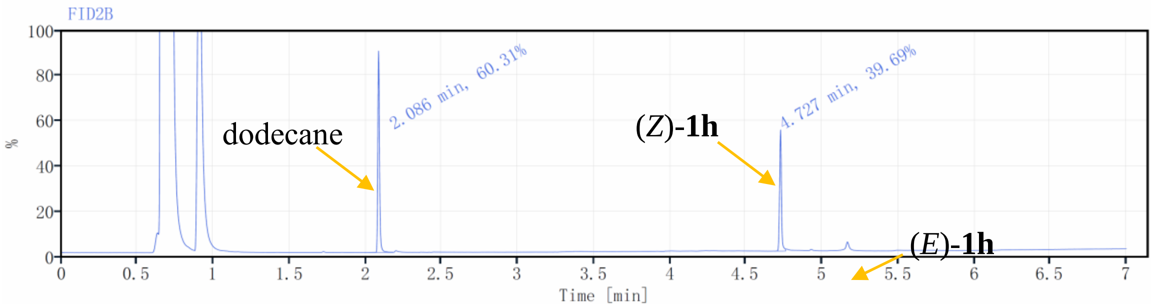

RetTime (min)	Signal	Width (min)	Area	Height	Area%
2.086	FID2B	0.158	23.5	30.2	60.31
4.727	FID2B	0.071	15.5	18.2	39.69

Supplementary Fig. 5. GC analysis for (*Z*)-**1h** under standard condition without ArI (quenched after 24h)

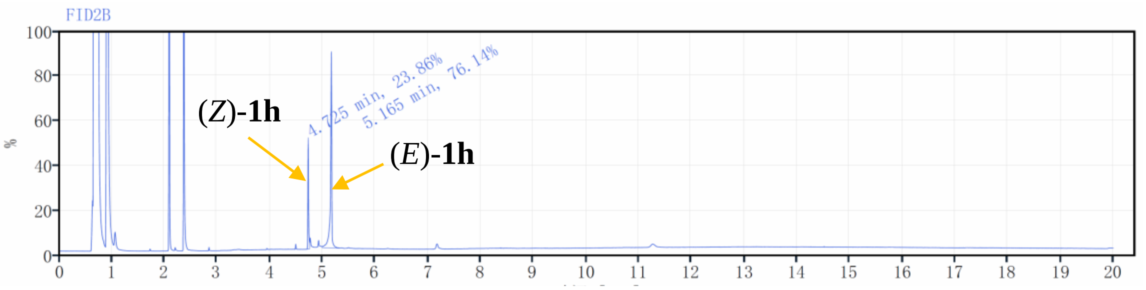

RetTime (min)	Signal	Width(min)	Area	Height	Area%
4.725	FID2B	0.059	7.3	8.1	23.86
5.165	FID2B	0.333	23.4	14.3	76.14

Supplementary Fig. 6. GC analysis for (*E*)-**1h** under standard condition (quenched after 12h)

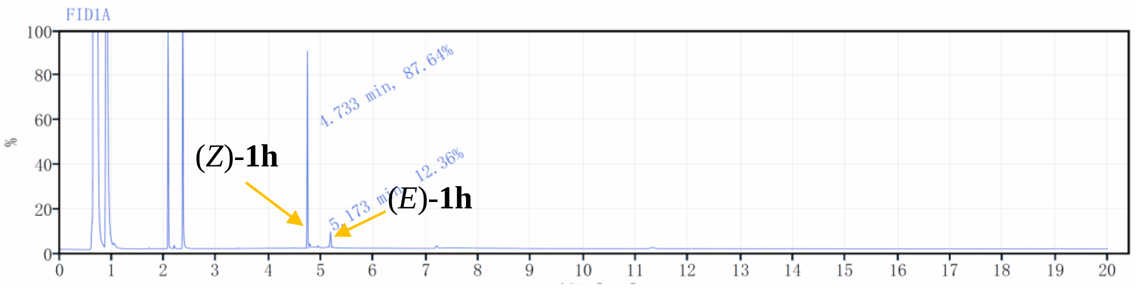

RetTime (min)	Signal	Width (min)	Area	Height	Area%
4.733	FID1A	0.072	14.8	18.7	87.64
5.173	FID1A	0.021	2.1	1.5	12.36

Supplementary Fig. 7. GC analysis for (*Z*)-**1h** under standard condition (quenched after 12h)

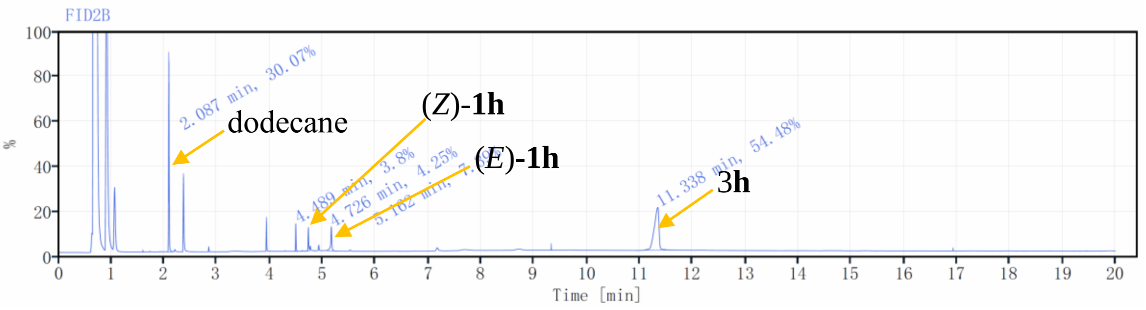

RetTime (min)	Signal	Width (min)	Area	Height	Area%
2.075	FID1A	0.110	21.3	26.9	30.36
4.497	FID1A	0.040	1.5	2.2	2.17
4.736	FID1A	0.084	2.4	2.7	3.35
5.177	FID1A	0.184	3.5	2.3	4.92
11.417	FID1A	0.566	41.6	6.4	59.19

Supplementary Fig. 8. GC analysis for (*E*)-**1h** under standard condition (quenched after 24h)

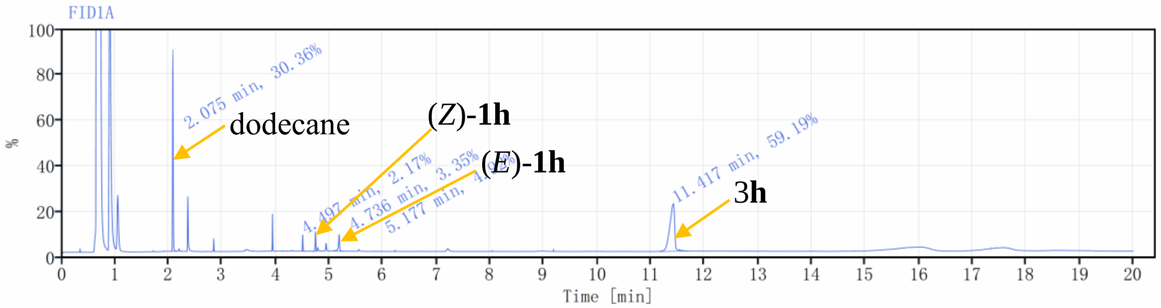

RetTime (min)	Signal	Width (min)	Area	Height	Area%
2.087	FID2B	0.105	23.7	31.8	30.07
4.489	FID2B	0.092	3.0	4.4	3.80
4.726	FID2B	0.107	3.4	3.7	4.25
5.162	FID2B	0.208	5.8	3.9	7.39
11.338	FID2B	0.105	43.0	6.9	54.48

Supplementary Fig. 9. GC analysis for (*Z*)-**1h** under standard condition (quenched after 24h)

IX. Preparation of Substrates

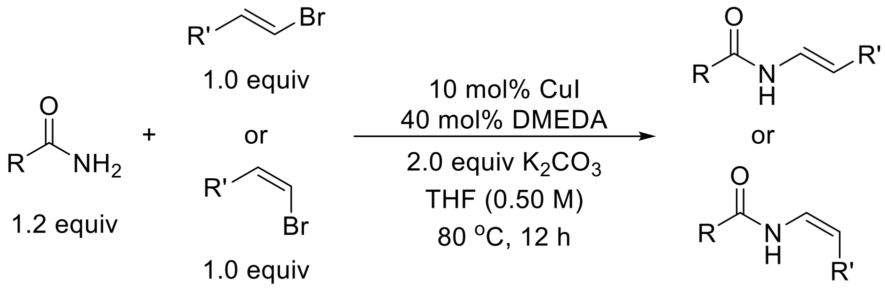

General Procedure (D) for the Olefin Synthesis. The substrates were synthesized according to the previously reported method with modified.[4] A resealable Schlenk tube was charged with CuI (19.2 mg, 0.10 mmol, 10 mol%), K_2CO_3 (280 mg, 2.0 mmol) and amide (1.2 mmol), evacuated and backfilled with argon. *N*,*N'*-Dimethylethylenediamine (488.0 uL, 0.40 mmol, 40 mol%), vinyl bromide (1.00 mmol) and THF (2.0 mL) were added under argon. The Schlenk tube was sealed with a Teflon valve, immerse in a preheated oil bath; the reaction mixture was stirred at the indicated temperature until the complete consumption of starting material was observed as indicated by GC analysis. The reaction vessel was removed from the oil bath and the resulting pale tan suspension was allowed to reach room temperature, then, it was filtered through a plug silica gel

eluting with ethyl acetate. The filtrate was concentrated and the residue was purified by column chromatography on silica gel to provide the desired product.

Characterization of substrates:

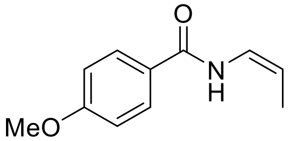

(*Z*)-4-Methoxy-*N*-(prop-1-en-1-yl)benzamide (**1b**):
Yield 37%, white sold;
^{1}H NMR (500 MHz, $CDCl_3$) δ 7.80 (d, *J* = 8.8 Hz, 2H), 7.57 (d, *J* = 10.6 Hz, 1H), 7.00 – 6.91 (m, 3H), 4.97 – 4.88 (m, 1H), 3.88 (s, 3H), 1.72 (dd, *J* = 7.0, 1.8 Hz, 3H);
^{13}C NMR (126 MHz, $CDCl_3$) δ 163.8, 162.5, 128.9, 126.2, 122.4, 113.9, 105.5, 77.3, 77.1, 76.8, 55.5, 10.9;
HRMS (ESI) calcd. for $C_{11}H_{14}NO_2$ $[M+H]^+$ *m/z* 192.1019, found 192.1017;
IR (neat, cm^{-1}) 3675, 2987, 1406, 1066;
m.p. 64.3 – 65.4 ^oC.

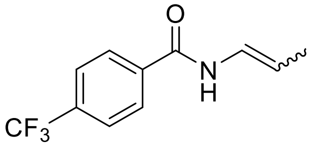

***N*-(Prop-1-en-1-yl)-4-(trifluoromethyl)benzamide** (**1c**):
Yield 82%, white sold;
^{1}H NMR (500 MHz, $CDCl_3$) δ 7.93 (d, *J* = 8.0 Hz, 2H), 7.74 (d, *J* = 7.8 Hz, 2H), 7.70 (d, *J* = 5.8 Hz, 1H), 6.97 – 6.89 (m, 1H), 5.06 – 4.98 (m, 1H), 1.74 (dd, *J* = 7.1, 1.9 Hz, 3H);
^{13}C NMR (126 MHz, $CDCl_3$) δ 163.2, 137.3, 133.6 (q, *J* = 32.9 Hz), 127.5, 125.8 (q, *J* = 3.7 Hz), 122.9 (q, *J* = 274.2 Hz), 121.9, 121.1, 107.4, 107.4, 11.1;
^{19}F NMR (471 MHz, $CDCl_3$) δ –62.9;
HRMS (ESI) calcd. for $C_{11}H_{11}F_3NO$ $[M+H]^+$ *m/z* 230.0787, found 230.0785;
IR (neat, cm^{-1}) 3675, 2987, 1066;
m.p. 100.8 – 101.3 ^oC.

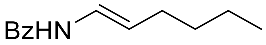

(*E*)-*N*-(Hex-1-en-1-yl)benzamide (**1g**):
Yield 90%, white sold;
^{1}H NMR (500 MHz, $CDCl_3$) δ 7.90 (d, *J* = 12.4 Hz, 1H), 7.85 – 7.79 (m, 2H), 7.52 (t, *J* = 7.4 Hz, 1H), 7.44 (t, *J* = 7.6 Hz, 2H), 6.97 (dd, *J* = 14.2, 10.5 Hz, 1H), 5.38 – 5.30 (m, 1H), 2.15 – 2.05 (m, 2H), 1.46 – 1.29 (m, 4H), 0.92 (t, *J* = 7.1 Hz, 3H);
^{13}C NMR (126 MHz, $CDCl_3$) δ 164.4, 133.9, 131.7, 128.6, 127.0, 122.8, 114.5, 32.0, 29.5, 22.1, 13.9;
HRMS (ESI) calcd. for $C_{13}H_{18}NO$ $[M+H]^+$ *m/z* 204.1383, found 204.1382;
IR (neat, cm^{-1}) 3674, 2987, 1451, 1075;
m.p. 90.2 – 90.7 ^oC.

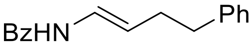

(*E*)-*N*-(4-Phenylbut-1-en-1-yl)benzamide (**1h**):
Yield 84%, white sold;
^{1}H NMR (500 MHz, $CDCl_3$) δ 7.81 (d, *J* = 7.2 Hz, 2H), 7.67 (d, *J* = 10.7 Hz, 1H), 7.54 (t, *J* = 7.4 Hz, 1H), 7.47 (t, *J* = 7.5 Hz, 2H), 7.35 – 7.29 (m, 2H), 7.26 – 7.19 (m, 3H), 7.04 (dd, *J* = 14.2, 10.5 Hz, 1H), 5.34 (dt, *J* = 14.2, 7.1 Hz, 1H), 2.75 (t, *J* = 7.7 Hz, 2H), 2.45 (q, *J* = 7.0 Hz, 2H);
^{13}C NMR (126 MHz, $CDCl_3$) δ 164.3, 141.5, 133.8, 131.9, 128.7, 128.5, 128.4, 127.0, 125.9, 123.4, 113.1, 36.4, 31.6;
HRMS (ESI) calcd. for $C_{17}H_{18}NO$ $[M+H]^+$ *m/z* 252.1383, found 252.1382;
IR (neat, cm^{-1}) 3674, 2987, 1451, 1066;
m.p. 118.9 – 119.1 °C.

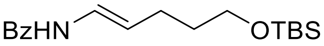

(*E*)-*N*-(5-((*tert*-Butyldimethylsilyl)oxy)pent-1-en-1-yl)benzamide (**1i**):
Yield 55%, white sold;
^{1}H NMR (500 MHz, $CDCl_3$) δ 7.81 (d, *J* = 7.3 Hz, 2H), 7.52 (t, *J* = 7.4 Hz, 1H), 7.49 – 7.41 (m, 2H), 7.04 – 6.94 (m, 1H), 5.38 – 5.30 (m, 1H), 3.65 (t, *J* = 6.3 Hz, 2H), 2.21 – 2.11 (m, 2H), 1.69 – 1.60 (m, 2H), 0.92 (s, 9H), 0.07 (s, 6H);
^{13}C NMR (126 MHz, $CDCl_3$) δ 164.3, 133.8, 131.8, 128.7, 127.0, 123.0, 113.8, 62.5, 32.9, 26.2, 25.9, –5.3;
HRMS (ESI) calcd. for $C_{18}H_{30}NO_2Si$ $[M+H]^+$ *m/z* 320.2040, found 320.2041;
IR (neat, cm^{-1}) 3688, 2987, 1449, 1075;
m.p. 88.2– 88.9 °C.

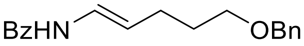

(*E*)-*N*-(5-(Benzyloxy)pent-1-en-1-yl)benzamide (**1j**):
Yield 63%, white sold;
^{1}H NMR (500 MHz, $CDCl_3$) δ 7.87 – 7.76 (m, 3H), 7.53 (t, *J* = 7.4 Hz, 1H), 7.45 (t, *J* = 7.5 Hz, 2H), 7.37 (d, *J* = 4.4 Hz, 4H), 7.34 – 7.26 (m, 1H), 6.99 (dd, *J* = 14.2, 10.5 Hz, 1H), 5.33 (dt, *J* = 14.3, 6.9 Hz, 1H), 4.53 (s, 2H), 3.52 (t, *J* = 6.4 Hz, 2H), 2.20 (q, *J* = 7.3 Hz, 2H), 1.77 – 1.71 (m, 2H);
^{13}C NMR (126 MHz, $CDCl_3$) δ 164.3, 138.5, 133.8, 131.8, 128.7, 128.4, 127.7, 127.6, 127.0, 123.21, 113.6, 72.9, 69.6, 29.9, 26.6;
HRMS (ESI) calcd. for $C_{19}H_{22}NO_2$ $[M+H]^+$ *m/z* 296.1645, found 296.1644;
IR (neat, cm^{-1}) 3674, 2987, 1452, 1066;
m.p. 55.7 – 55.9 °C.

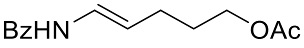

(*E*)-5-Benzamidopent-4-en-1-yl acetate (**1k**):
Yield 67%, white sold;
^{1}H NMR (500 MHz, $CDCl_3$) δ 8.20 (d, 1H), 7.85 (d, *J* = 7.2 Hz, 1H), 7.60 – 7.51 (m, 2H), 7.51 – 7.44 (m, 1H), 6.82 (dd, *J* = 60.9, 9.3 Hz, 1H), 5.08 – 4.81 (m, 1H), 4.20 – 4.12 (m, 1H), 4.10 – 4.00 (m, 1H), 2.28 – 2.13 (m, 1H), 2.03 (d, *J* = 23.0 Hz, 3H), 1.92 – 1.85 (m, 1H), 1.84 – 1.61 (m, 2H);

^{13}C NMR (126 MHz, $CDCl_3$) δ 171.3, 164.5, 133.3, 131.8, 131.8, 128.7, 128.6, 127.1, 127.1, 123.7, 112.5, 112.5, 63.7, 28.8, 26.3, 21.0;
HRMS (ESI) calcd. for $C_{14}H_{18}NO_3$ $[M+H]^+$ *m/z* 243.1280, found 243.1281;
IR (neat, cm^{-1}) 3674, 2987, 1406, 1075;
m.p. 88.7 – 89.6 °C.

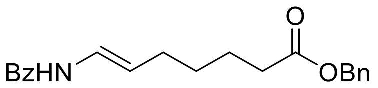

Benzyl (*E*)-7-benzamidohept-6-enoate (**1l**):
Yield 48%, white sold;
^{1}H NMR (500 MHz, $CDCl_3$) δ 7.81 (d, *J* = 7.7 Hz, 2H), 7.69 (d, *J* = 10.3 Hz, 1H), 7.54 (t, *J* = 7.4 Hz, 1H), 7.47 (t, *J* = 7.5 Hz, 2H), 7.42 – 7.36 (m, 4H), 7.31 (s, 1H), 6.98 (dd, *J* = 13.6, 10.0 Hz, 1H), 5.32 – 5.25 (m, 1H), 5.14 (s, 2H), 2.39 (t, *J* = 7.5 Hz, 2H), 2.16 – 2.07 (m, 2H), 1.74 – 1.68 (m, 2H), 1.50 – 1.40 (m, 2H);
^{13}C NMR (126 MHz, $CDCl_3$) δ 173.5, 164.3, 136.1, 133.8, 131.8, 128.7, 128.6, 128.2, 128.2, 127.0, 123.2, 113.5, 66.2, 34.1, 29.4, 29.3, 24.3;
HRMS (ESI) calcd. for $C_{21}H_{24}NO_3$ $[M+H]^+$ *m/z* 338.1751, found 338.1750;
IR (neat, cm^{-1}) 3674, 2987, 1717, 1635, 1075;
m.p. 46.3 – 46.9 °C.

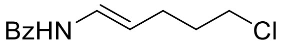

(*E*)-*N*-(5-Chloropent-1-en-1-yl)benzamide (**1m**):
Yield 67%, white sold;
^{1}H NMR (500 MHz, $CDCl_3$) δ 7.82 (d, *J* = 8.3 Hz, 2H), 7.54 (t, *J* = 7.4 Hz, 1H), 7.46 (t, *J* = 7.7 Hz, 2H), 7.03 (dd, *J* = 14.2, 10.5 Hz, 1H), 5.31 (dt, *J* = 14.3, 7.2 Hz, 1H), 3.58 (t, *J* = 6.6 Hz, 2H), 2.31 – 2.23 (m, 2H), 1.94 – 1.85 (m, 2H);
^{13}C NMR (126 MHz, $CDCl_3$) δ 164.4, 133.7, 131.9, 128.7, 127.0, 127.0, 123.9, 112.0, 111.9, 44.2, 32.6, 27.0;
HRMS (ESI) calcd. for $C_{12}H_{14}ClNO$ $[M+H]^+$ *m/z* 224.0837, found 224.0835;
IR (neat, cm^{-1}) 3675, 2987, 1473, 1075;
m.p. 100.4 – 100.8 °C.

X. Spectroscopic Data (NMR Spectrum)

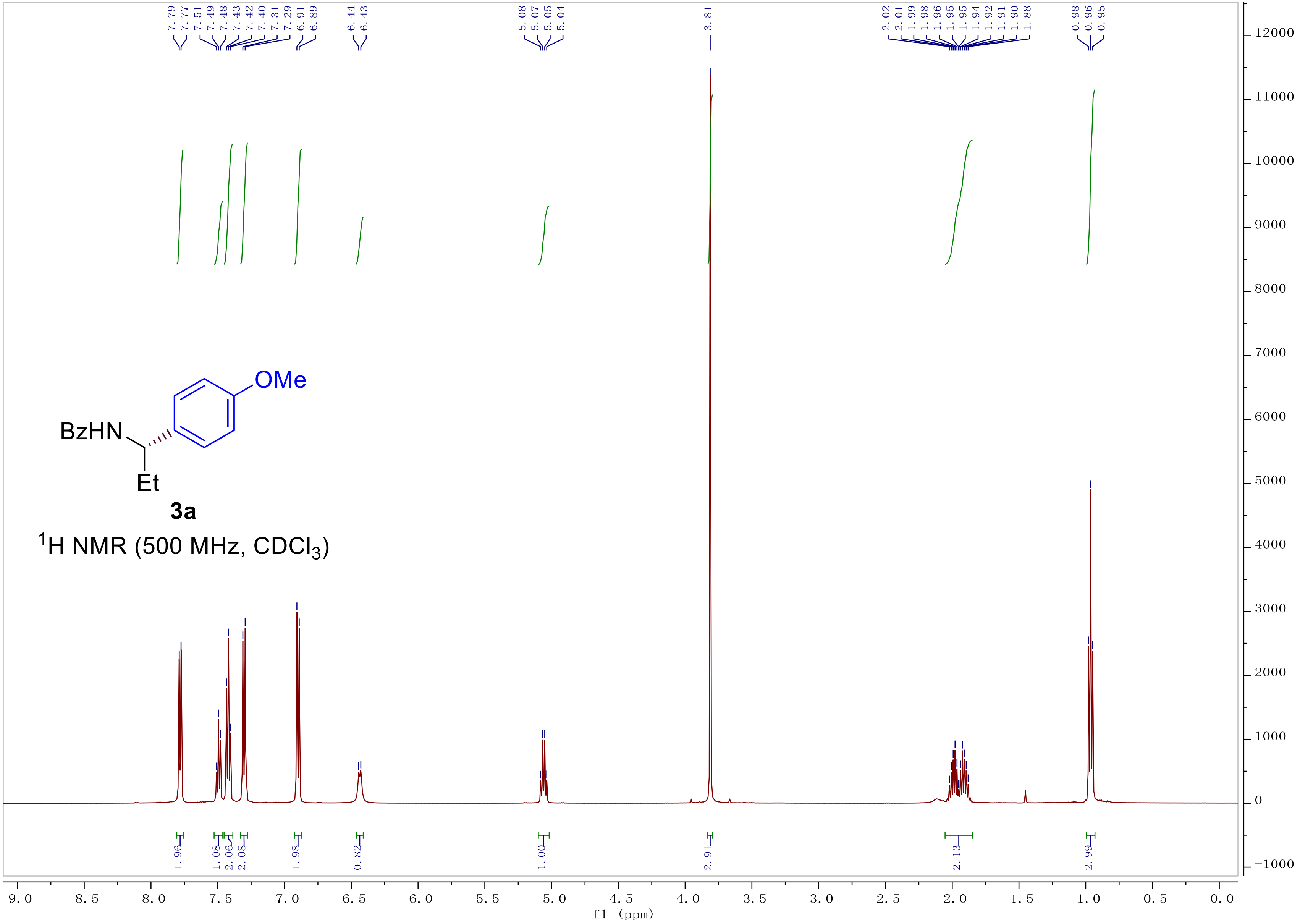

7.79
7.77
7.51
7.49
7.48
7.43
7.42
7.40
7.31
7.29
6.91
6.89
6.44
6.43
5.08
5.07
5.05
5.04
3.81
2.02
2.01
1.99
1.98
1.96
1.95
1.95
1.94
1.92
1.91
1.90
1.88
0.98
0.96
0.95
OMe
BzHN
Et
3a
^{1}H NMR (500 MHz, CDCl$_3$)
1.96
1.08
2.06
2.08
1.98
0.82
1.00
2.91
2.13
2.99
f1 (ppm)

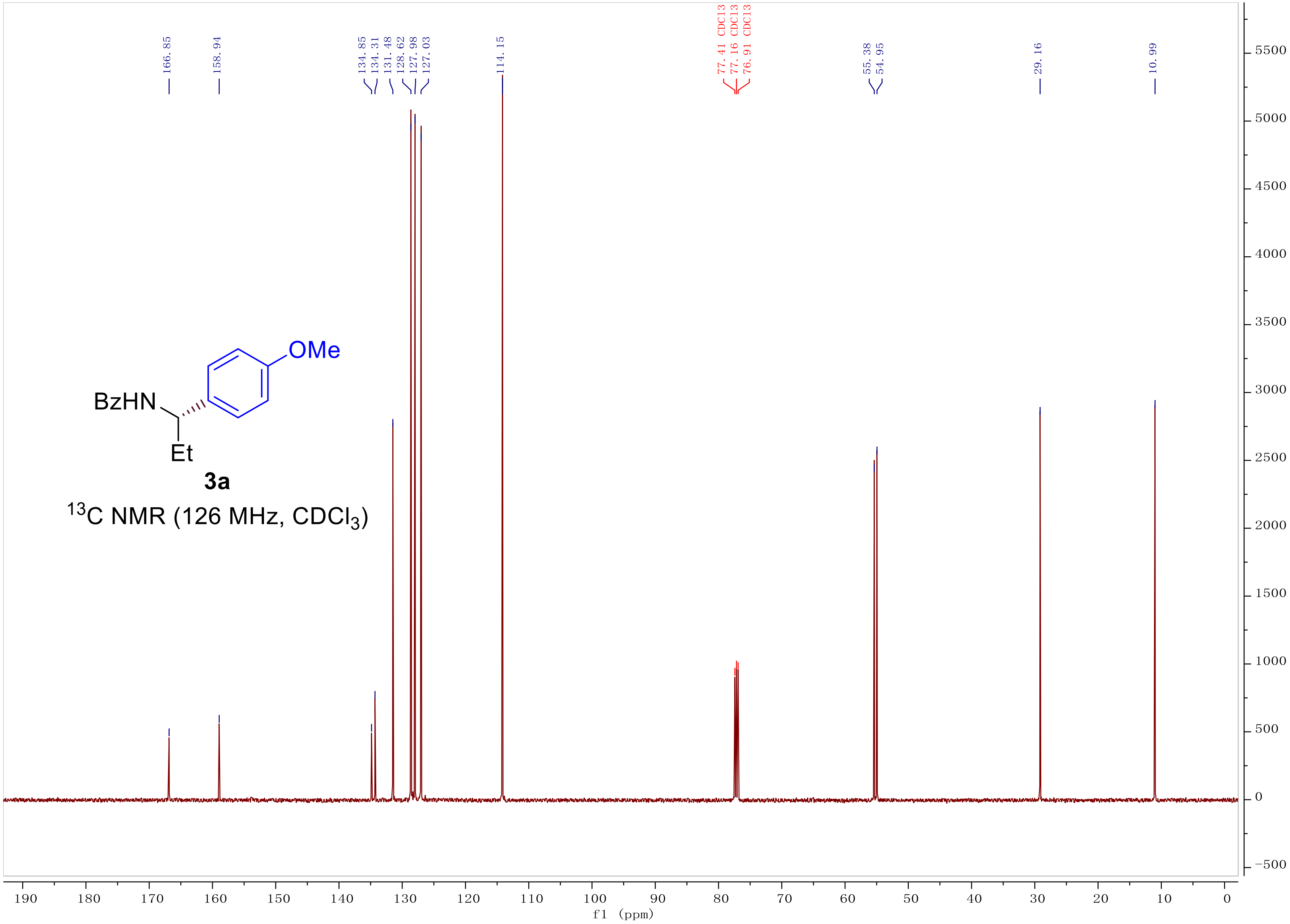
166.85
158.94
134.85
134.31
131.48
128.62
127.98
127.03
114.15
77.41 CDCl3
77.16 CDCl3
76.91 CDCl3
55.38
54.95
29.16
10.99
OMe
BzHN
Et
3a
13C NMR (126 MHz, CDCl3)
f1 (ppm)

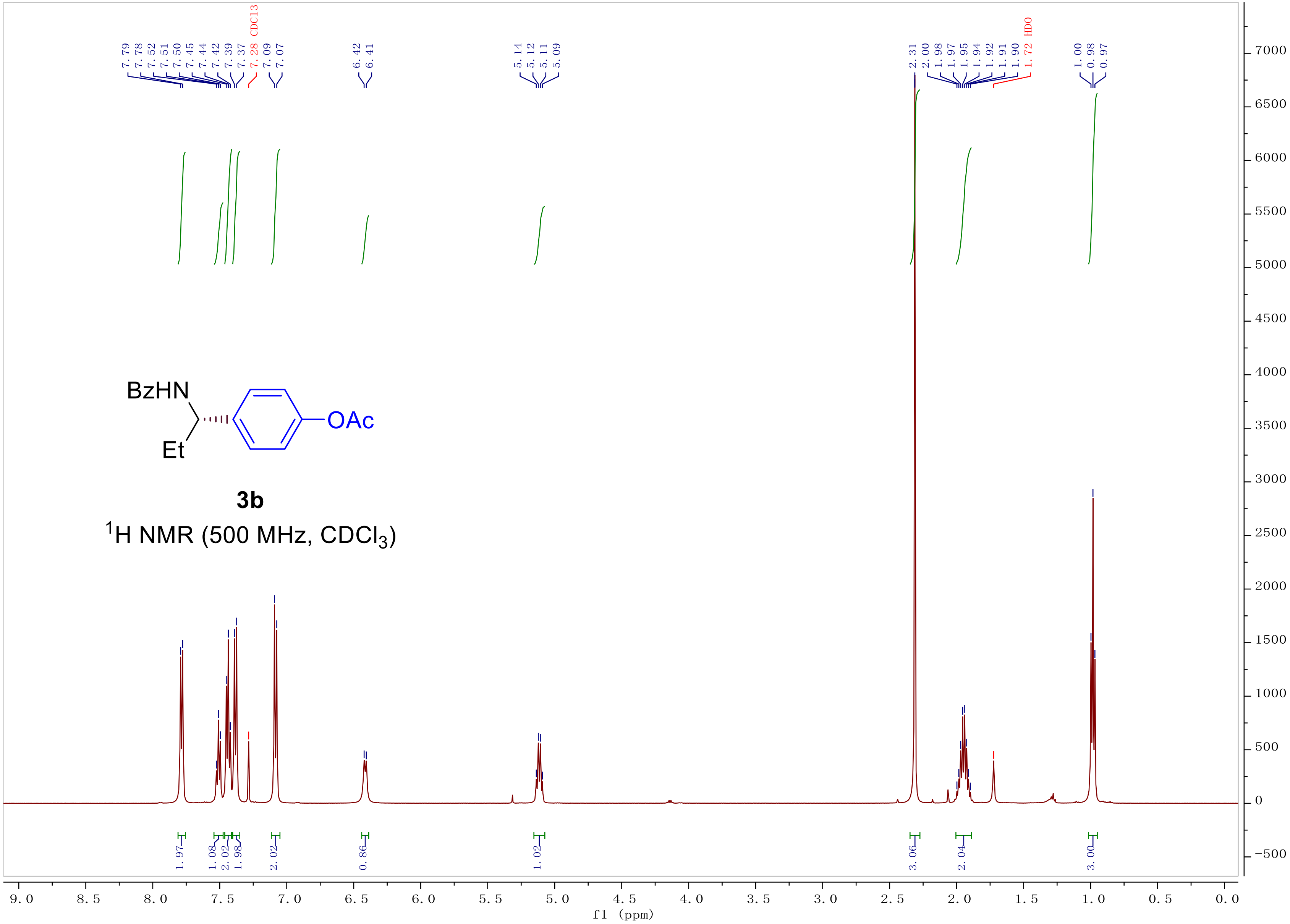

3b

^{1}H NMR (500 MHz, $CDCl_3$)

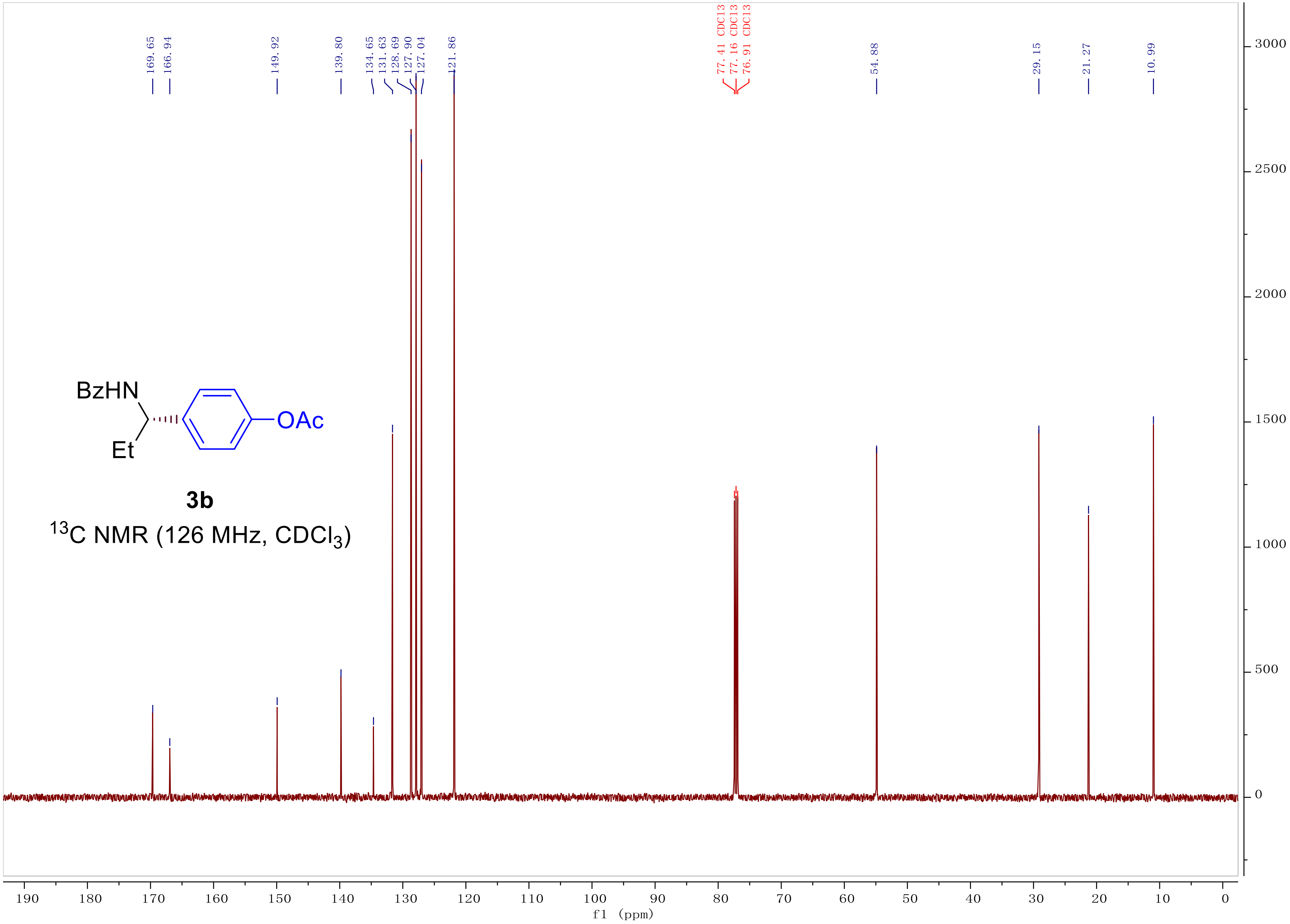
169.65
166.94
149.92
139.80
134.65
131.63
128.69
127.90
127.04
121.86
77.41 CDCl3
77.16 CDCl3
76.91 CDCl3
54.88
29.15
21.27
10.99
BzHN
Et
OAc
3b
^{13}C NMR (126 MHz, $CDCl_3$)
3000
2500
2000
1500
1000
500
0
190 180 170 160 150 140 130 120 110 100 90 80 70 60 50 40 30 20 10 0
f1 (ppm)

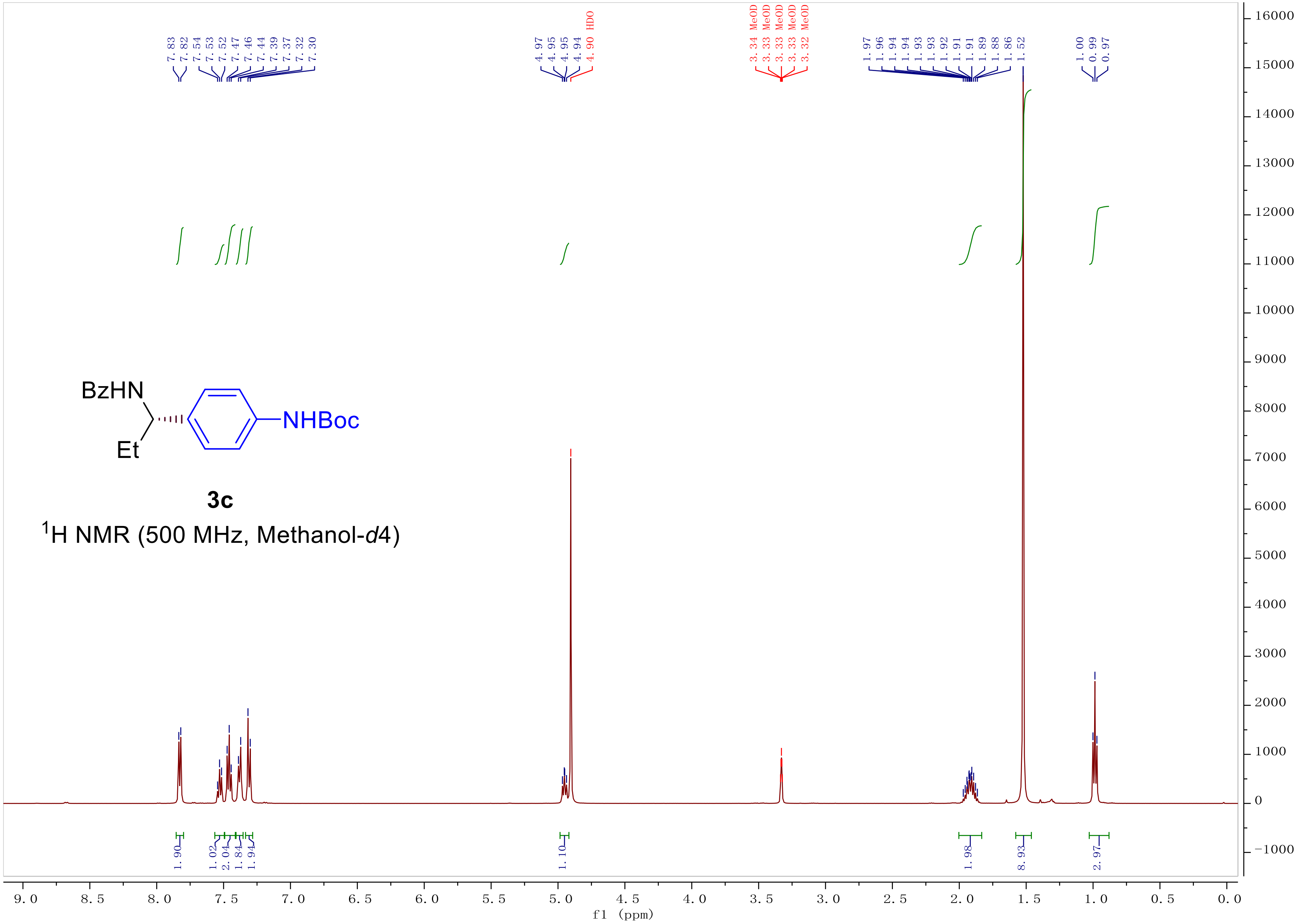

BzHN
Et
NHBoc
3c
^{1}H NMR (500 MHz, Methanol-d4)
f1 (ppm)

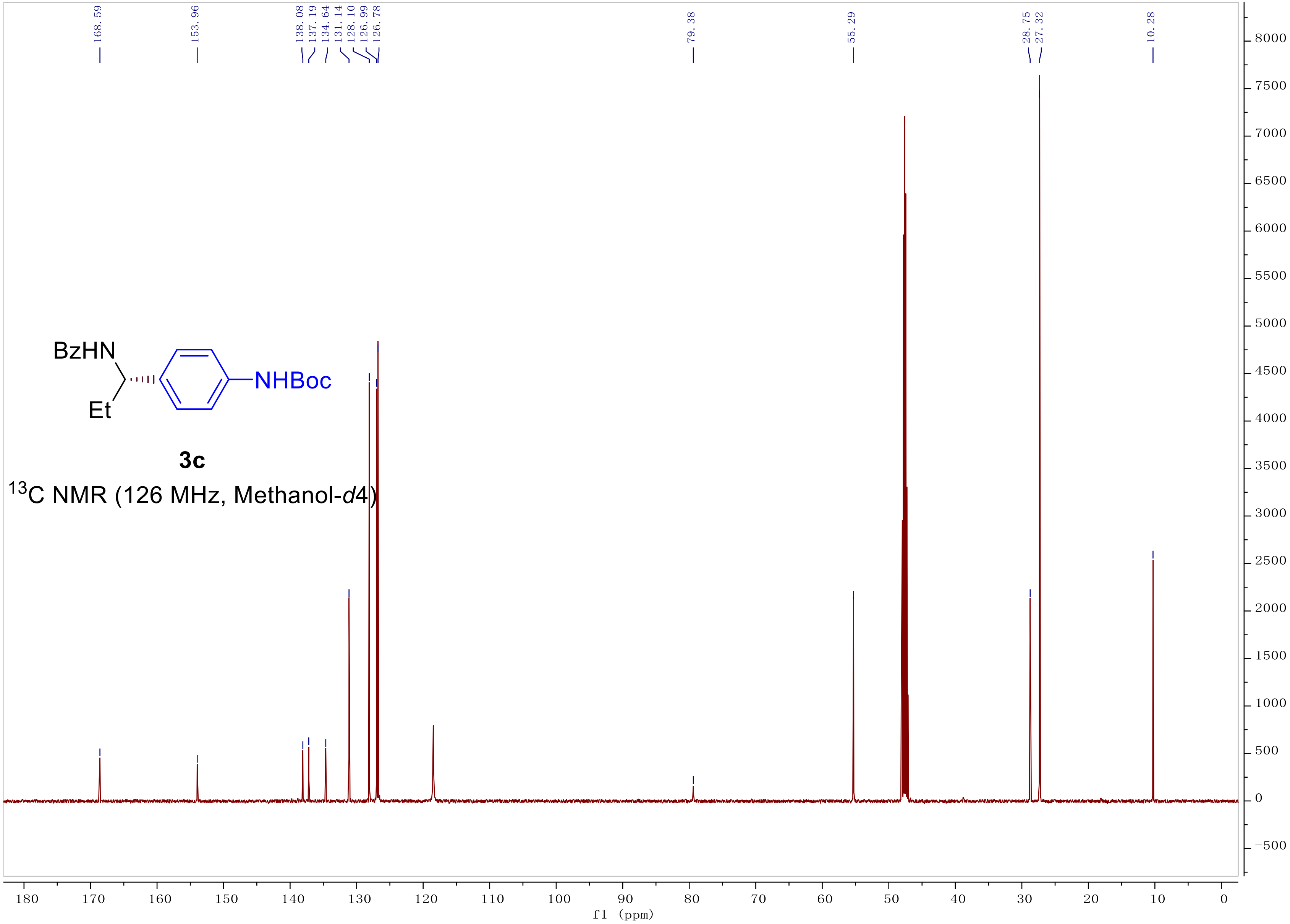
168.59
153.96
138.08
137.19
134.64
131.14
128.10
126.99
126.78
79.38
55.29
28.75
27.32
10.28
BzHN
Et
NHBoc
3c
^{13}C NMR (126 MHz, Methanol-d4)
f1 (ppm)

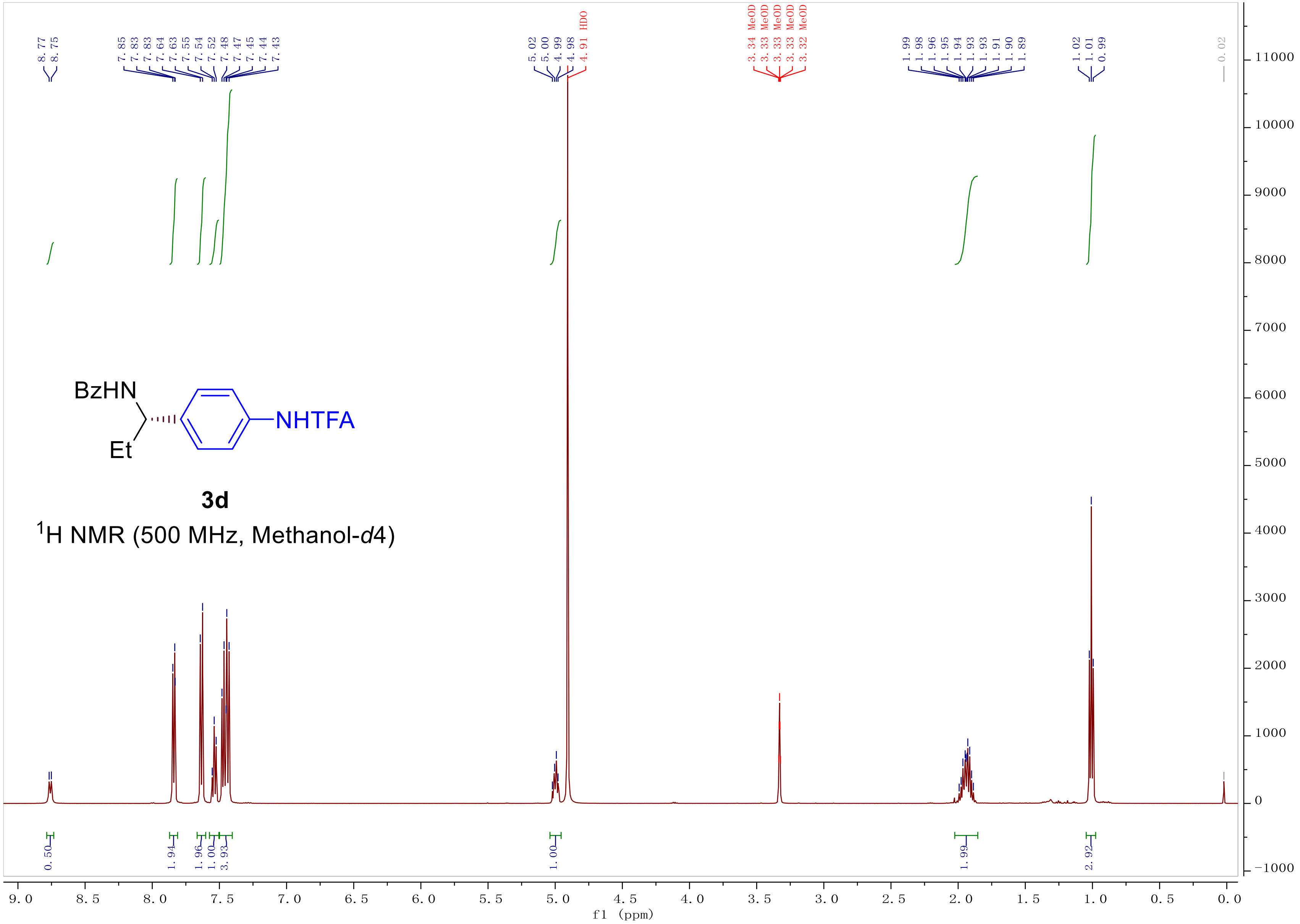

BzHN
Et
NHTFA
3d
¹H NMR (500 MHz, Methanol-d4)
8.77
8.75
7.85
7.83
7.83
7.64
7.63
7.55
7.54
7.52
7.48
7.47
7.45
7.44
7.43
5.02
5.00
4.99
4.98
4.91 HDO
3.34 MeOD
3.33 MeOD
3.33 MeOD
3.33 MeOD
3.32 MeOD
1.99
1.98
1.96
1.95
1.94
1.93
1.93
1.91
1.90
1.89
1.02
1.01
0.99
0.02
0.50
1.94
1.96
1.00
3.93
1.00
1.99
2.92
f1 (ppm)

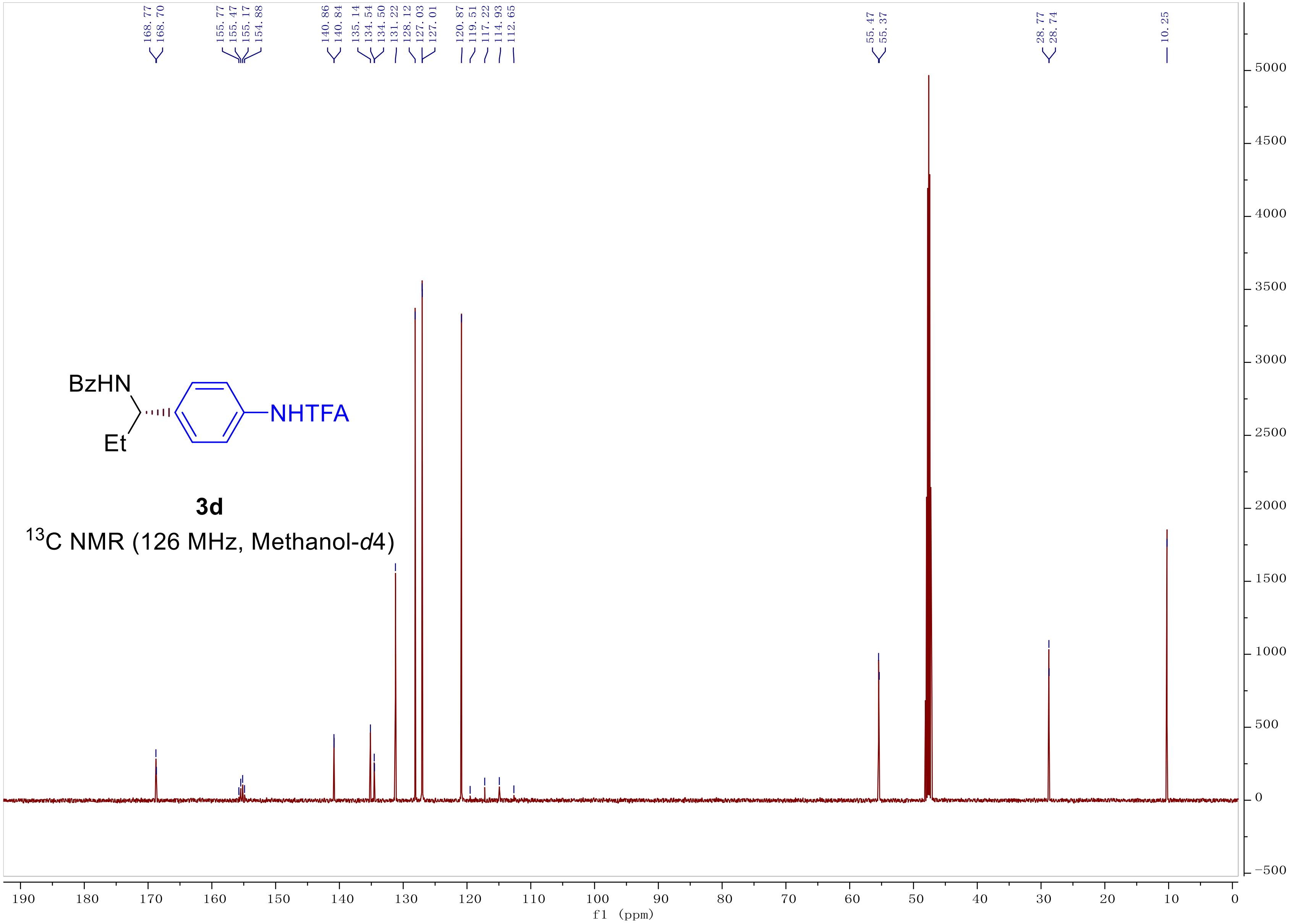

3d

^{13}C NMR (126 MHz, Methanol-*d*4)

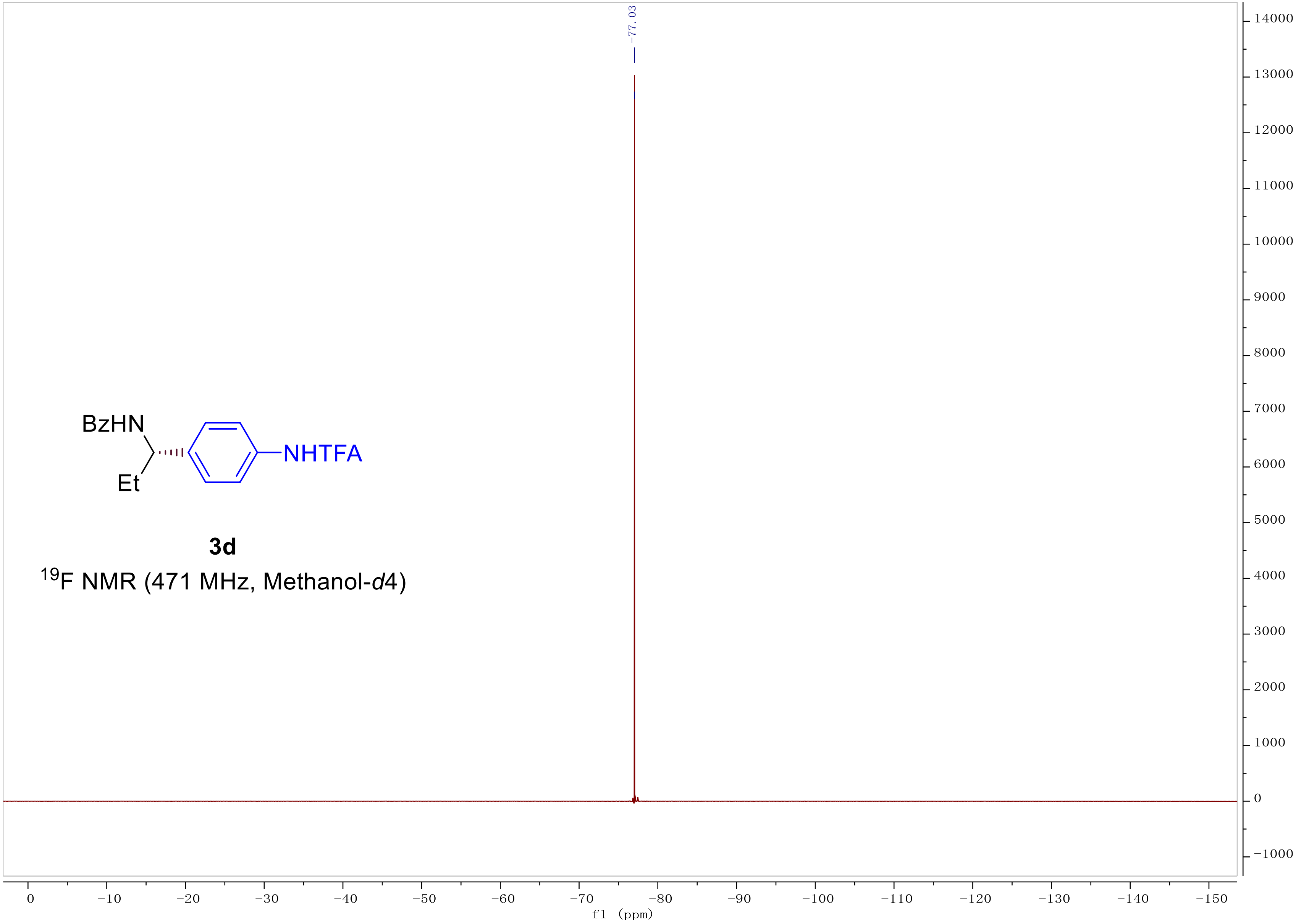

3d

^{19}F NMR (471 MHz, Methanol-*d*4)

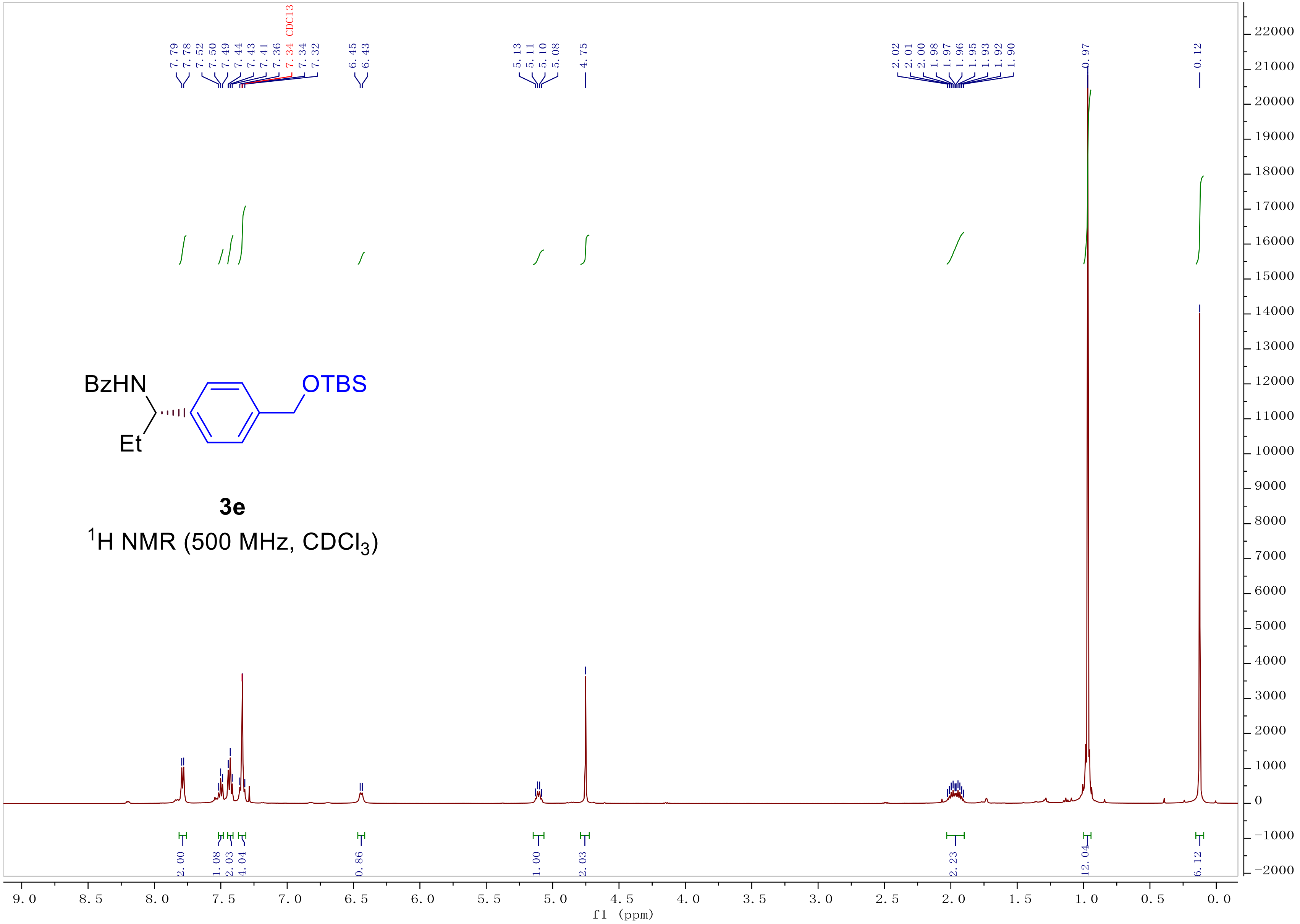
BzHN
Et
OTBS
3e
^{1}H NMR (500 MHz, CDCl$_3$)
f1 (ppm)

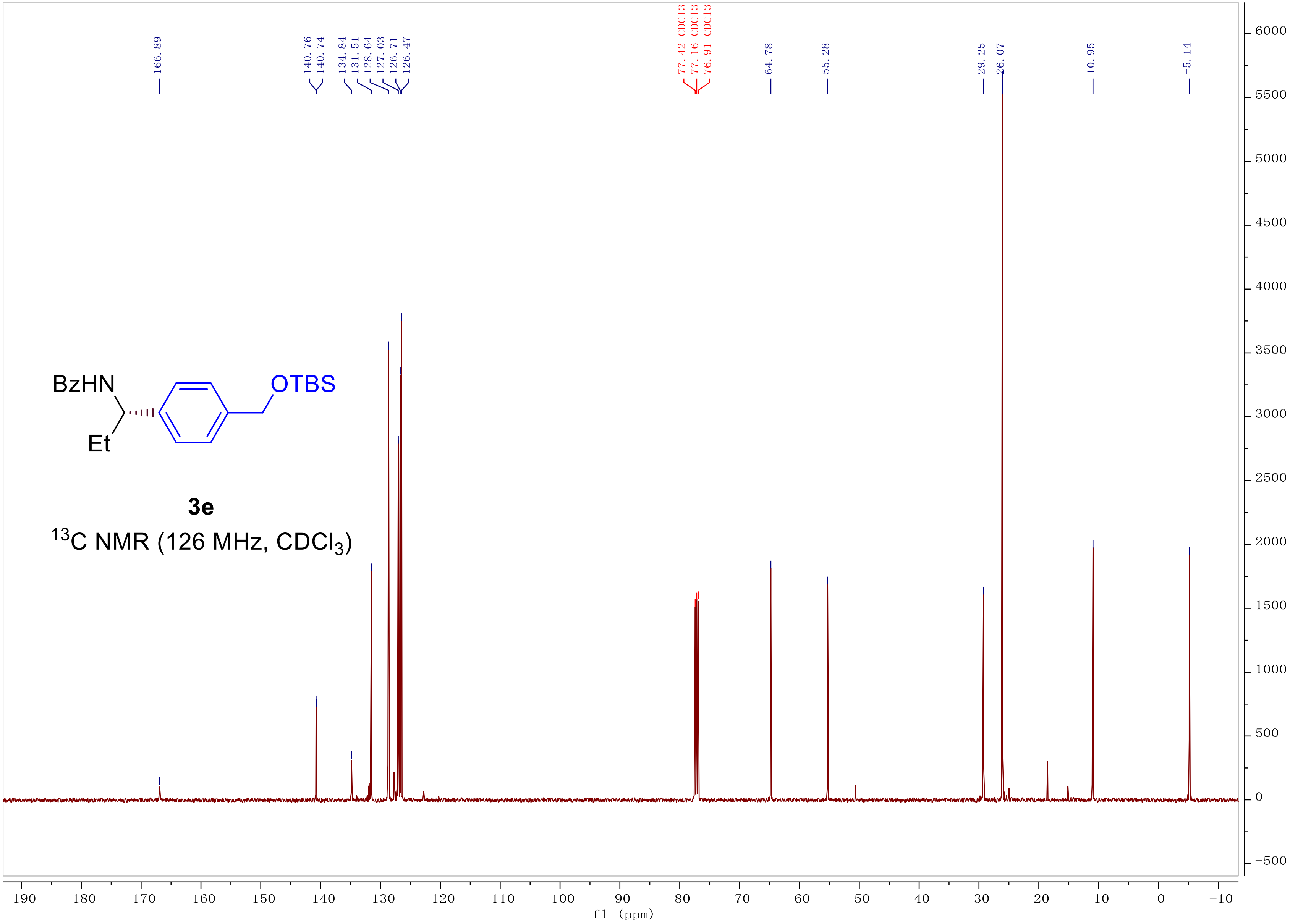
166.89
140.76
140.74
134.84
131.51
128.64
127.03
126.71
126.47
77.42 CDCl3
77.16 CDCl3
76.91 CDCl3
64.78
55.28
29.25
26.07
10.95
-5.14
BzHN
OTBS
Et
3e
^{13}C NMR (126 MHz, $CDCl_3$)
f1 (ppm)

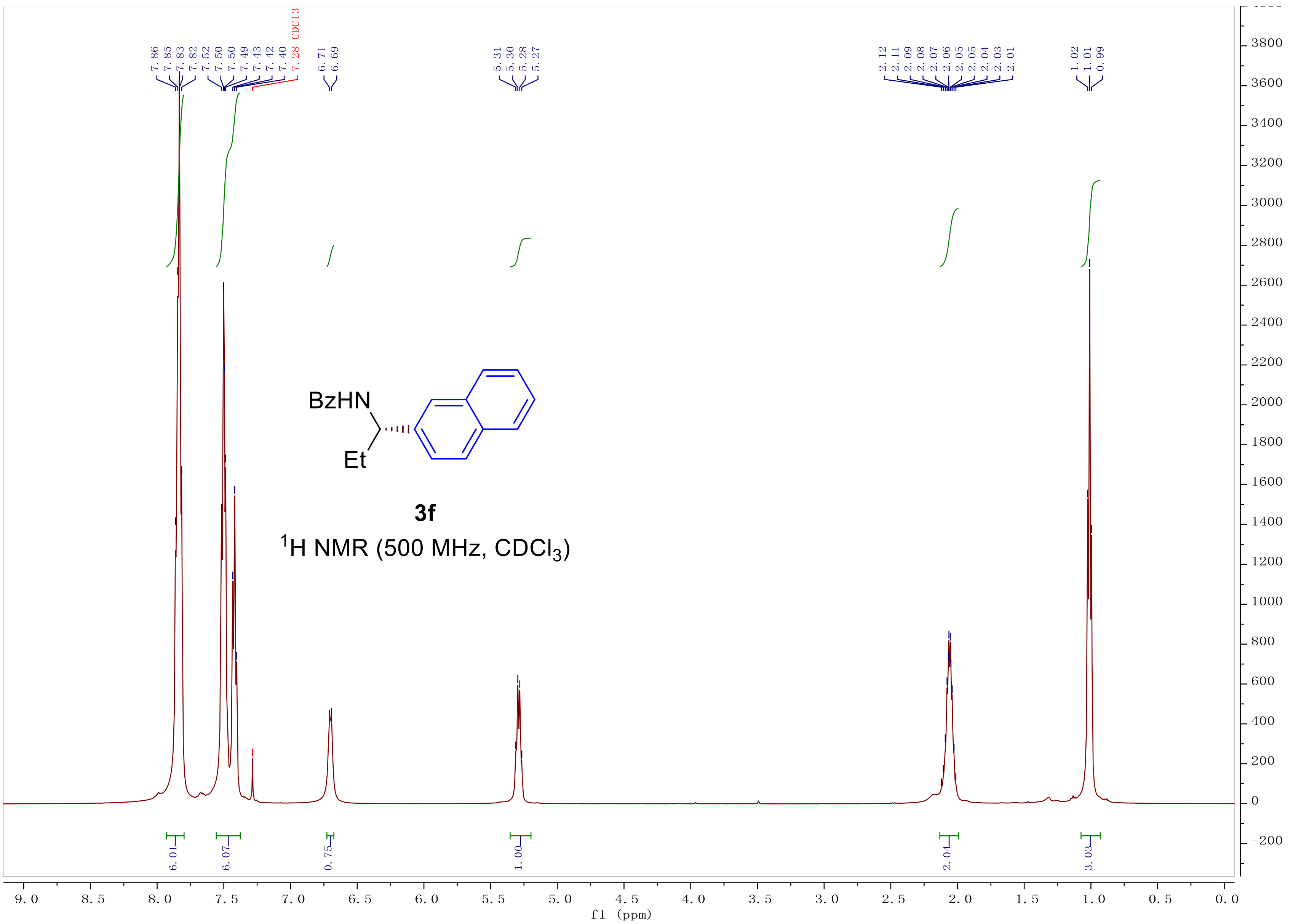
7.86 7.85 7.83 7.82 7.52 7.50 7.50 7.49 7.43 7.42 7.40 7.28 CDCl3 6.71 6.69
5.31 5.30 5.28 5.27
2.12 2.11 2.09 2.08 2.07 2.06 2.05 2.04 2.03 2.01
1.02 1.01 0.99
BzHN
Et
3f
1H NMR (500 MHz, CDCl3)
6.01 6.07 0.75 1.00 2.04 3.03
9.0 8.5 8.0 7.5 7.0 6.5 6.0 5.5 5.0 4.5 4.0 3.5 3.0 2.5 2.0 1.5 1.0 0.5 0.0
f1 (ppm)
3800 3600 3400 3200 3000 2800 2600 2400 2200 2000 1800 1600 1400 1200 1000 800 600 400 200 0 -200

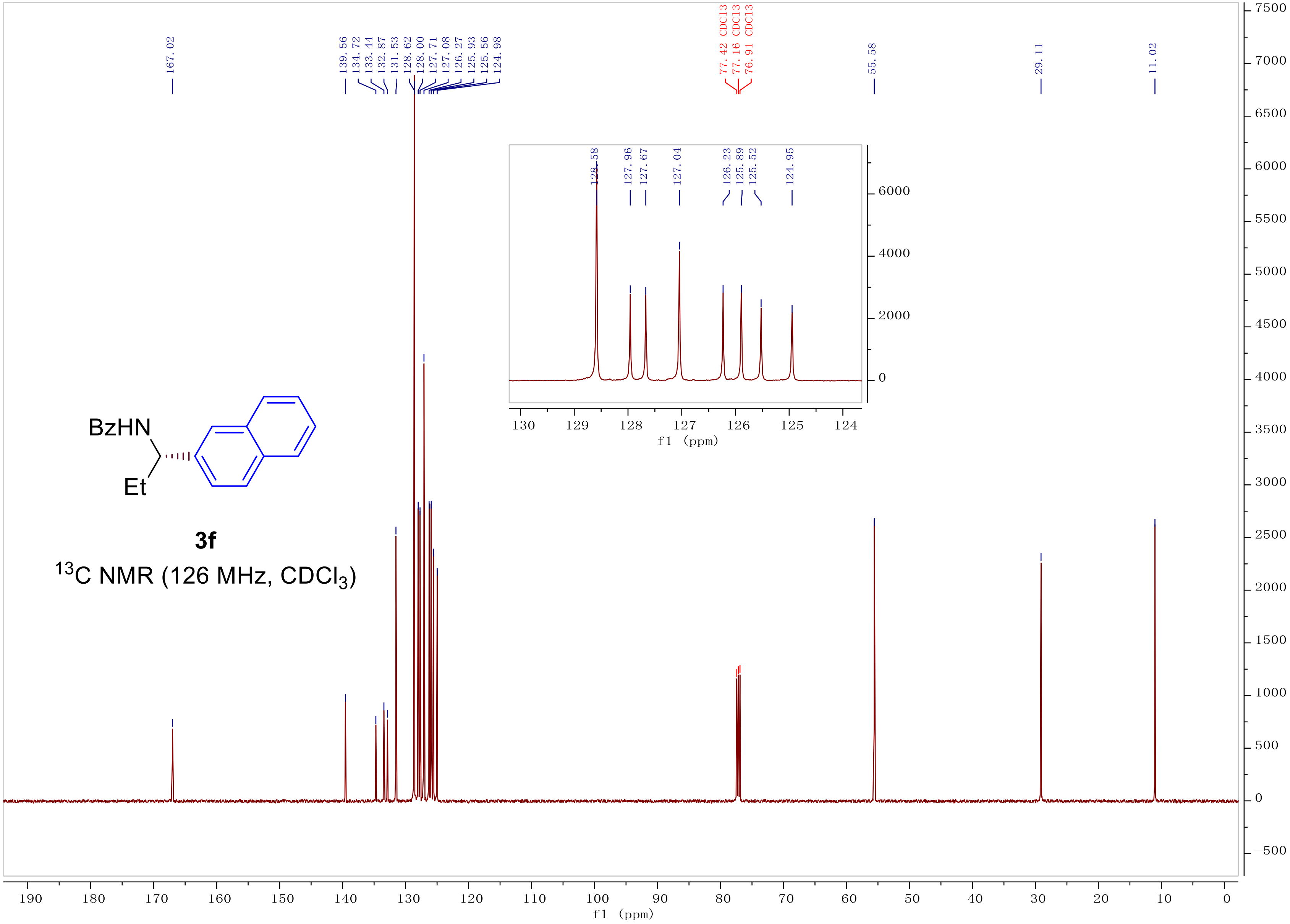

167.02
139.56
134.72
133.44
132.87
131.53
128.62
128.00
127.71
127.08
126.27
125.93
125.56
124.98
77.42 CDCl3
77.16 CDCl3
76.91 CDCl3
55.58
29.11
11.02
128.58
127.96
127.67
127.04
126.23
125.89
125.52
124.95
f1 (ppm)
BzHN
Et
3f
^{13}C NMR (126 MHz, $CDCl_3$)

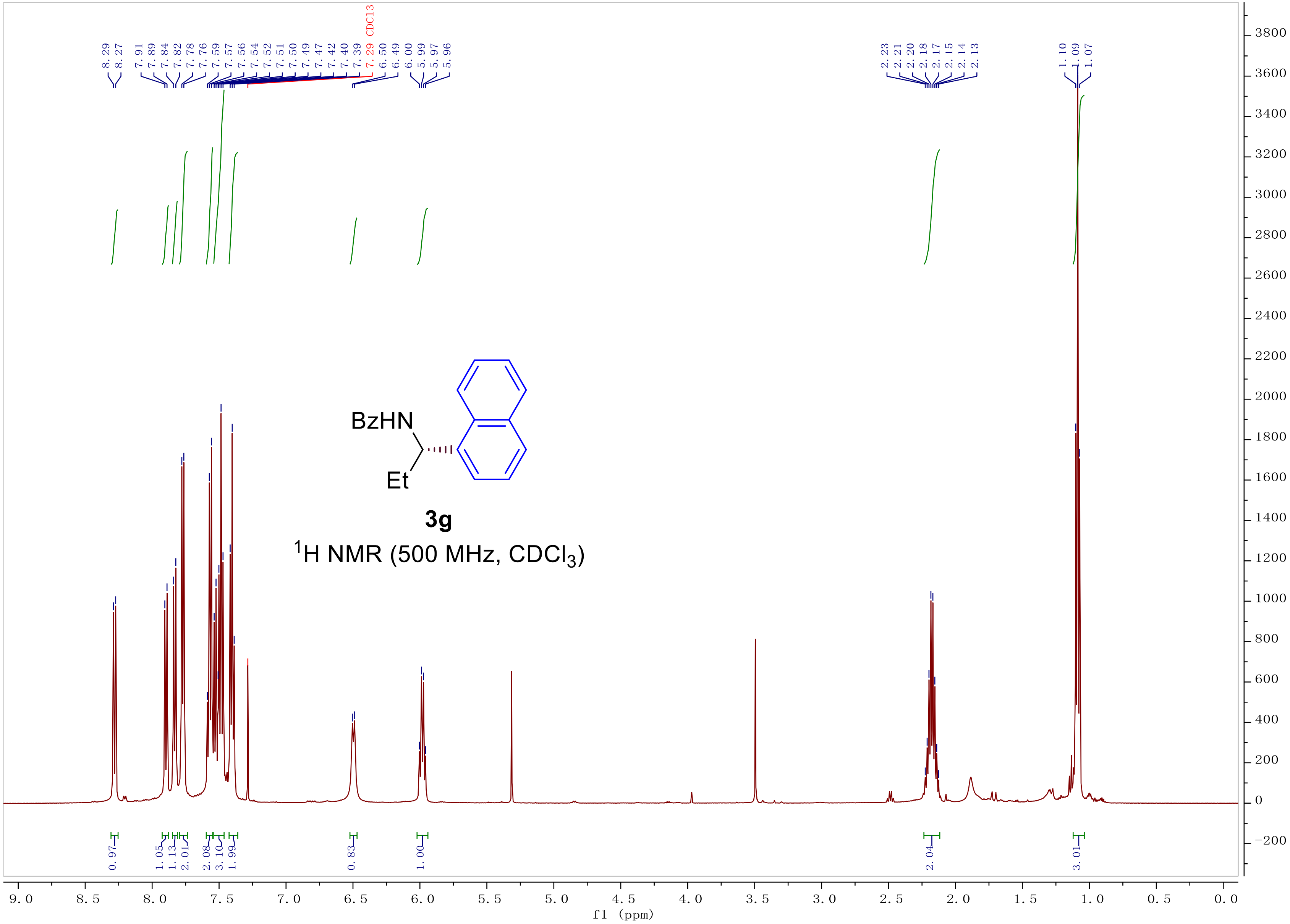

BzHN
Et
3g
^{1}H NMR (500 MHz, CDCl$_3$)
f1 (ppm)

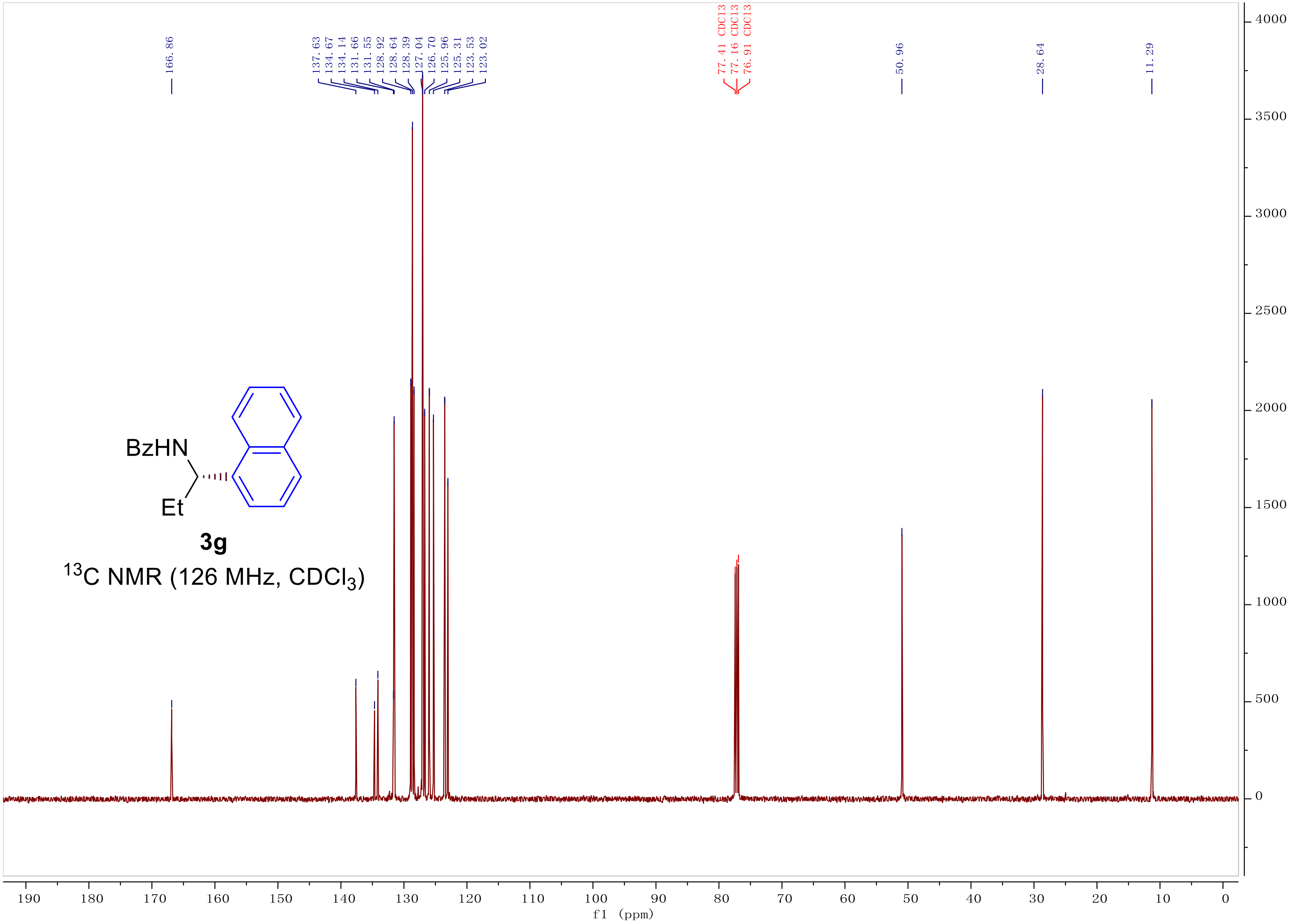
166.86
137.63
134.67
134.14
131.66
131.55
128.92
128.64
128.39
127.04
126.70
125.96
125.31
123.53
123.02
77.41 CDCl3
77.16 CDCl3
76.91 CDCl3
50.96
28.64
11.29
BzHN
Et
3g
13C NMR (126 MHz, CDCl3)
f1 (ppm)

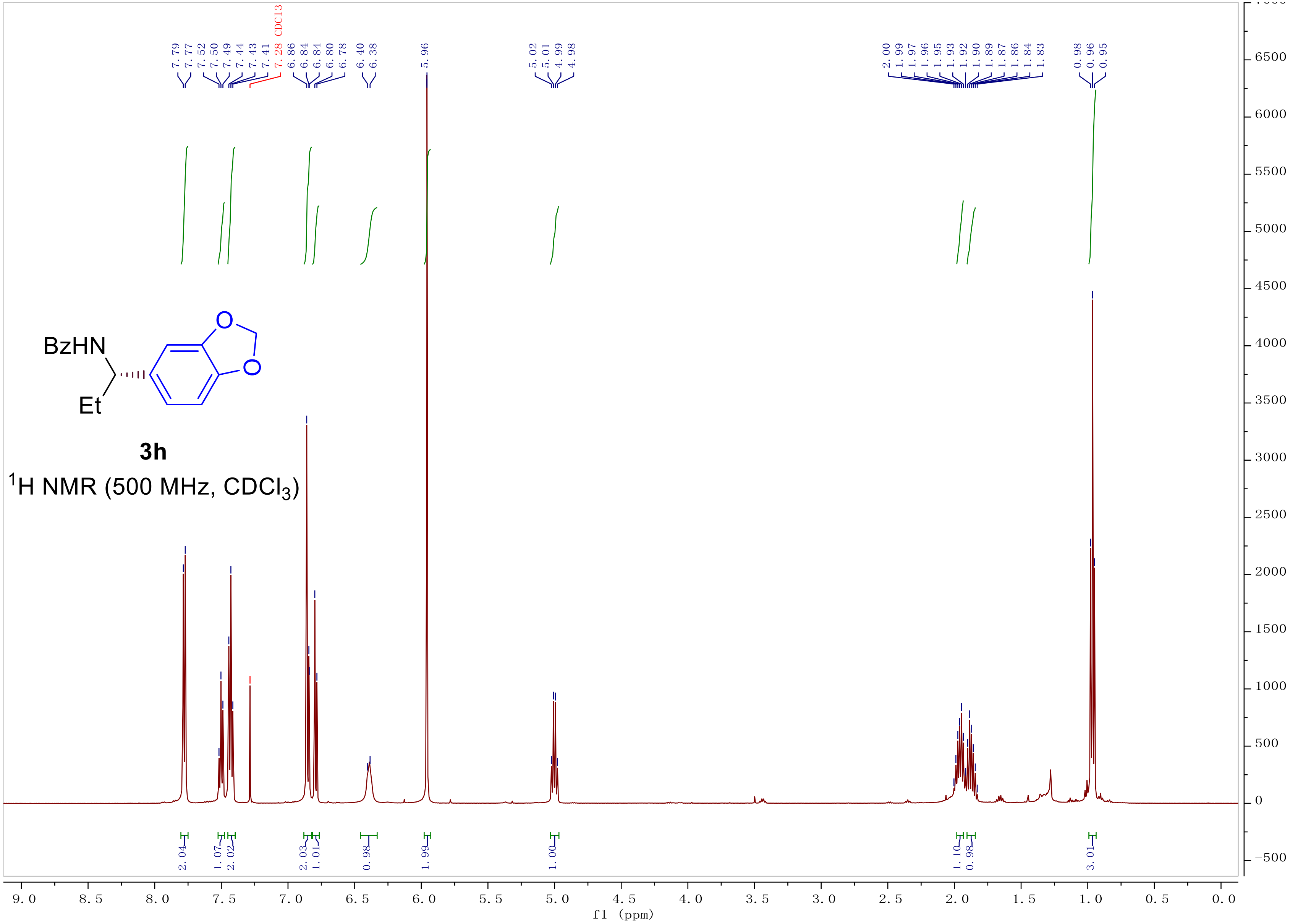

BzHN
Et
3h
1H NMR (500 MHz, CDCl3)
f1 (ppm)

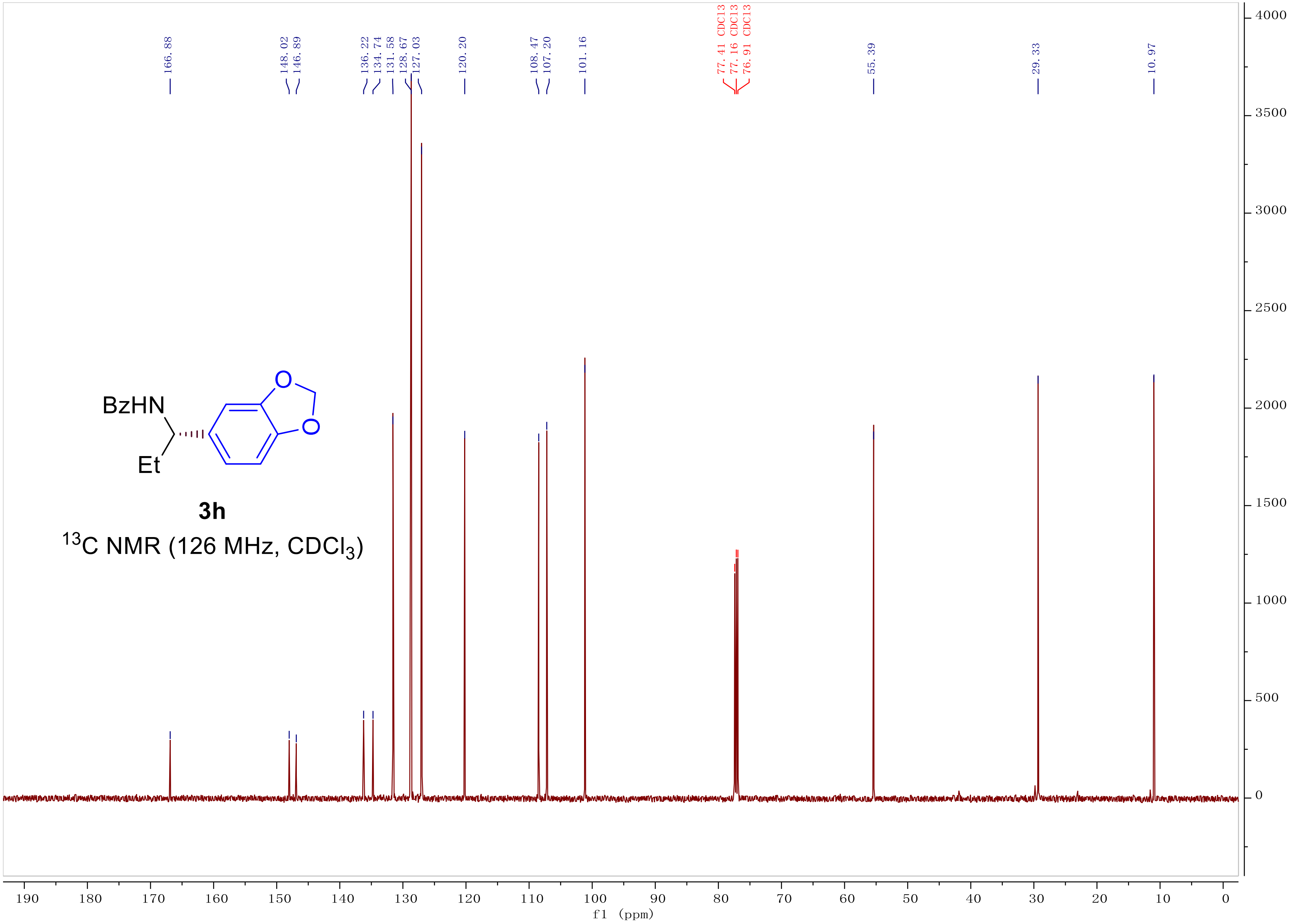

166.88
148.02
146.89
136.22
134.74
131.58
128.67
127.03
120.20
108.47
107.20
101.16
77.41 CDCl3
77.16 CDCl3
76.91 CDCl3
55.39
29.33
10.97
BzHN
Et
3h
^{13}C NMR (126 MHz, $CDCl_3$)
f1 (ppm)

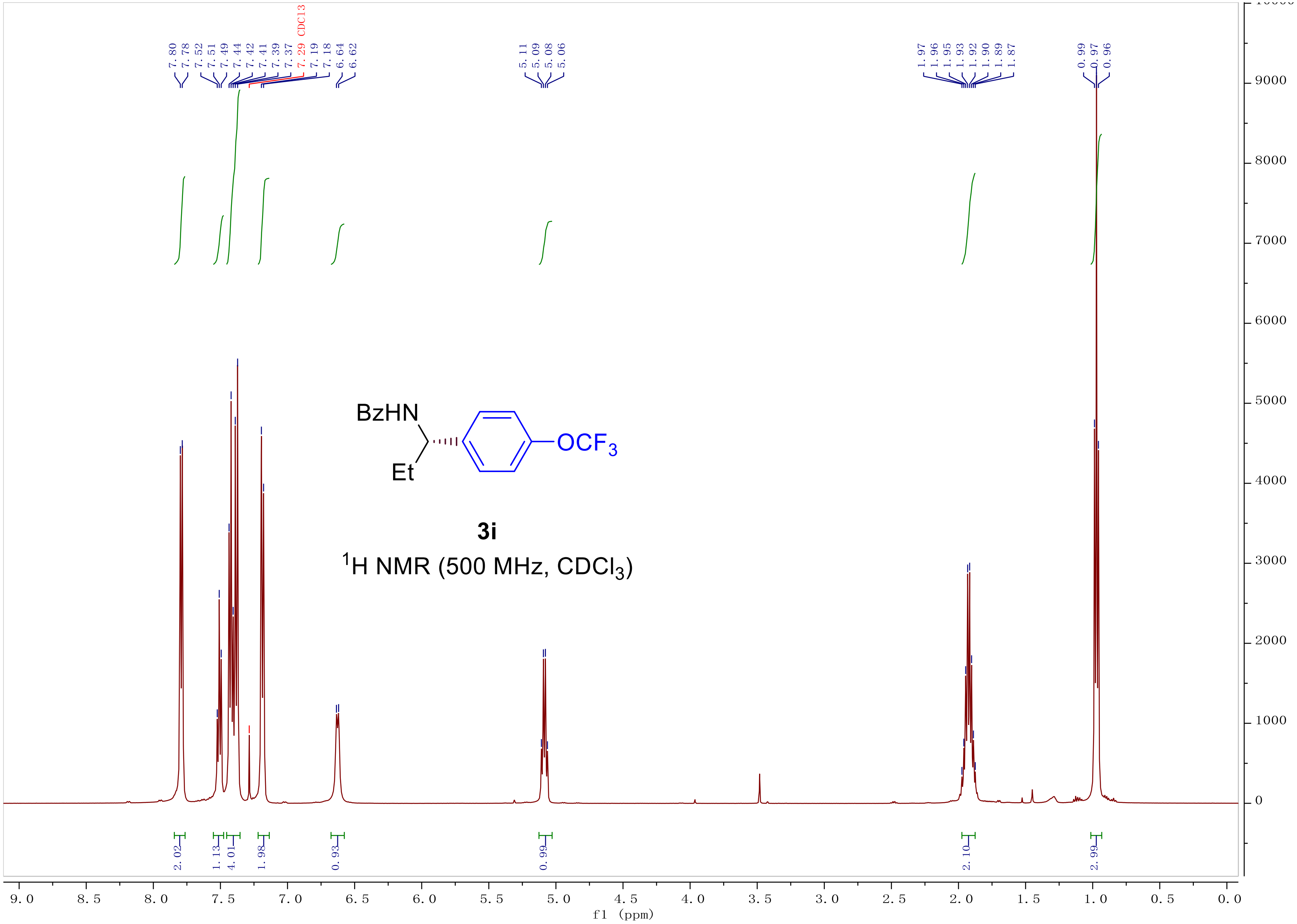

3i

^{1}H NMR (500 MHz, $CDCl_3$)

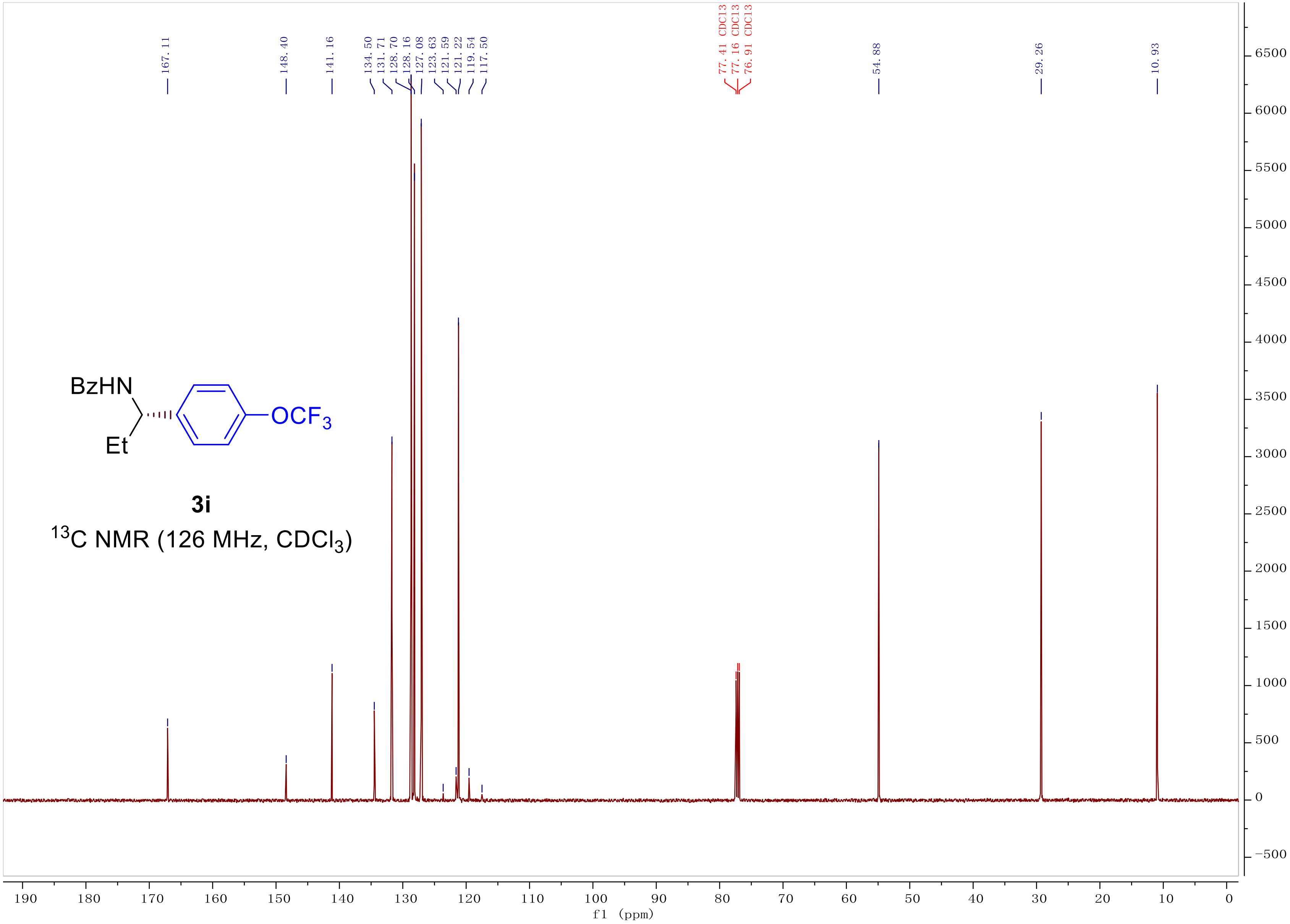

167.11
148.40
141.16
134.50
131.71
128.70
128.16
127.08
123.63
121.59
121.22
119.54
117.50
77.41 CDCl3
77.16 CDCl3
76.91 CDCl3
54.88
29.26
10.93
BzHN
Et
OCF3
3i
13C NMR (126 MHz, CDCl3)
f1 (ppm)

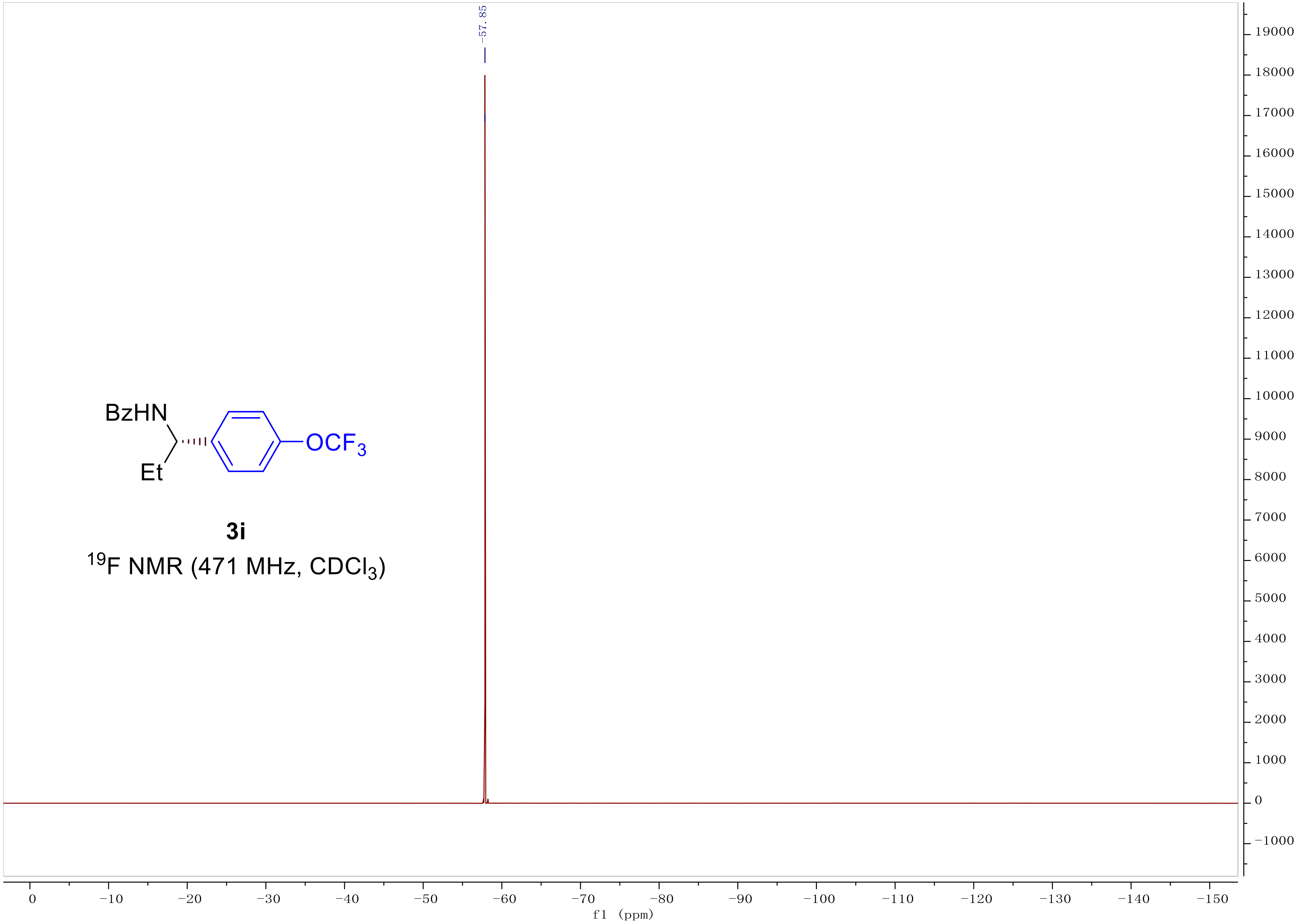

-57.85
BzHN
Et
OCF3
3i
19F NMR (471 MHz, CDCl3)
f1 (ppm)

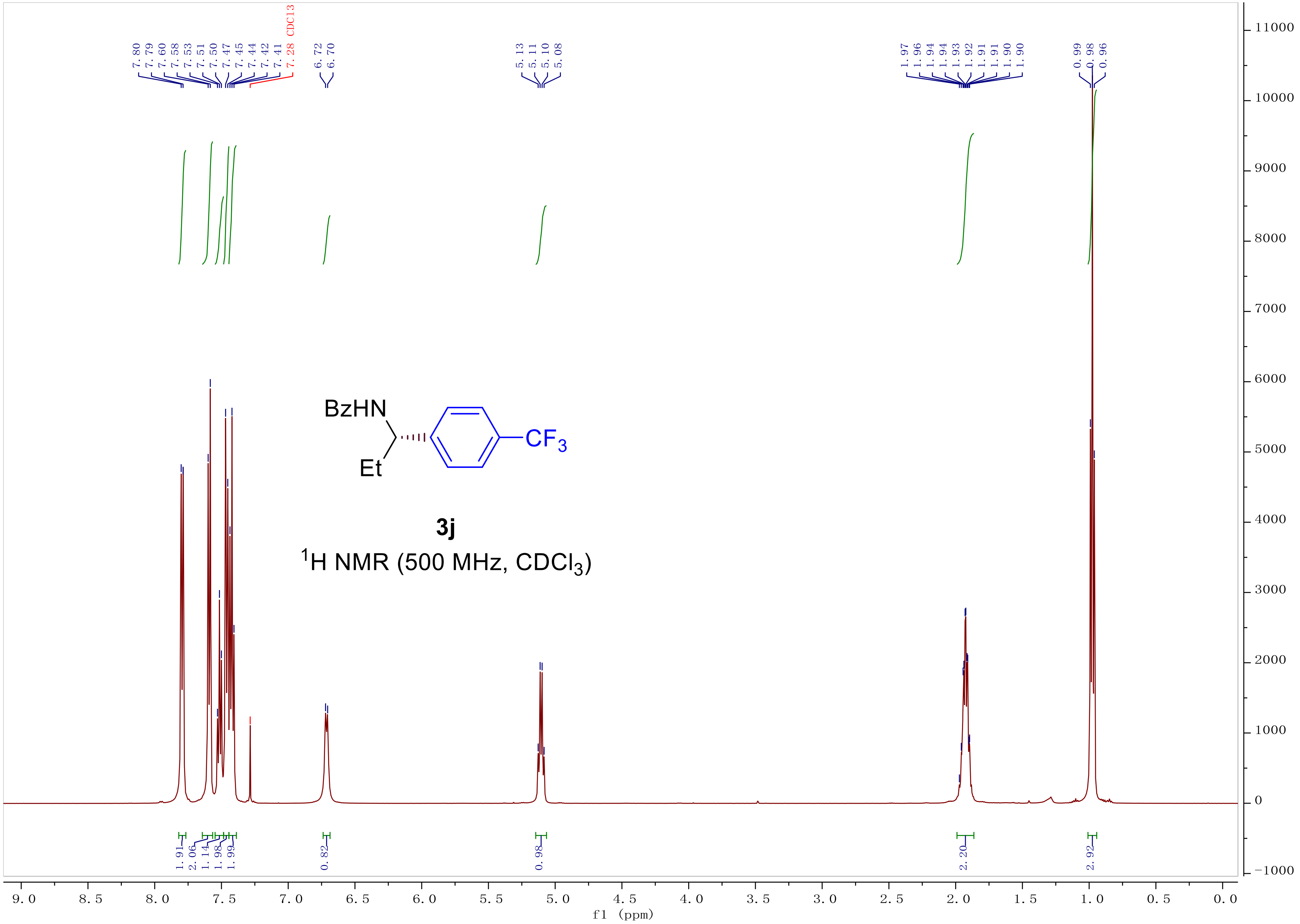
7.80
7.79
7.60
7.58
7.53
7.51
7.50
7.47
7.45
7.44
7.42
7.41
7.28 CDCl3
6.72
6.70
5.13
5.11
5.10
5.08
1.97
1.96
1.94
1.93
1.92
1.91
1.91
1.90
1.90
0.99
0.98
0.96
BzHN
Et
CF3
3j
1H NMR (500 MHz, CDCl3)
1.91
2.06
1.14
1.98
1.99
0.82
0.98
2.20
2.92
f1 (ppm)

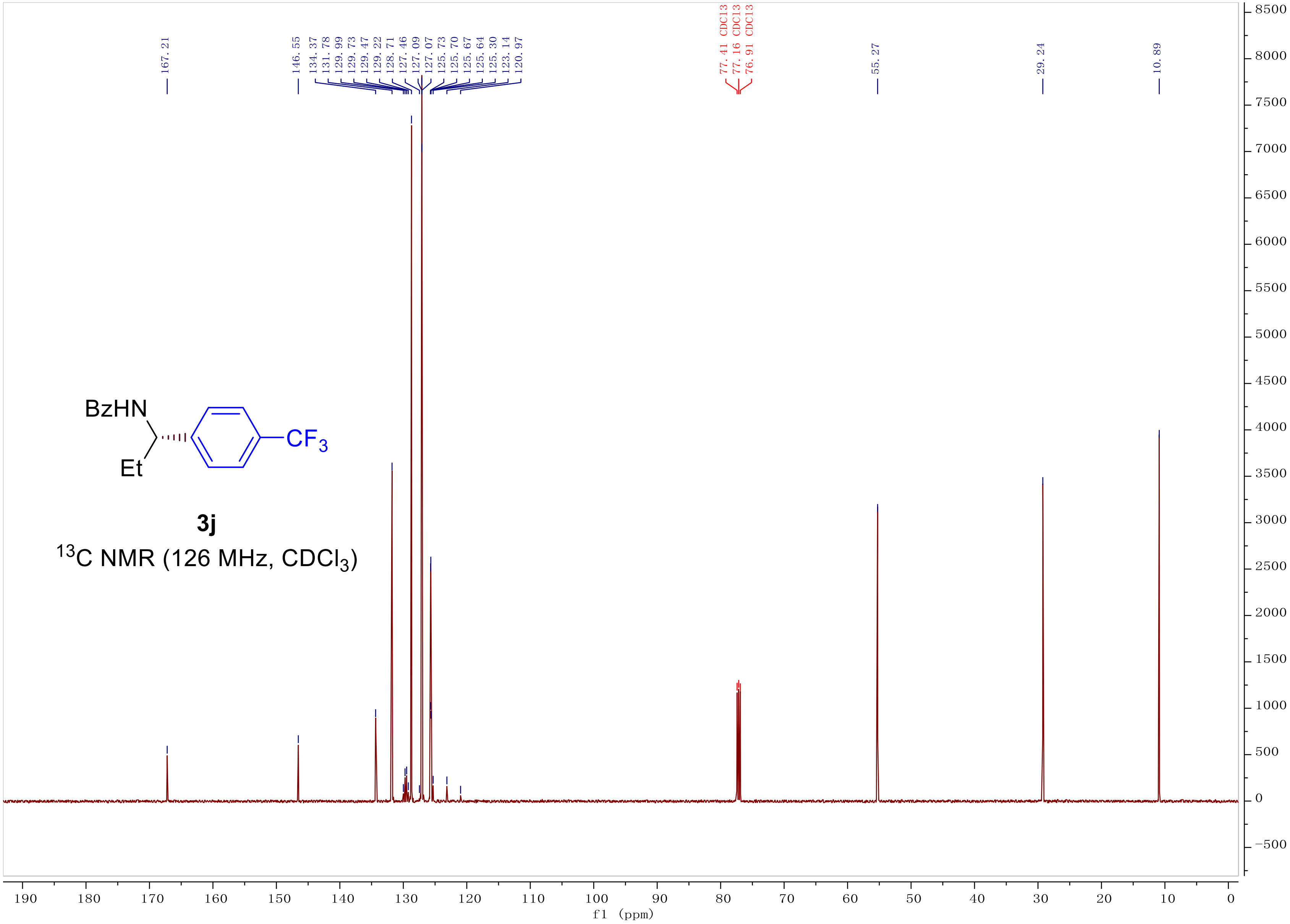
167.21
146.55
134.37
131.78
129.99
129.73
129.47
129.22
128.71
127.46
127.09
127.07
125.73
125.70
125.67
125.64
125.30
123.14
120.97
77.41 CDCl3
77.16 CDCl3
76.91 CDCl3
55.27
29.24
10.89
BzHN
Et
CF3
3j
13C NMR (126 MHz, CDCl3)
f1 (ppm)

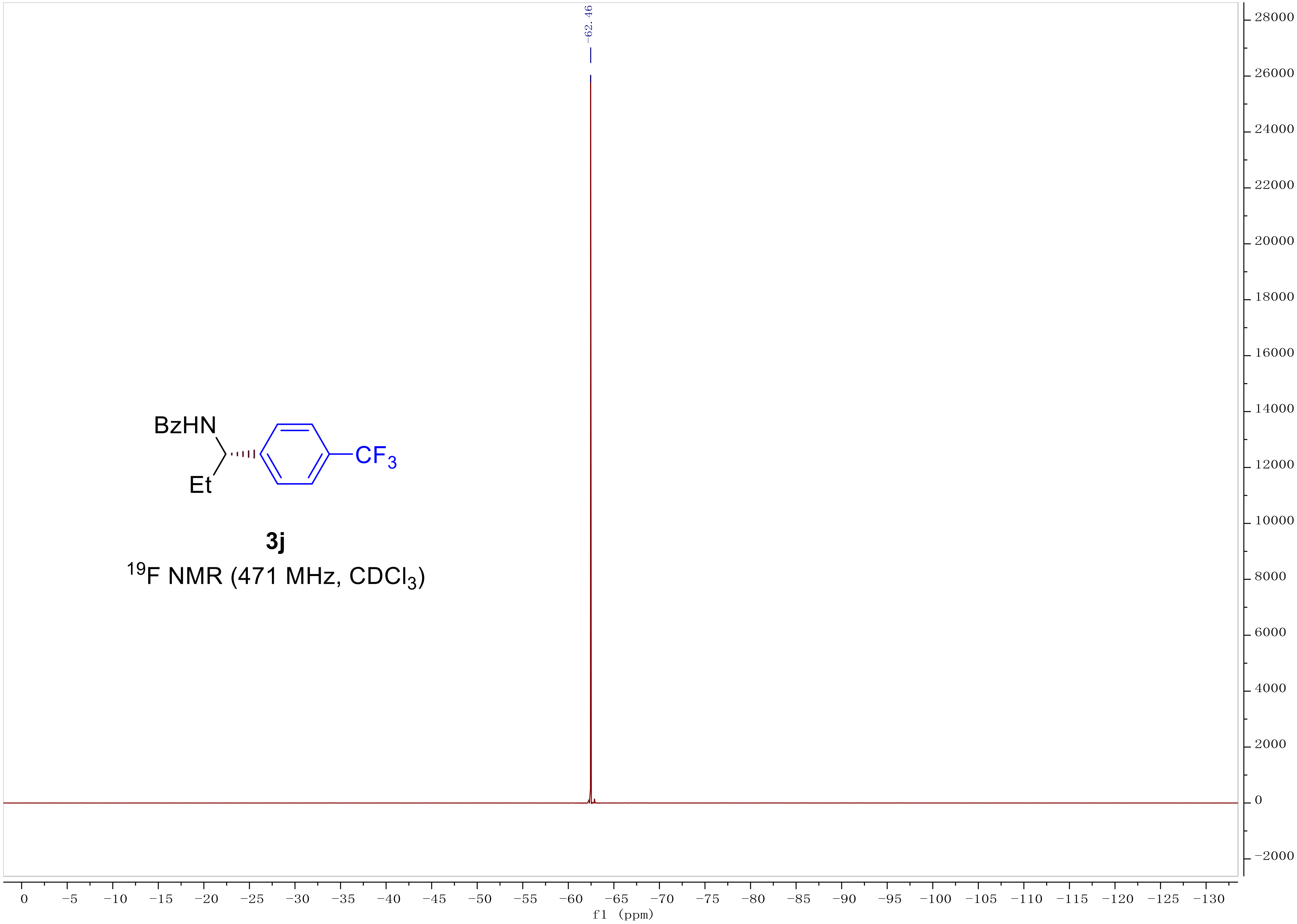

3j

^{19}F NMR (471 MHz, $CDCl_3$)

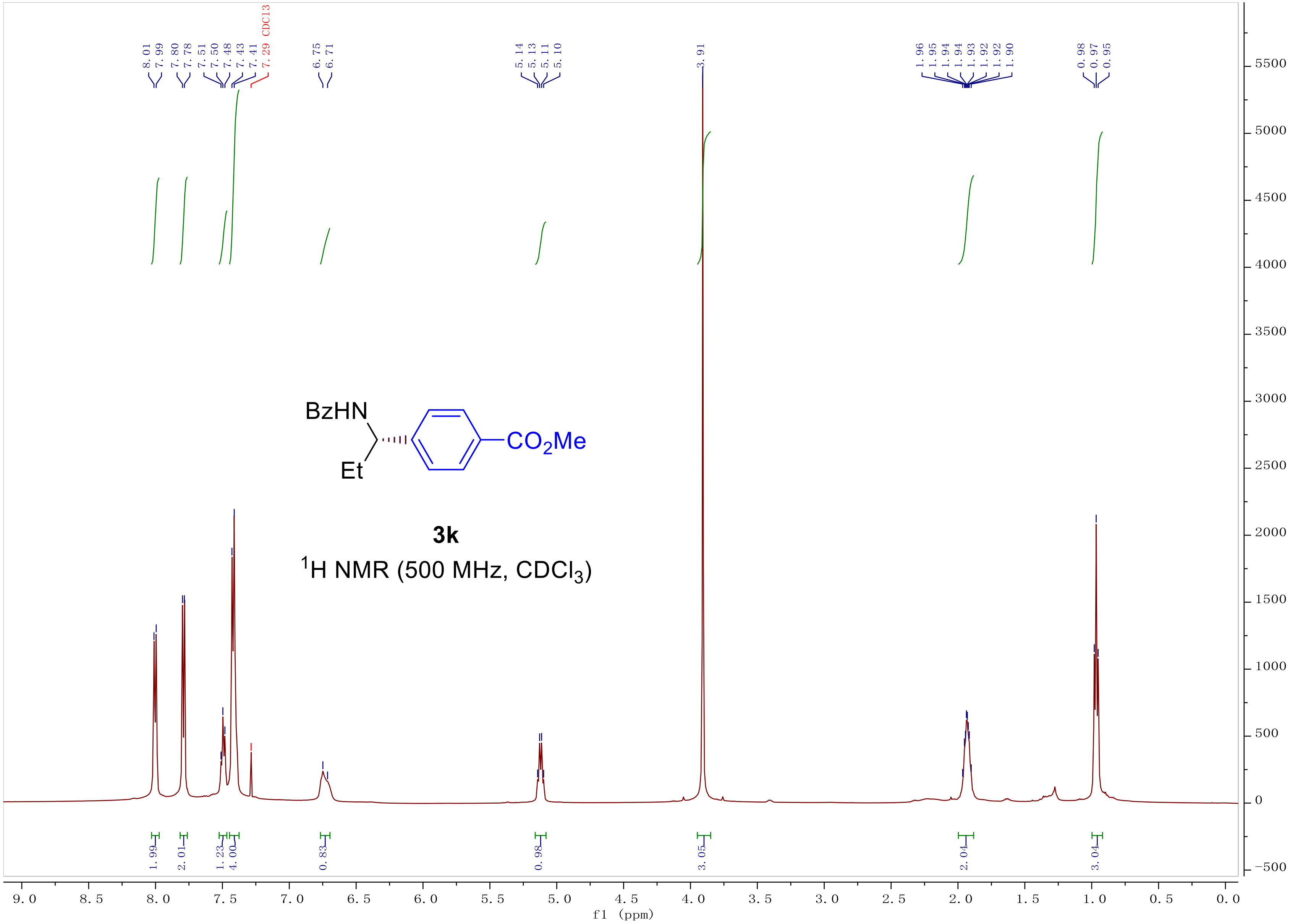
BzHN
Et
CO2Me
3k
1H NMR (500 MHz, CDCl3)
f1 (ppm)

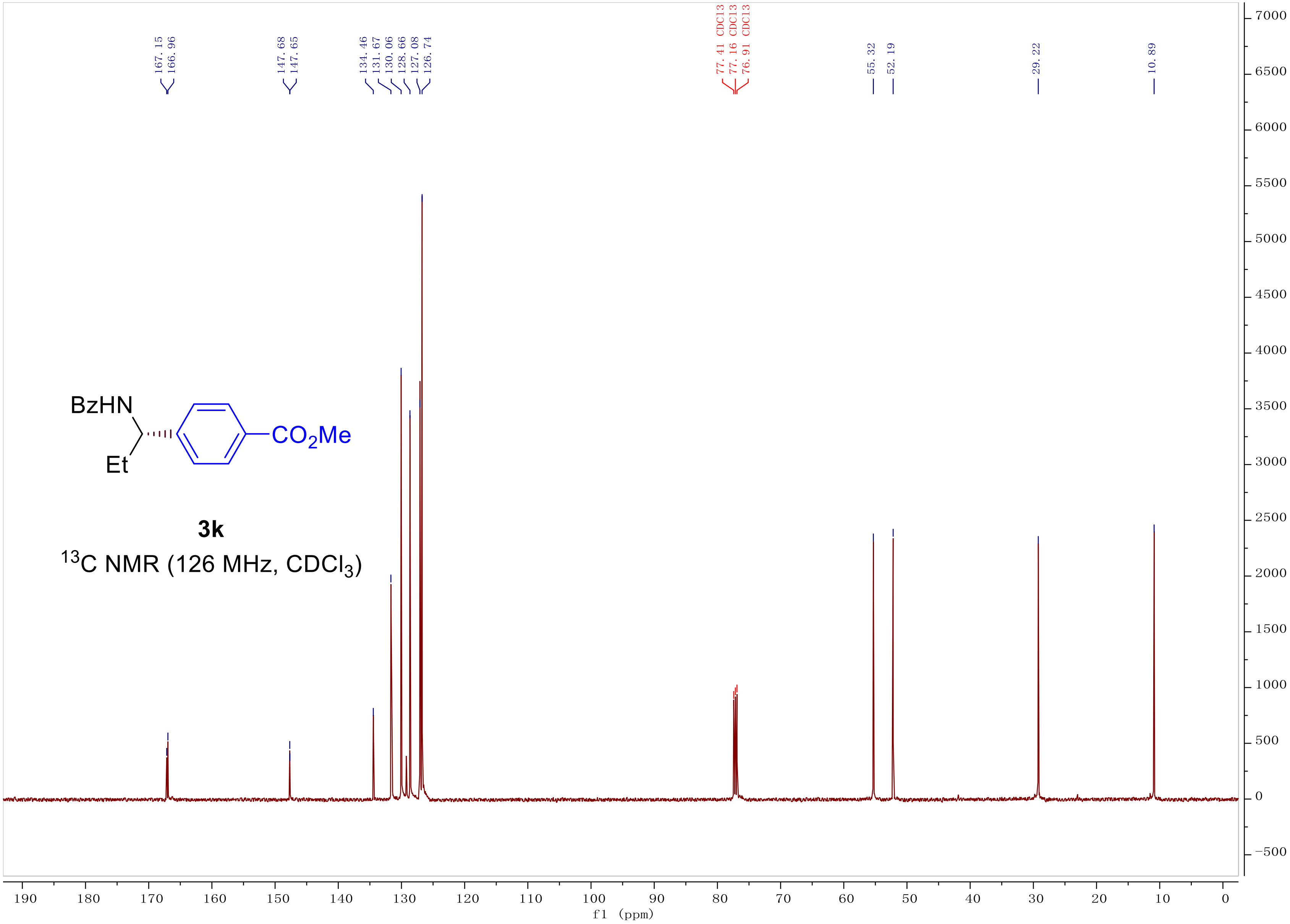

167.15
166.96
147.68
147.65
134.46
131.67
130.06
128.66
127.08
126.74
77.41 CDCl3
77.16 CDCl3
76.91 CDCl3
55.32
52.19
29.22
10.89
BzHN
Et
CO_2Me
3k
^{13}C NMR (126 MHz, $CDCl_3$)
f1 (ppm)

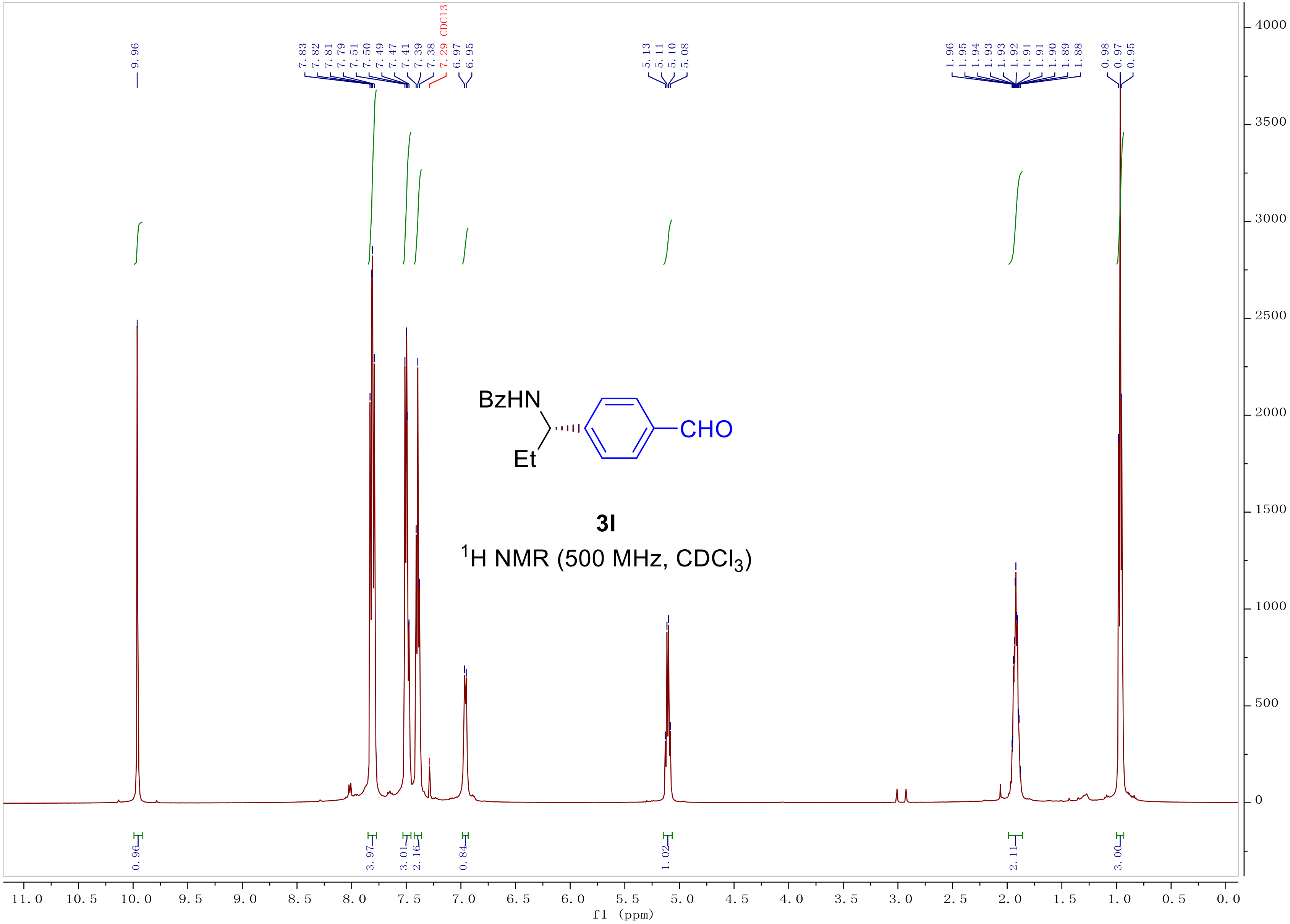
BzHN
Et
CHO
3l
1H NMR (500 MHz, CDCl3)
9.96
7.83
7.82
7.81
7.79
7.51
7.50
7.49
7.47
7.41
7.39
7.38
7.29 CDCl3
6.97
6.95
5.13
5.11
5.10
5.08
1.96
1.95
1.94
1.93
1.93
1.92
1.91
1.91
1.90
1.89
1.88
0.98
0.97
0.95
0.96
3.97
3.01
2.16
0.84
1.02
2.11
3.00
f1 (ppm)

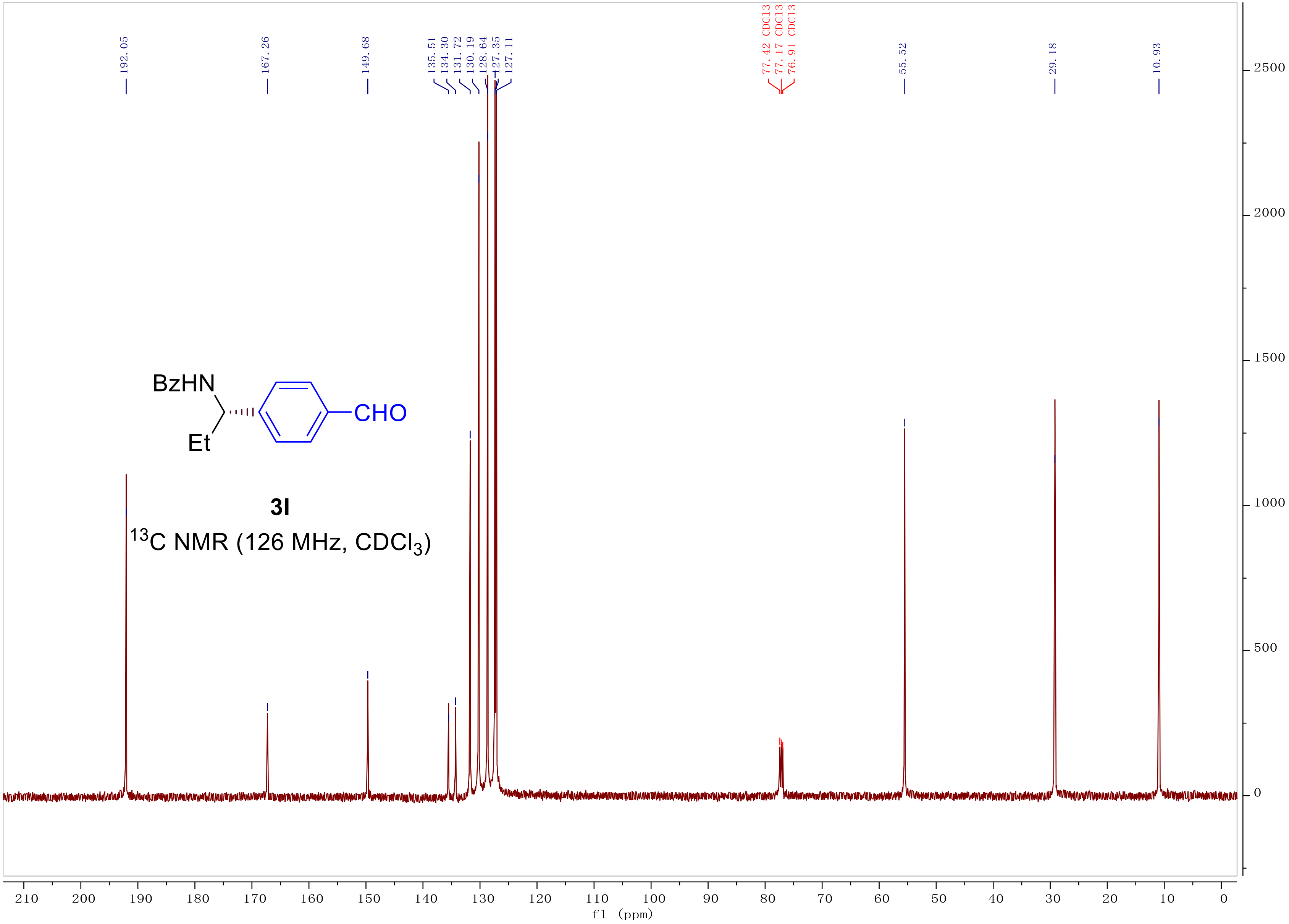
192.05
167.26
149.68
135.51
134.30
131.72
130.19
128.64
127.35
127.11
77.42 CDCl3
77.17 CDCl3
76.91 CDCl3
55.52
29.18
10.93
BzHN
Et
CHO
3l
^{13}C NMR (126 MHz, CDCl$_3$)
2500
2000
1500
1000
500
0
210 200 190 180 170 160 150 140 130 120 110 100 90 80 70 60 50 40 30 20 10 0
f1 (ppm)

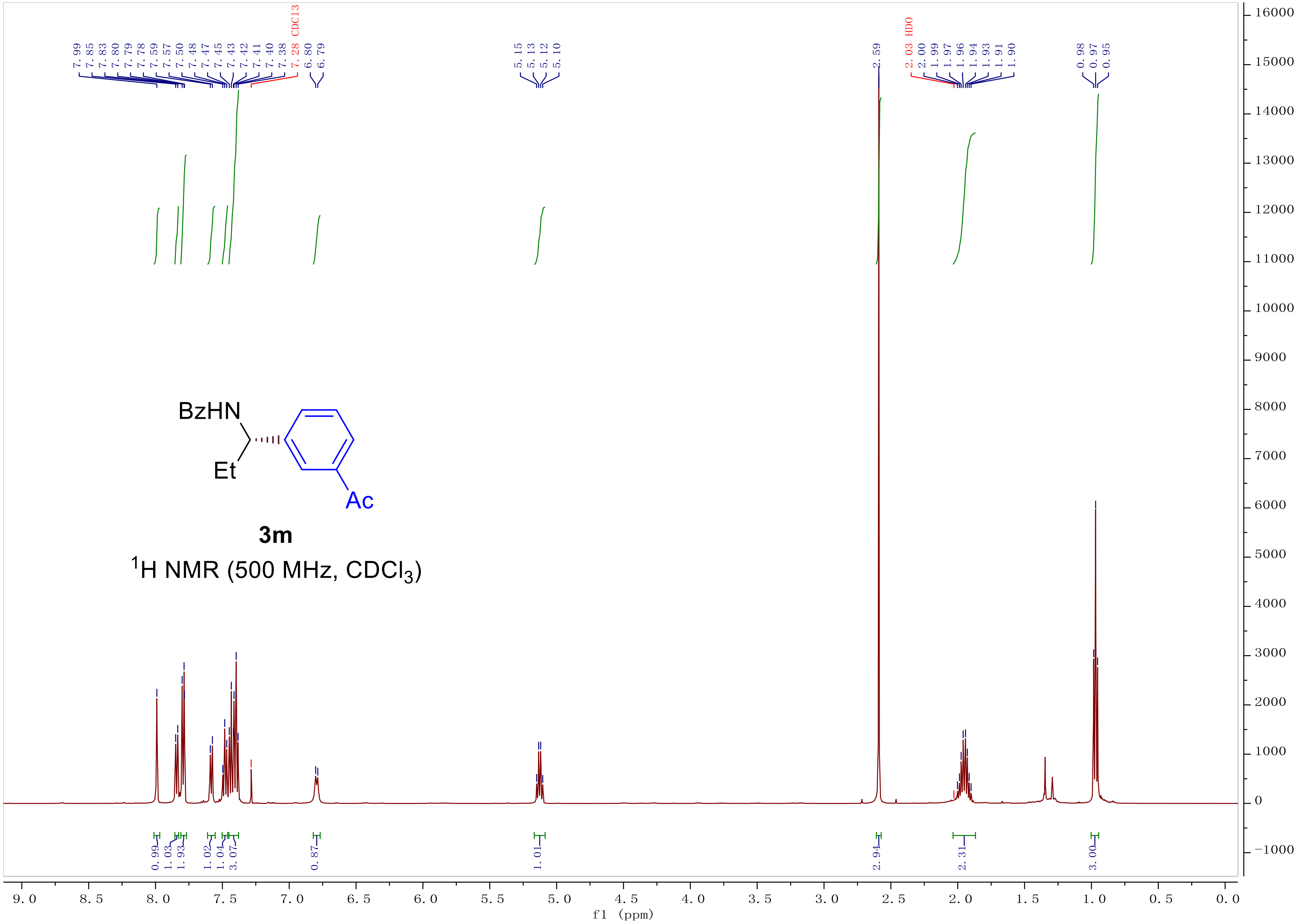
BzHN
Et
Ac
3m
^{1}H NMR (500 MHz, CDCl$_3$)
f1 (ppm)
CDCl3
HDO

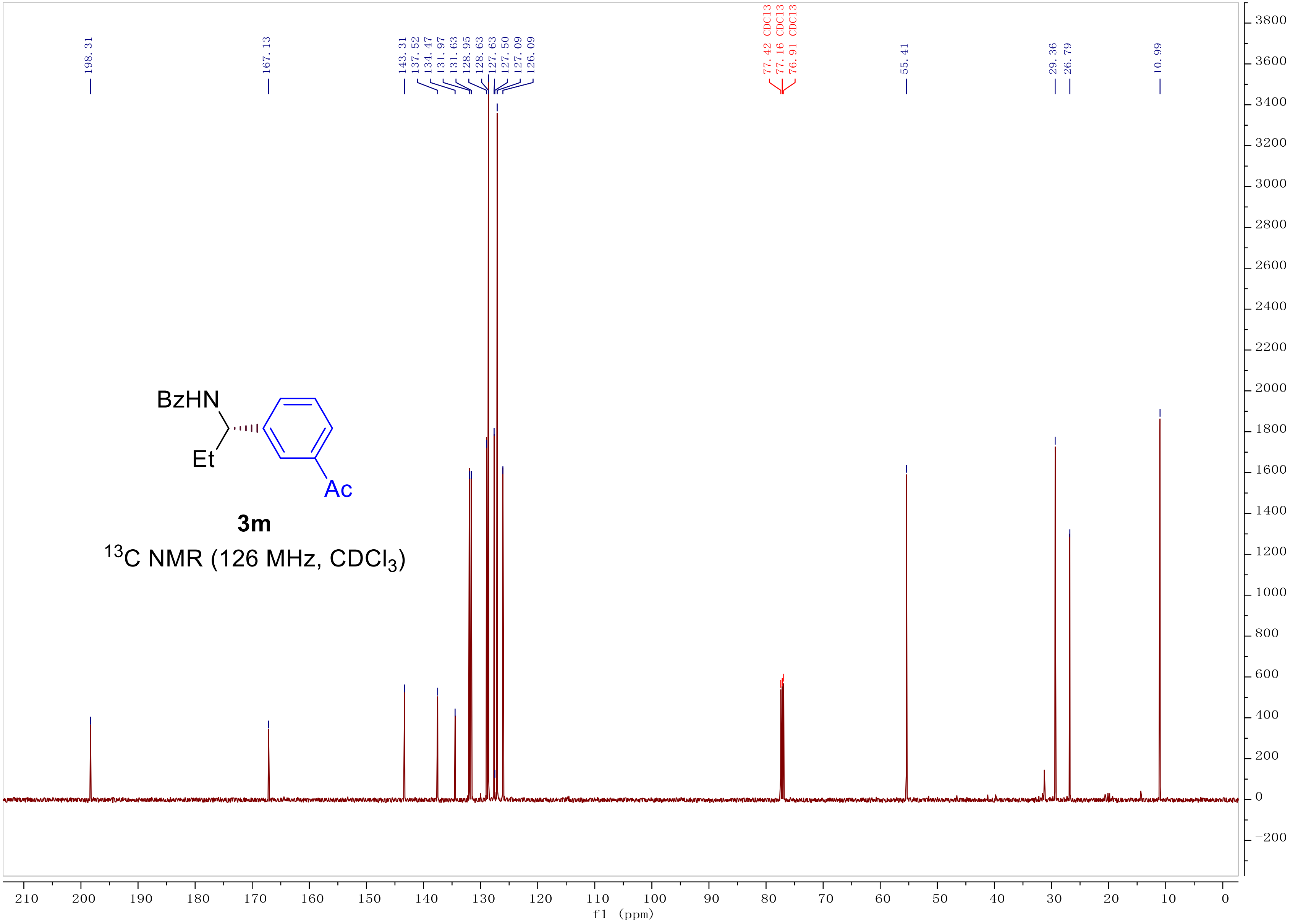
198.31
167.13
143.31
137.52
134.47
131.97
131.63
128.95
128.63
127.63
127.50
127.09
126.09
77.42 CDCl3
77.16 CDCl3
76.91 CDCl3
55.41
29.36
26.79
10.99
BzHN
Et
Ac
3m
^{13}C NMR (126 MHz, $CDCl_3$)
f1 (ppm)

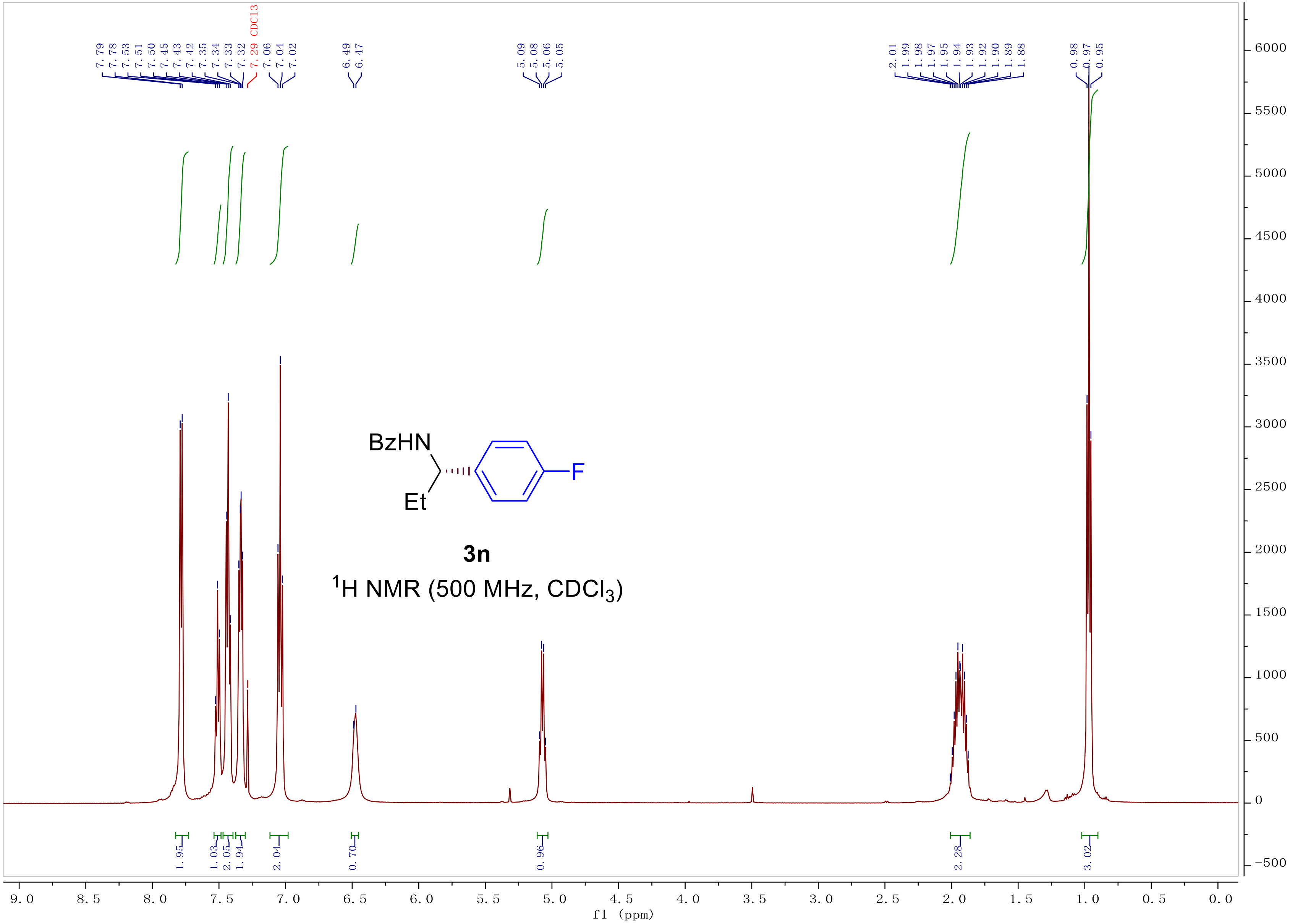

7.79
7.78
7.53
7.51
7.50
7.45
7.43
7.42
7.35
7.34
7.33
7.32
7.29 CDCl3
7.06
7.04
7.02
6.49
6.47
5.09
5.08
5.06
5.05
2.01
1.99
1.98
1.97
1.95
1.94
1.93
1.92
1.90
1.89
1.88
0.98
0.97
0.95
BzHN
Et
F
3n
^{1}H NMR (500 MHz, $CDCl_3$)
1.95
1.03
2.05
1.94
2.04
0.70
0.96
2.28
3.02
f1 (ppm)

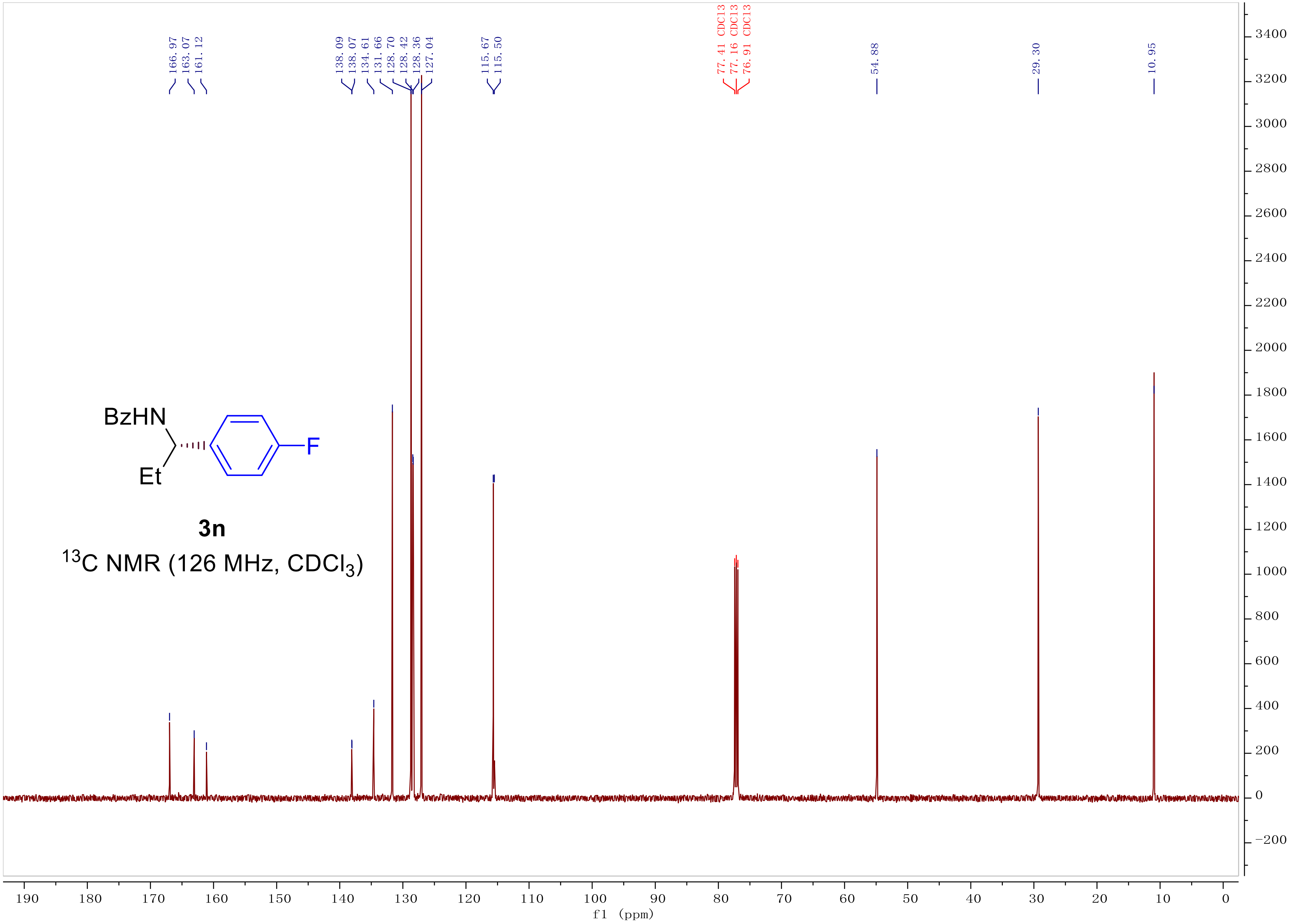

166.97
163.07
161.12
138.09
138.07
134.61
131.66
128.70
128.42
128.36
127.04
115.67
115.50
77.41 CDCl3
77.16 CDCl3
76.91 CDCl3
54.88
29.30
10.95
BzHN
Et
F
3n
13C NMR (126 MHz, CDCl3)
f1 (ppm)

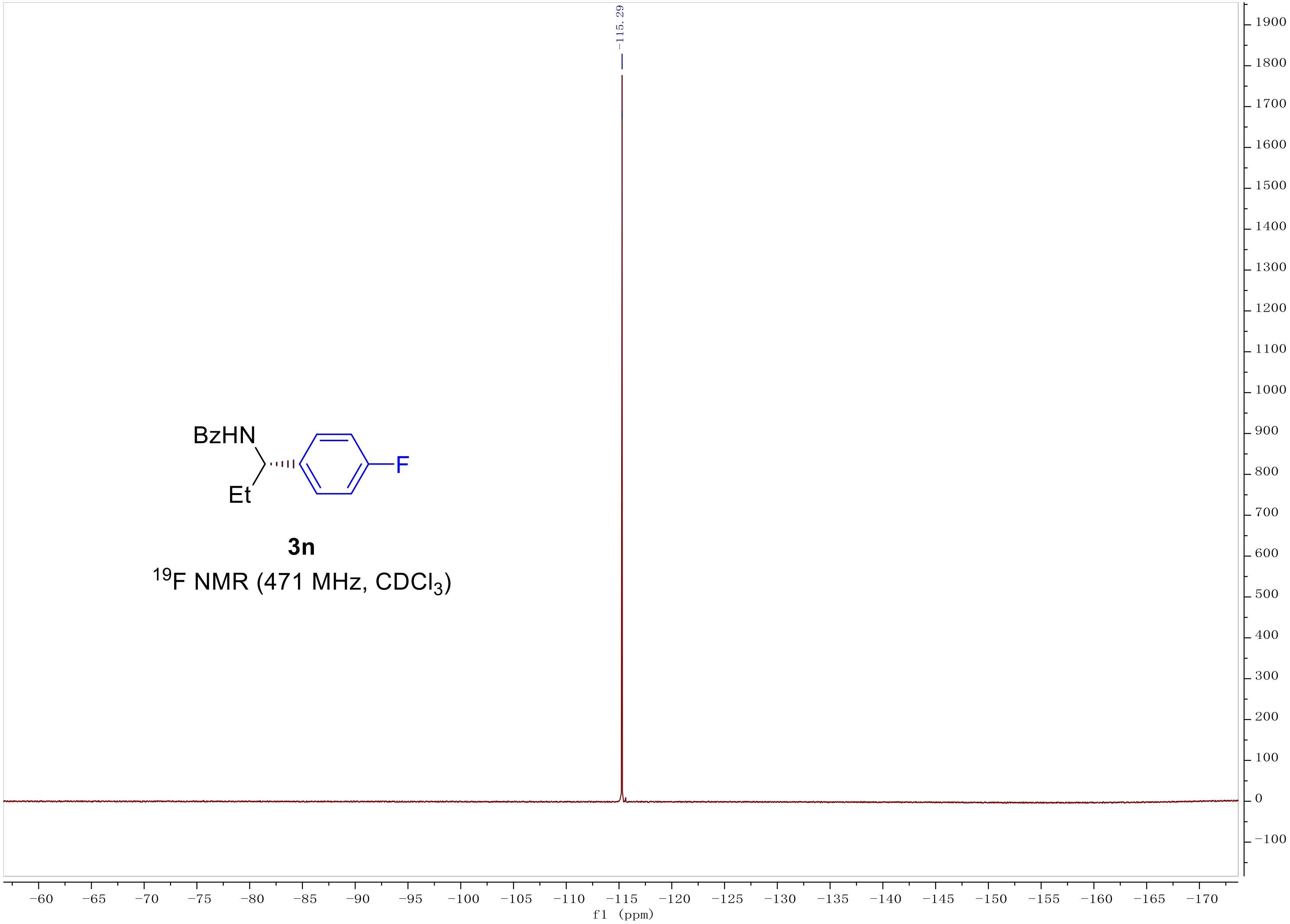
-115.29
BzHN
Et
F
3n
^{19}F NMR (471 MHz, $CDCl_3$)
f1 (ppm)

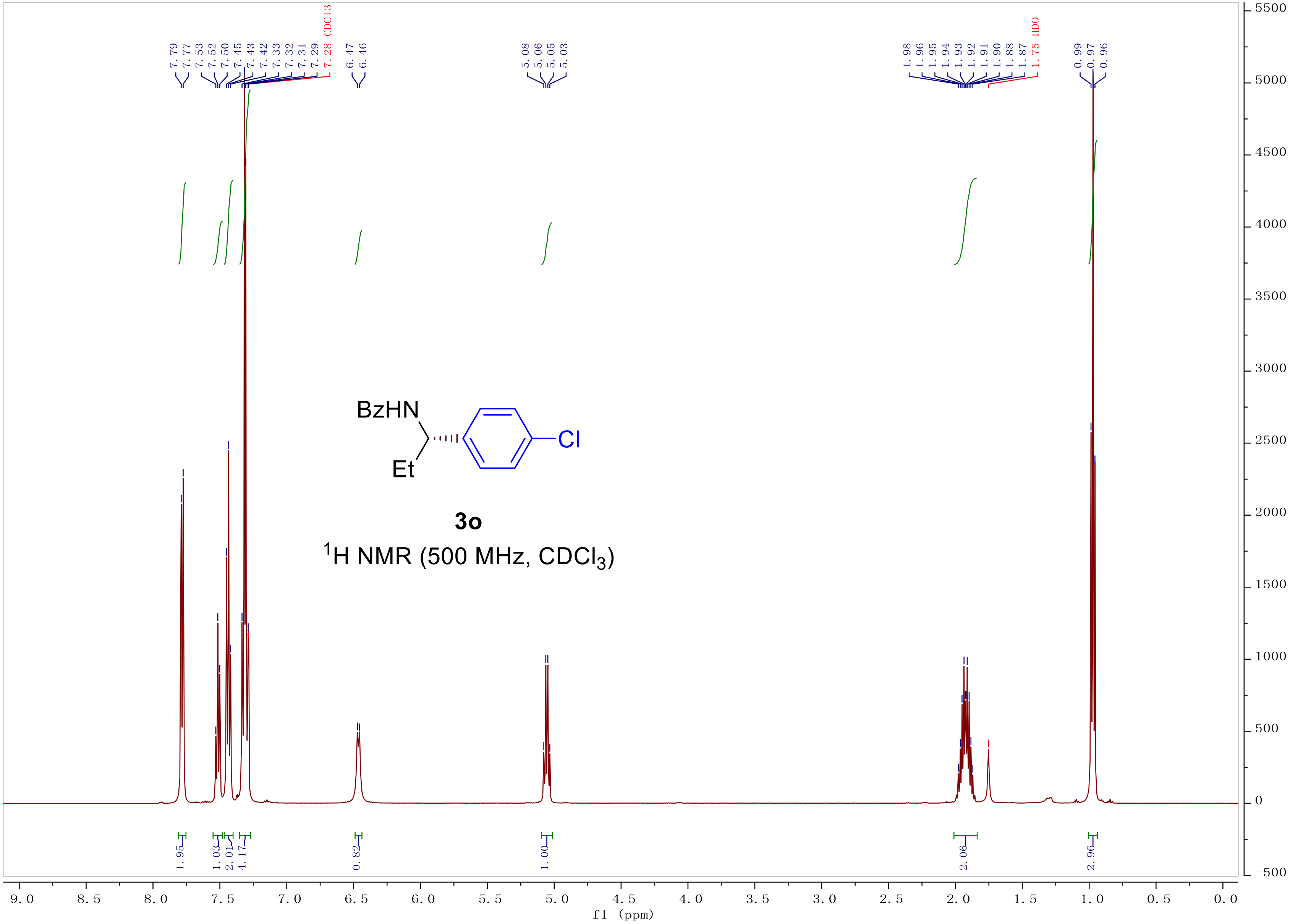
BzHN
Et
Cl
3o
1H NMR (500 MHz, CDCl3)
f1 (ppm)

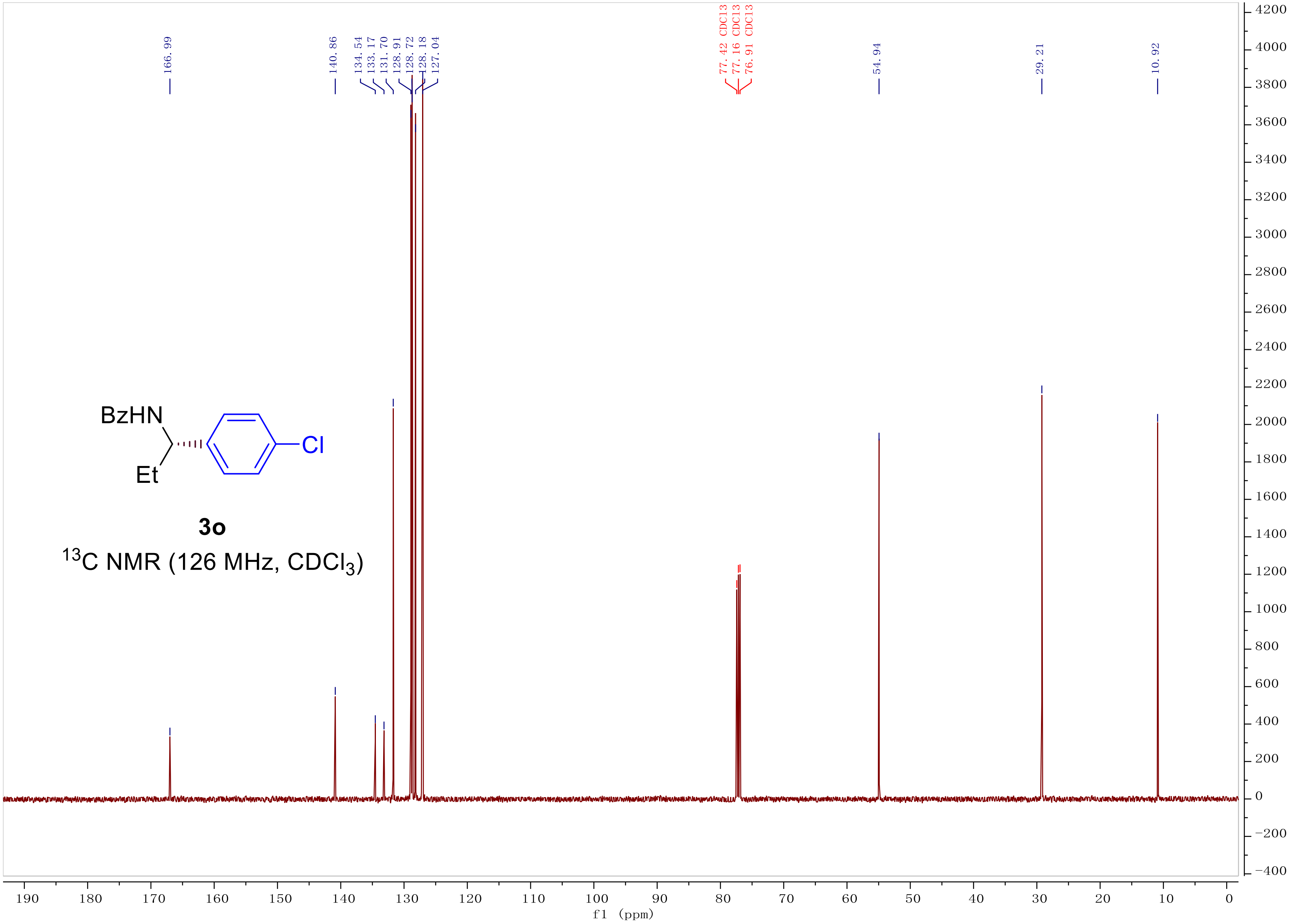

166.99
140.86
134.54
133.17
131.70
128.91
128.72
128.18
127.04
77.42 CDCl3
77.16 CDCl3
76.91 CDCl3
54.94
29.21
10.92
BzHN
Et
Cl
3o
13C NMR (126 MHz, CDCl3)
f1 (ppm)

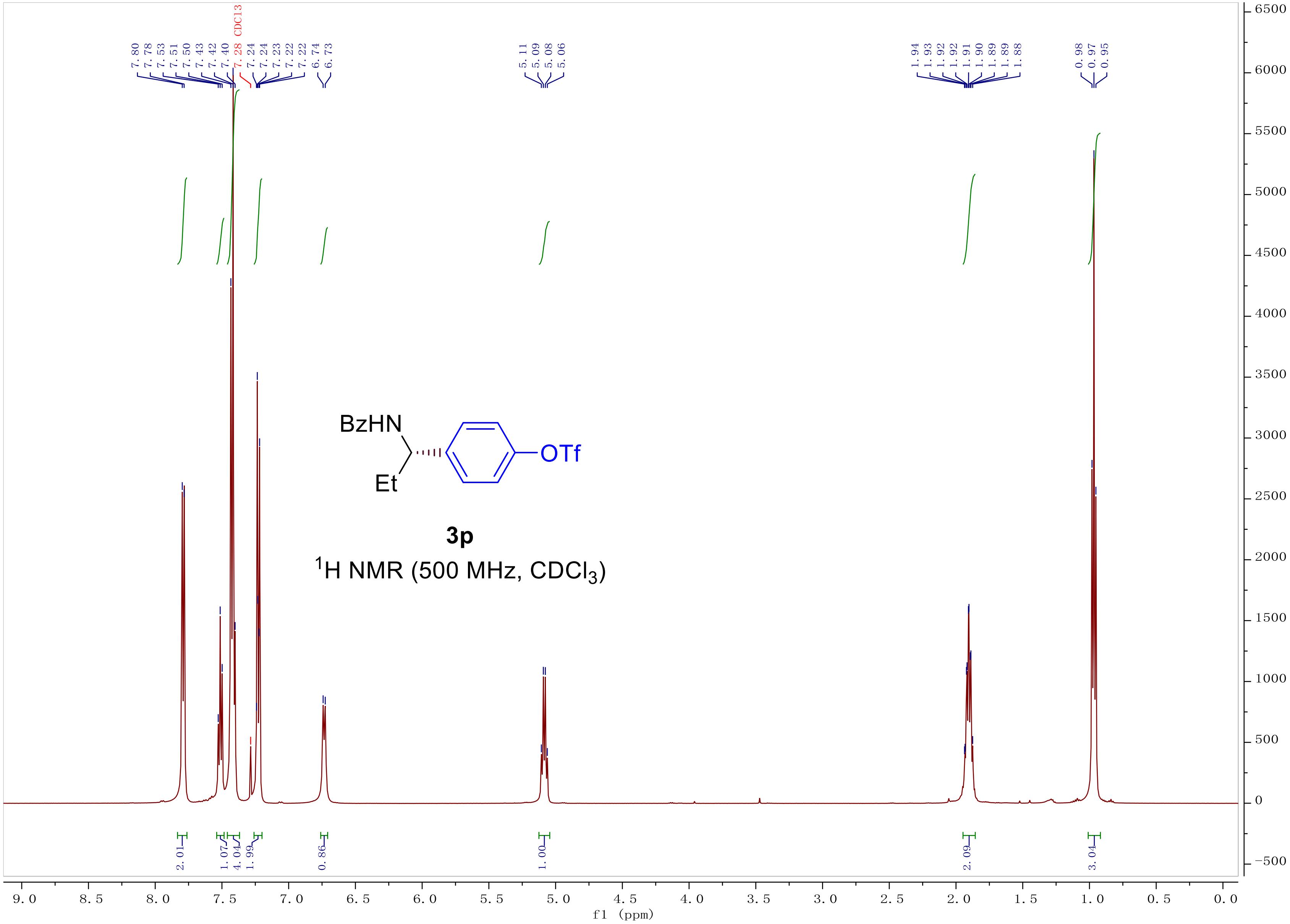

7.80
7.78
7.53
7.51
7.50
7.43
7.42
7.40
7.28 CDCl3
7.24
7.24
7.23
7.22
7.22
6.74
6.73
5.11
5.09
5.08
5.06
1.94
1.93
1.92
1.92
1.91
1.90
1.89
1.89
1.88
0.98
0.97
0.95
BzHN
OTf
Et
3p
^{1}H NMR (500 MHz, CDCl$_3$)
2.01
1.07
4.04
1.99
0.86
1.00
2.09
3.04
f1 (ppm)

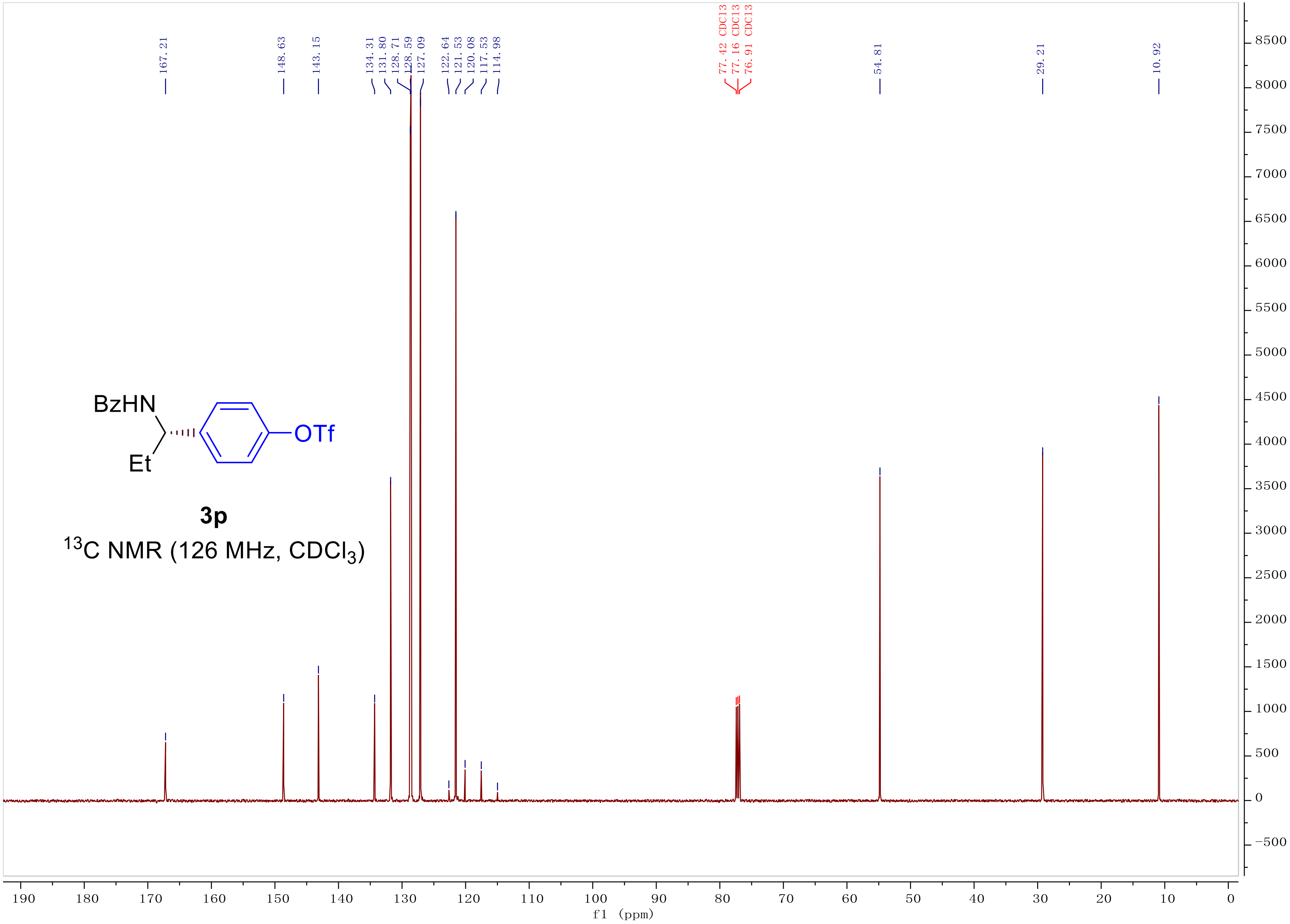
167.21
148.63
143.15
134.31
131.80
128.71
128.59
127.09
122.64
121.53
120.08
117.53
114.98
77.42 CDCl3
77.16 CDCl3
76.91 CDCl3
54.81
29.21
10.92
BzHN
Et
OTf
3p
13C NMR (126 MHz, CDCl3)
f1 (ppm)

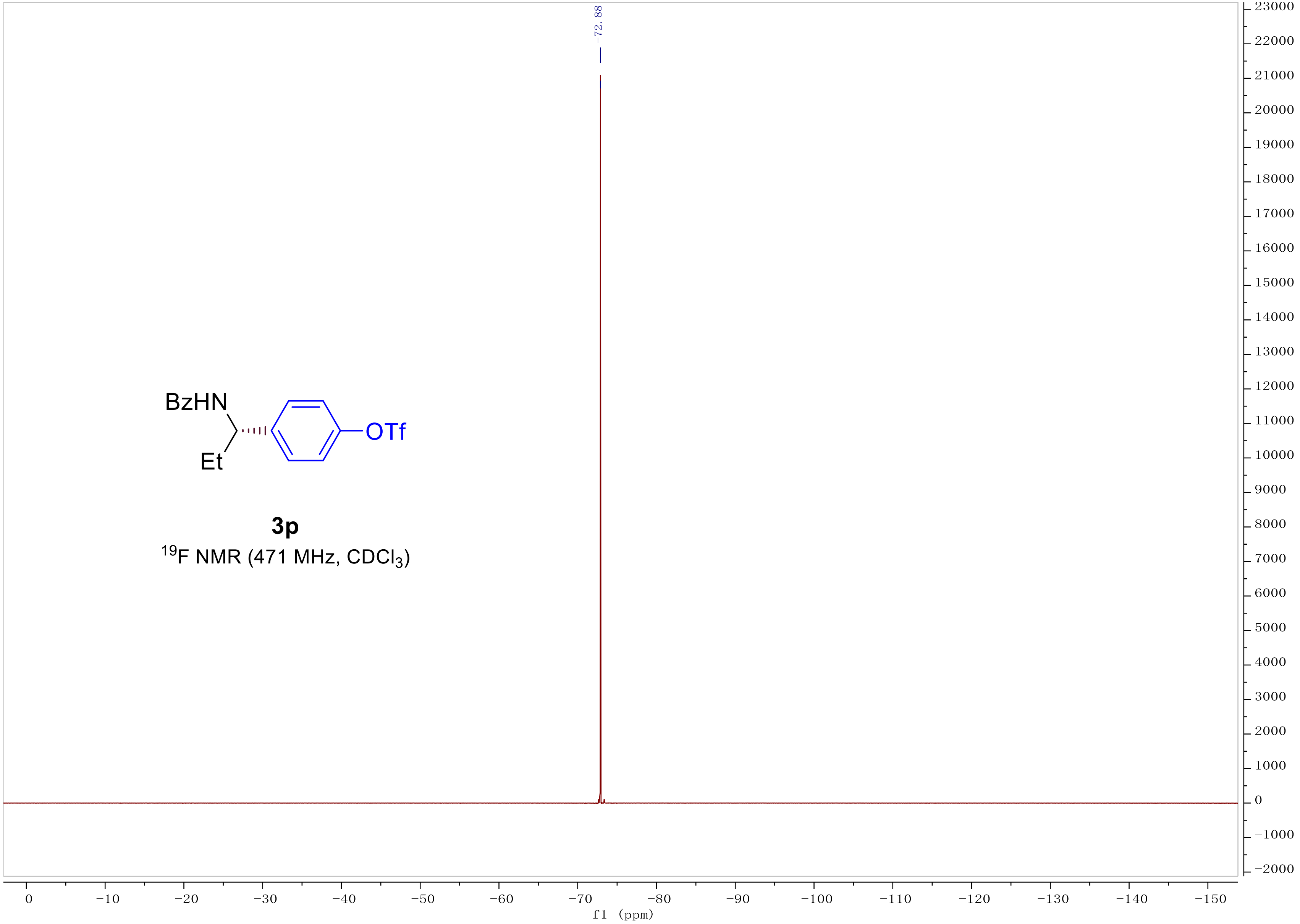

3p

^{19}F NMR (471 MHz, $CDCl_3$)

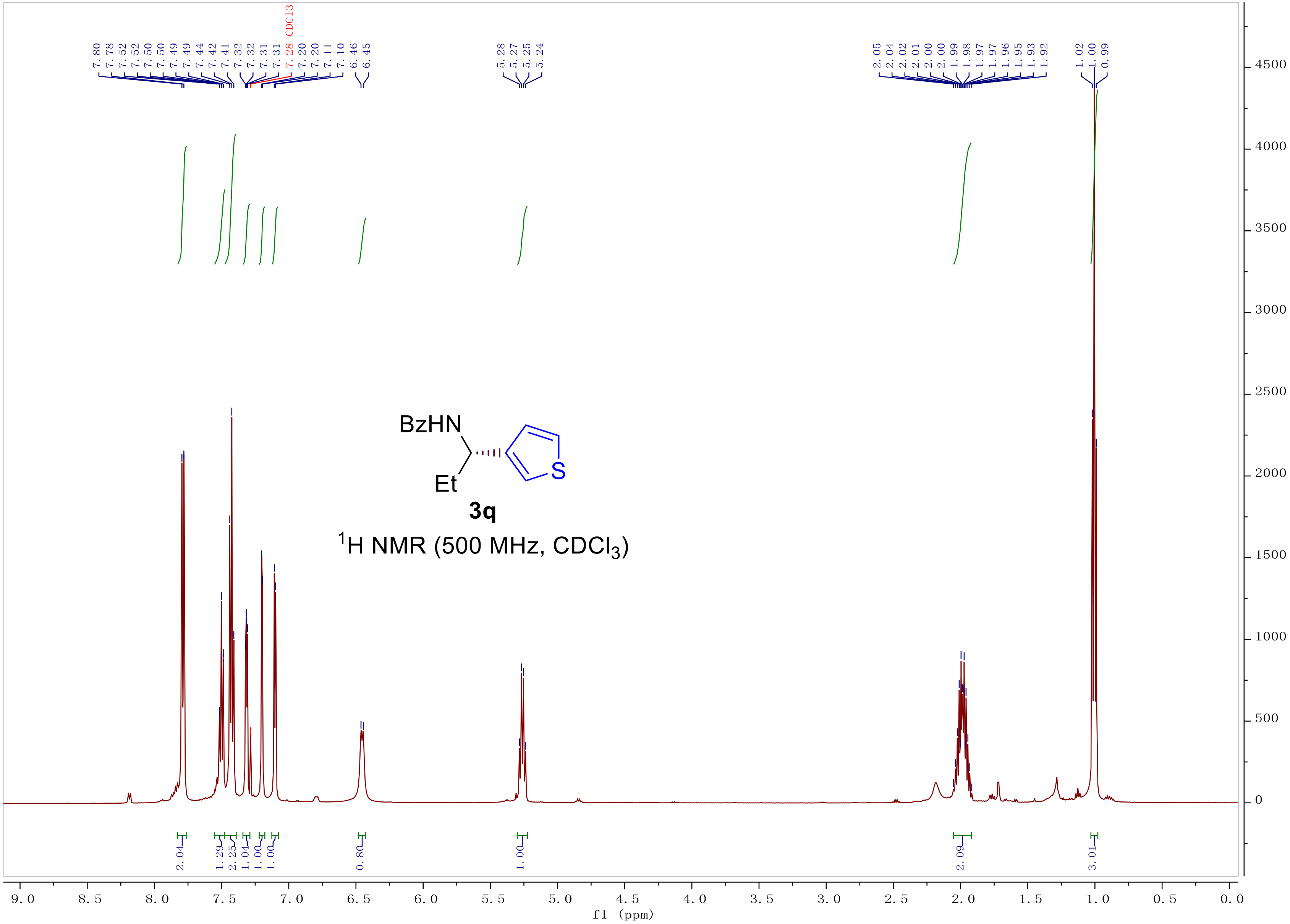

BzHN
Et
S
3q
^{1}H NMR (500 MHz, $CDCl_3$)
f1 (ppm)

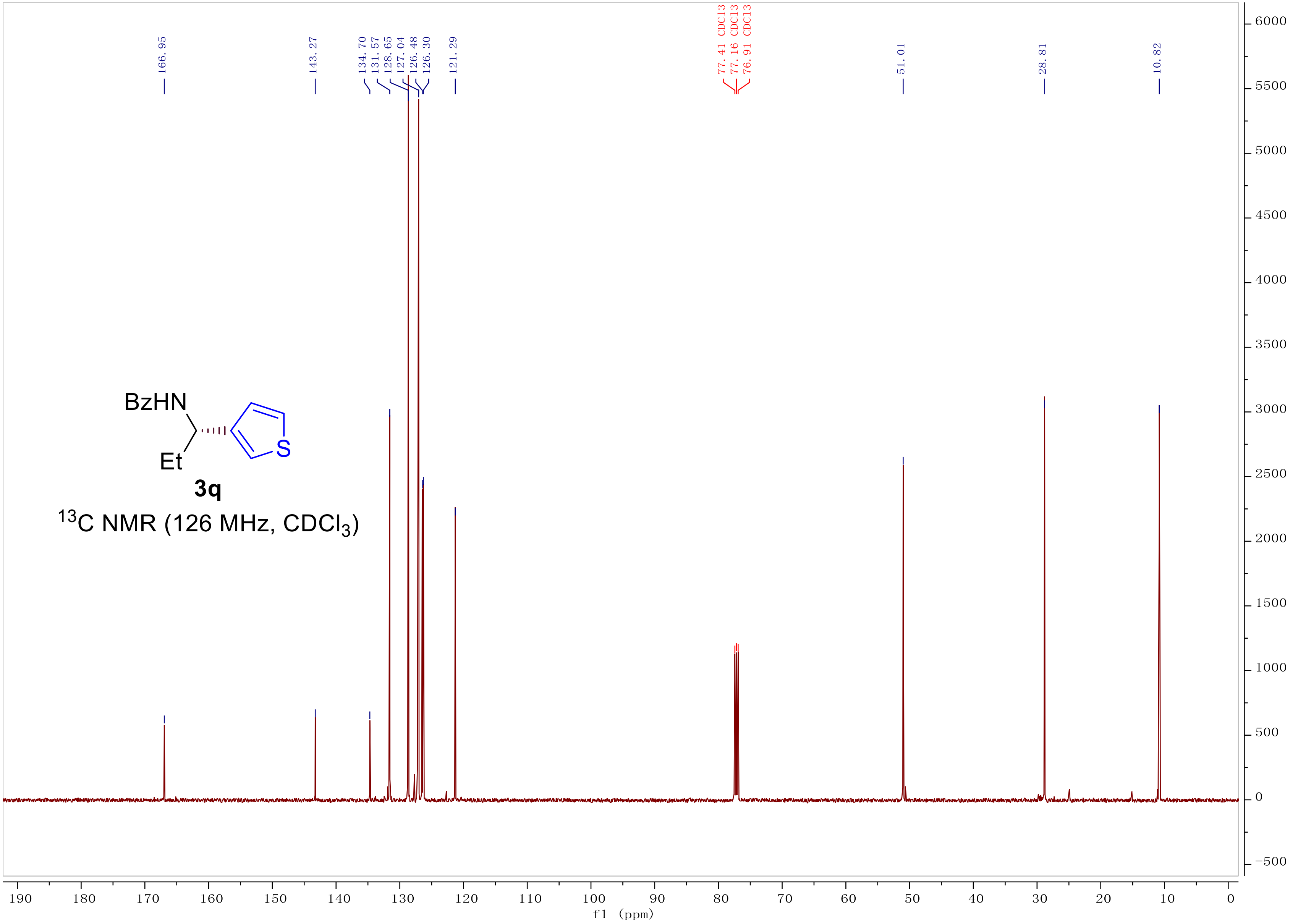
166.95
143.27
134.70
131.57
128.65
127.04
126.48
126.30
121.29
77.41 CDCl3
77.16 CDCl3
76.91 CDCl3
51.01
28.81
10.82
BzHN
Et
S
3q
^{13}C NMR (126 MHz, CDCl$_3$)
f1 (ppm)

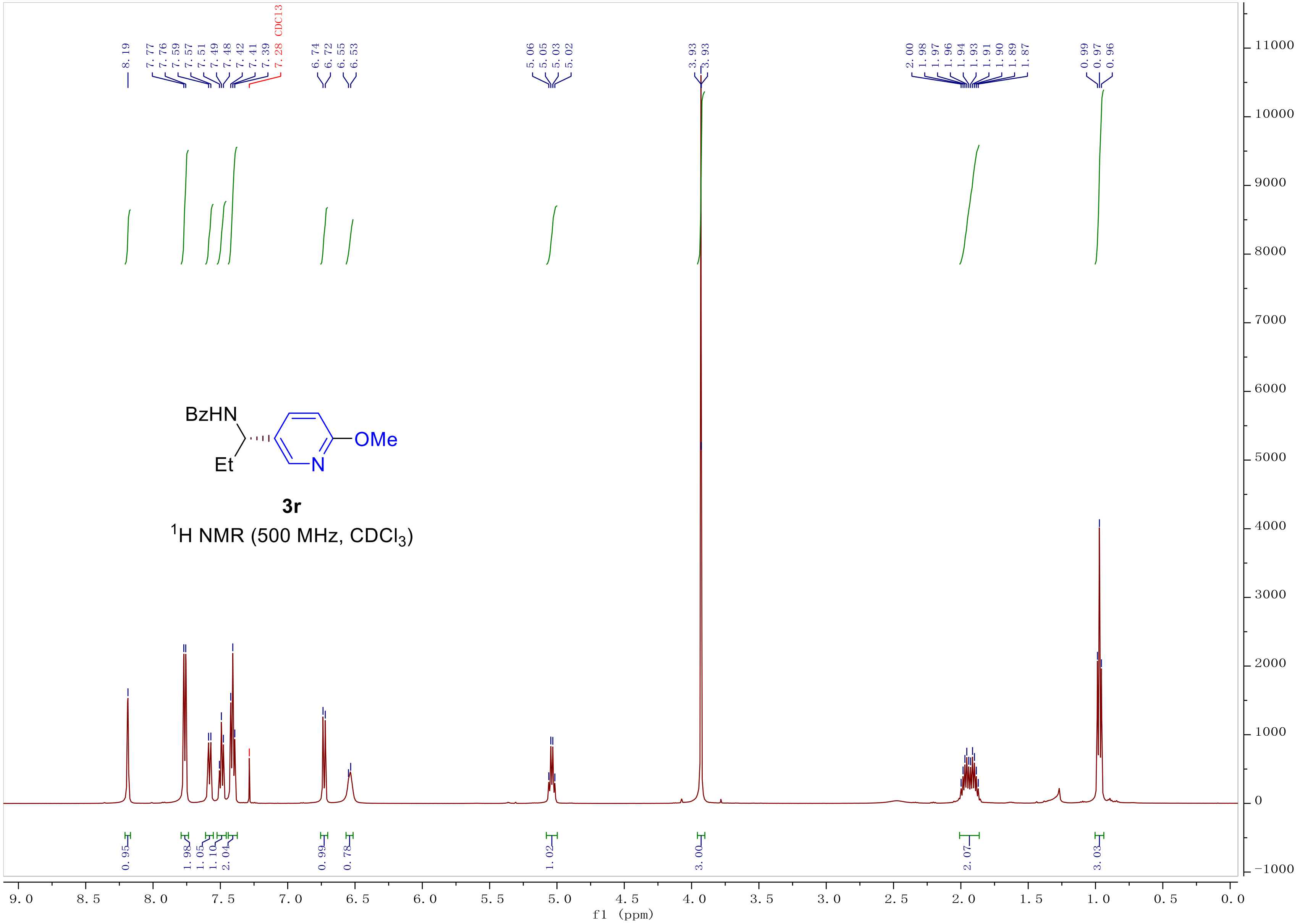

BzHN
Et
OMe
N
3r
^{1}H NMR (500 MHz, $CDCl_3$)
8.19
7.77
7.76
7.59
7.57
7.51
7.49
7.48
7.42
7.41
7.39
7.28 CDCl3
6.74
6.72
6.55
6.53
5.06
5.05
5.03
5.02
3.93
3.93
2.00
1.98
1.97
1.96
1.94
1.93
1.91
1.90
1.89
1.87
0.99
0.97
0.96
0.95
1.98
1.05
1.10
2.04
0.99
0.78
1.02
3.00
2.07
3.03
f1 (ppm)

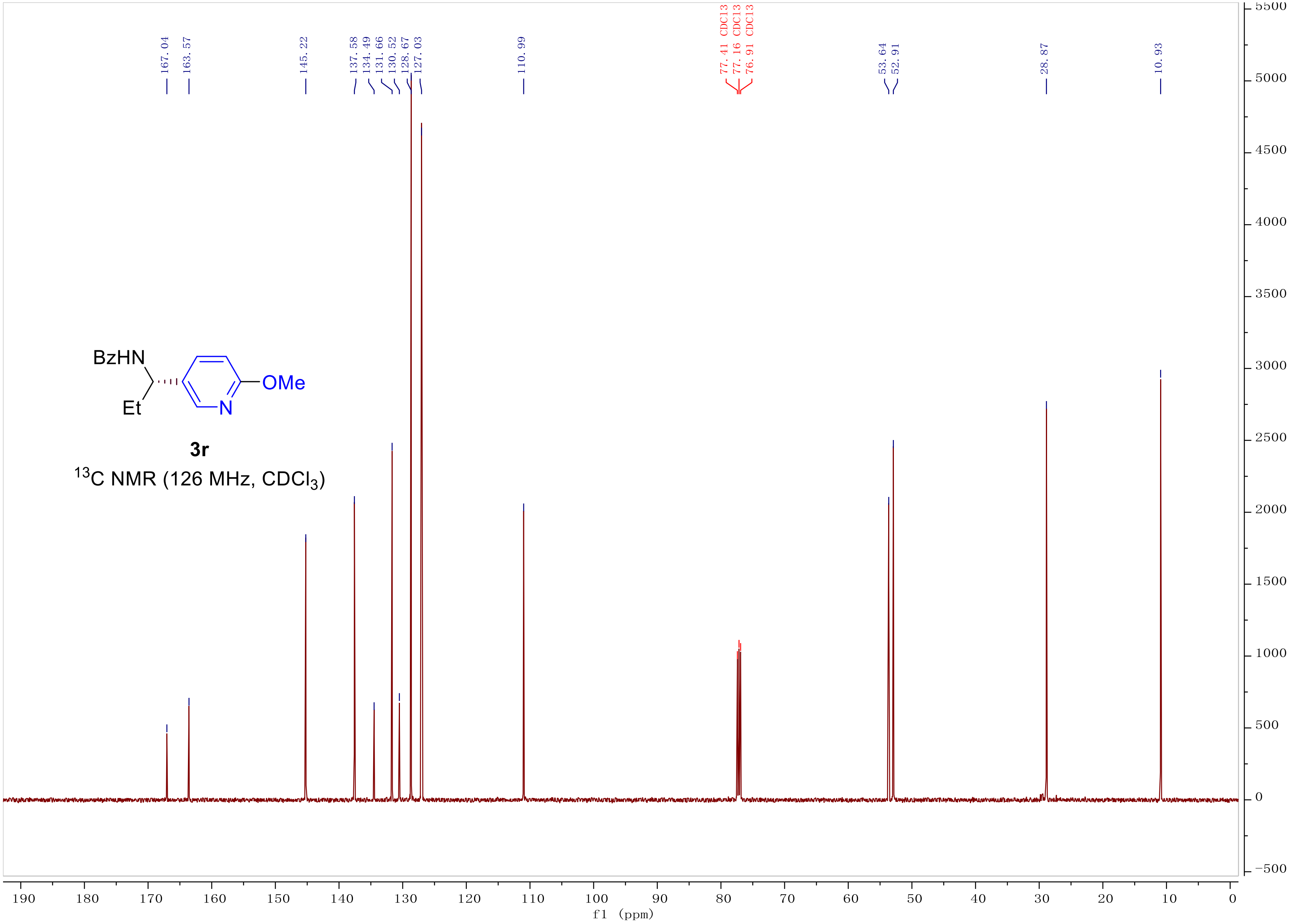

167.04
163.57
145.22
137.58
134.49
131.66
130.52
128.67
127.03
110.99
77.41 CDCl3
77.16 CDCl3
76.91 CDCl3
53.64
52.91
28.87
10.93
BzHN
OMe
N
Et
3r
^{13}C NMR (126 MHz, CDCl$_3$)
f1 (ppm)

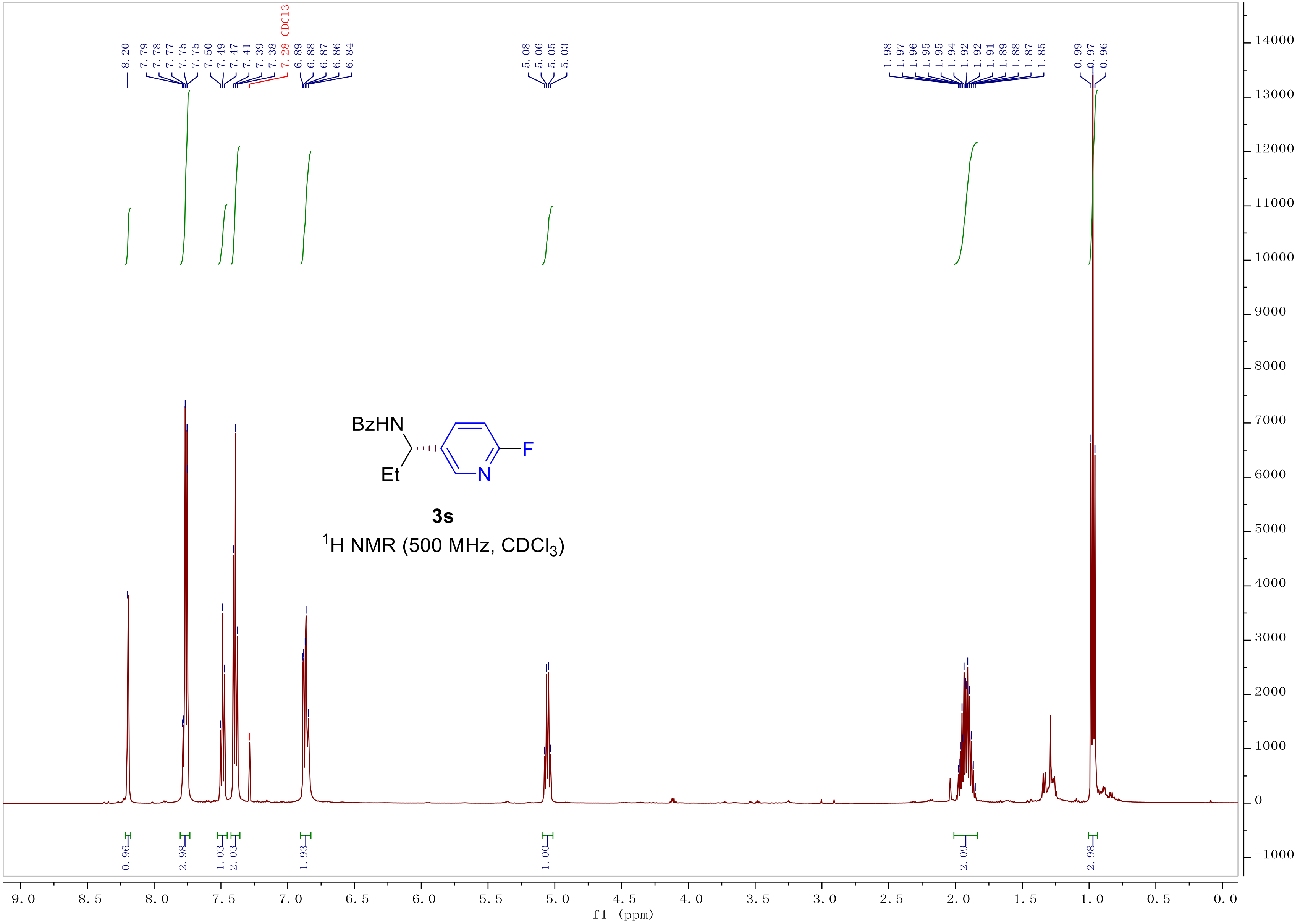

BzHN
Et
F
N
3s
^{1}H NMR (500 MHz, CDCl$_3$)
f1 (ppm)

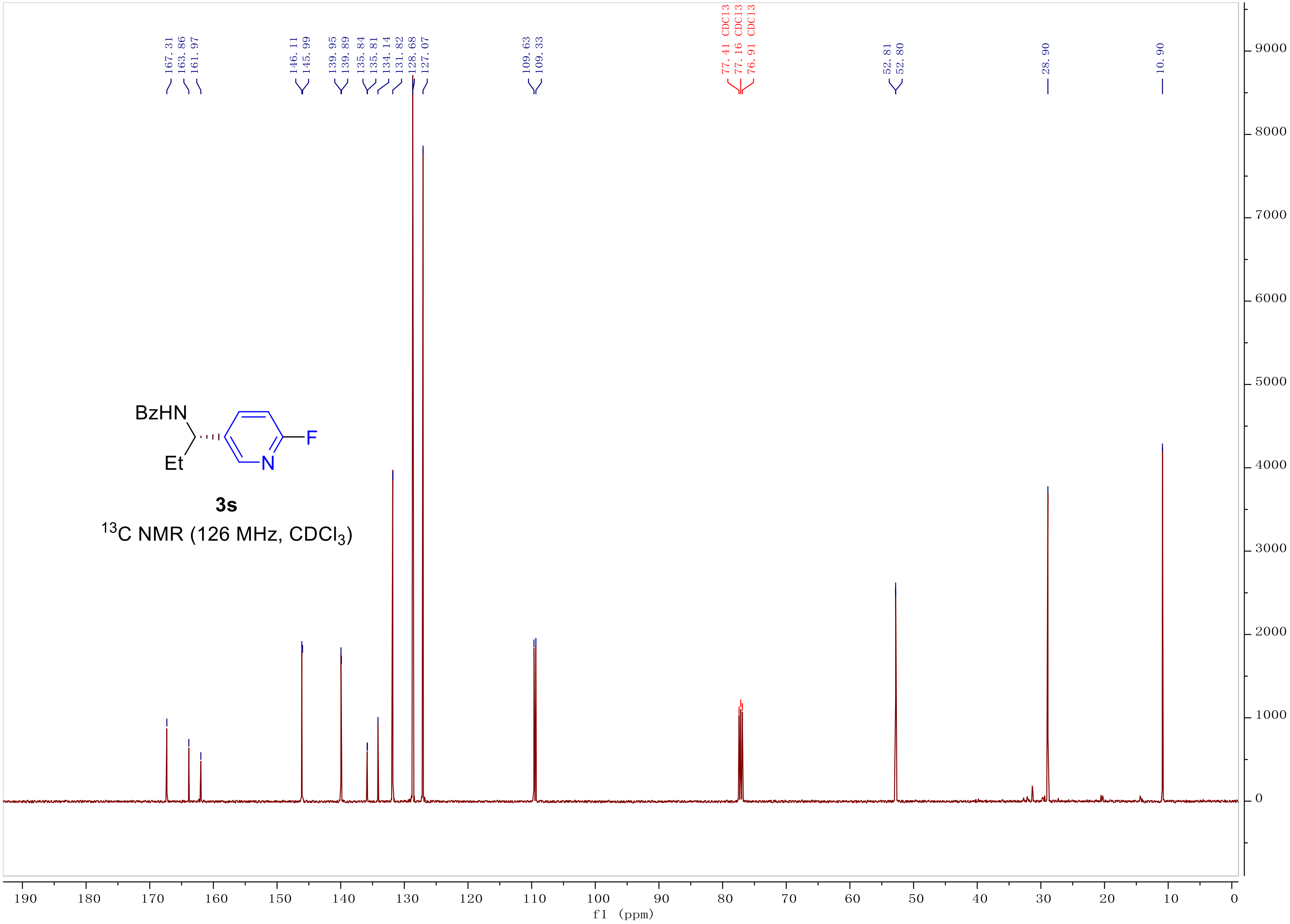

167.31
163.86
161.97
146.11
145.99
139.95
139.89
135.84
135.81
134.14
131.82
128.68
127.07
109.63
109.33
77.41 CDCl3
77.16 CDCl3
76.91 CDCl3
52.81
52.80
28.90
10.90
BzHN
F
N
Et
3s
^{13}C NMR (126 MHz, CDCl$_3$)
f1 (ppm)

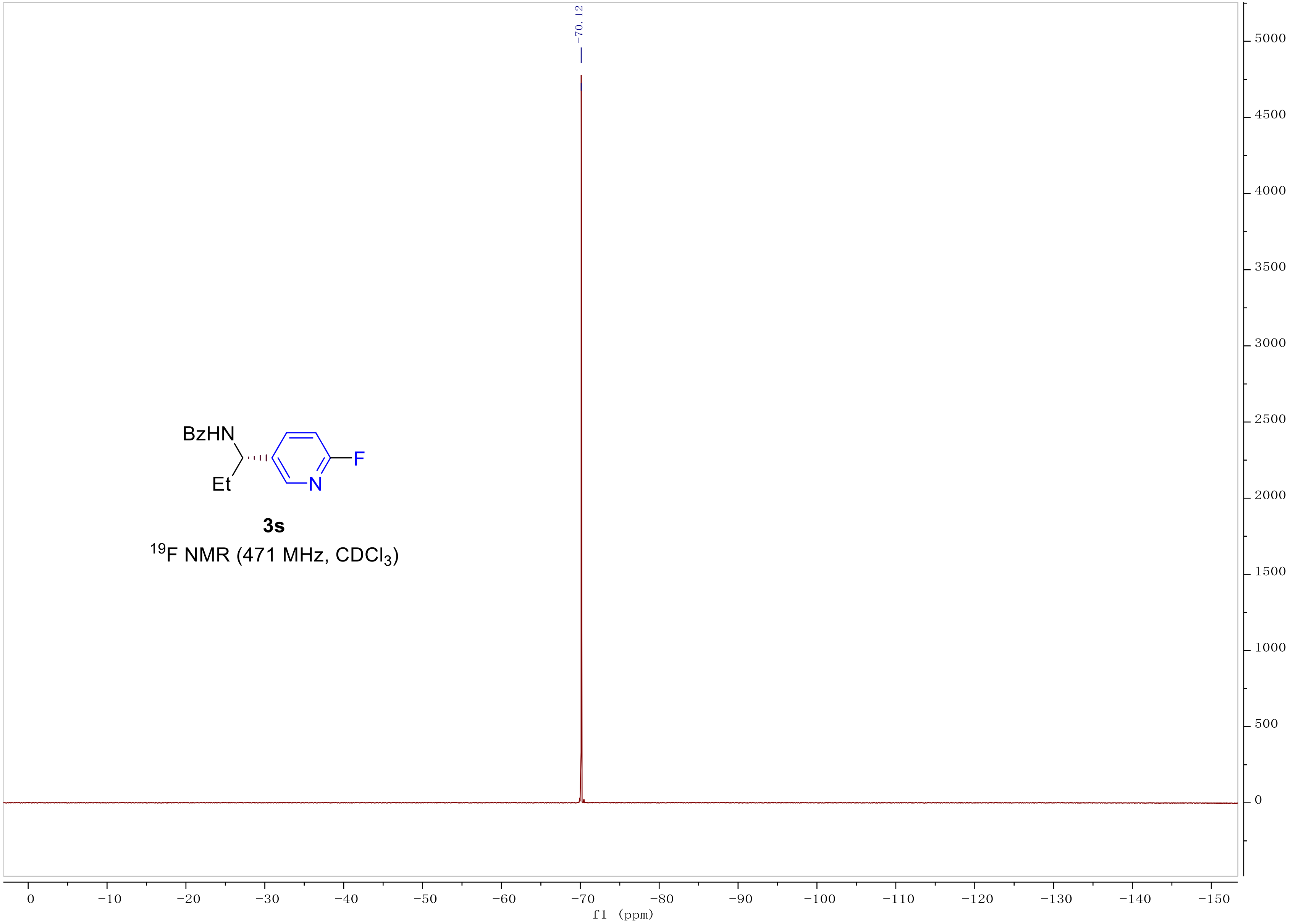
-70.12
BzHN
Et
F
N
3s
19F NMR (471 MHz, CDCl3)
5000
4500
4000
3500
3000
2500
2000
1500
1000
500
0
0 −10 −20 −30 −40 −50 −60 −70 −80 −90 −100 −110 −120 −130 −140 −150
f1 (ppm)

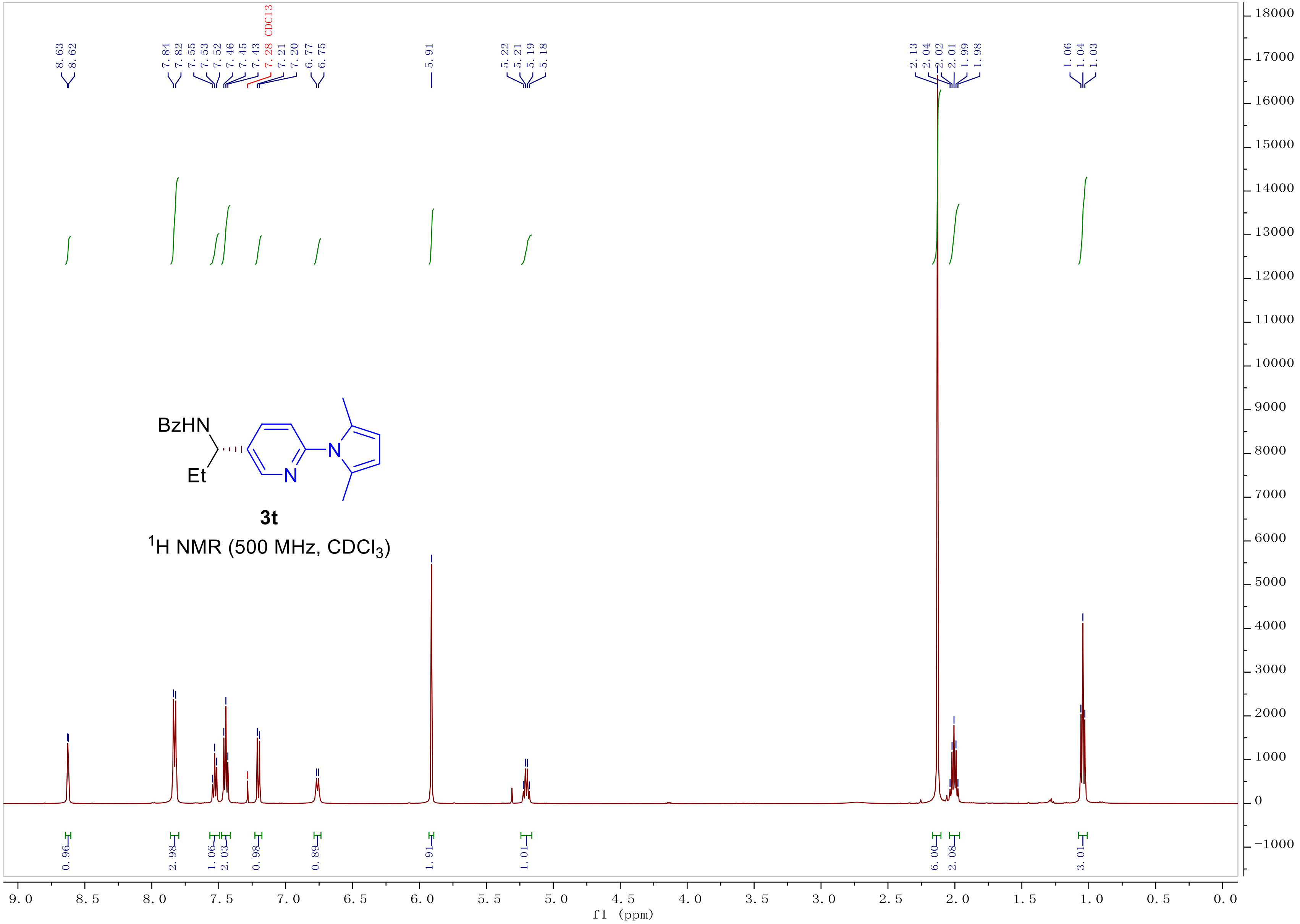
8.63
8.62
7.84
7.82
7.55
7.53
7.52
7.46
7.45
7.43
7.28 CDCl3
7.21
7.20
6.77
6.75
5.91
5.22
5.21
5.19
5.18
2.13
2.04
2.02
2.01
1.99
1.98
1.06
1.04
1.03
BzHN
Et
N
N
3t
^{1}H NMR (500 MHz, $CDCl_3$)
0.96
2.98
1.06
2.03
0.98
0.89
1.91
1.01
6.00
2.08
3.01
f1 (ppm)

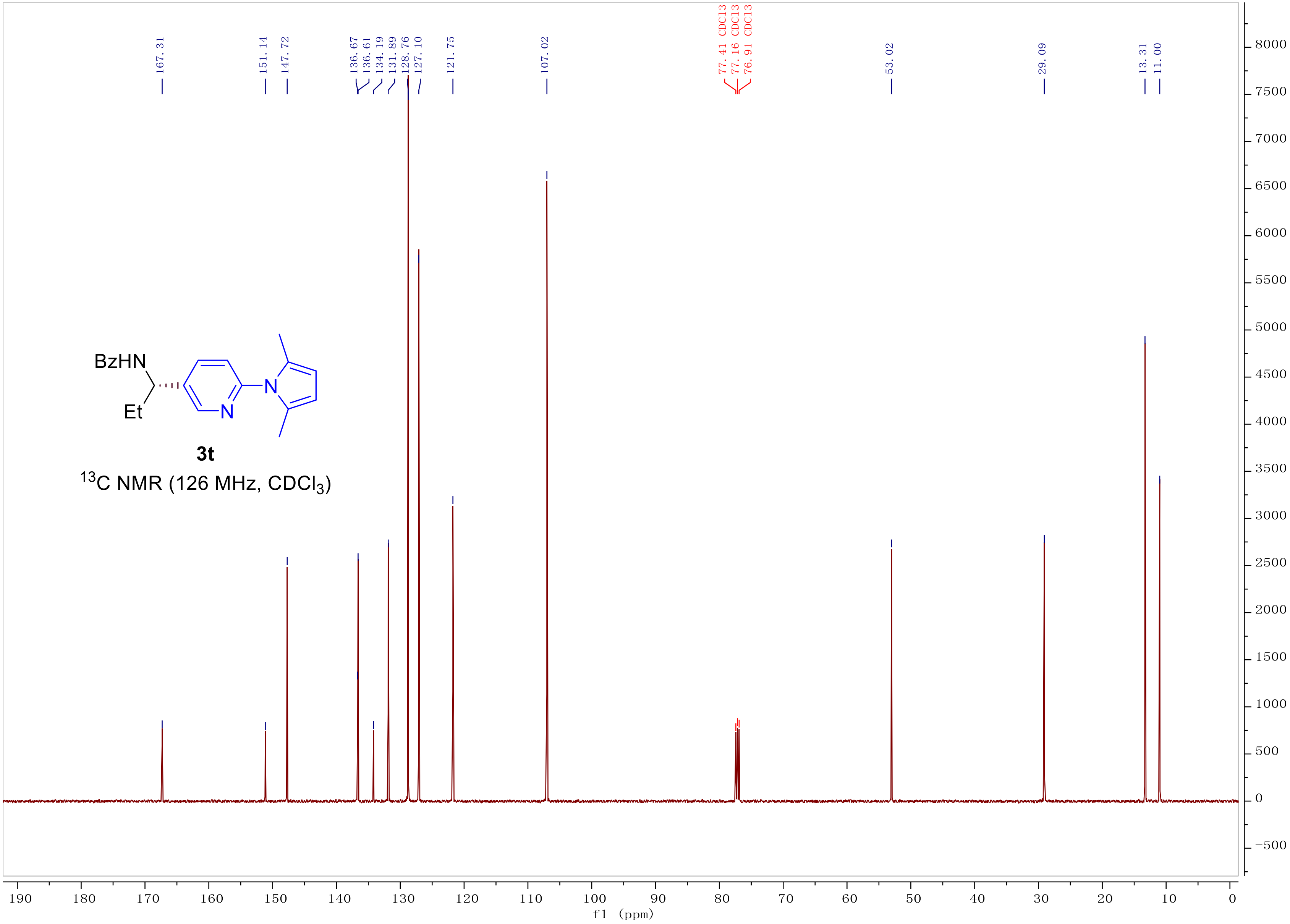

167.31
151.14
147.72
136.67
136.61
134.19
131.89
128.76
127.10
121.75
107.02
77.41 CDCl3
77.16 CDCl3
76.91 CDCl3
53.02
29.09
13.31
11.00
BzHN
Et
N
N
3t
13C NMR (126 MHz, CDCl3)
f1 (ppm)

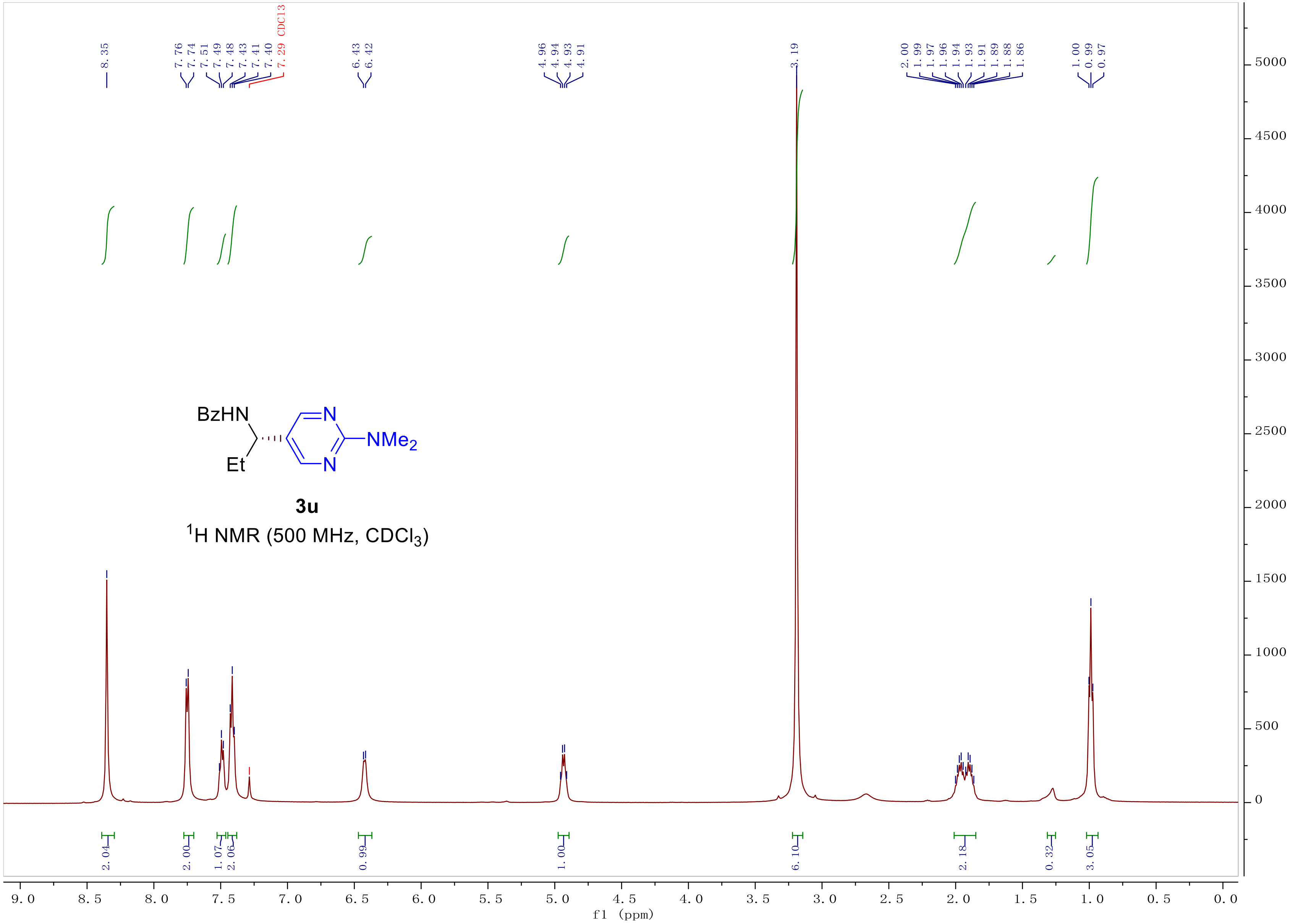
BzHN
Et
N
N
NMe2
3u
1H NMR (500 MHz, CDCl3)
8.35
7.76
7.74
7.51
7.49
7.48
7.43
7.41
7.40
7.29 CDCl3
6.43
6.42
4.96
4.94
4.93
4.91
3.19
2.00
1.99
1.97
1.96
1.94
1.93
1.91
1.89
1.88
1.86
1.00
0.99
0.97
2.04
2.00
1.07
2.06
0.99
1.00
6.10
2.18
0.32
3.05
f1 (ppm)

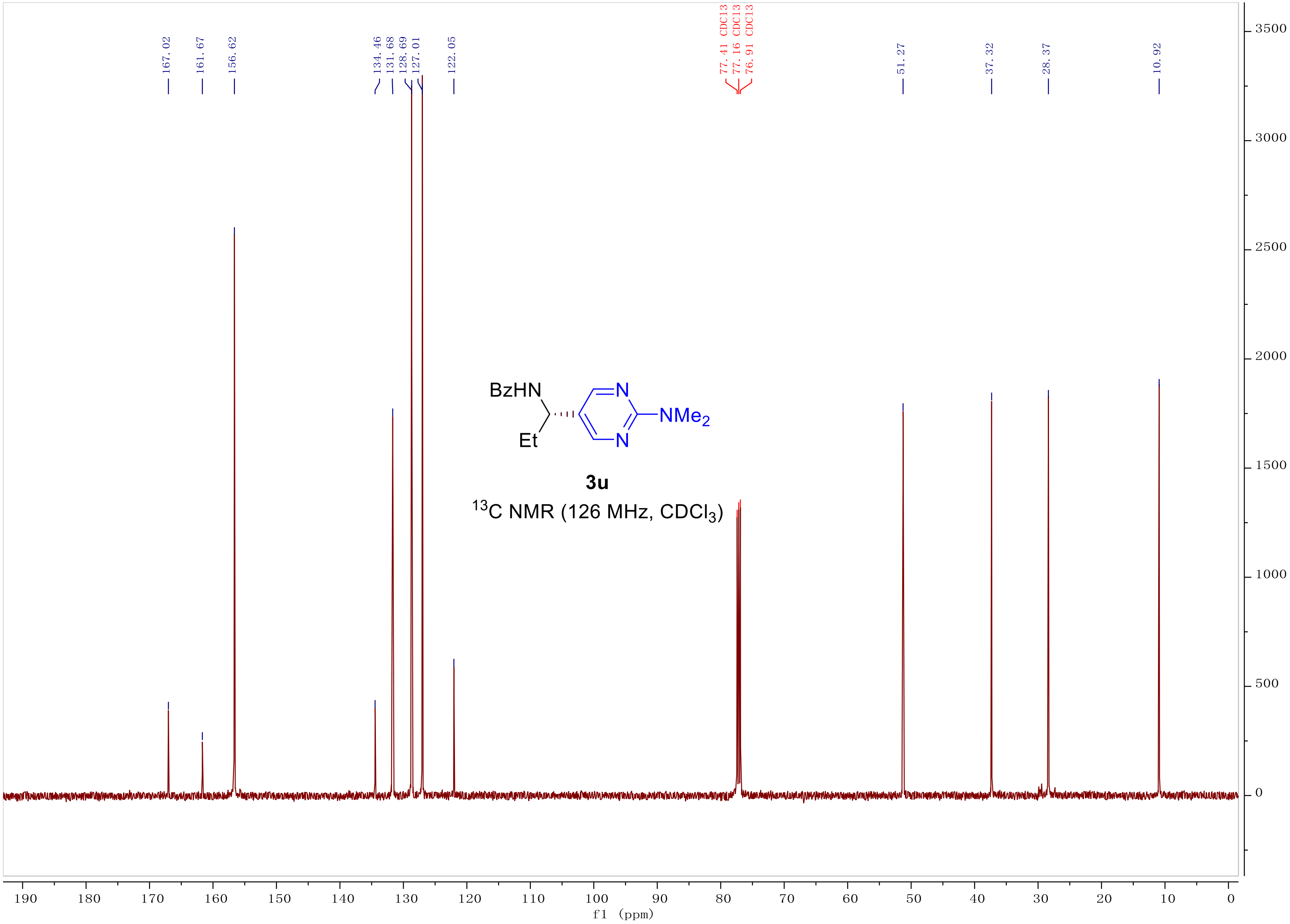

167.02
161.67
156.62
134.46
131.68
128.69
127.01
122.05
77.41 CDCl3
77.16 CDCl3
76.91 CDCl3
51.27
37.32
28.37
10.92
BzHN
Et
N
N
NMe2
3u
13C NMR (126 MHz, CDCl3)
3500
3000
2500
2000
1500
1000
500
0
190
180
170
160
150
140
130
120
110
100
90
80
70
60
50
40
30
20
10
0
f1 (ppm)

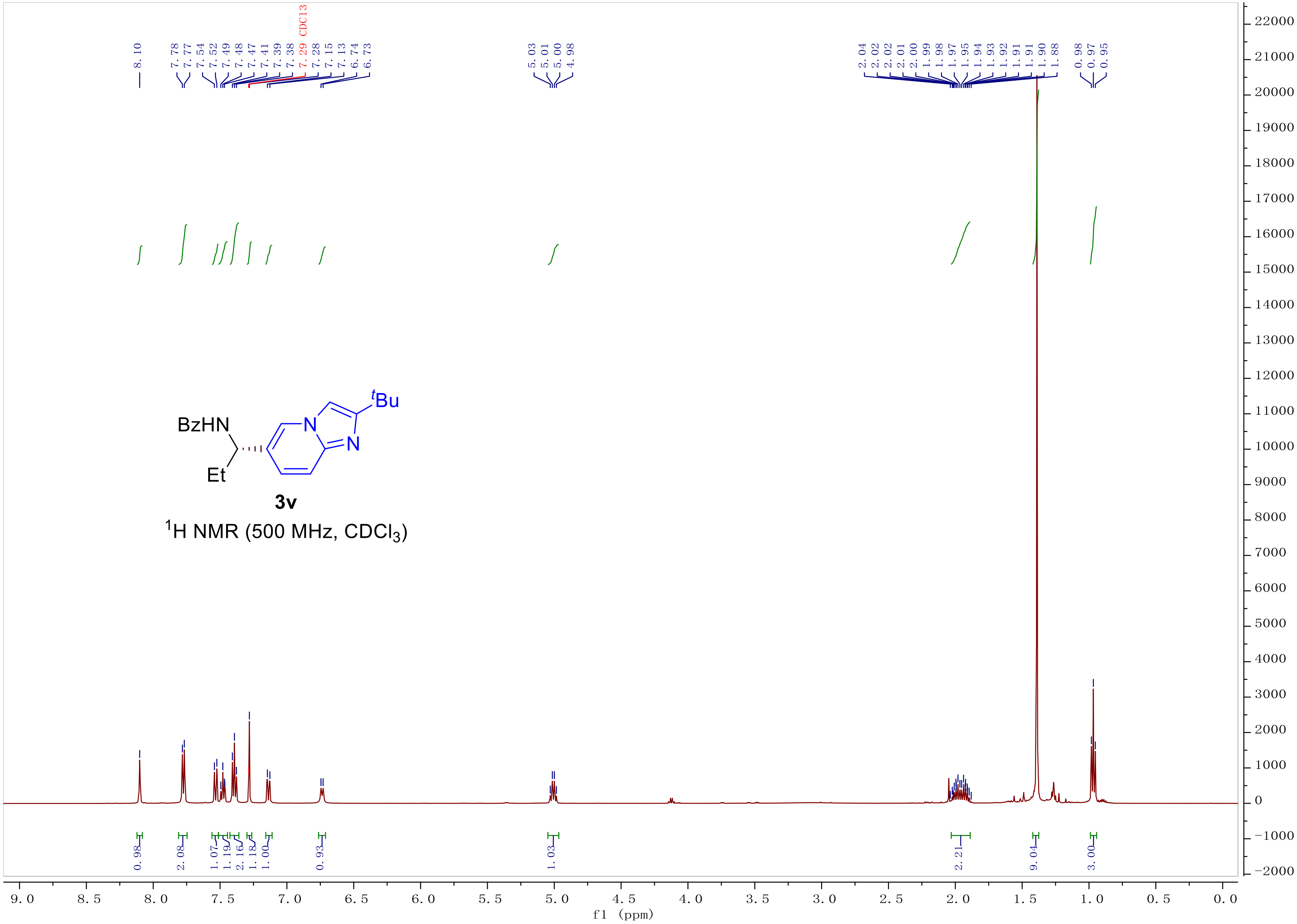

8.10
7.78
7.77
7.54
7.52
7.49
7.48
7.47
7.41
7.39
7.38
7.29 CDCl3
7.28
7.15
7.13
6.74
6.73
5.03
5.01
5.00
4.98
2.04
2.02
2.02
2.01
2.00
1.99
1.98
1.97
1.95
1.94
1.93
1.92
1.91
1.91
1.90
1.88
0.98
0.97
0.95
BzHN
Et
tBu
N
N
3v
1H NMR (500 MHz, CDCl3)
0.98
2.08
1.07
1.19
2.16
1.18
1.00
0.93
1.03
2.21
9.04
3.00
f1 (ppm)

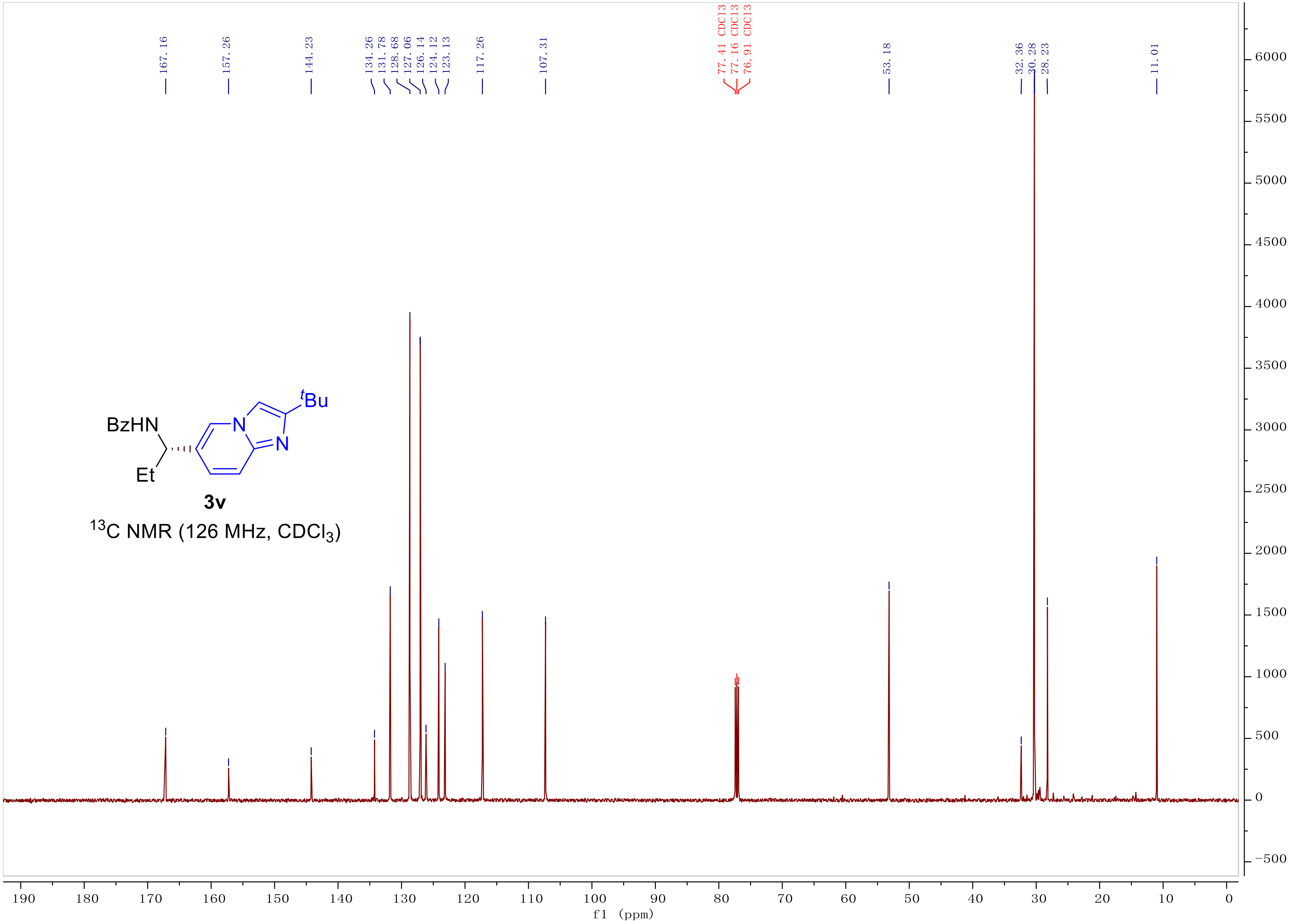

3v

^{13}C NMR (126 MHz, $CDCl_3$)

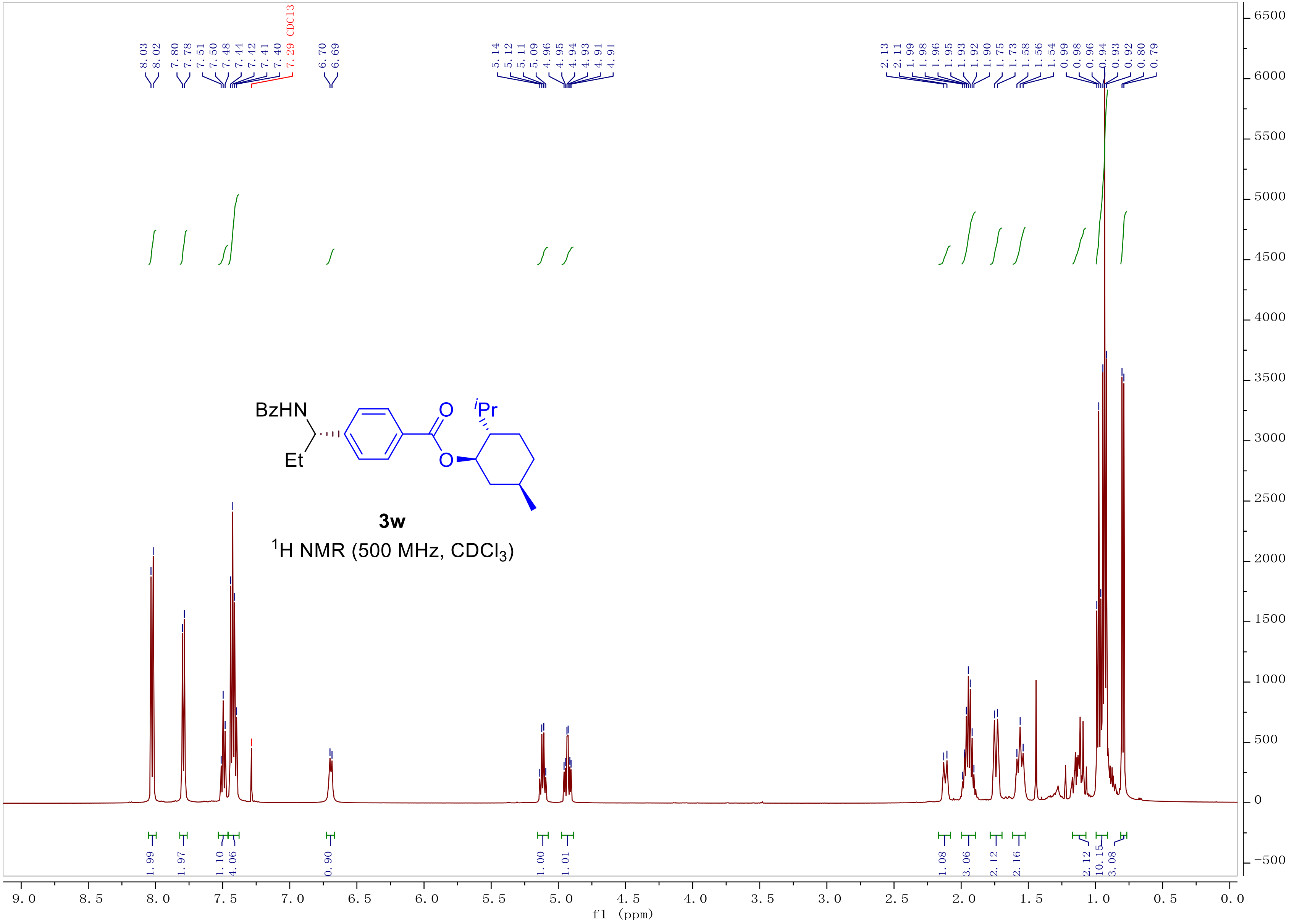

BzHN
Et
O
O
iPr
3w
1H NMR (500 MHz, CDCl3)
f1 (ppm)

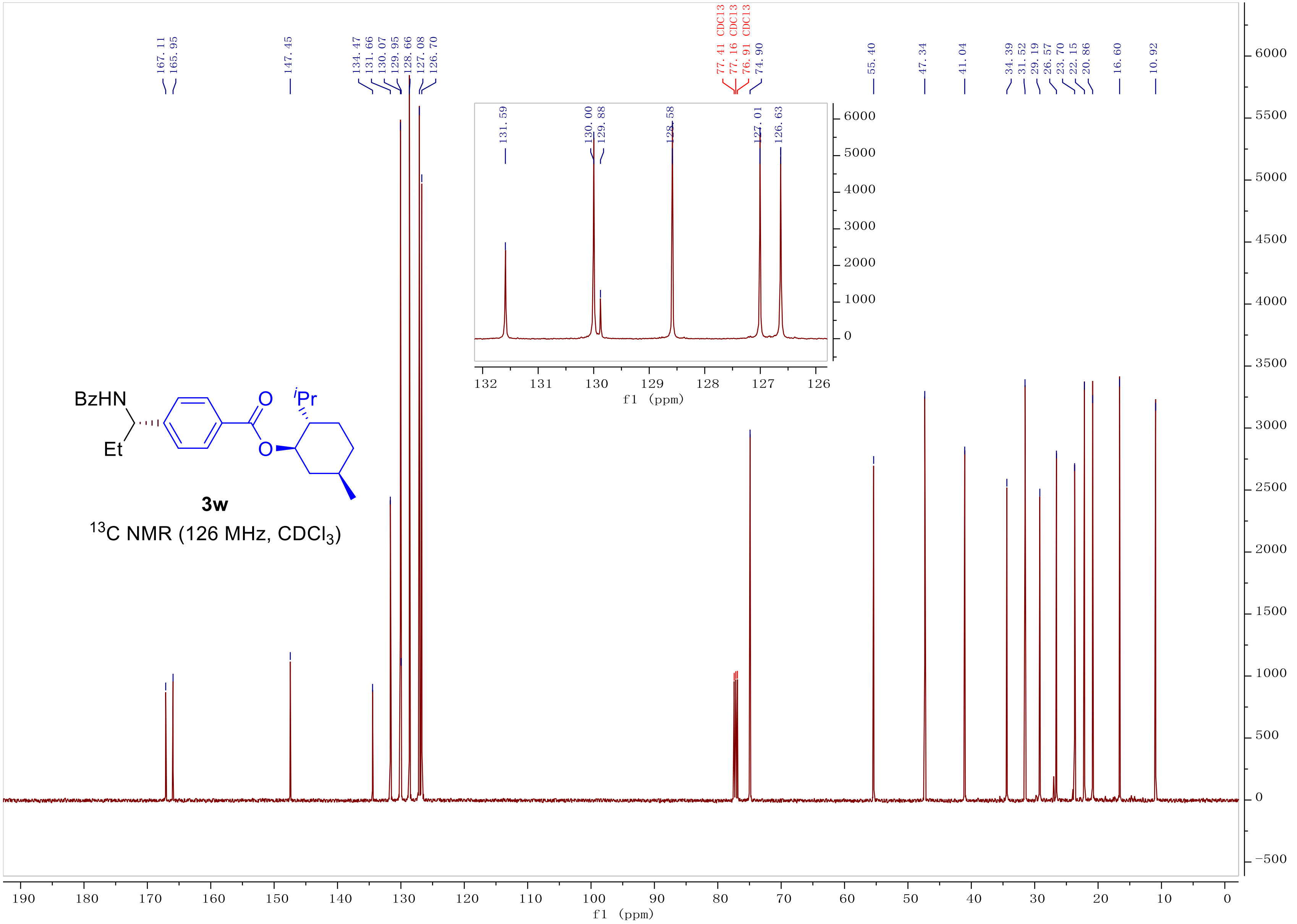
BzHN
Et
O
O
iPr
3w
13C NMR (126 MHz, CDCl3)
167.11
165.95
147.45
134.47
131.66
130.07
129.95
128.66
127.08
126.70
77.41 CDCl3
77.16 CDCl3
76.91 CDCl3
74.90
55.40
47.34
41.04
34.39
31.52
29.19
26.57
23.70
22.15
20.86
16.60
10.92
131.59
130.00
129.88
128.58
127.01
126.63
f1 (ppm)

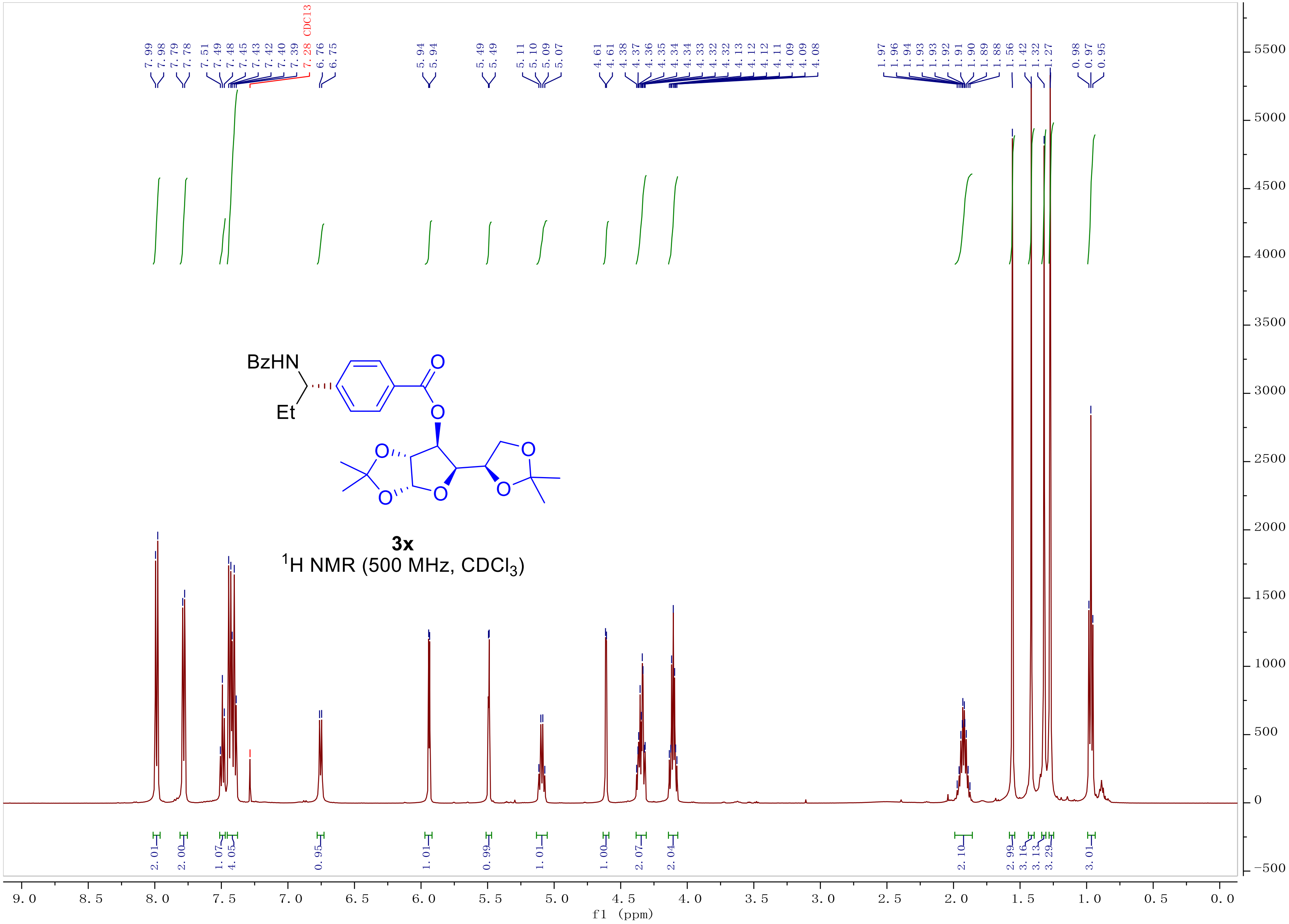
7.99 7.98 7.79 7.78 7.51 7.49 7.48 7.45 7.43 7.42 7.40 7.39 7.28 CDCl3 6.76 6.75
5.94 5.94
5.49 5.49
5.11 5.10 5.09 5.07
4.61 4.61 4.38 4.37 4.36 4.35 4.34 4.34 4.33 4.32 4.32 4.13 4.12 4.12 4.11 4.09 4.09 4.08
1.97 1.96 1.94 1.93 1.93 1.92 1.91 1.90 1.89 1.88 1.56 1.42 1.32 1.27
0.98 0.97 0.95
BzHN
Et
O
3x
^{1}H NMR (500 MHz, CDCl$_3$)
2.01 2.00 1.07 4.05 0.95 1.01 0.99 1.01 1.00 2.07 2.04 2.10 2.99 3.16 3.13 3.29 3.01
f1 (ppm)

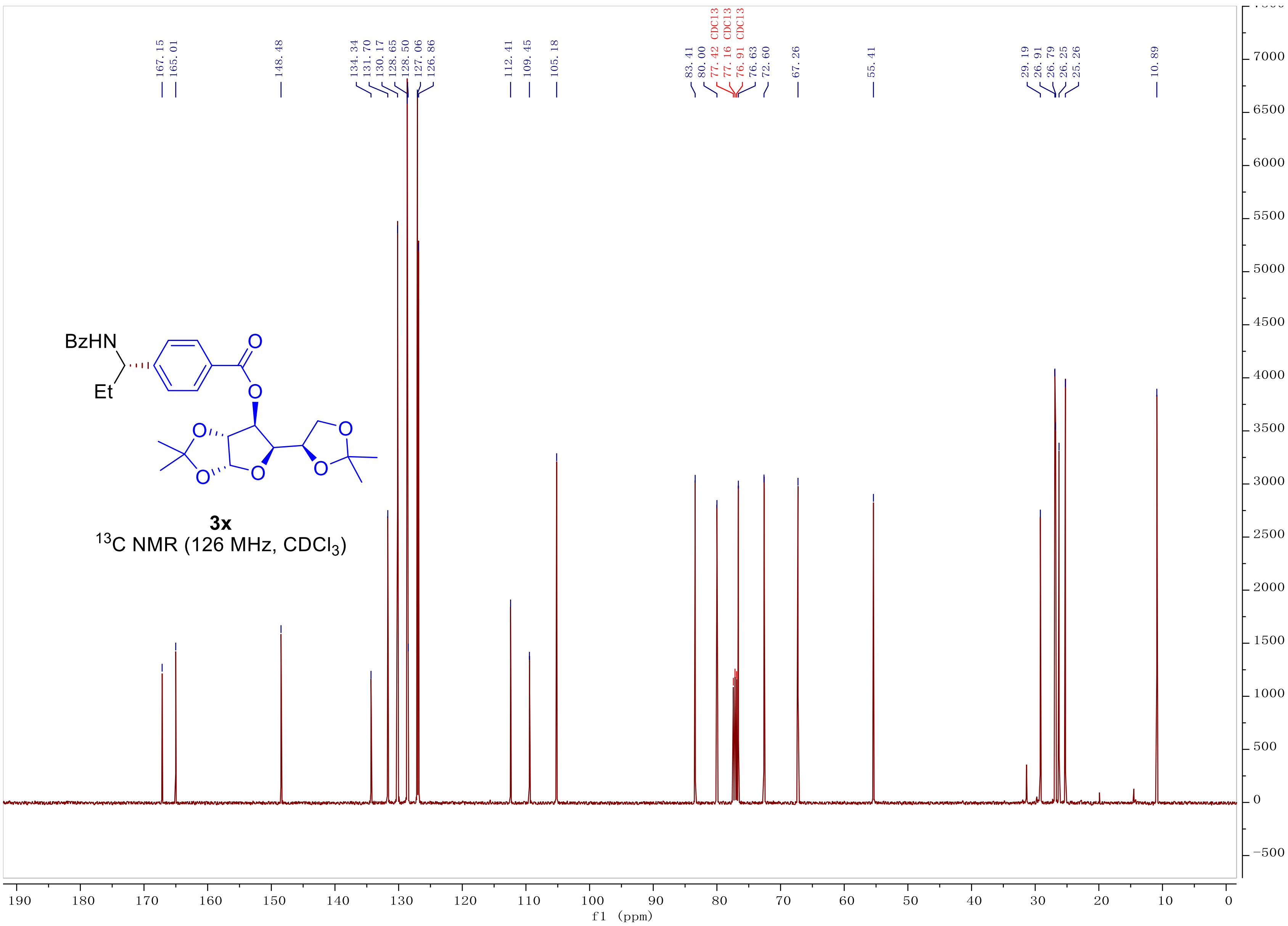

3x

^{13}C NMR (126 MHz, $CDCl_3$)

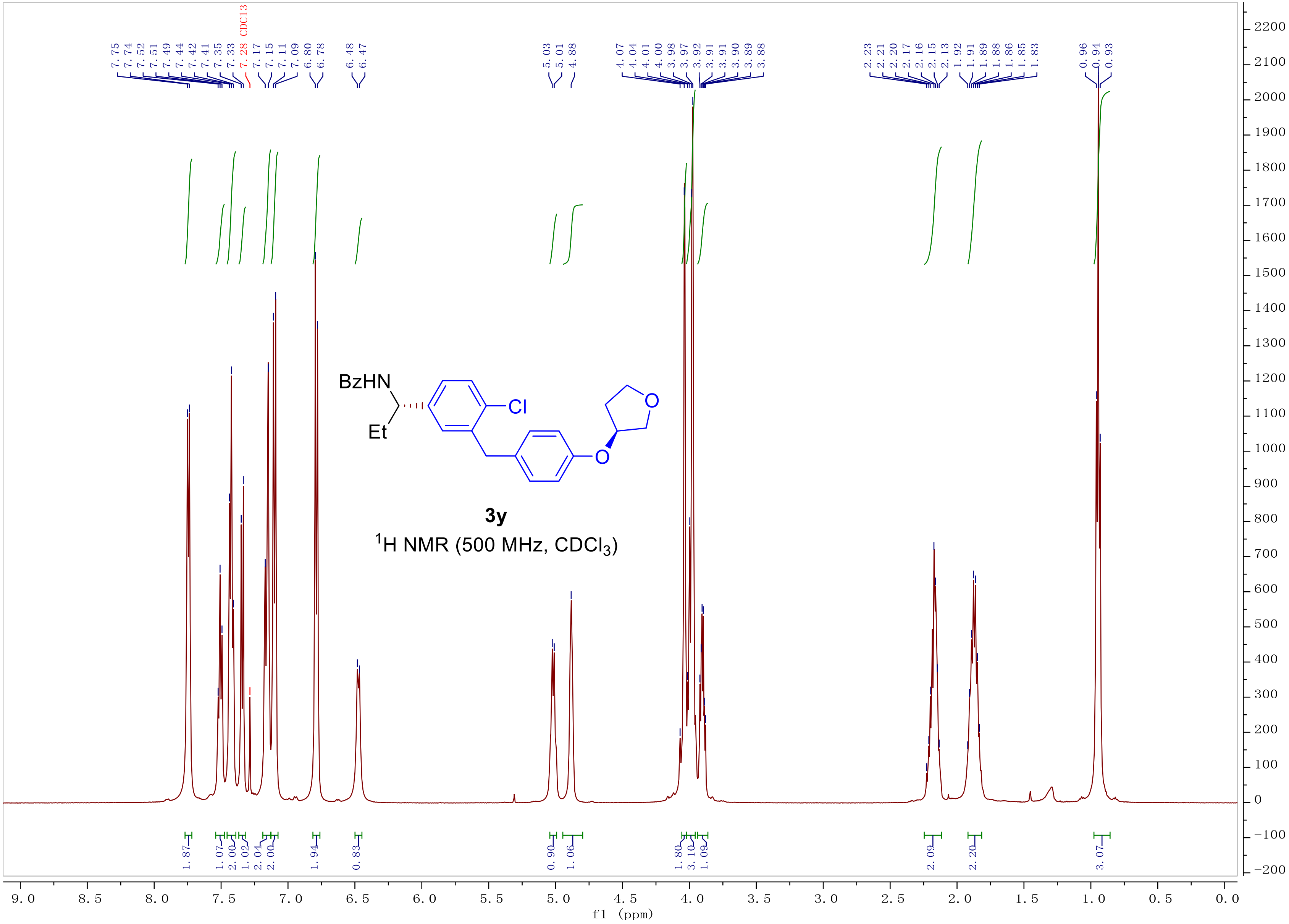
BzHN
Et
Cl
O
O
3y
1H NMR (500 MHz, CDCl3)
f1 (ppm)

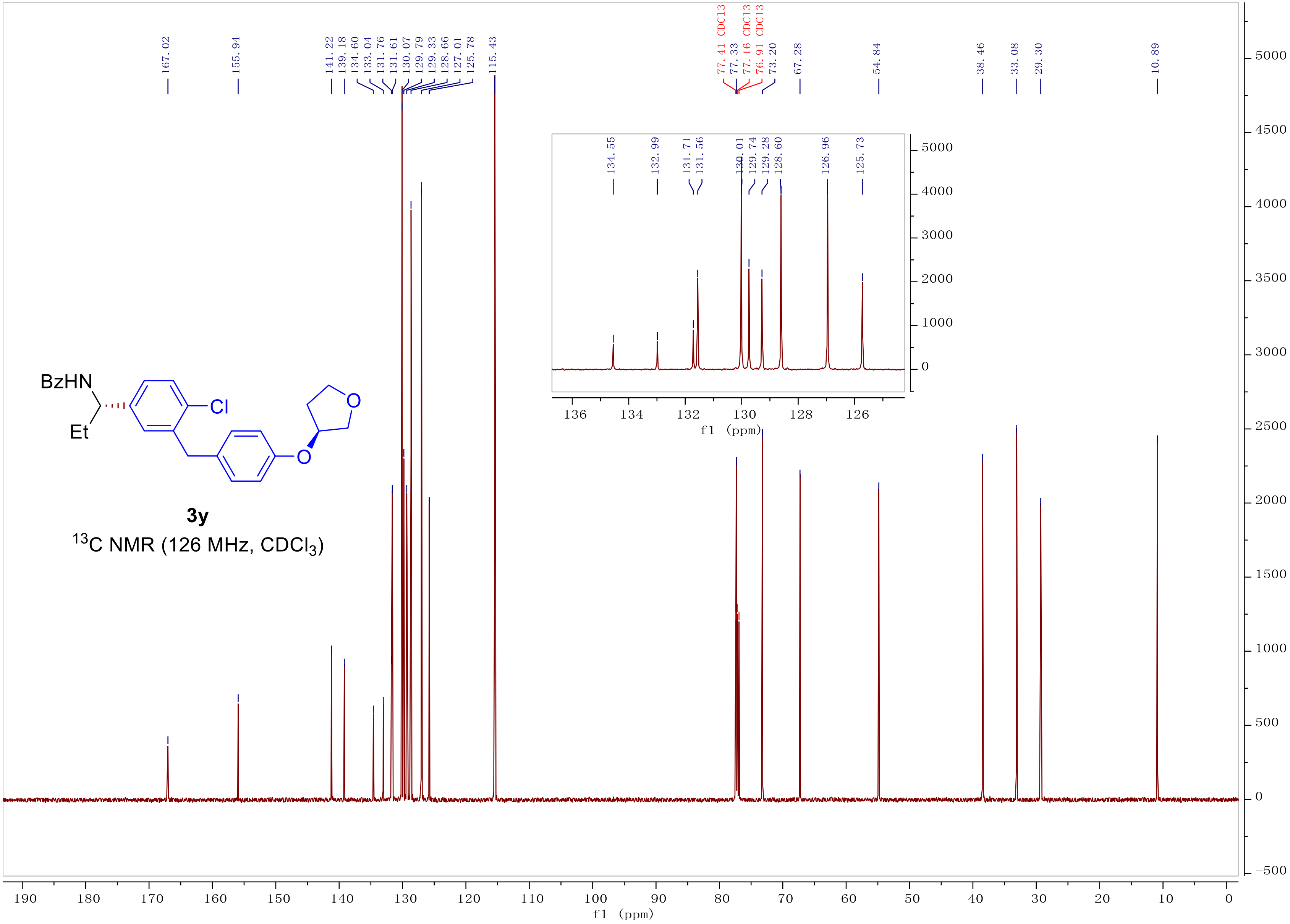

3y

^{13}C NMR (126 MHz, $CDCl_3$)

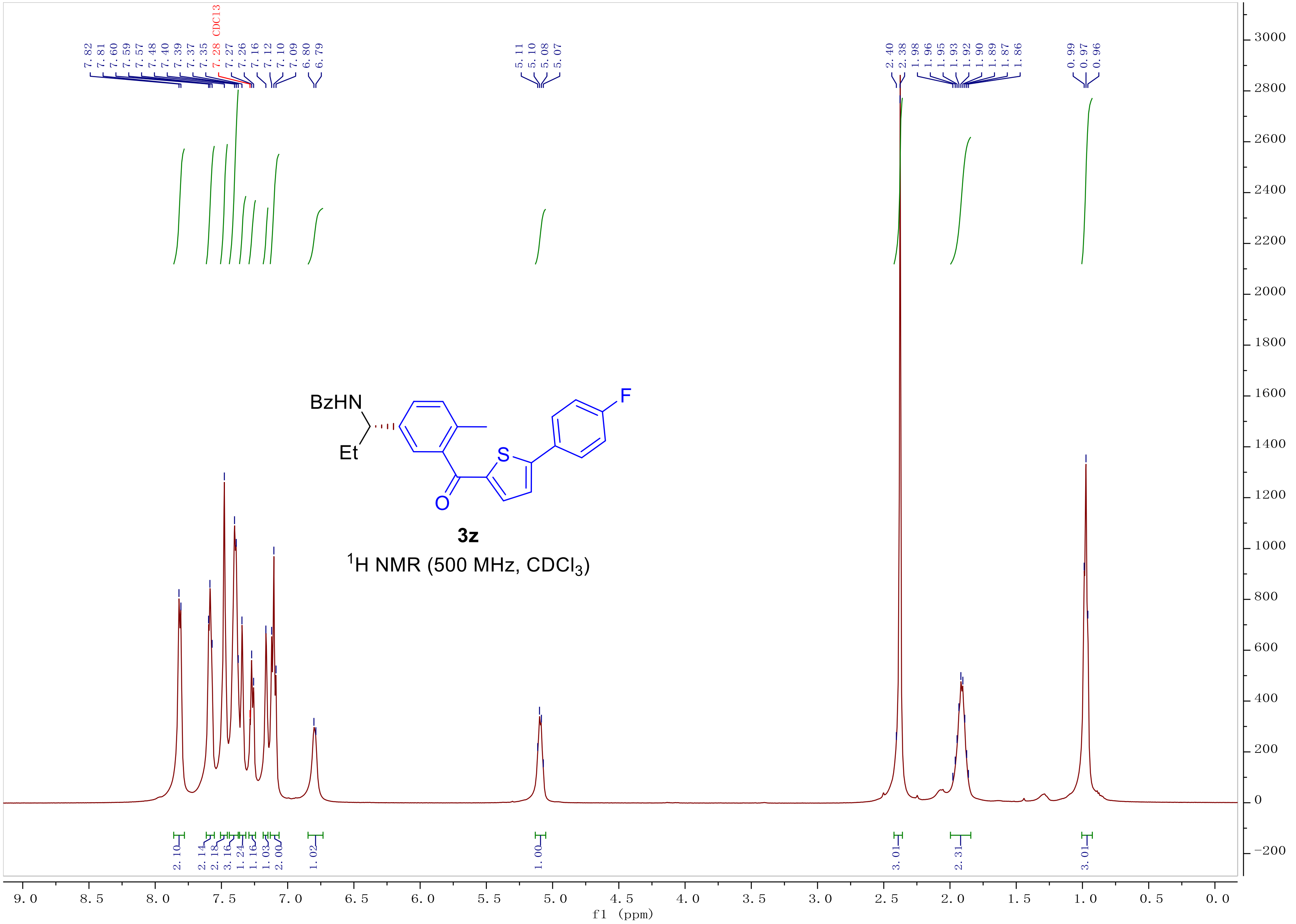
7.82
7.81
7.60
7.59
7.57
7.48
7.40
7.39
7.37
7.35
7.28 CDCl3
7.27
7.26
7.16
7.12
7.10
7.09
6.80
6.79
5.11
5.10
5.08
5.07
2.40
2.38
1.98
1.96
1.95
1.93
1.92
1.90
1.89
1.87
1.86
0.99
0.97
0.96
BzHN
Et
S
F
O
3z
^{1}H NMR (500 MHz, $CDCl_3$)
2.10
2.14
2.18
3.16
1.24
1.16
1.03
2.00
1.02
1.00
3.01
2.31
3.01
f1 (ppm)

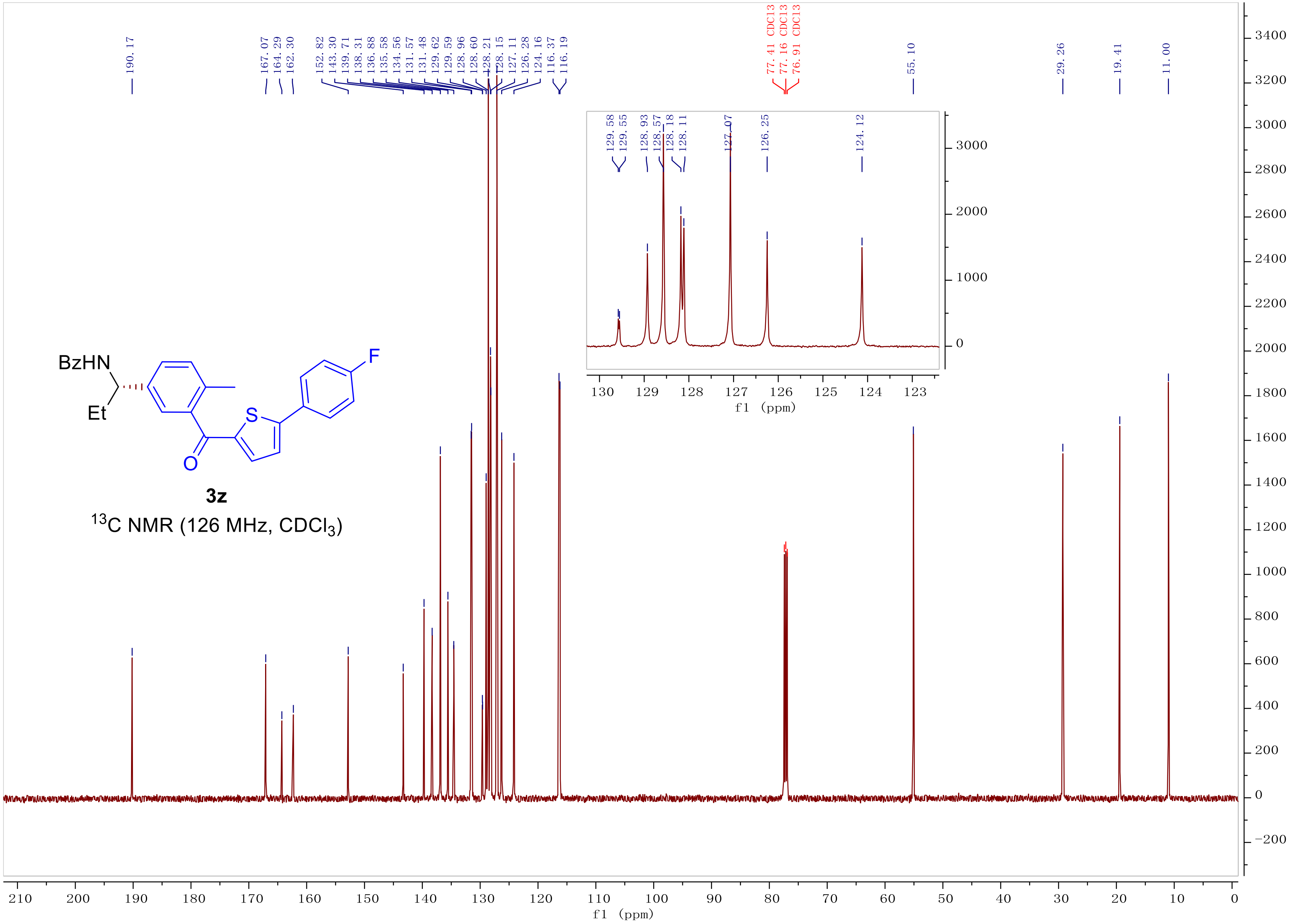

3z

^{13}C NMR (126 MHz, $CDCl_3$)

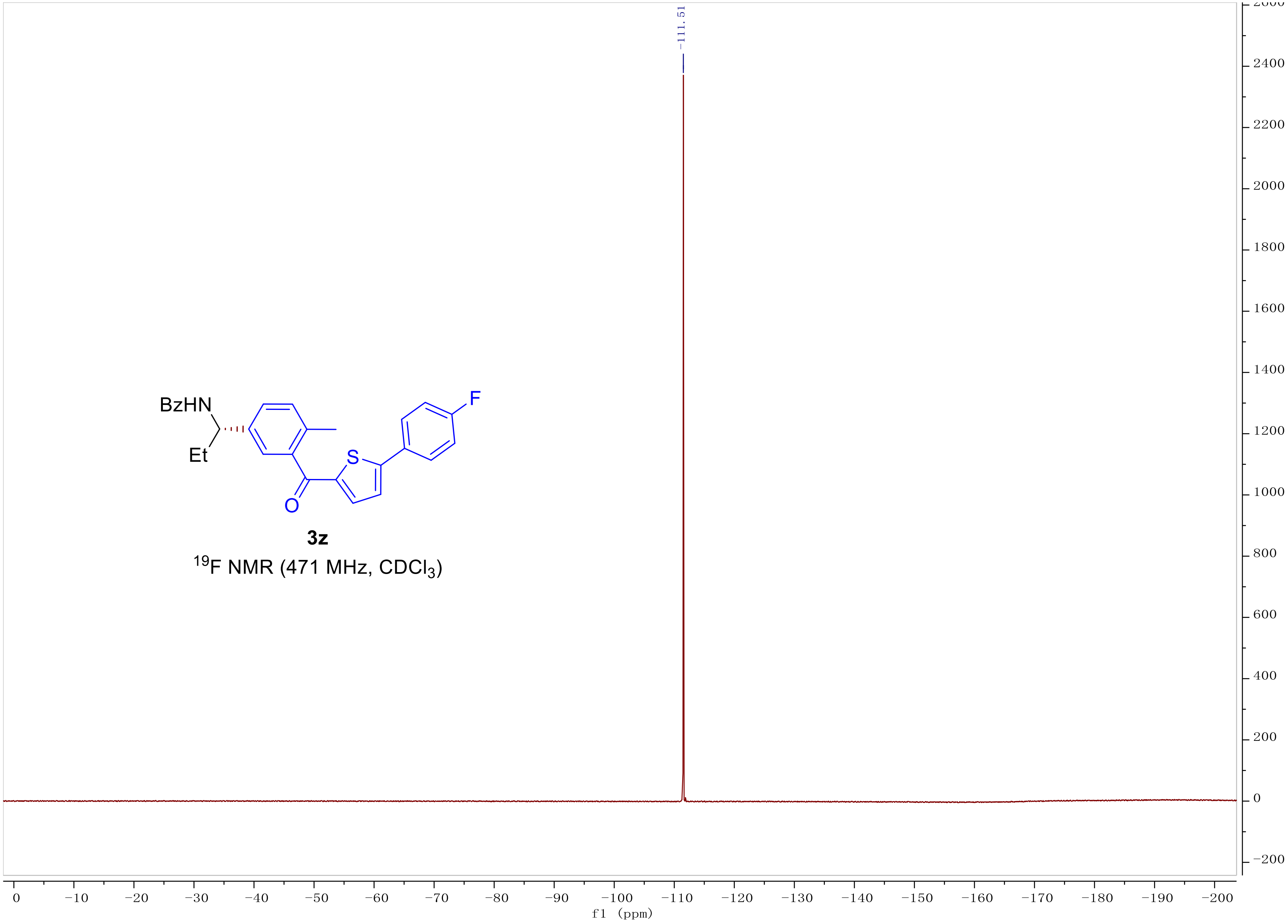

3z

^{19}F NMR (471 MHz, $CDCl_3$)

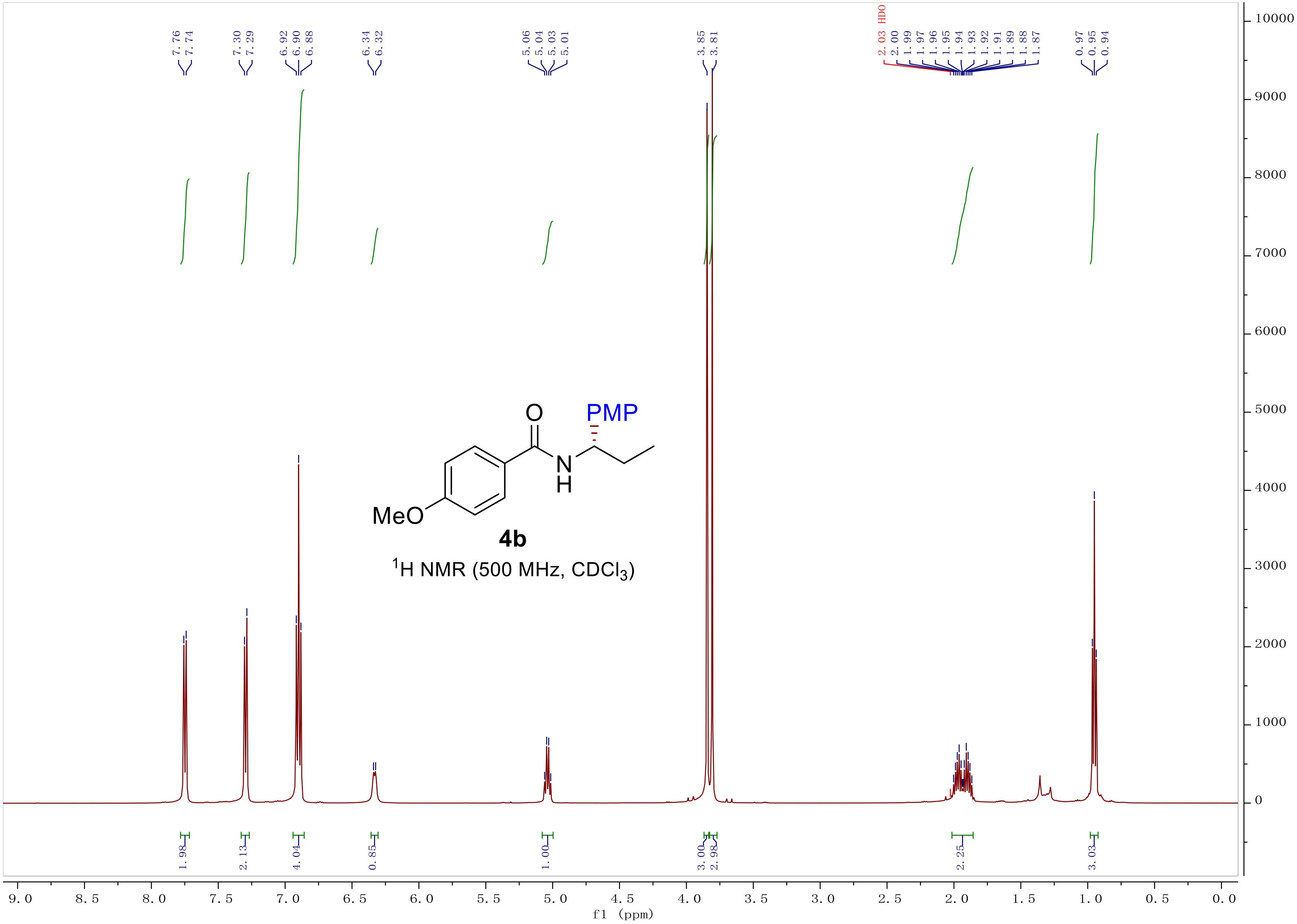

7.76
7.74
7.30
7.29
6.92
6.90
6.88
6.34
6.32
5.06
5.04
5.03
5.01
3.85
3.81
2.03 HDO
2.00
1.99
1.97
1.96
1.95
1.94
1.93
1.92
1.91
1.89
1.88
1.87
0.97
0.95
0.94
O
PMP
N
H
MeO
4b
1H NMR (500 MHz, CDCl3)
1.98
2.13
4.04
0.85
1.00
3.00
2.98
2.25
3.03
f1 (ppm)

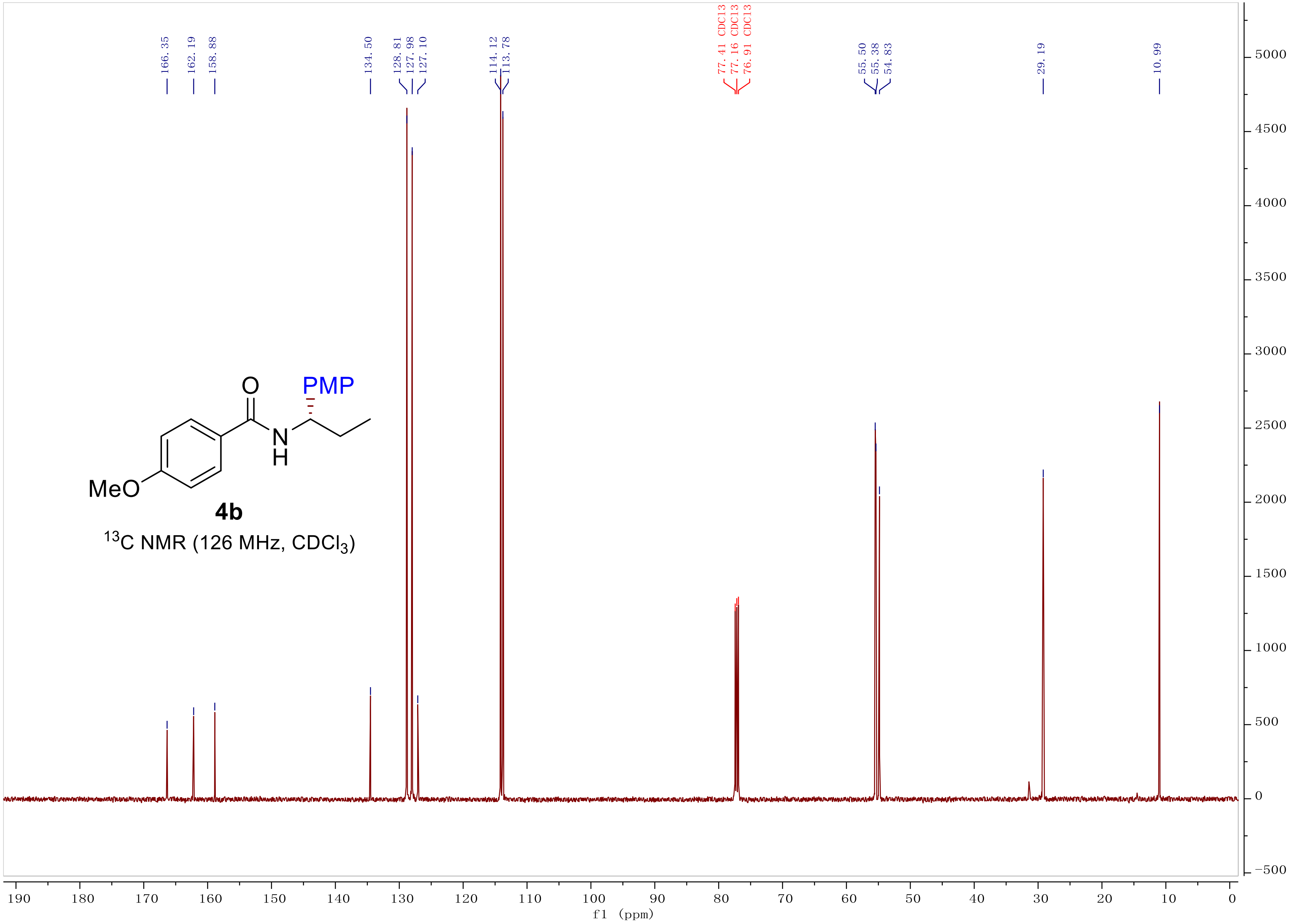

166.35
162.19
158.88
134.50
128.81
127.98
127.10
114.12
113.78
77.41 CDCl3
77.16 CDCl3
76.91 CDCl3
55.50
55.38
54.83
29.19
10.99
O
PMP
N
H
MeO
4b
^{13}C NMR (126 MHz, CDCl$_3$)
f1 (ppm)

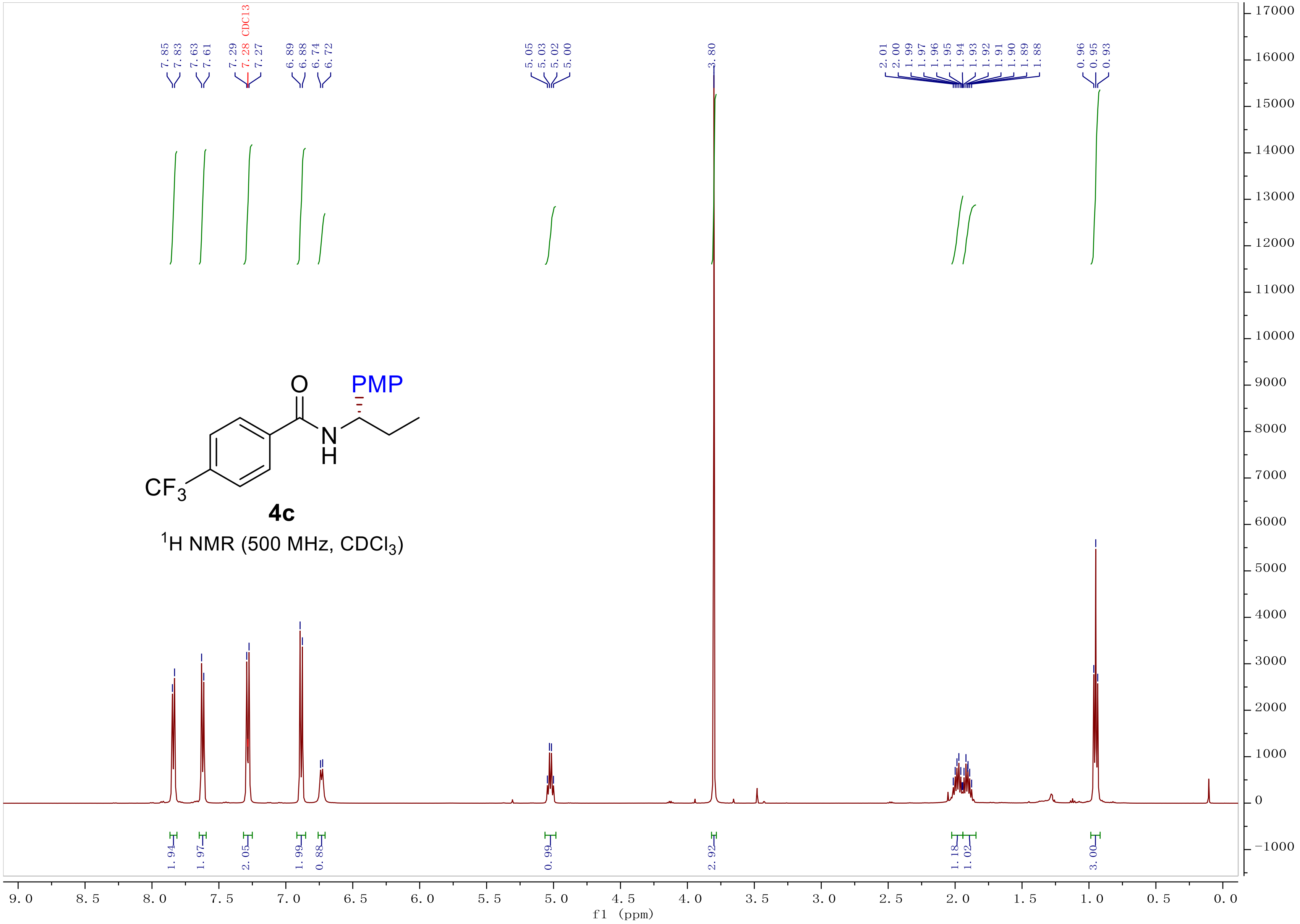

7.85
7.83
7.63
7.61
7.29
7.28 CDCl3
7.27
6.89
6.88
6.74
6.72
5.05
5.03
5.02
5.00
3.80
2.01
2.00
1.99
1.97
1.96
1.95
1.94
1.93
1.92
1.91
1.90
1.89
1.88
0.96
0.95
0.93
PMP
O
N
H
CF3
4c
1H NMR (500 MHz, CDCl3)
1.94
1.97
2.05
1.99
0.88
0.99
2.92
1.18
1.02
3.00
f1 (ppm)

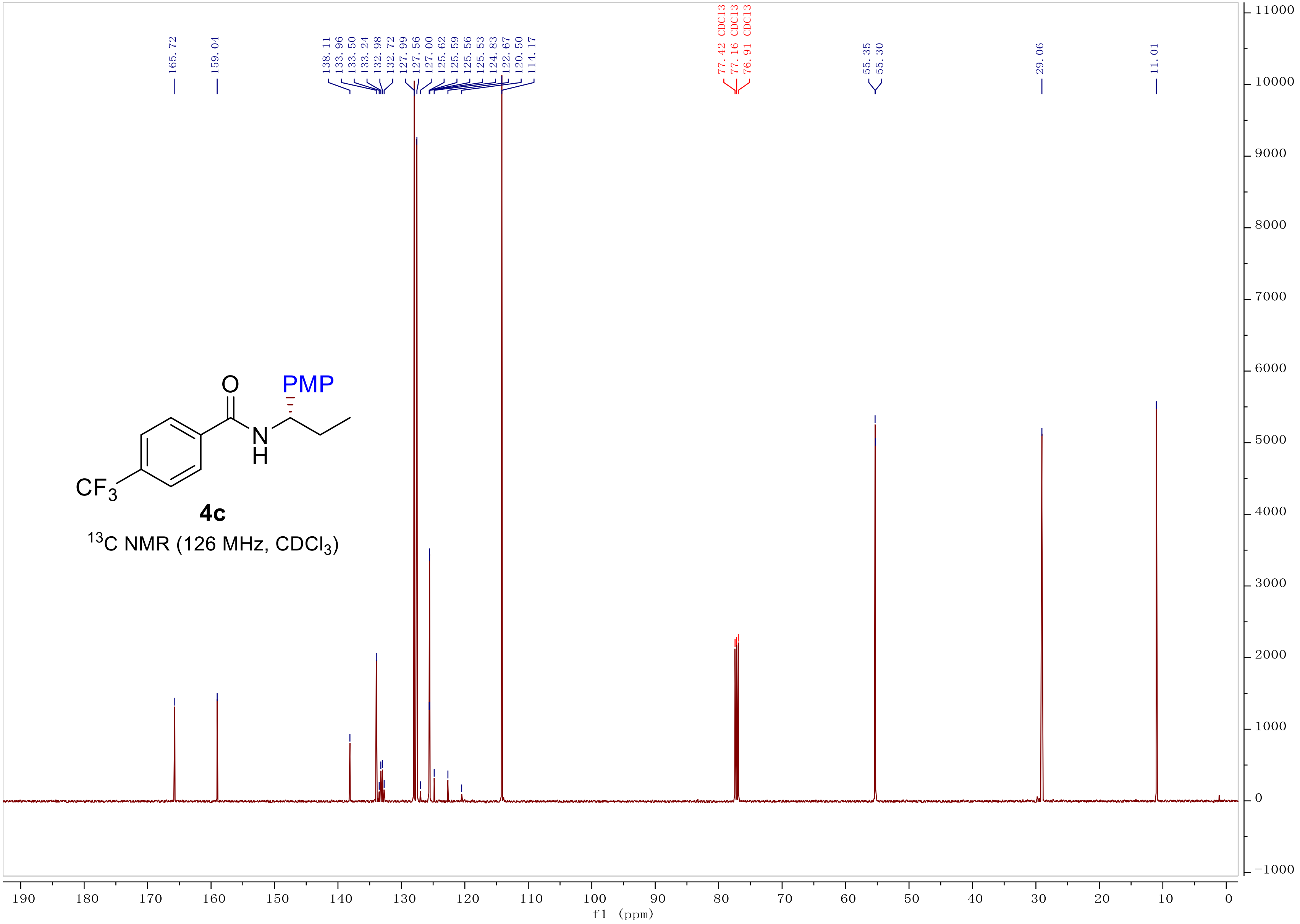
165.72
159.04
138.11
133.96
133.50
133.24
132.98
132.72
127.99
127.56
127.00
125.62
125.59
125.56
125.53
124.83
122.67
120.50
114.17
77.42 CDCl3
77.16 CDCl3
76.91 CDCl3
55.35
55.30
29.06
11.01
O
PMP
N
H
CF3
4c
13C NMR (126 MHz, CDCl3)
f1 (ppm)

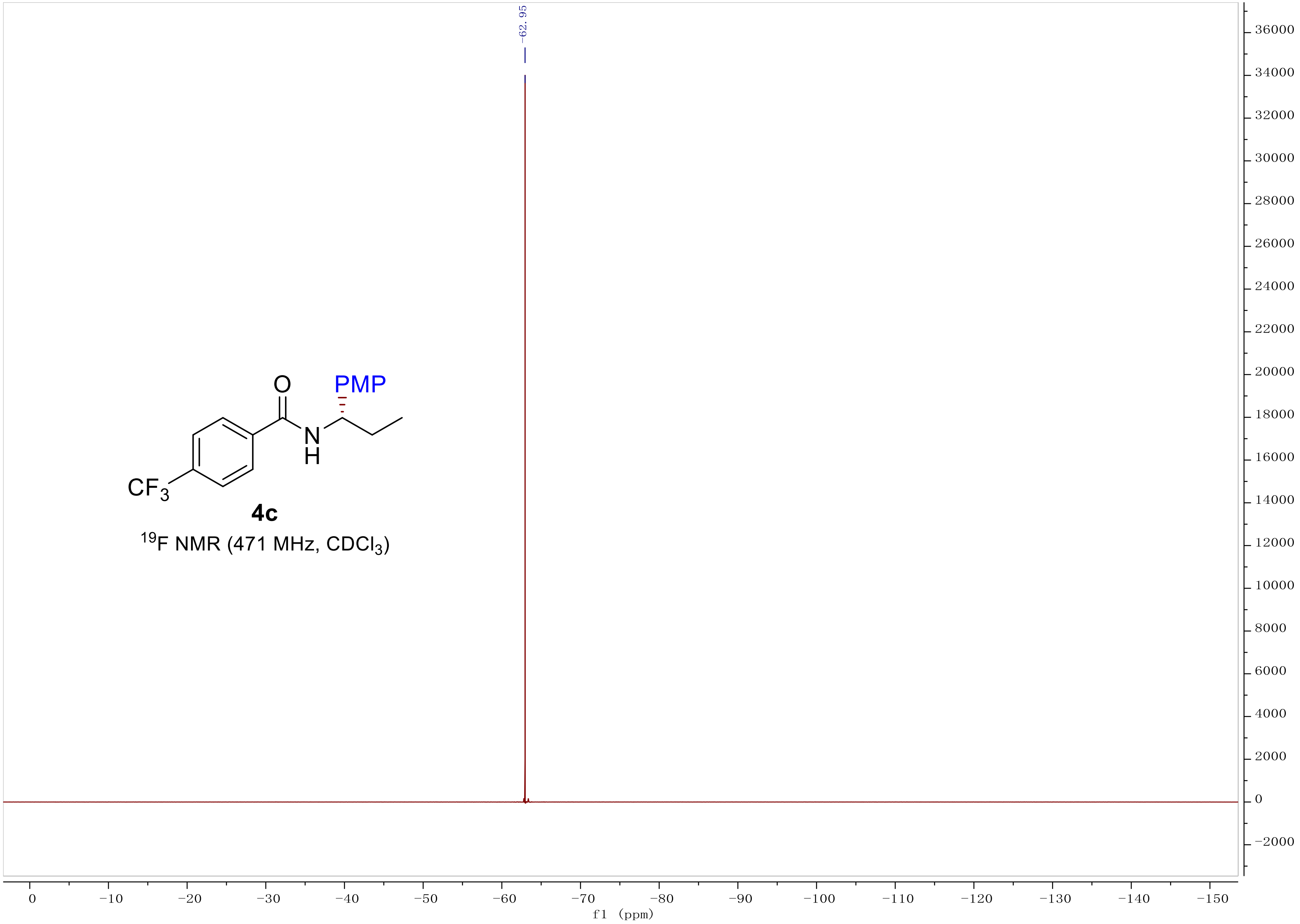

4c

^{19}F NMR (471 MHz, $CDCl_3$)

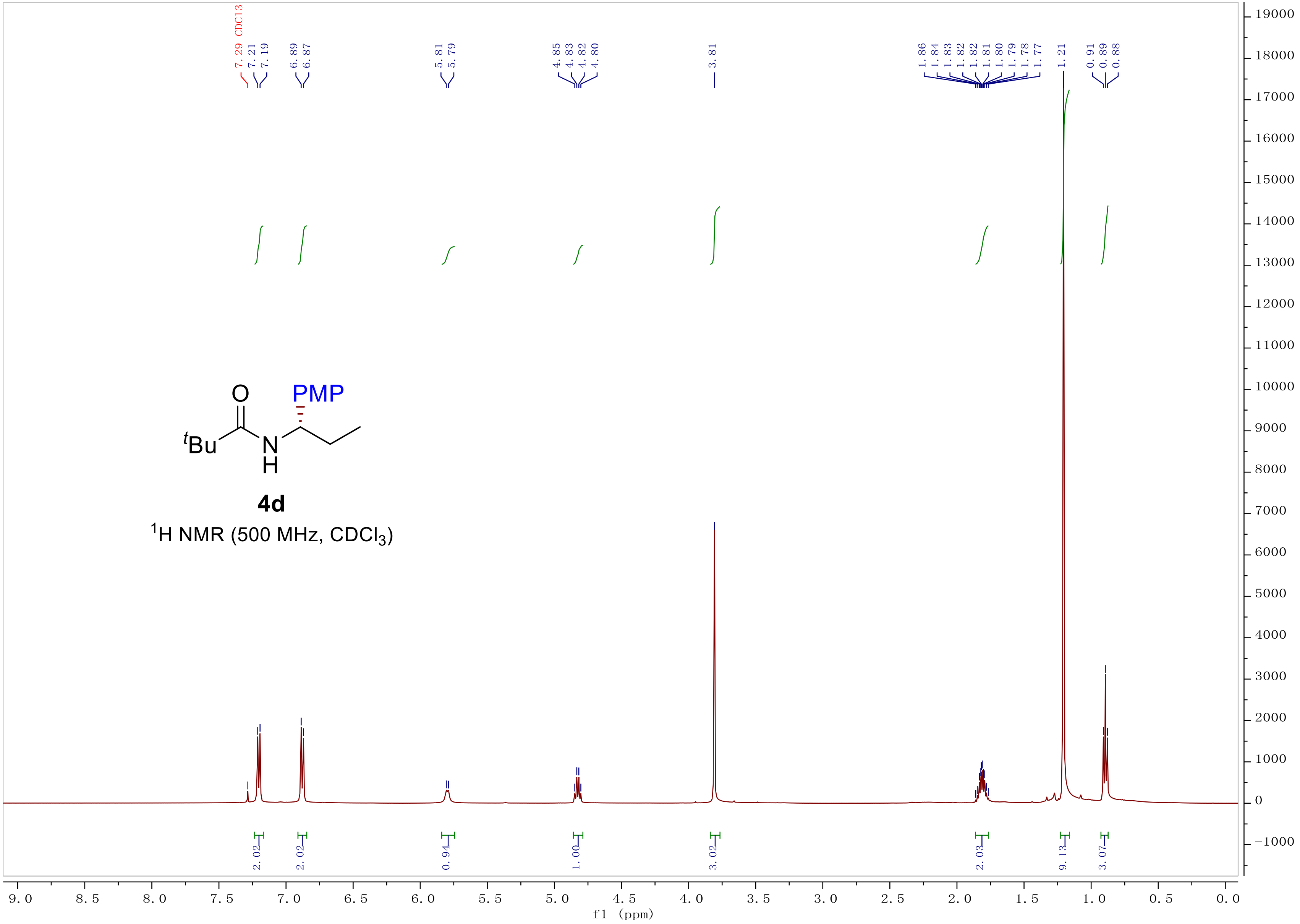

4d

^{1}H NMR (500 MHz, $CDCl_3$)

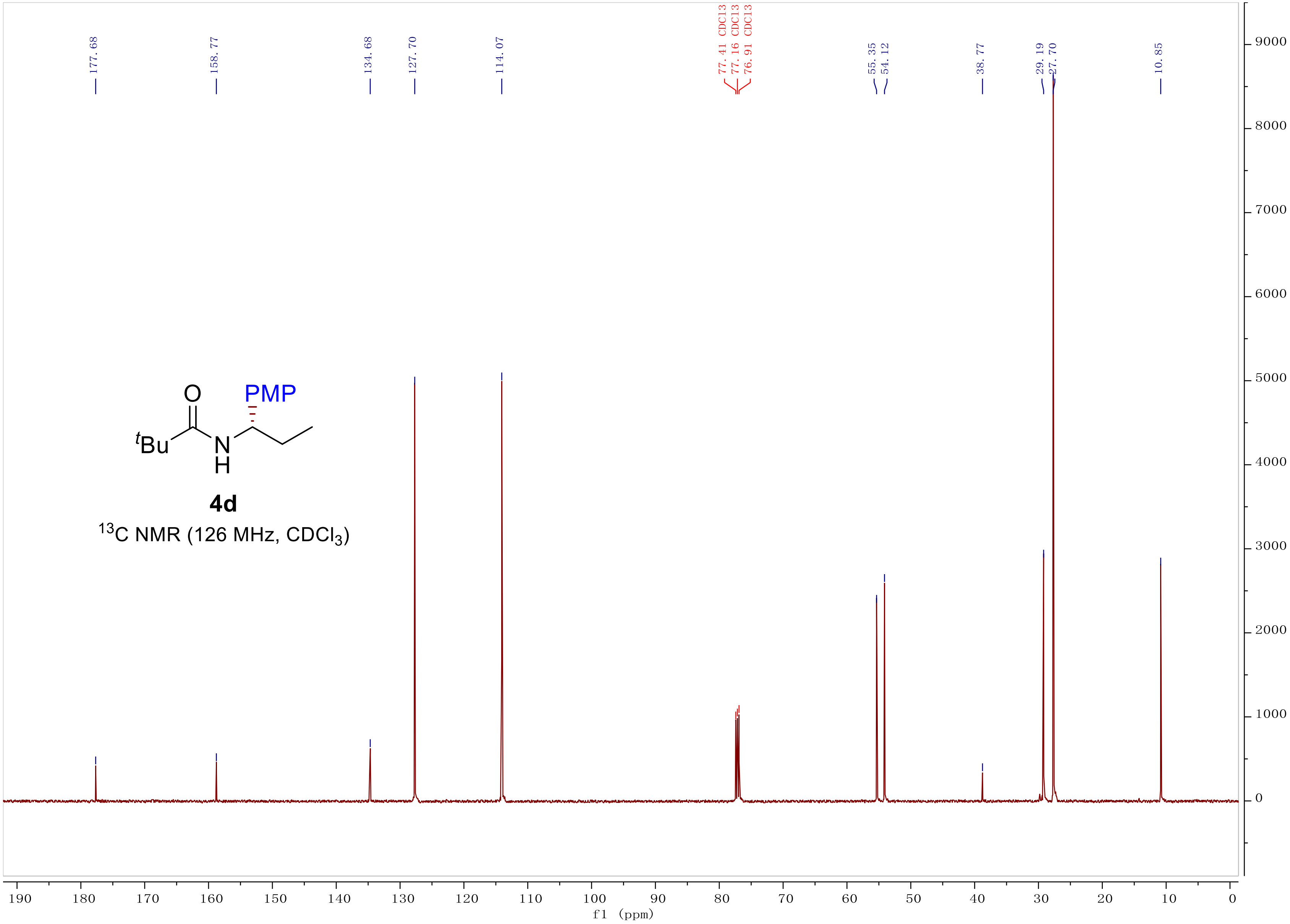

4d

^{13}C NMR (126 MHz, $CDCl_3$)

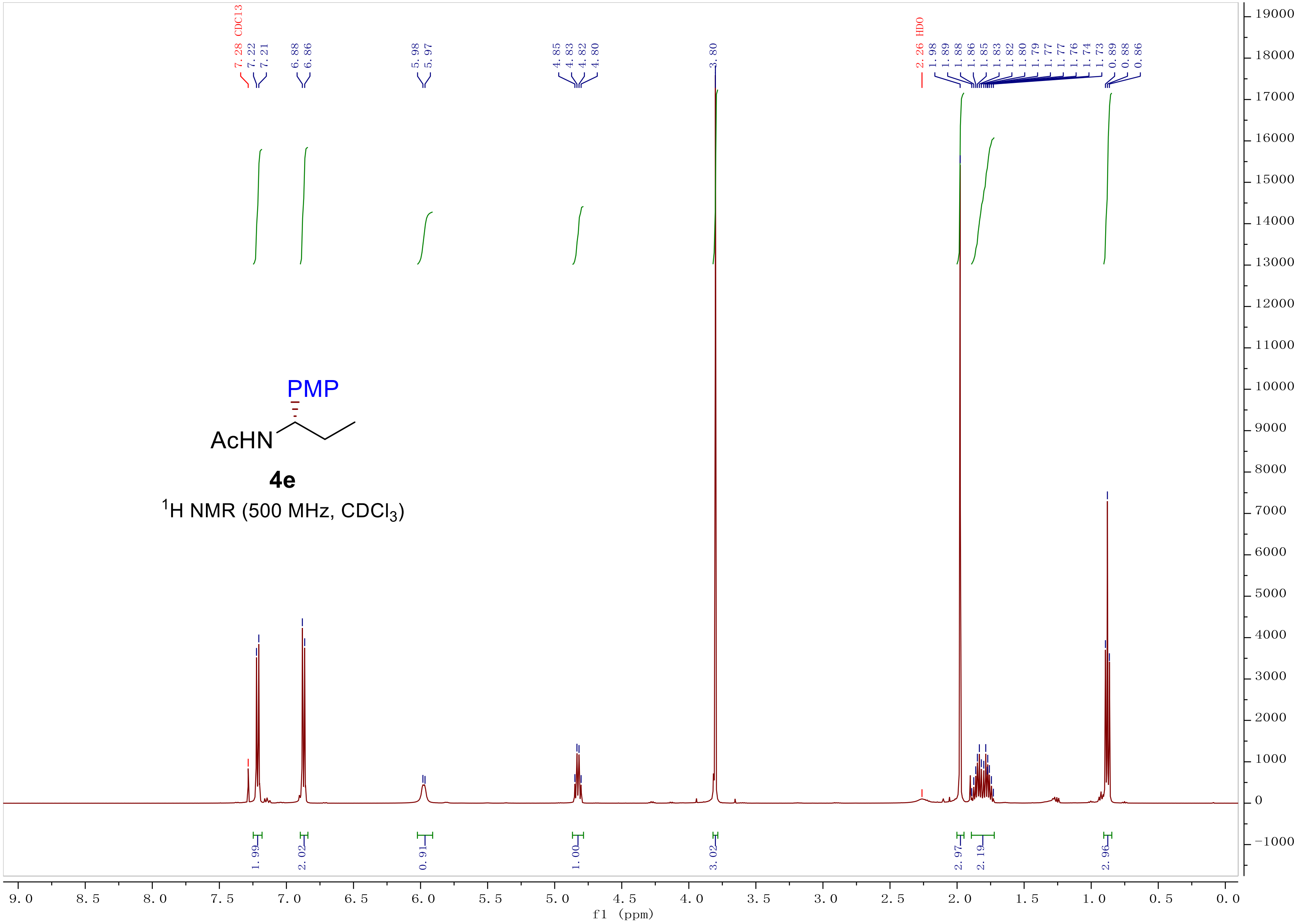

4e

^{1}H NMR (500 MHz, $CDCl_3$)

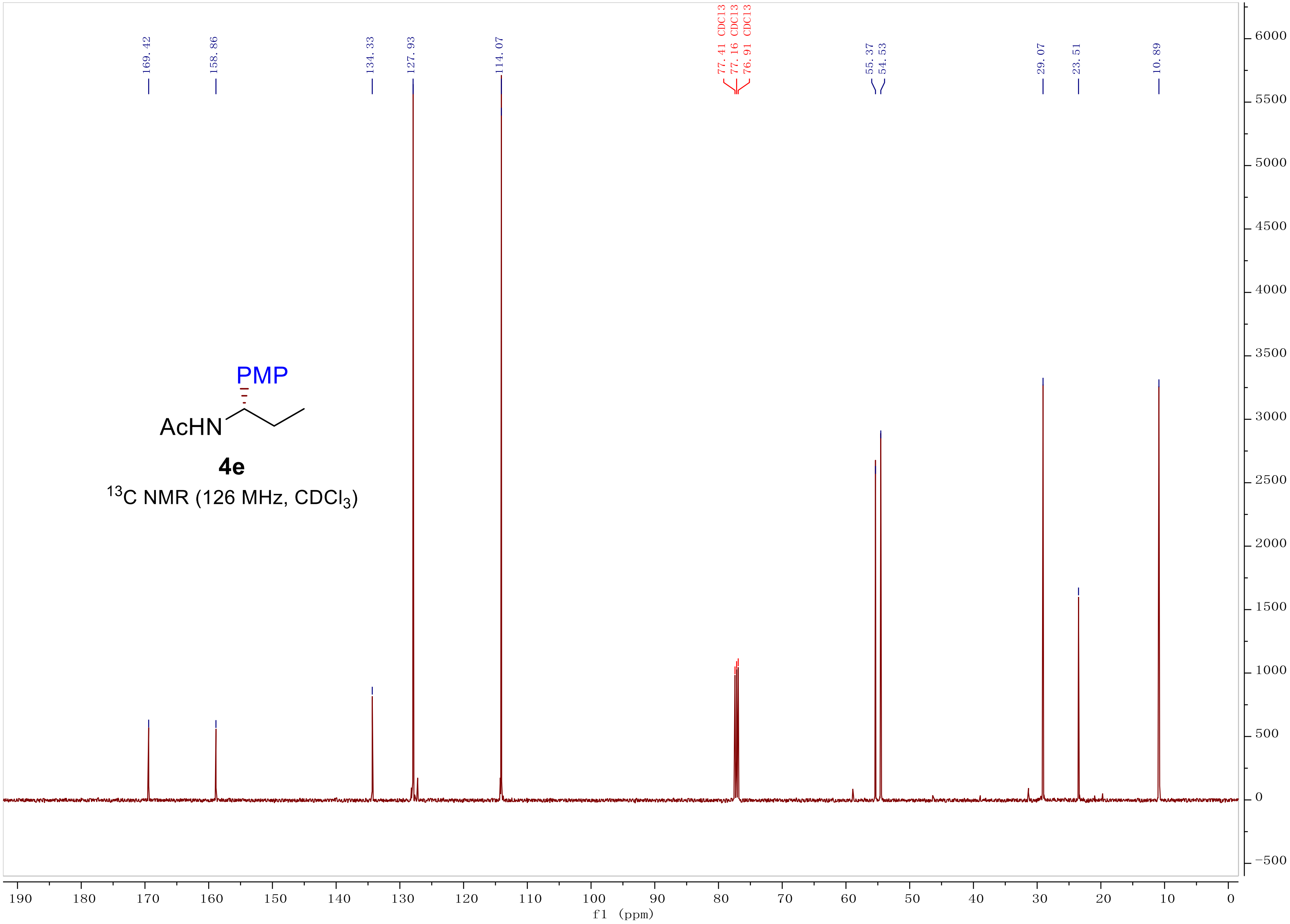
169.42
158.86
134.33
127.93
114.07
77.41 CDCl3
77.16 CDCl3
76.91 CDCl3
55.37
54.53
29.07
23.51
10.89
PMP
AcHN
4e
13C NMR (126 MHz, CDCl3)
f1 (ppm)

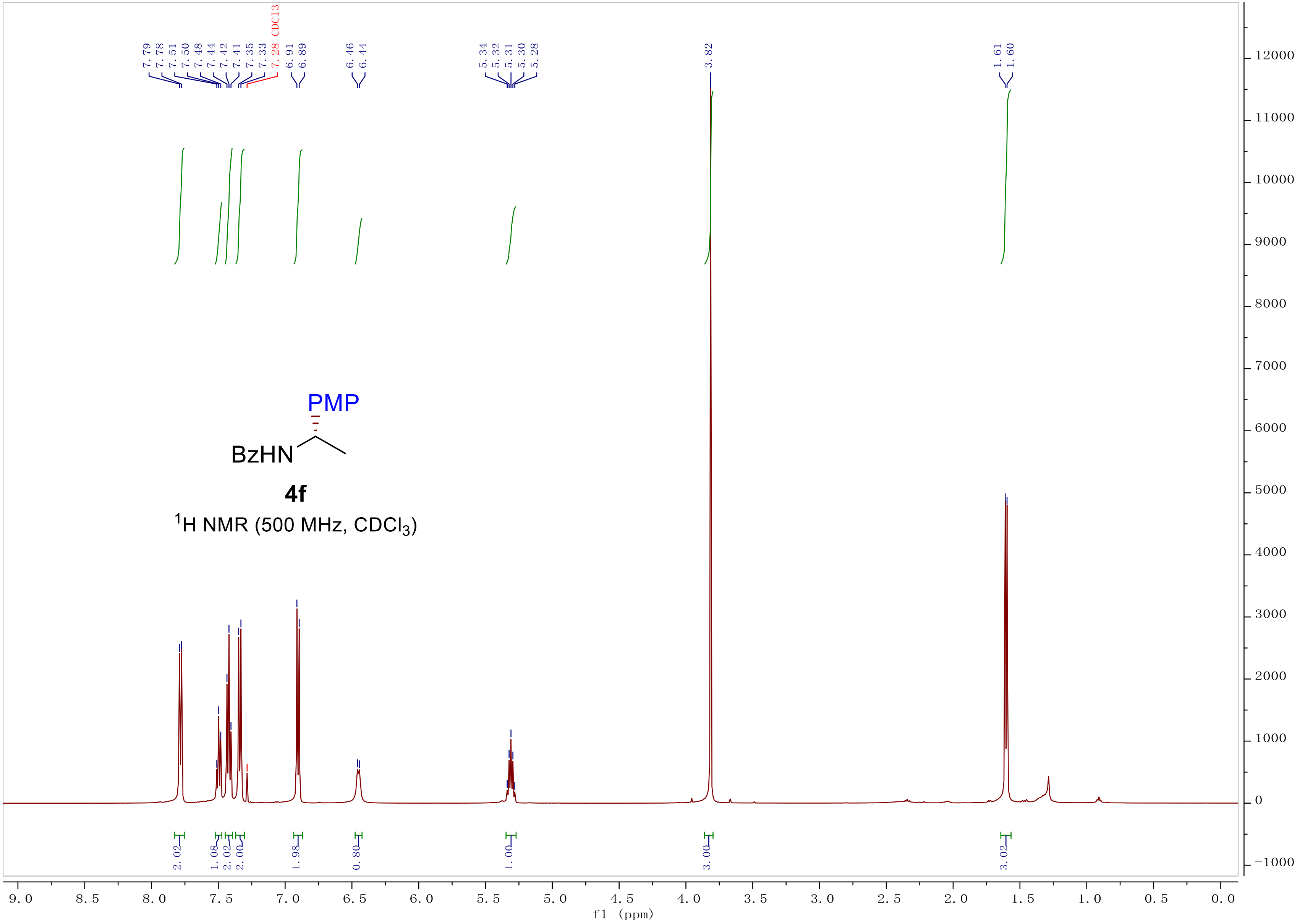
7.79
7.78
7.51
7.50
7.48
7.44
7.42
7.41
7.35
7.33
7.28 CDCl3
6.91
6.89
6.46
6.44
5.34
5.32
5.31
5.30
5.28
3.82
1.61
1.60
PMP
BzHN
4f
1H NMR (500 MHz, CDCl3)
2.02
1.08
2.02
2.00
1.98
0.80
1.00
3.00
3.02
f1 (ppm)

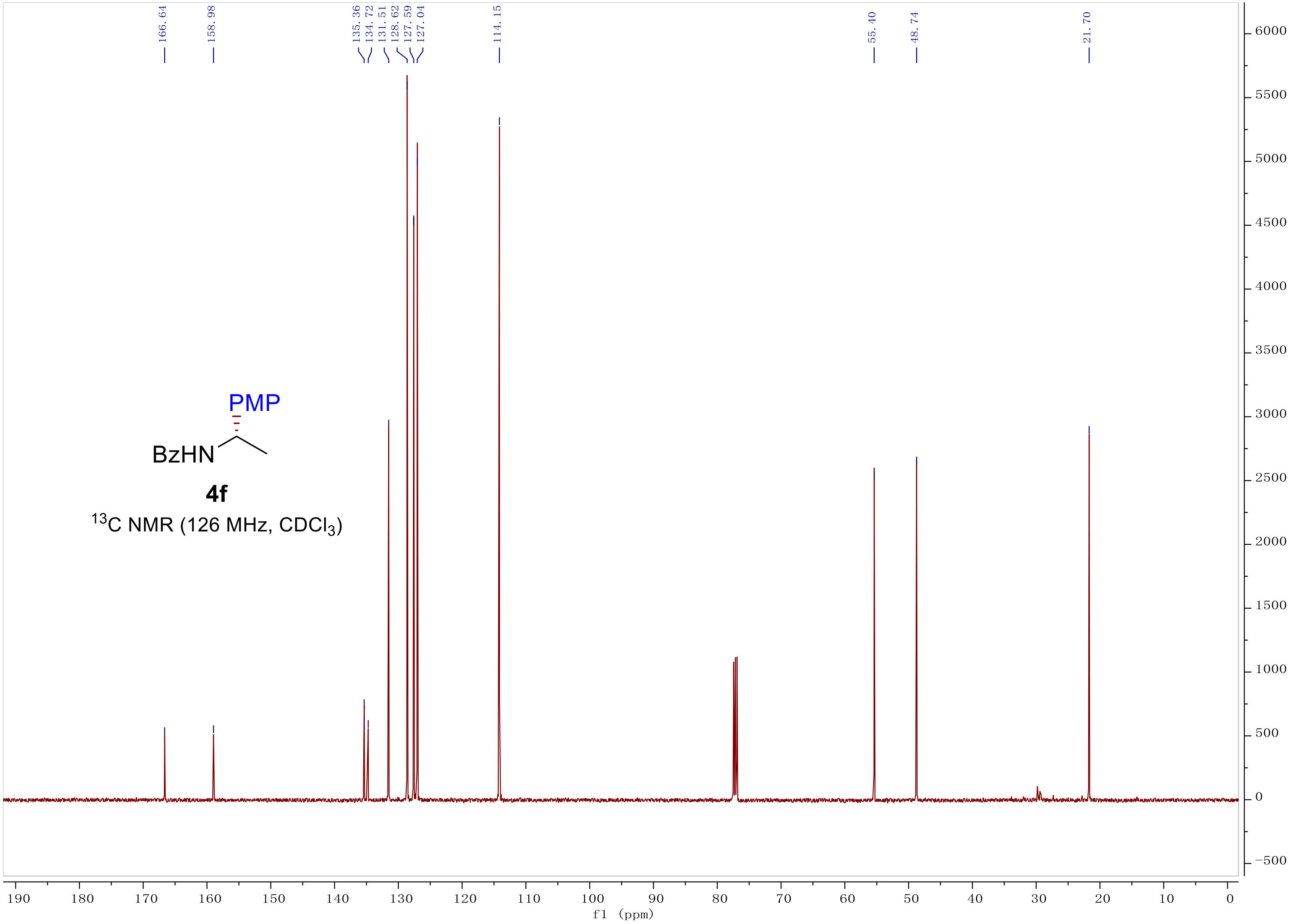

166.64
158.98
135.36
134.72
131.51
128.62
127.59
127.04
114.15
55.40
48.74
21.70
PMP
BzHN
4f
^{13}C NMR (126 MHz, CDCl$_3$)
f1 (ppm)

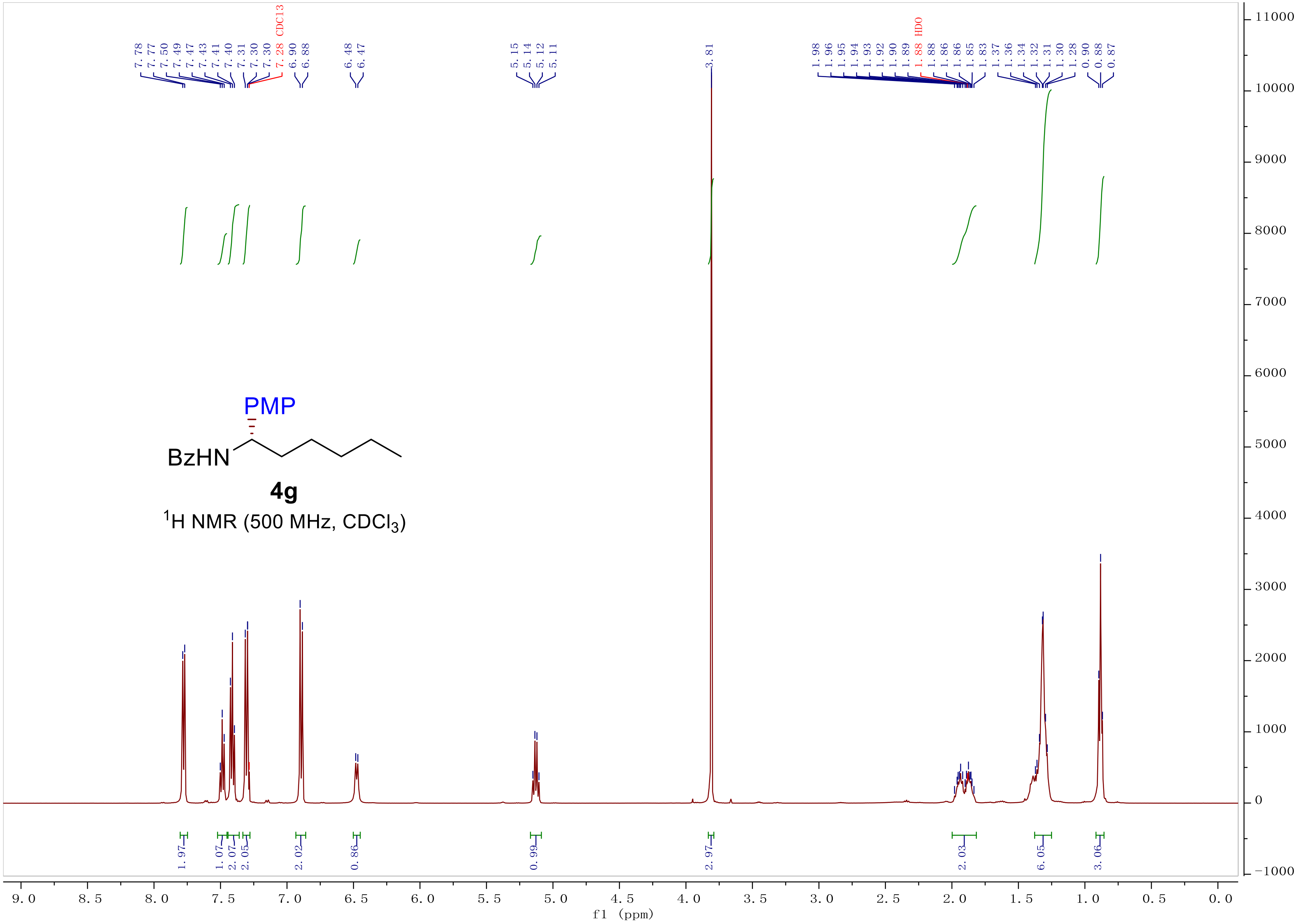

7.78 7.77 7.50 7.49 7.47 7.43 7.41 7.40 7.31 7.30 7.30 7.28 CDCl3 6.90 6.88 6.48 6.47
5.15 5.14 5.12 5.11
3.81
1.98 1.96 1.95 1.94 1.93 1.92 1.90 1.89 1.88 HDO 1.88 1.86 1.85 1.83 1.37 1.36 1.34 1.32 1.31 1.30 1.28 0.90 0.88 0.87
PMP
BzHN
4g
^{1}H NMR (500 MHz, CDCl$_3$)
1.97 1.07 2.07 2.05 2.02 0.86 0.99 2.97 2.03 6.05 3.06
f1 (ppm)

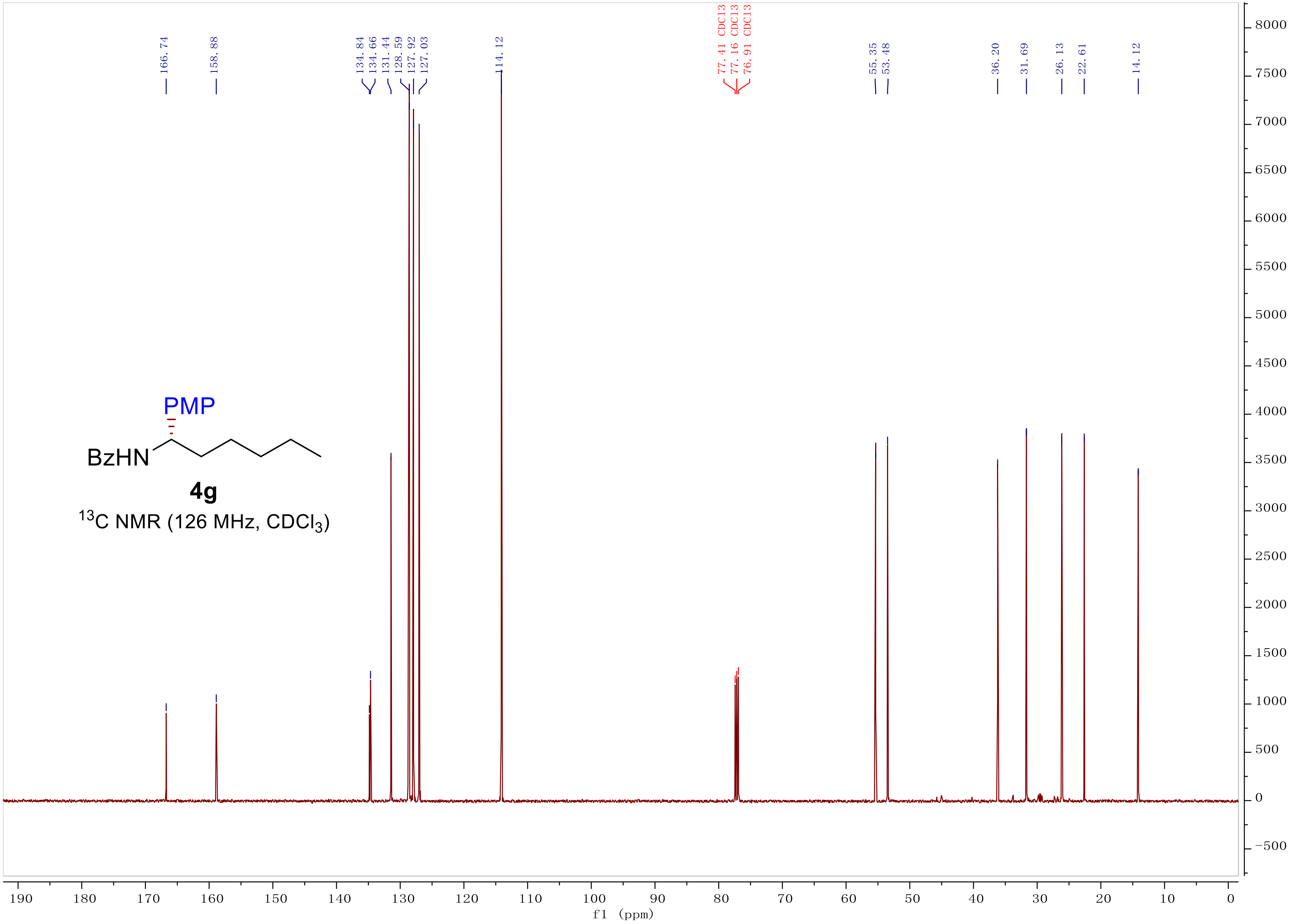
166.74
158.88
134.84
134.66
131.44
128.59
127.92
127.03
114.12
77.41 CDCl3
77.16 CDCl3
76.91 CDCl3
55.35
53.48
36.20
31.69
26.13
22.61
14.12
PMP
BzHN
4g
13C NMR (126 MHz, CDCl3)
f1 (ppm)

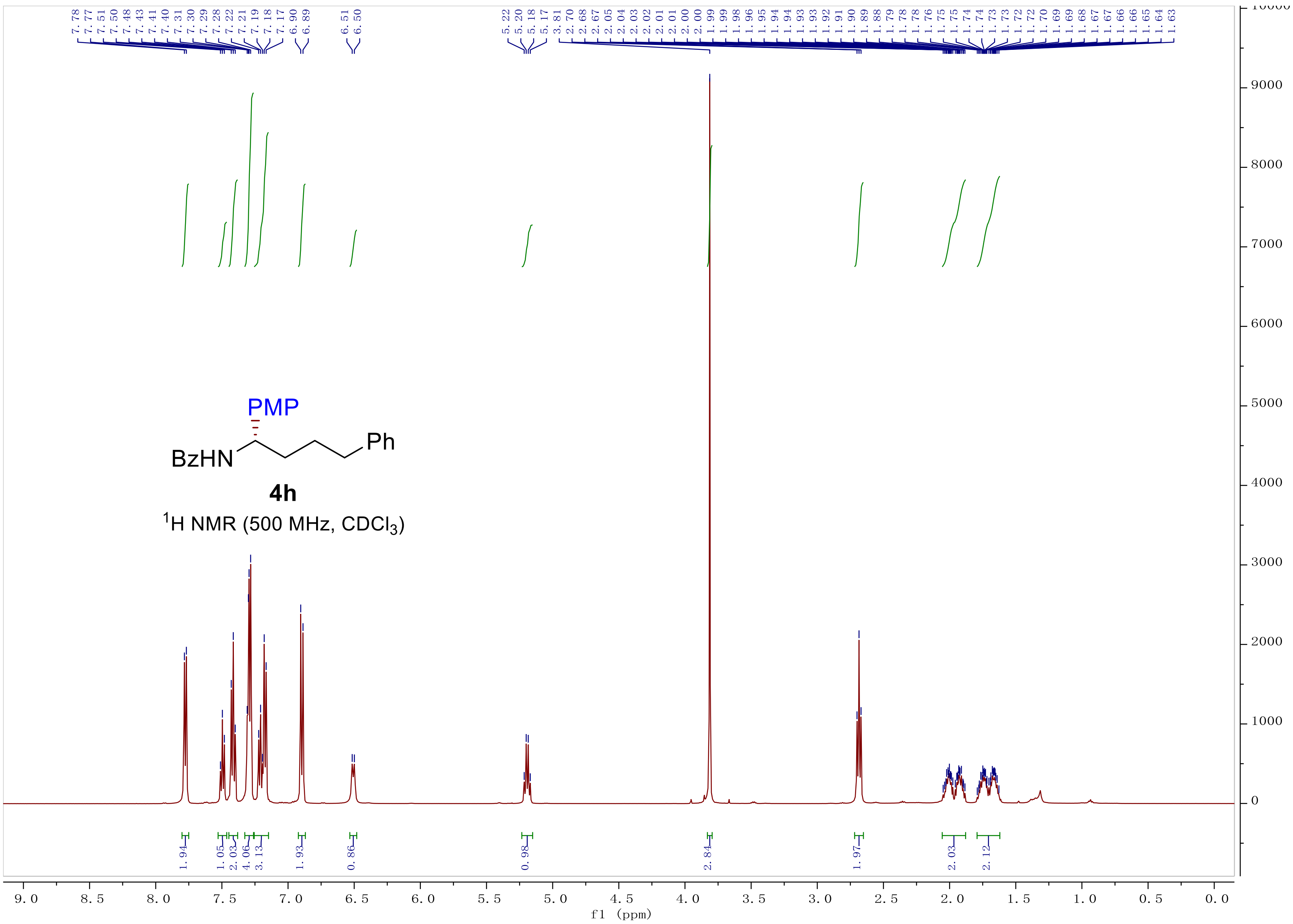

PMP
BzHN
Ph
4h
1H NMR (500 MHz, CDCl3)
f1 (ppm)

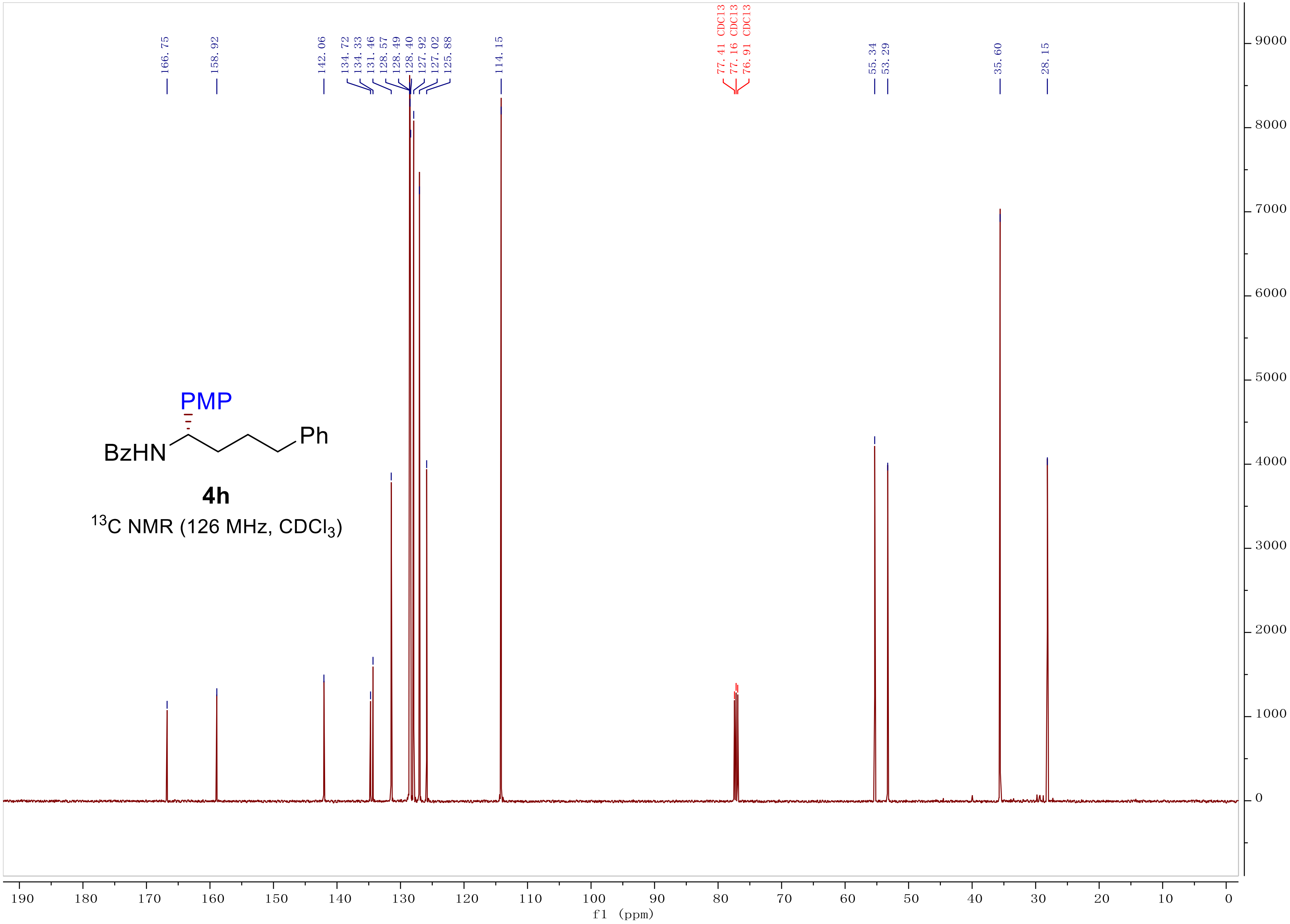

4h

^{13}C NMR (126 MHz, CDCl$_3$)

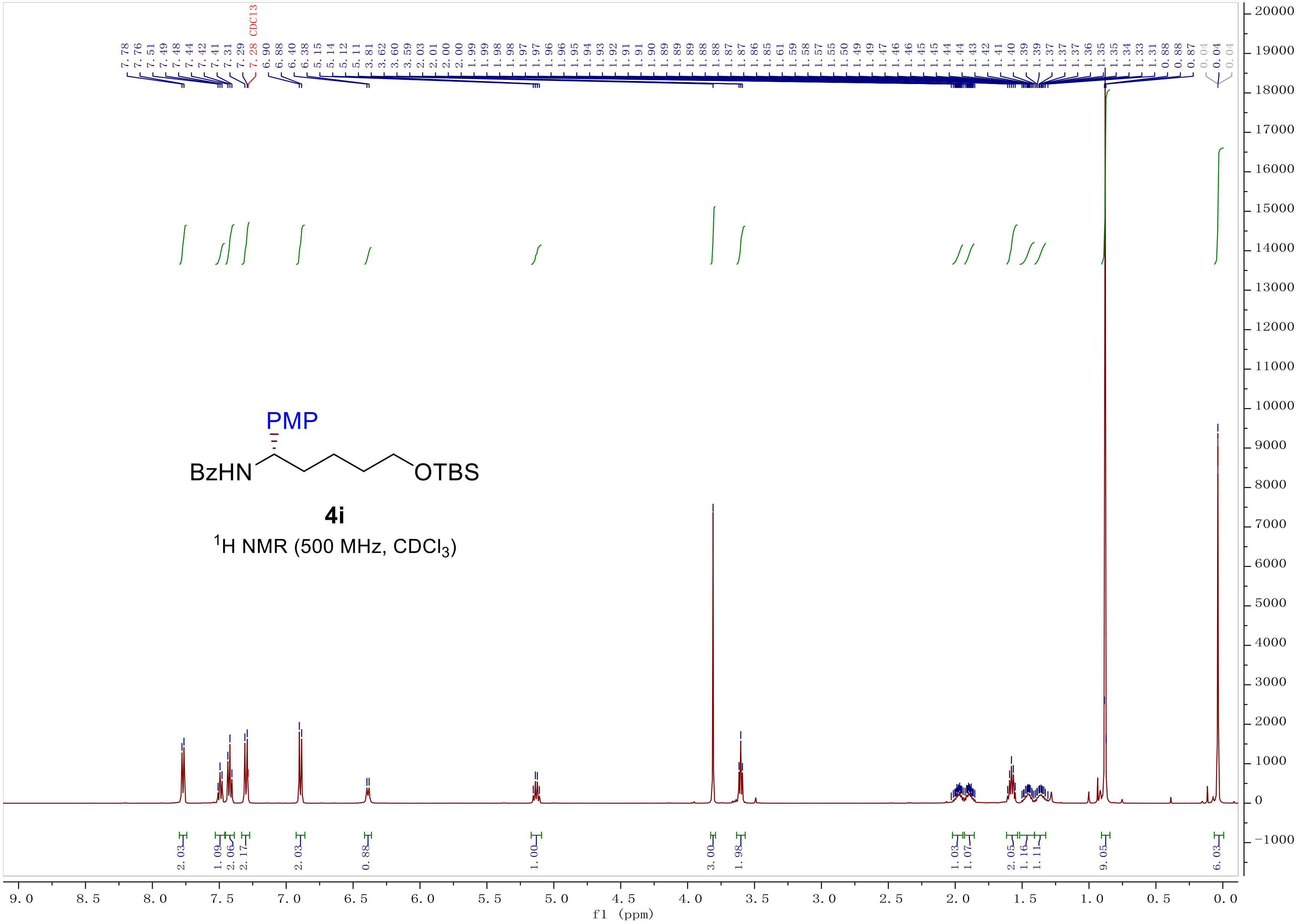

4i

^{1}H NMR (500 MHz, $CDCl_3$)

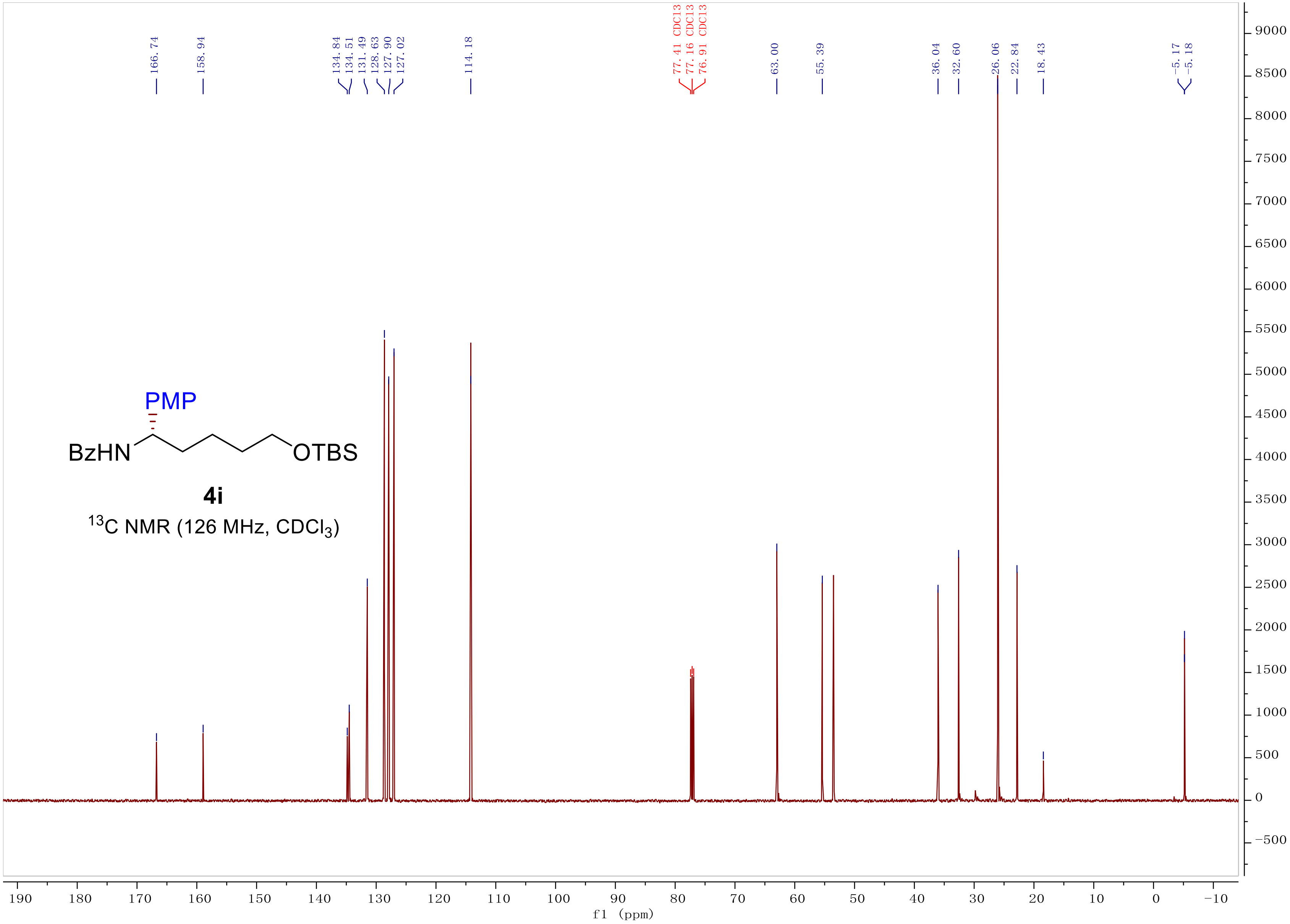

4i

^{13}C NMR (126 MHz, $CDCl_3$)

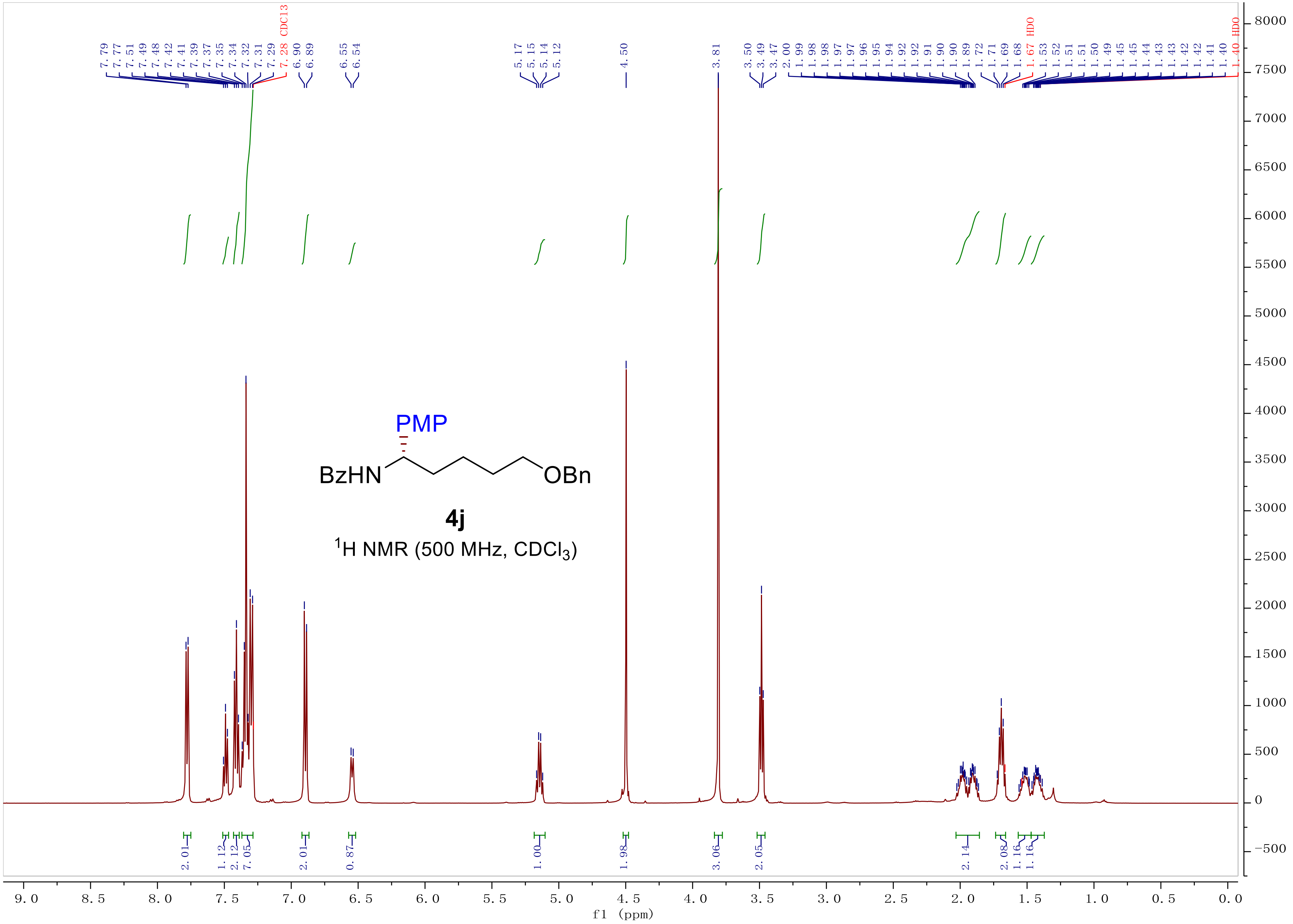
PMP
BzHN
OBn
4j
^{1}H NMR (500 MHz, $CDCl_3$)
f1 (ppm)

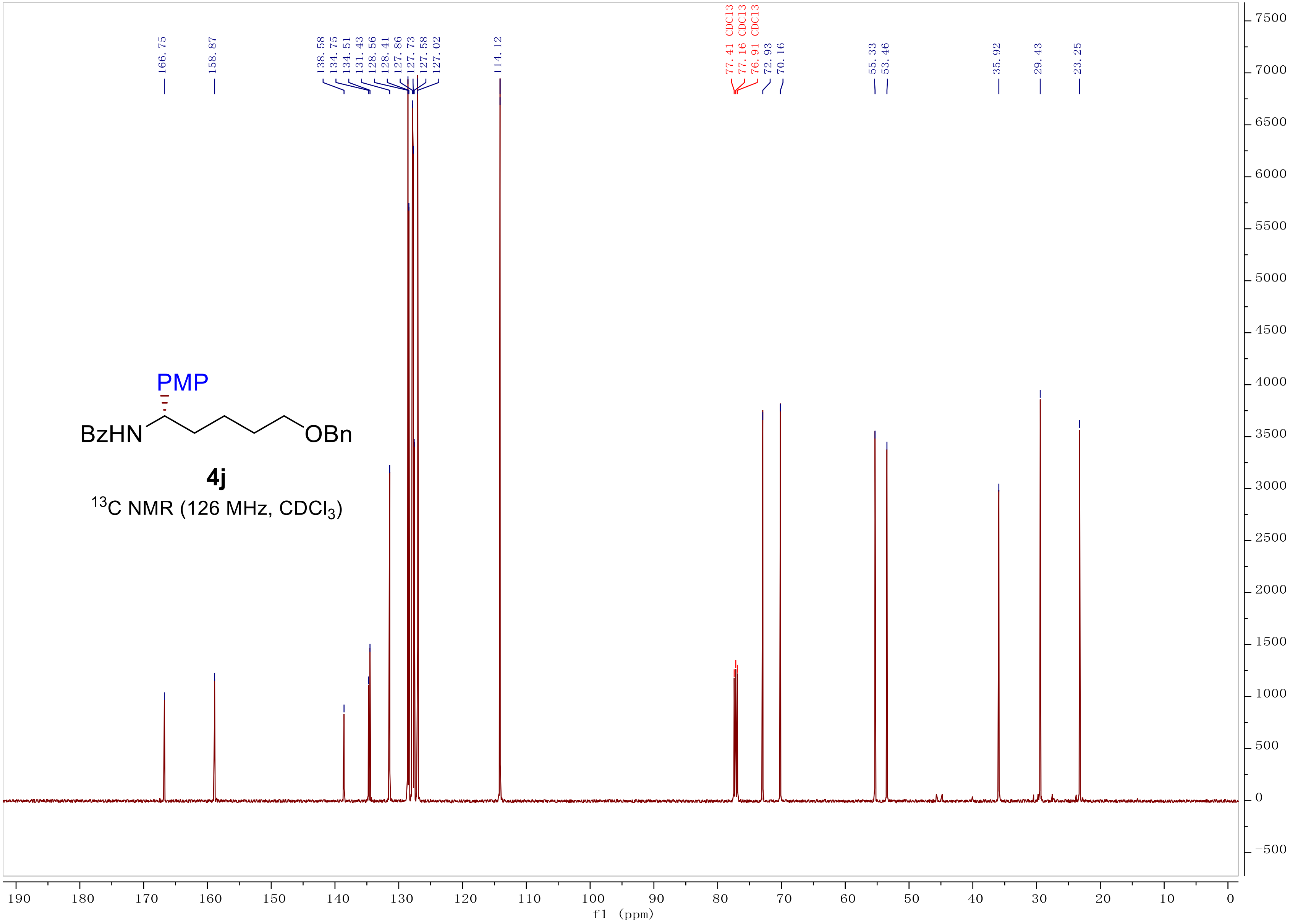
166.75
158.87
138.58
134.75
134.51
131.43
128.56
128.41
127.86
127.73
127.58
127.02
114.12
77.41 CDCl3
77.16 CDCl3
76.91 CDCl3
72.93
70.16
55.33
53.46
35.92
29.43
23.25
PMP
BzHN
OBn
4j
^{13}C NMR (126 MHz, $CDCl_3$)
f1 (ppm)

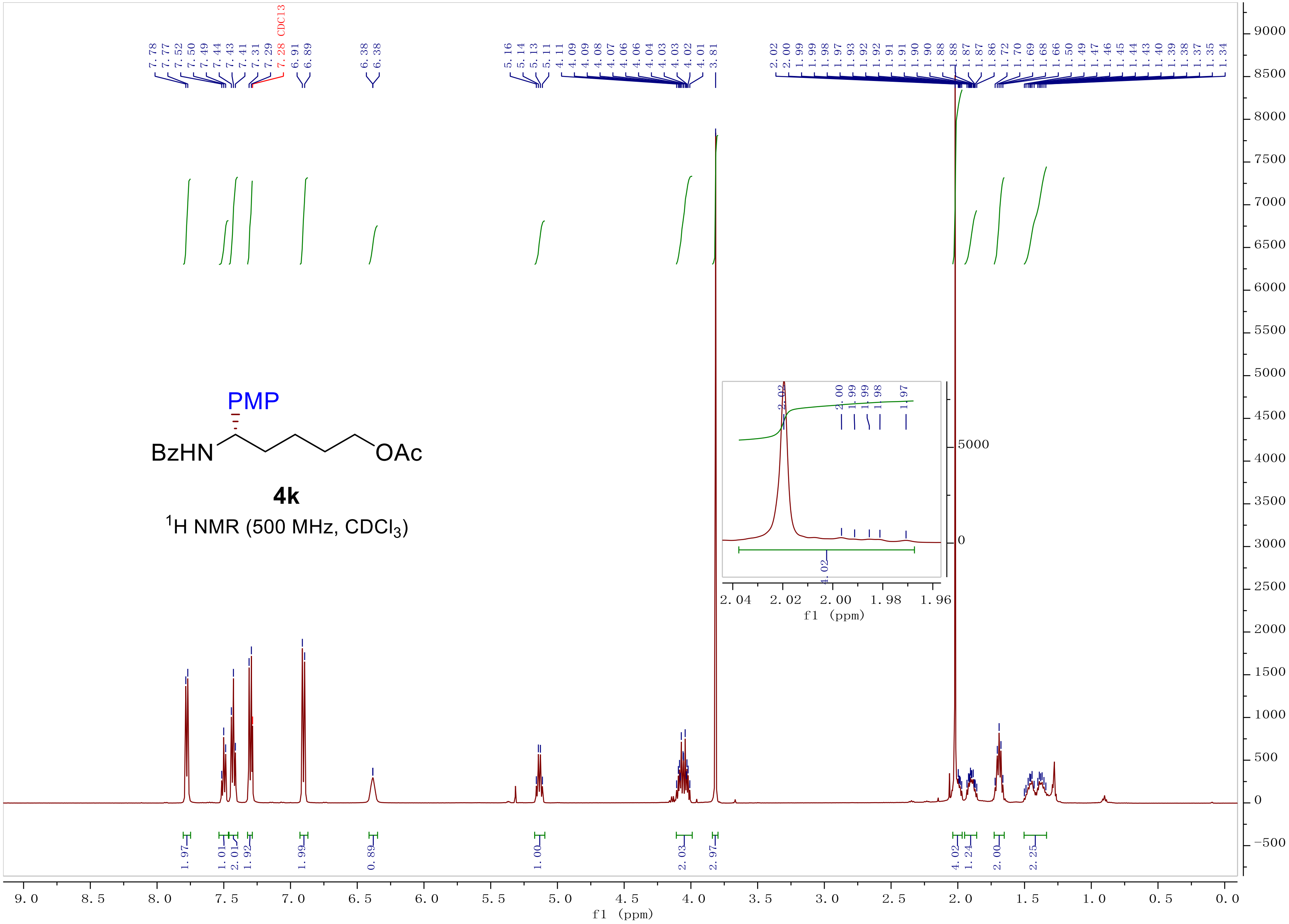
PMP
BzHN
OAc
4k
^{1}H NMR (500 MHz, CDCl$_3$)
f1 (ppm)

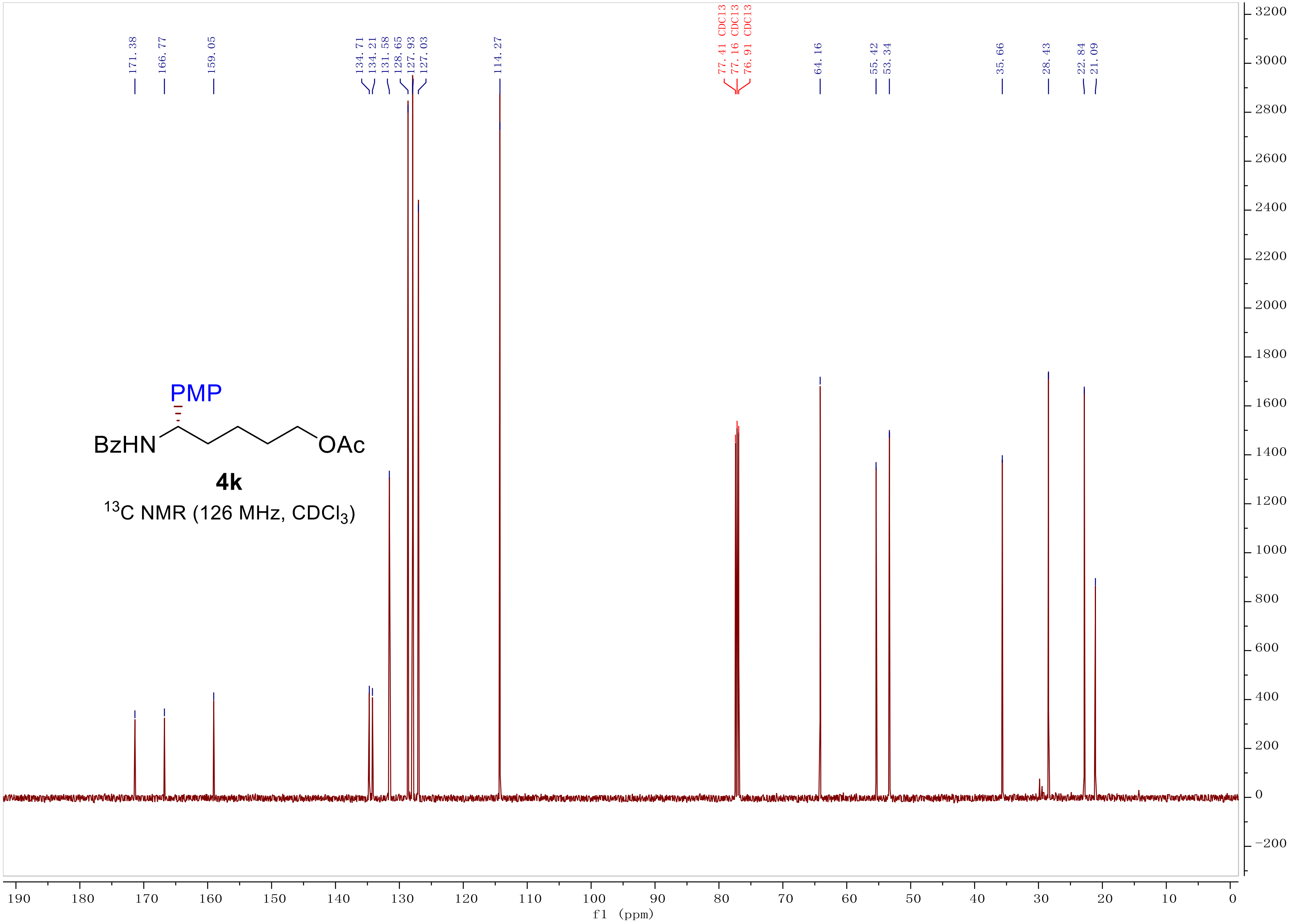

171.38
166.77
159.05
134.71
134.21
131.58
128.65
127.93
127.03
114.27
77.41 CDCl3
77.16 CDCl3
76.91 CDCl3
64.16
55.42
53.34
35.66
28.43
22.84
21.09
PMP
BzHN
OAc
4k
13C NMR (126 MHz, CDCl3)
f1 (ppm)

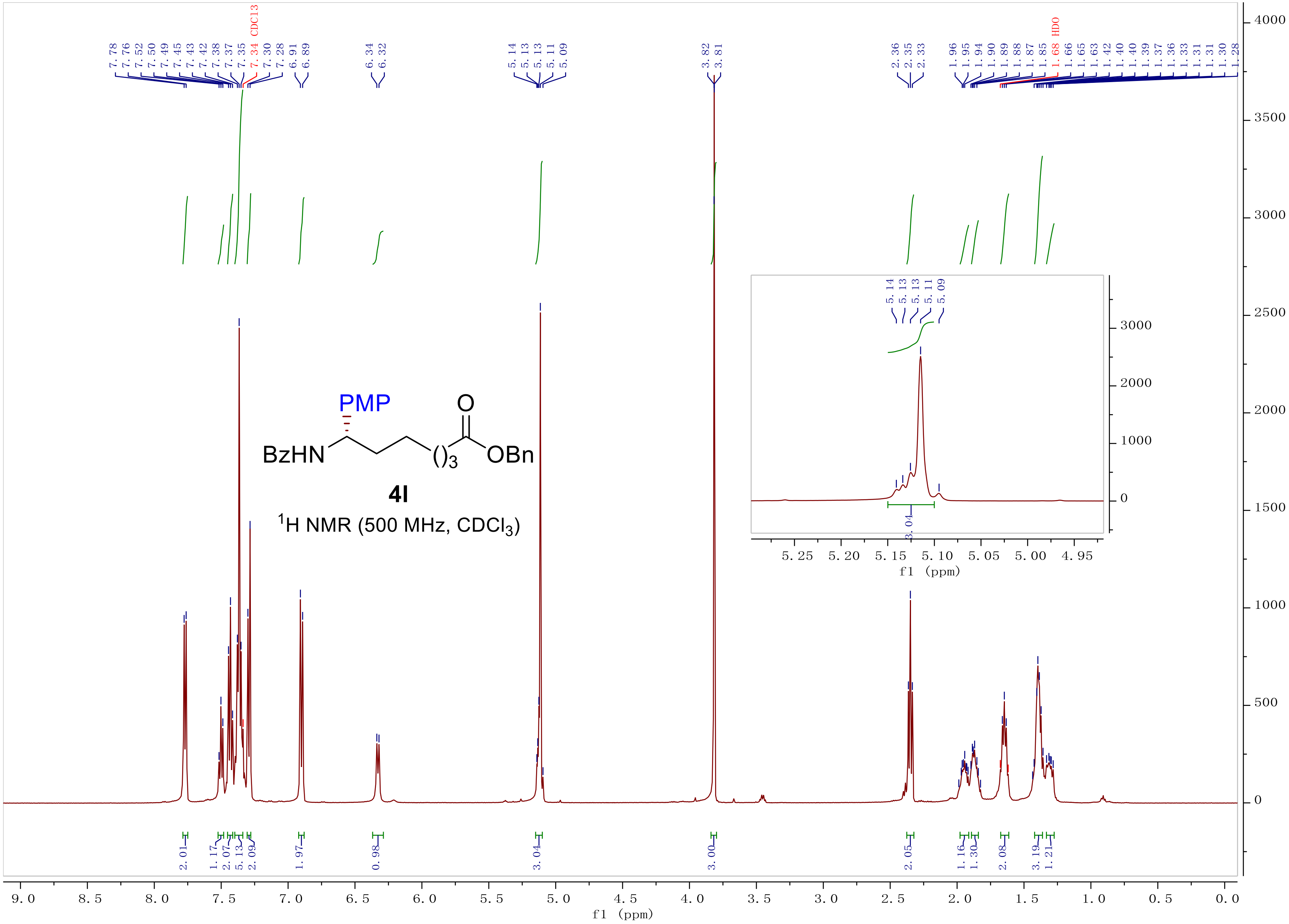
PMP
BzHN
O
OBn
4l
^{1}H NMR (500 MHz, CDCl$_3$)
f1 (ppm)

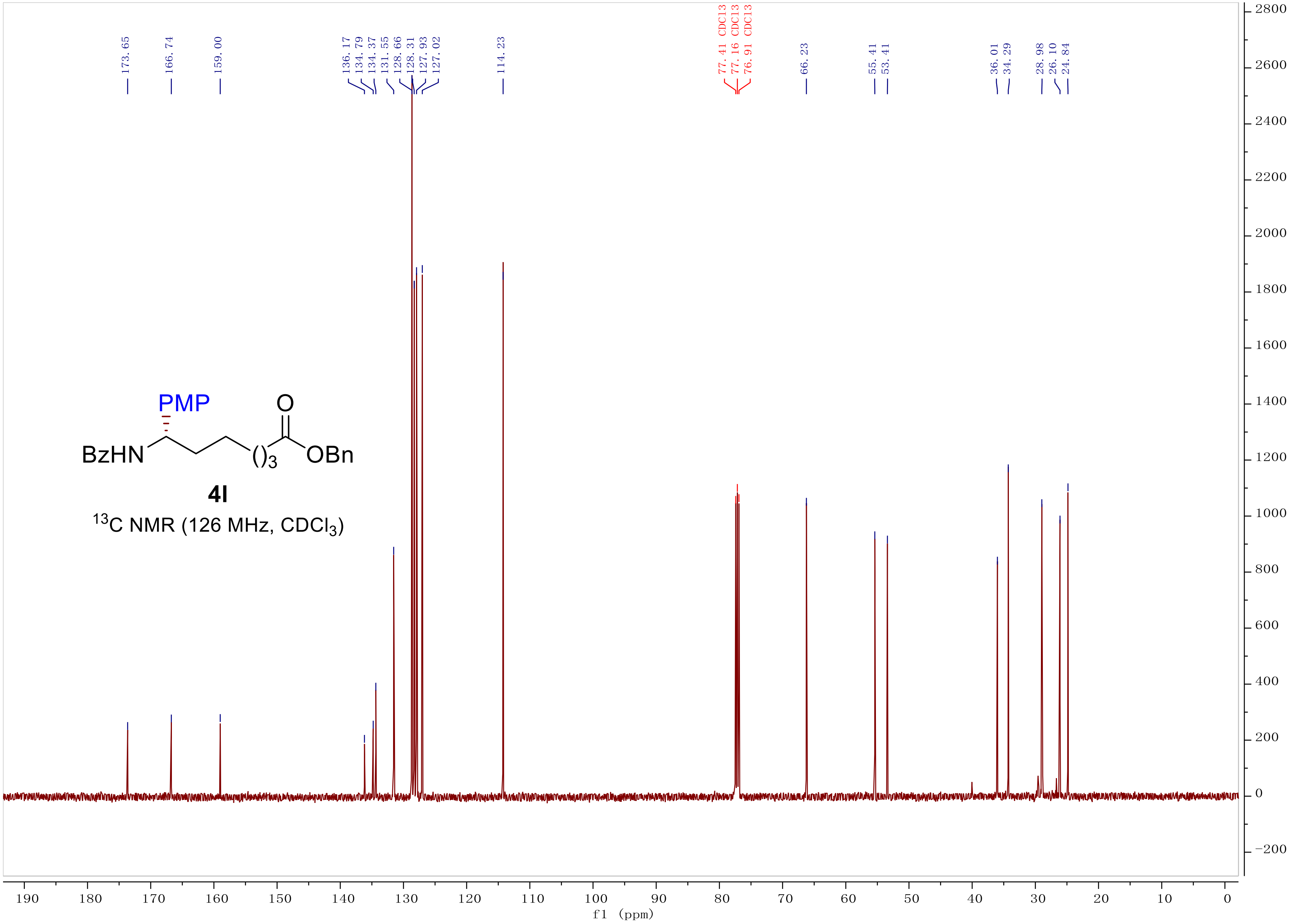

4l

^{13}C NMR (126 MHz, $CDCl_3$)

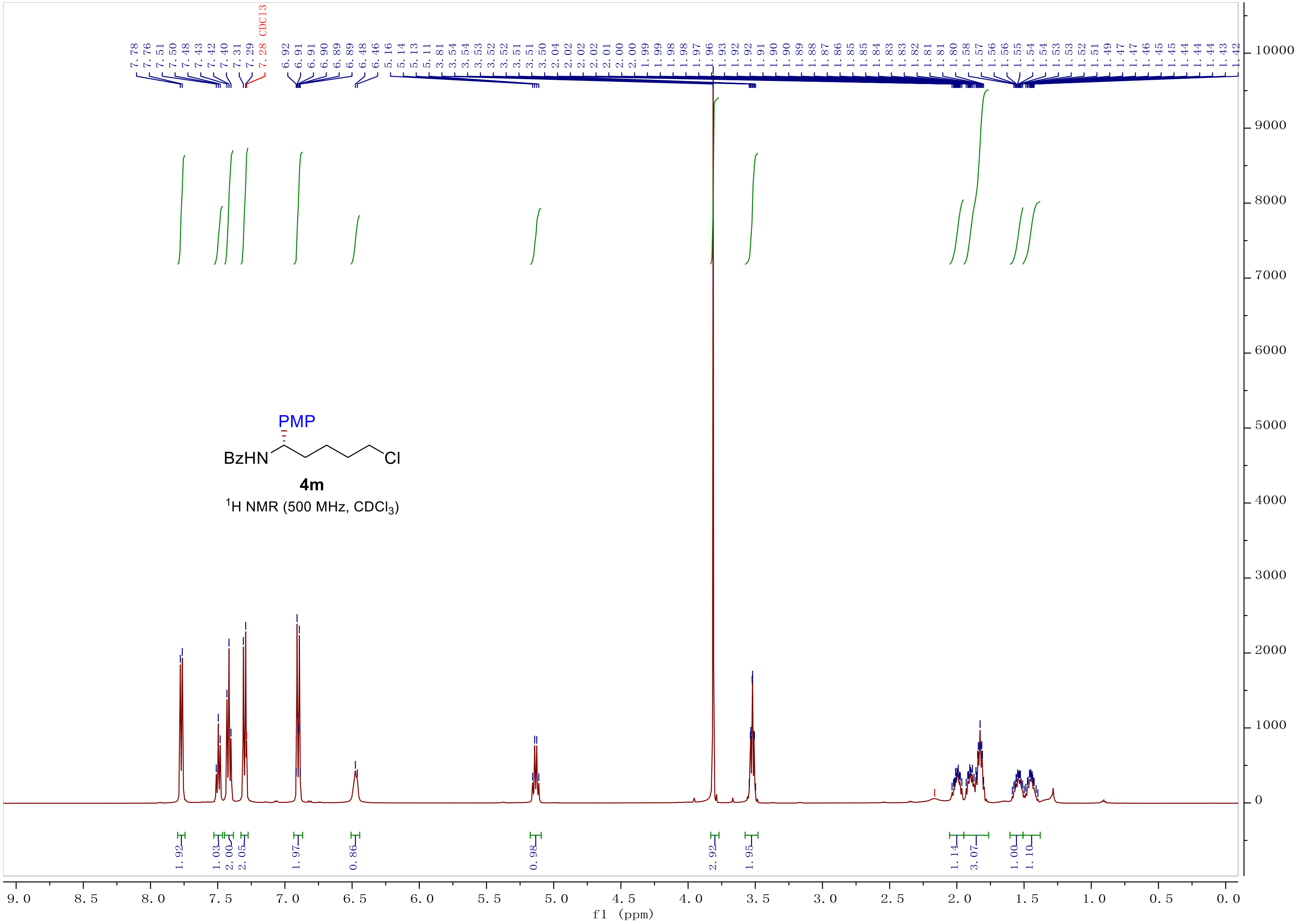
PMP
BzHN
Cl
4m
^{1}H NMR (500 MHz, CDCl$_3$)
f1 (ppm)
CDCl3

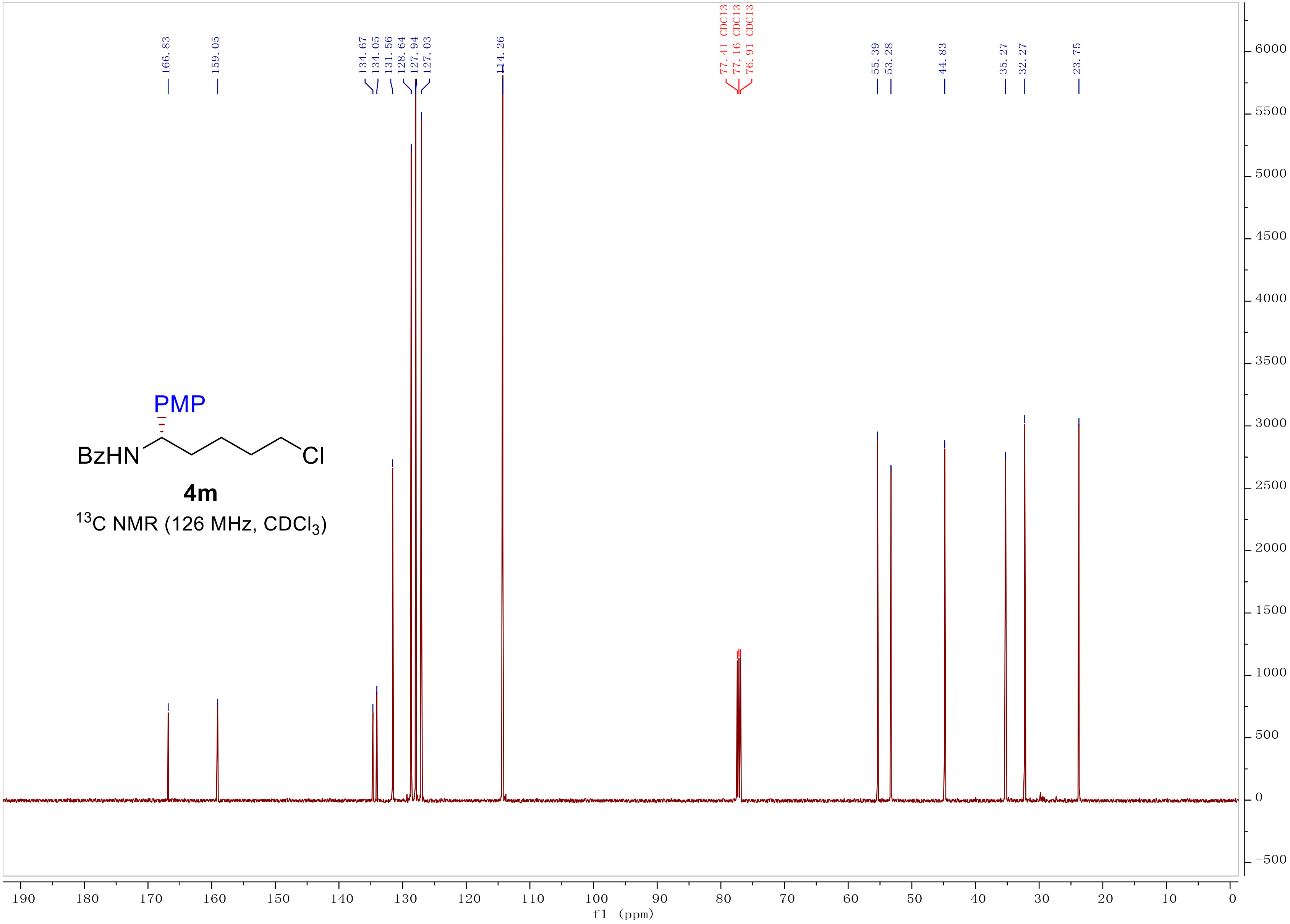
166.83
159.05
134.67
134.05
131.56
128.64
127.94
127.03
114.26
77.41 CDCl3
77.16 CDCl3
76.91 CDCl3
55.39
53.28
44.83
35.27
32.27
23.75
PMP
BzHN
Cl
4m
13C NMR (126 MHz, CDCl3)
f1 (ppm)

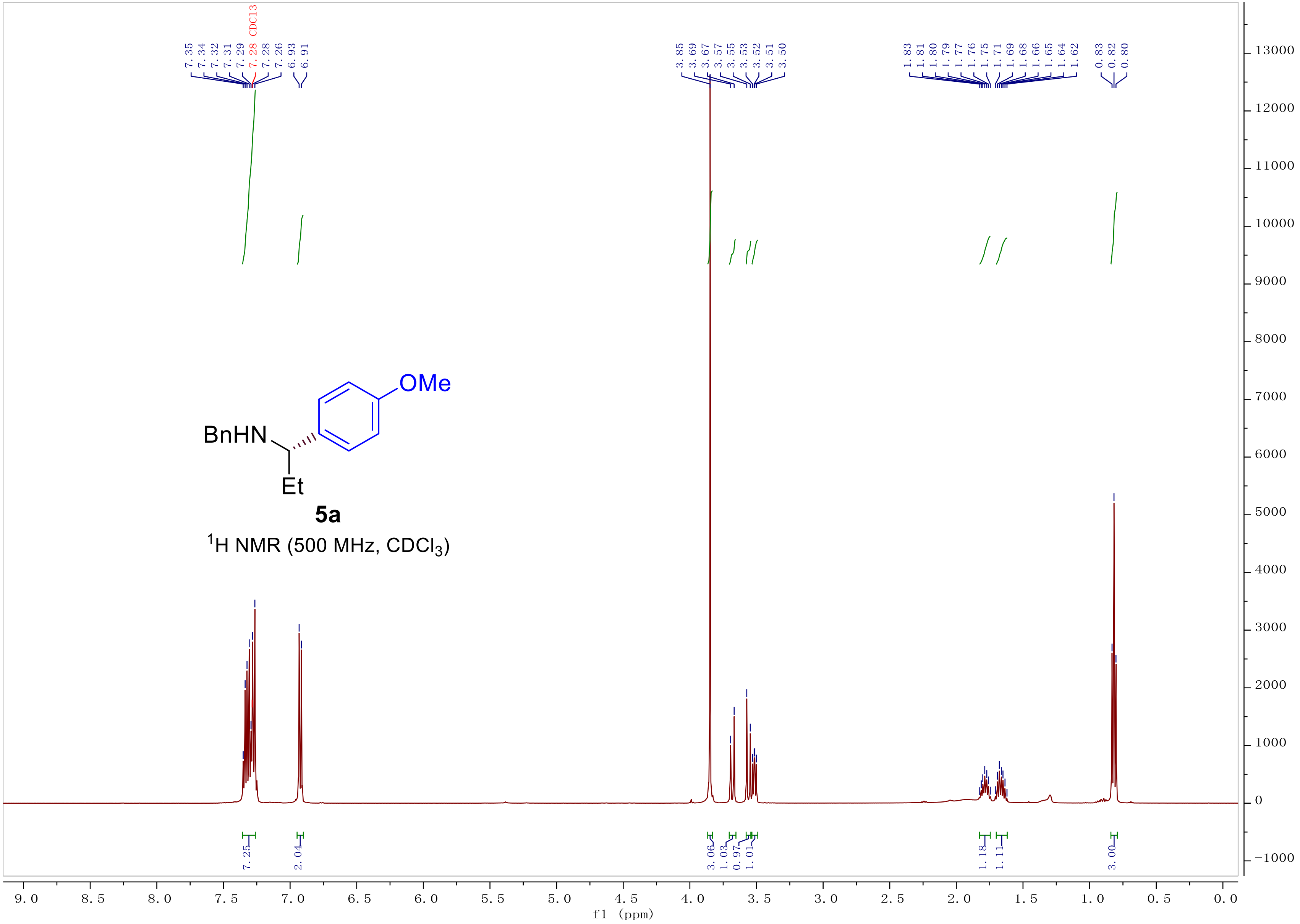

5a

^{1}H NMR (500 MHz, $CDCl_3$)

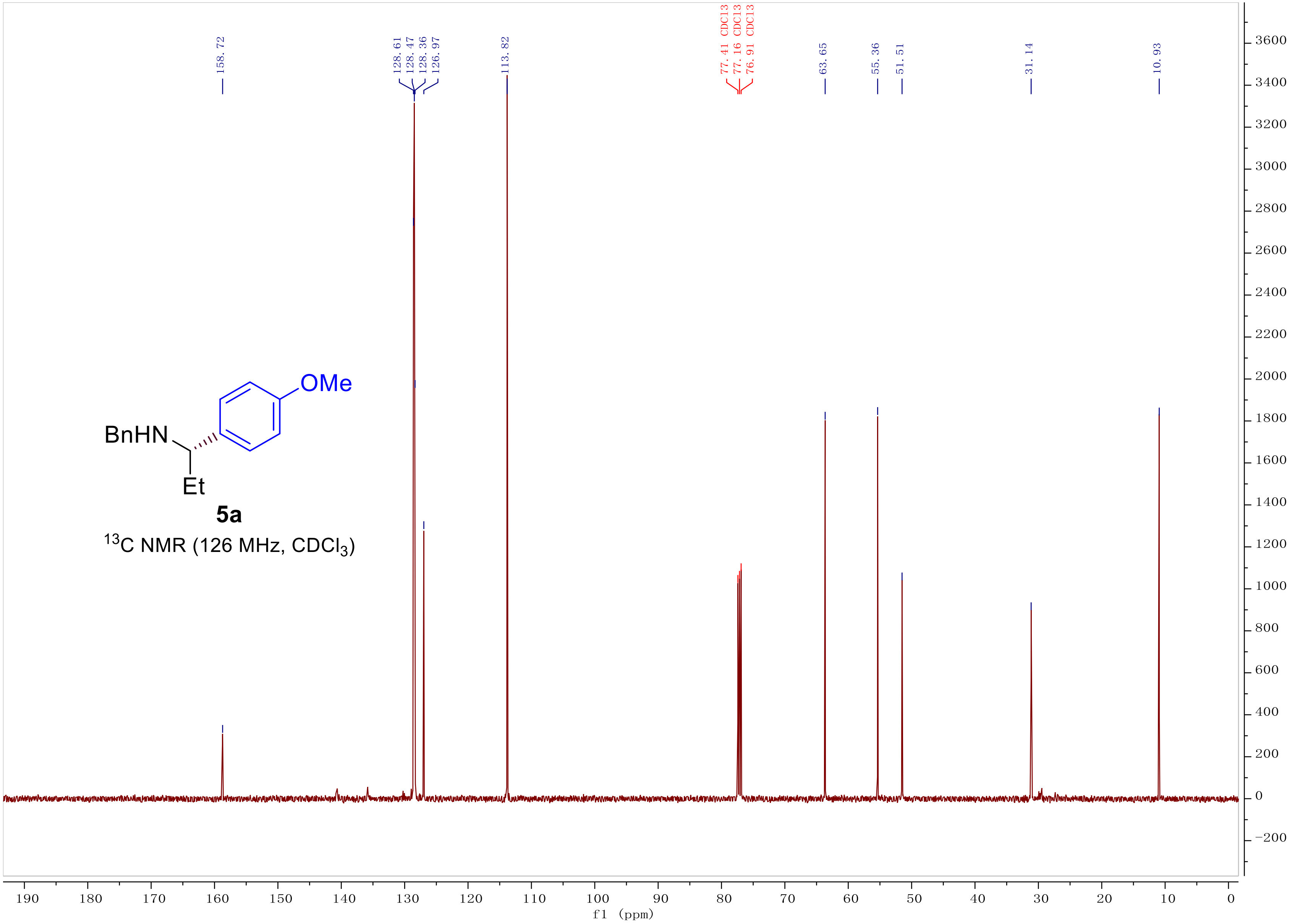
158.72
128.61
128.47
128.36
126.97
113.82
77.41 CDCl3
77.16 CDCl3
76.91 CDCl3
63.65
55.36
51.51
31.14
10.93
BnHN
OMe
Et
5a
13C NMR (126 MHz, CDCl3)
f1 (ppm)

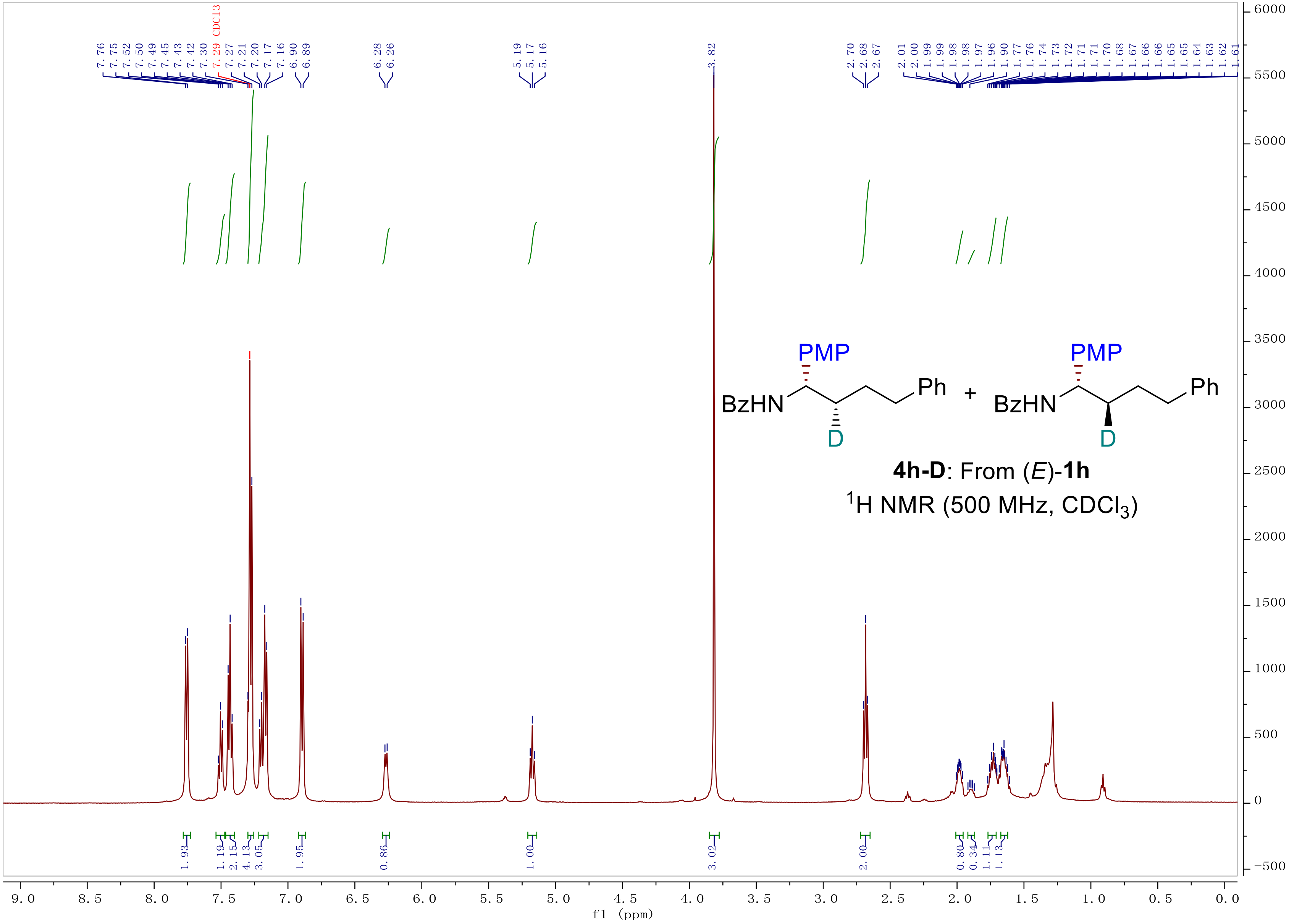

7.76
7.75
7.52
7.50
7.49
7.45
7.43
7.42
7.30
7.29 CDCl3
7.27
7.21
7.20
7.17
7.16
6.90
6.89
6.28
6.26
5.19
5.17
5.16
3.82
2.70
2.68
2.67
2.01
2.00
1.99
1.99
1.98
1.98
1.97
1.96
1.90
1.77
1.76
1.74
1.73
1.72
1.71
1.71
1.70
1.68
1.67
1.66
1.66
1.65
1.65
1.64
1.63
1.62
1.61
PMP
BzHN
Ph
D
+
PMP
BzHN
Ph
D
4h-D: From (*E*)-**1h**
^{1}H NMR (500 MHz, $CDCl_3$)
1.93
1.19
2.15
4.13
3.05
1.95
0.86
1.00
3.02
2.00
0.80
0.34
1.11
1.13
f1 (ppm)

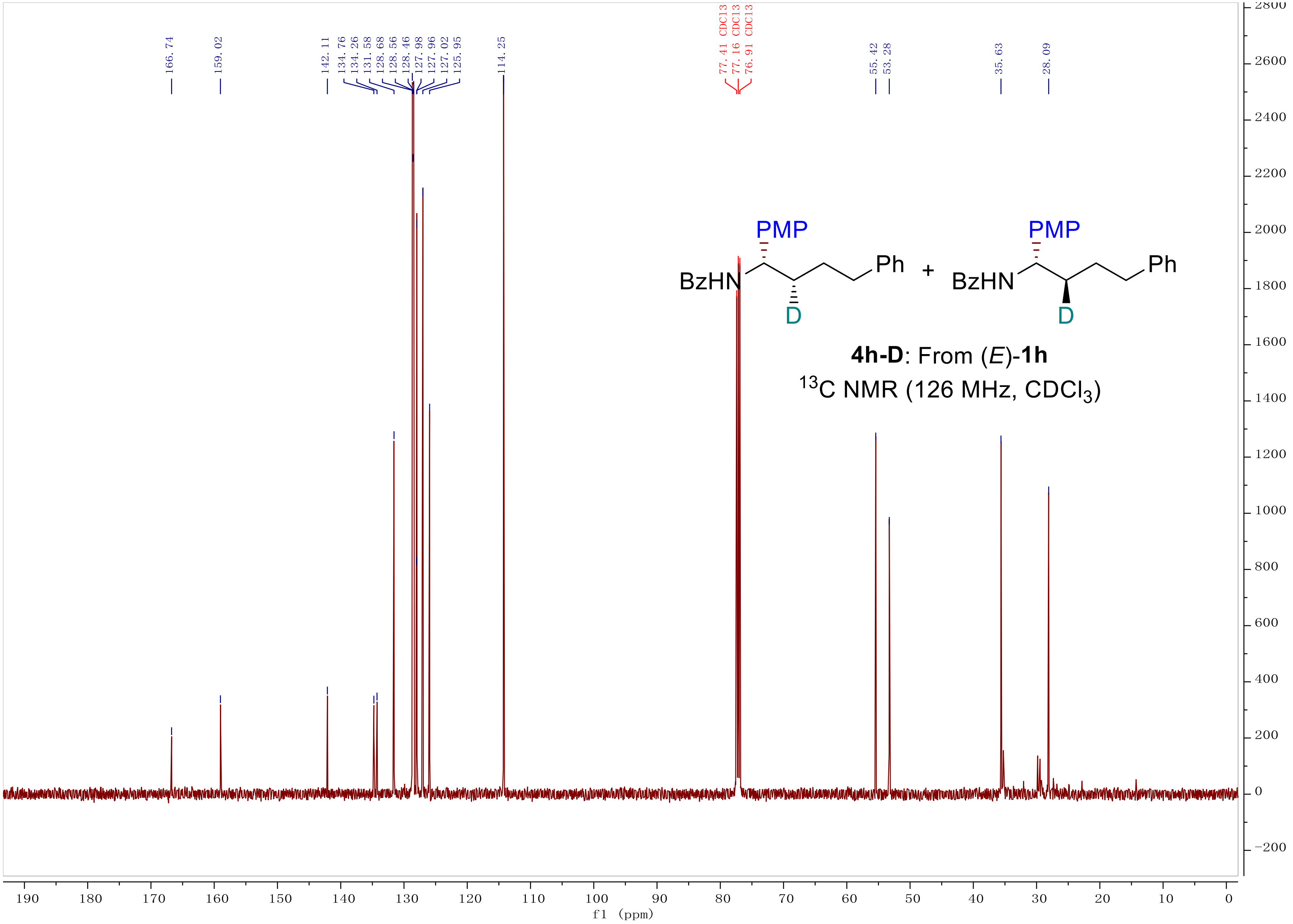

166.74
159.02
142.11
134.76
134.26
131.58
128.68
128.56
128.46
127.98
127.96
127.02
125.95
114.25
77.41 CDCl3
77.16 CDCl3
76.91 CDCl3
55.42
53.28
35.63
28.09
PMP
BzHN
Ph
+
D
4h-D: From (E)-1h
13C NMR (126 MHz, CDCl3)
f1 (ppm)

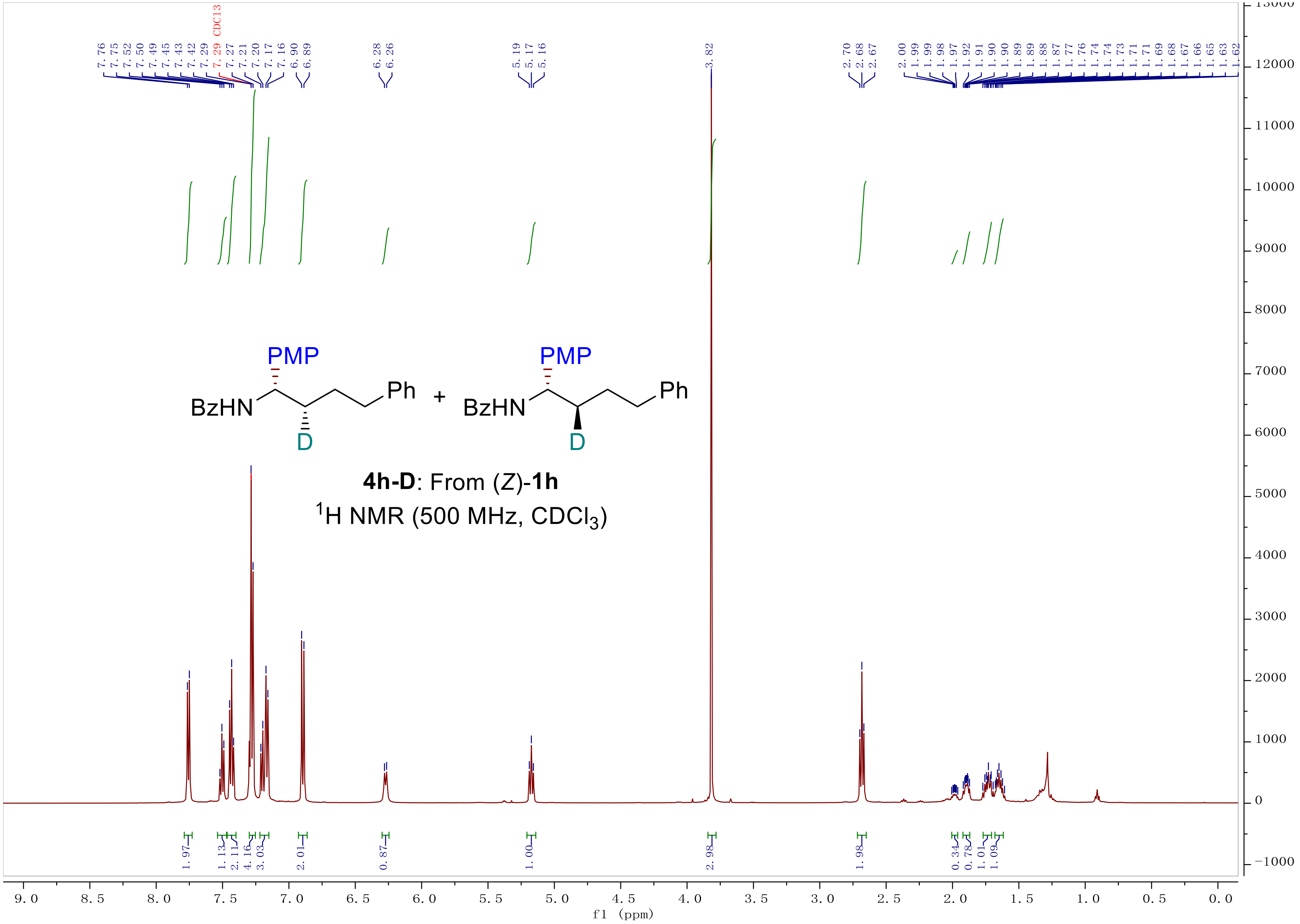
4h-D: From (Z)-1h
1H NMR (500 MHz, CDCl3)
PMP
BzHN
Ph
D
+
f1 (ppm)

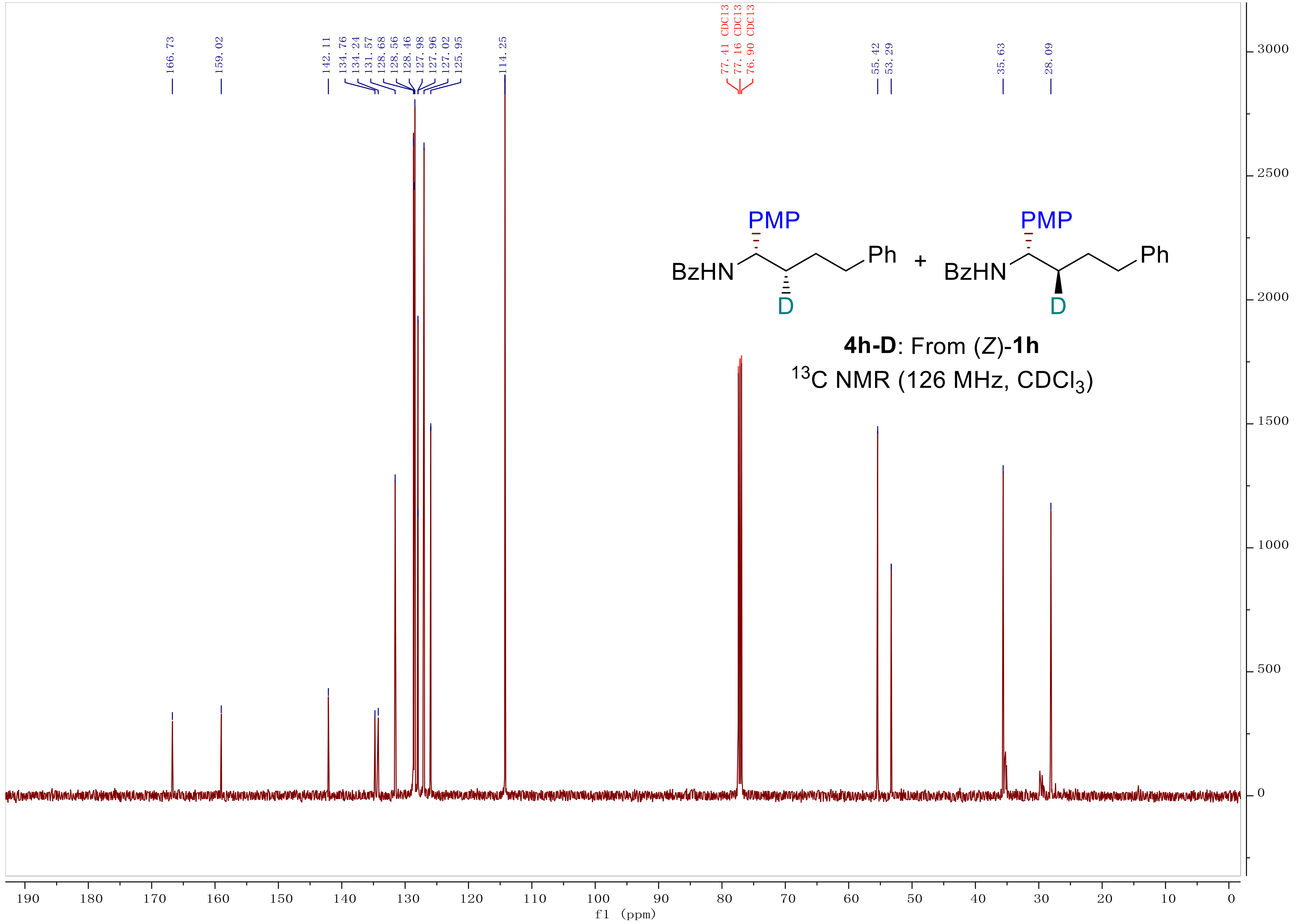

166.73
159.02
142.11
134.76
134.24
131.57
128.68
128.56
128.46
127.98
127.96
127.02
125.95
114.25
77.41 CDCl3
77.16 CDCl3
76.90 CDCl3
55.42
53.29
35.63
28.09
PMP
BzHN
Ph
D
+
4h-D: From (Z)-1h
13C NMR (126 MHz, CDCl3)
f1 (ppm)

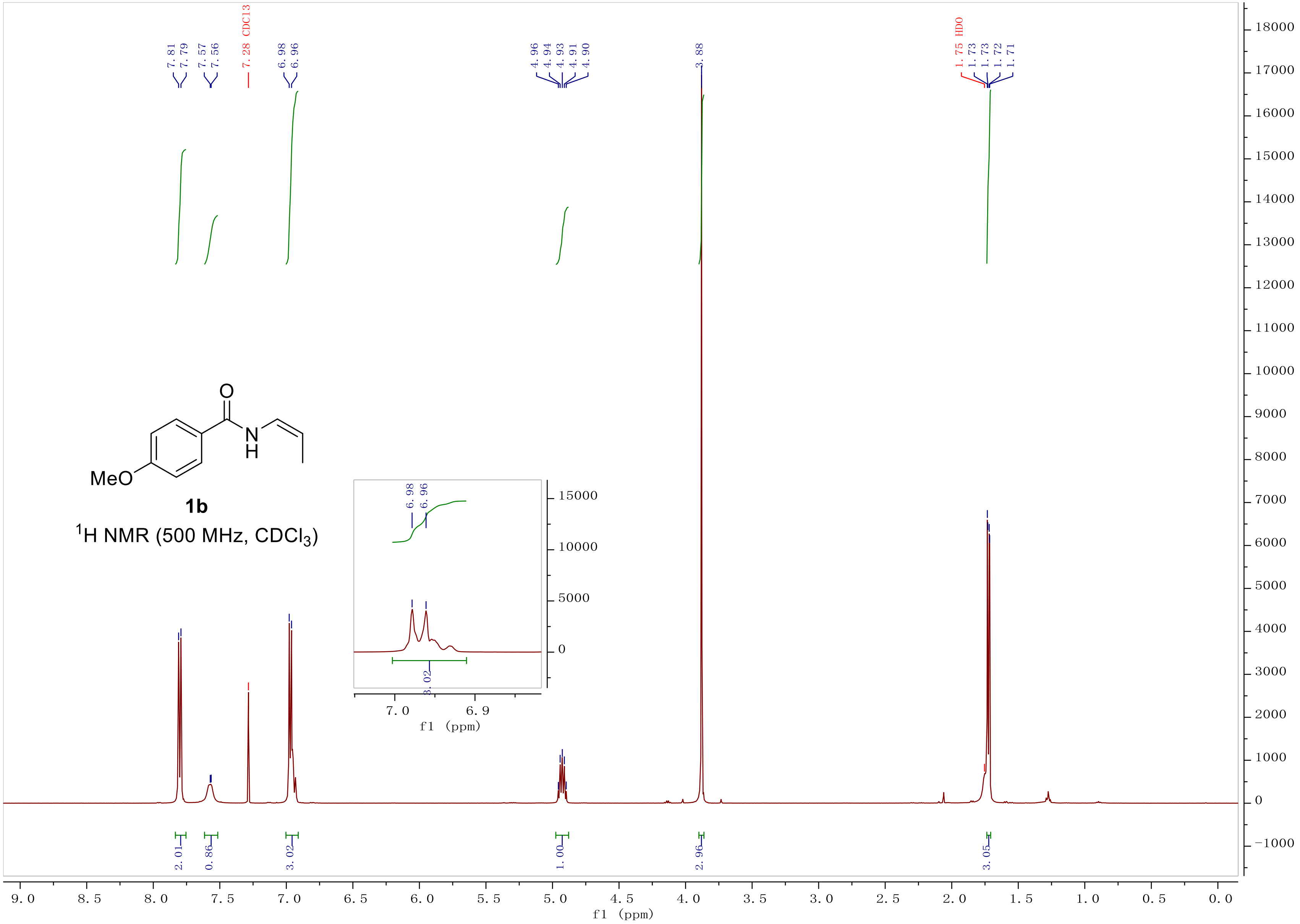
MeO
O
N
H
1b
^{1}H NMR (500 MHz, $CDCl_3$)
7.81
7.79
7.57
7.56
7.28 CDCl3
6.98
6.96
4.96
4.94
4.93
4.91
4.90
3.88
1.75 HDO
1.73
1.73
1.72
1.71
2.01
0.86
3.02
1.00
2.96
3.05
f1 (ppm)

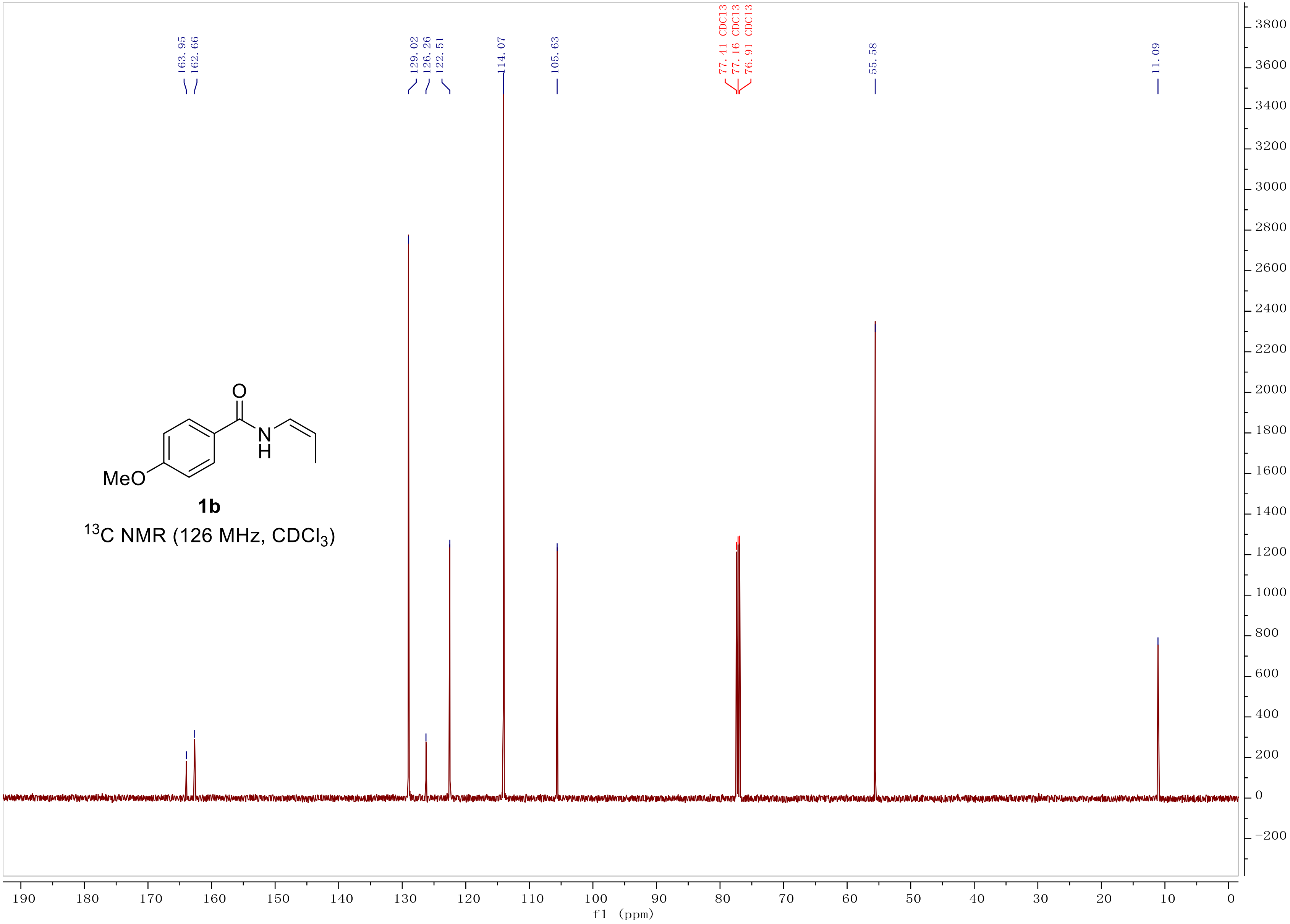

163.95
162.66
129.02
126.26
122.51
114.07
105.63
77.41 CDCl3
77.16 CDCl3
76.91 CDCl3
55.58
11.09
1b
13C NMR (126 MHz, CDCl3)
f1 (ppm)

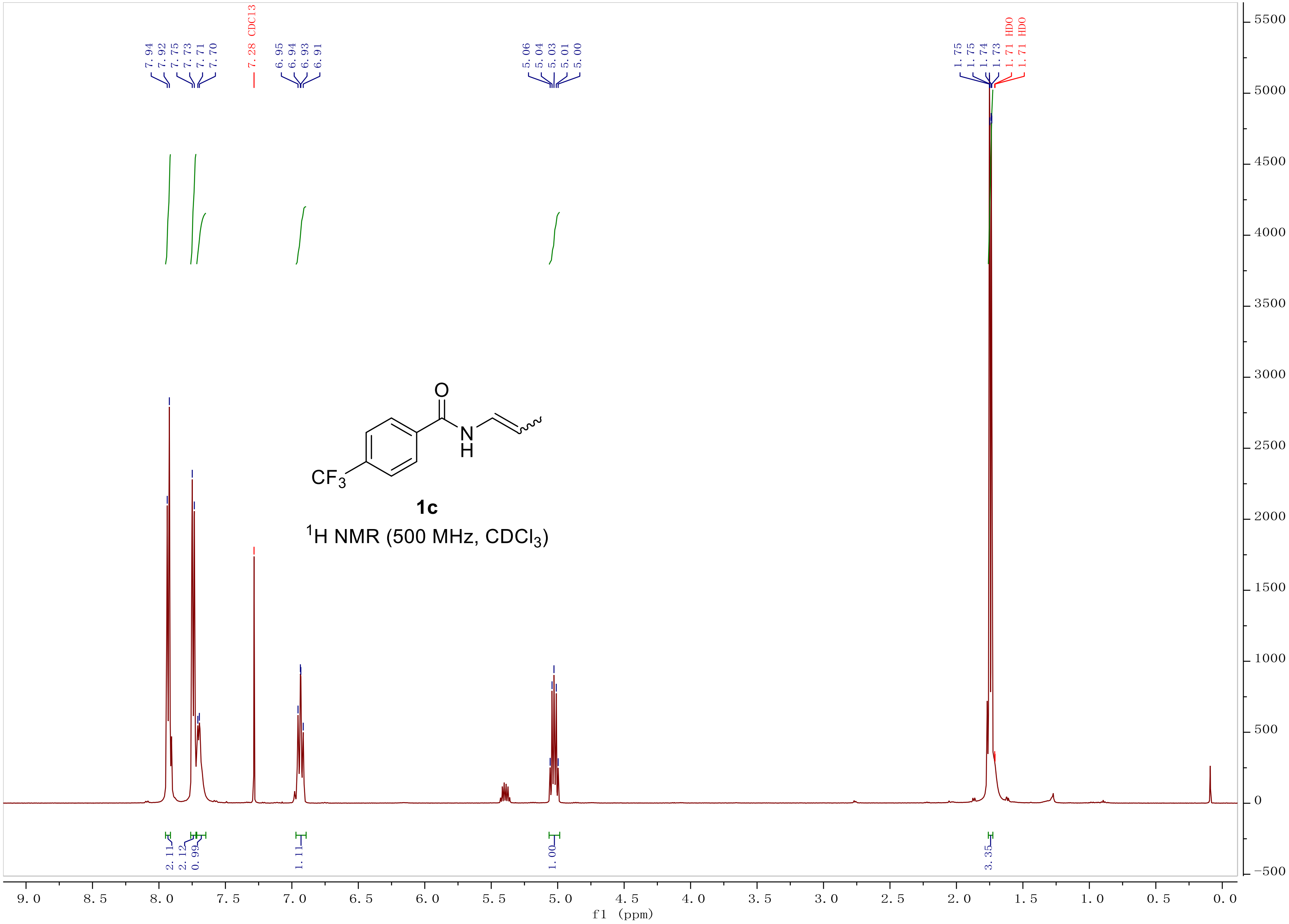
7.94
7.92
7.75
7.73
7.71
7.70
7.28 CDCl3
6.95
6.94
6.93
6.91
5.06
5.04
5.03
5.01
5.00
1.75
1.75
1.74
1.73
1.71 HDO
1.71 HDO
O
N
H
CF3
1c
1H NMR (500 MHz, CDCl3)
2.11
2.12
0.99
1.11
1.00
3.35
f1 (ppm)

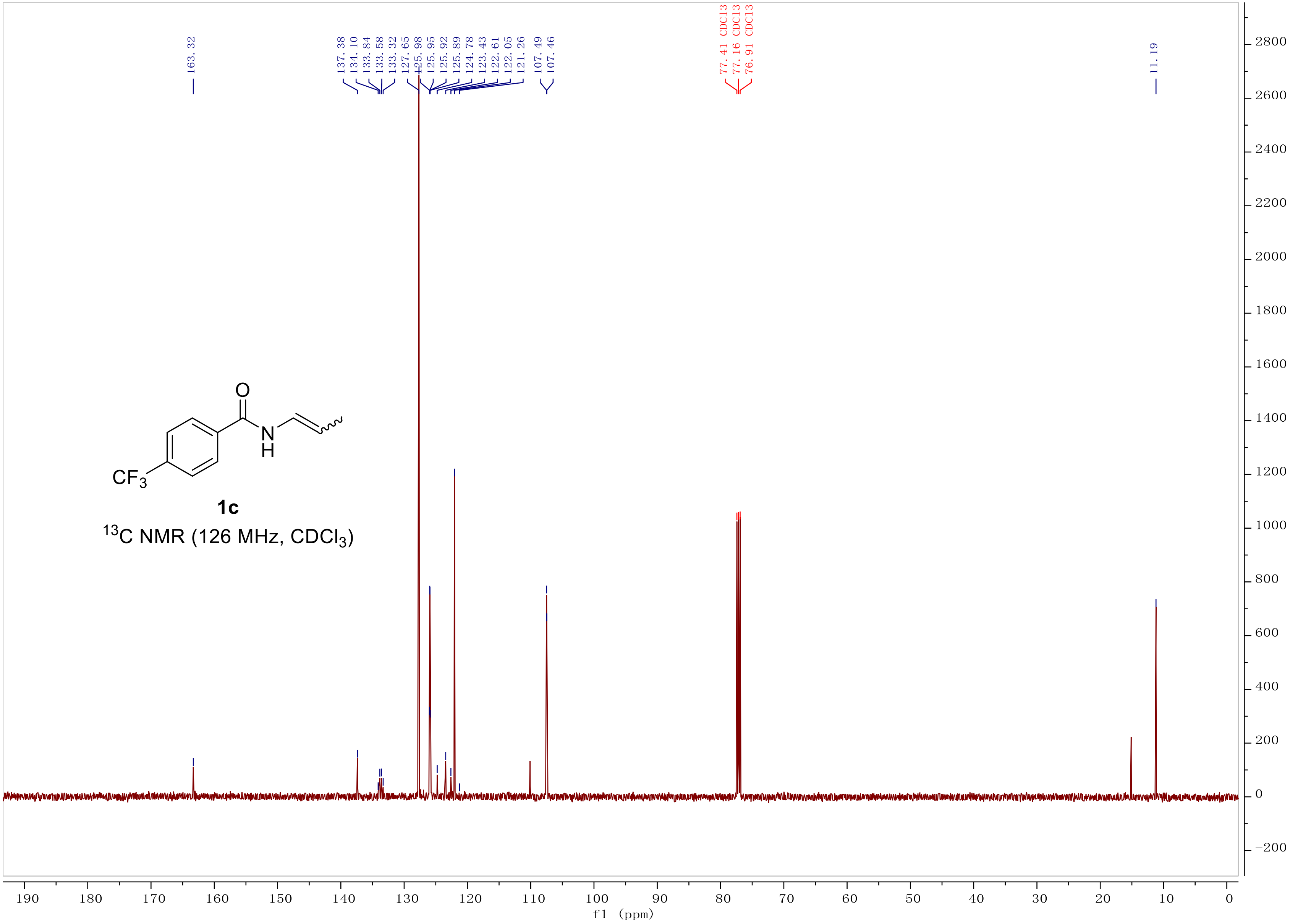
163.32
137.38
134.10
133.84
133.58
133.32
127.65
125.98
125.95
125.92
125.89
124.78
123.43
122.61
122.05
121.26
107.49
107.46
77.41 CDCl3
77.16 CDCl3
76.91 CDCl3
11.19
O
N
H
CF_3
1c
^{13}C NMR (126 MHz, $CDCl_3$)
f1 (ppm)

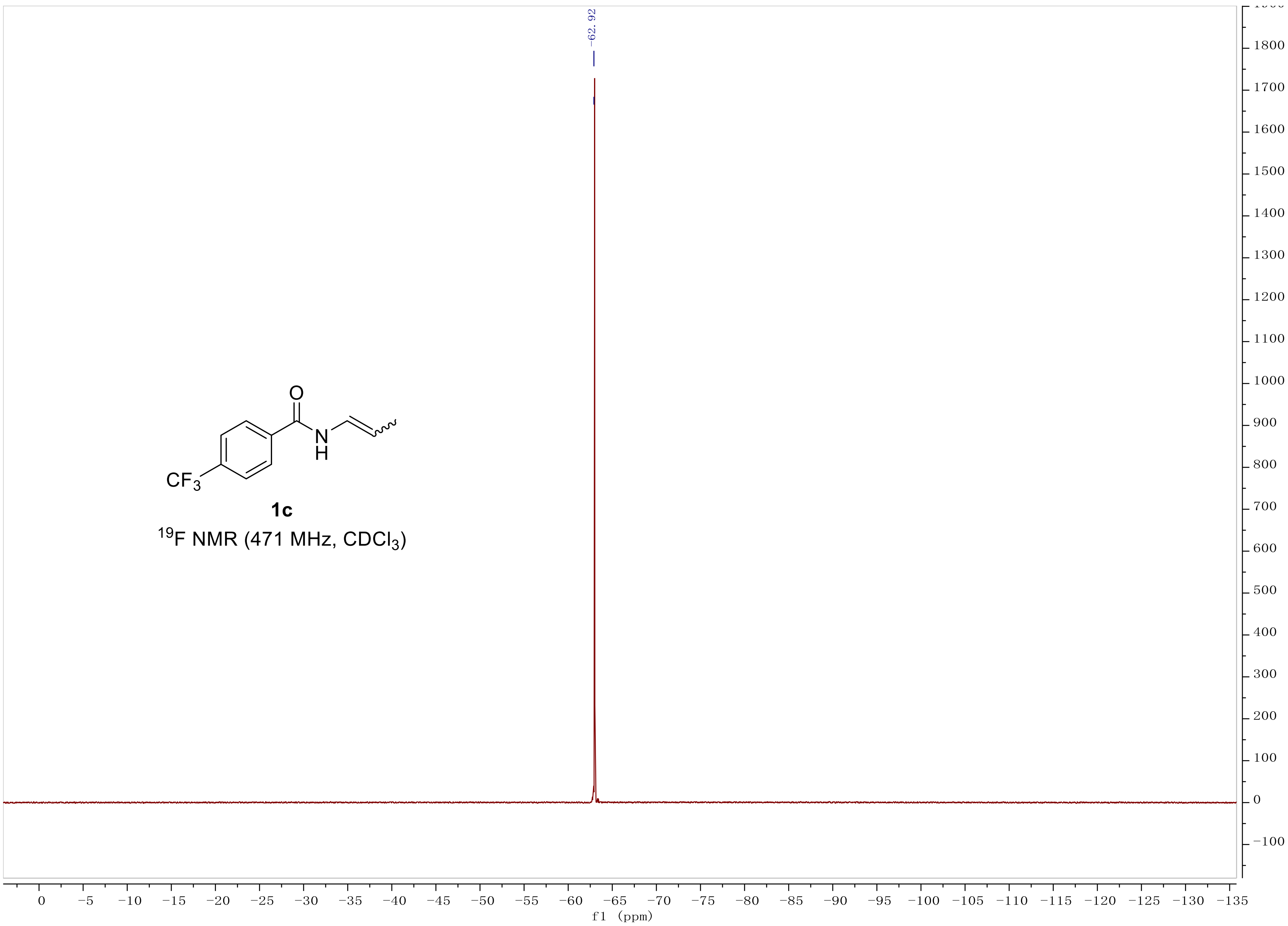

1c

^{19}F NMR (471 MHz, $CDCl_3$)

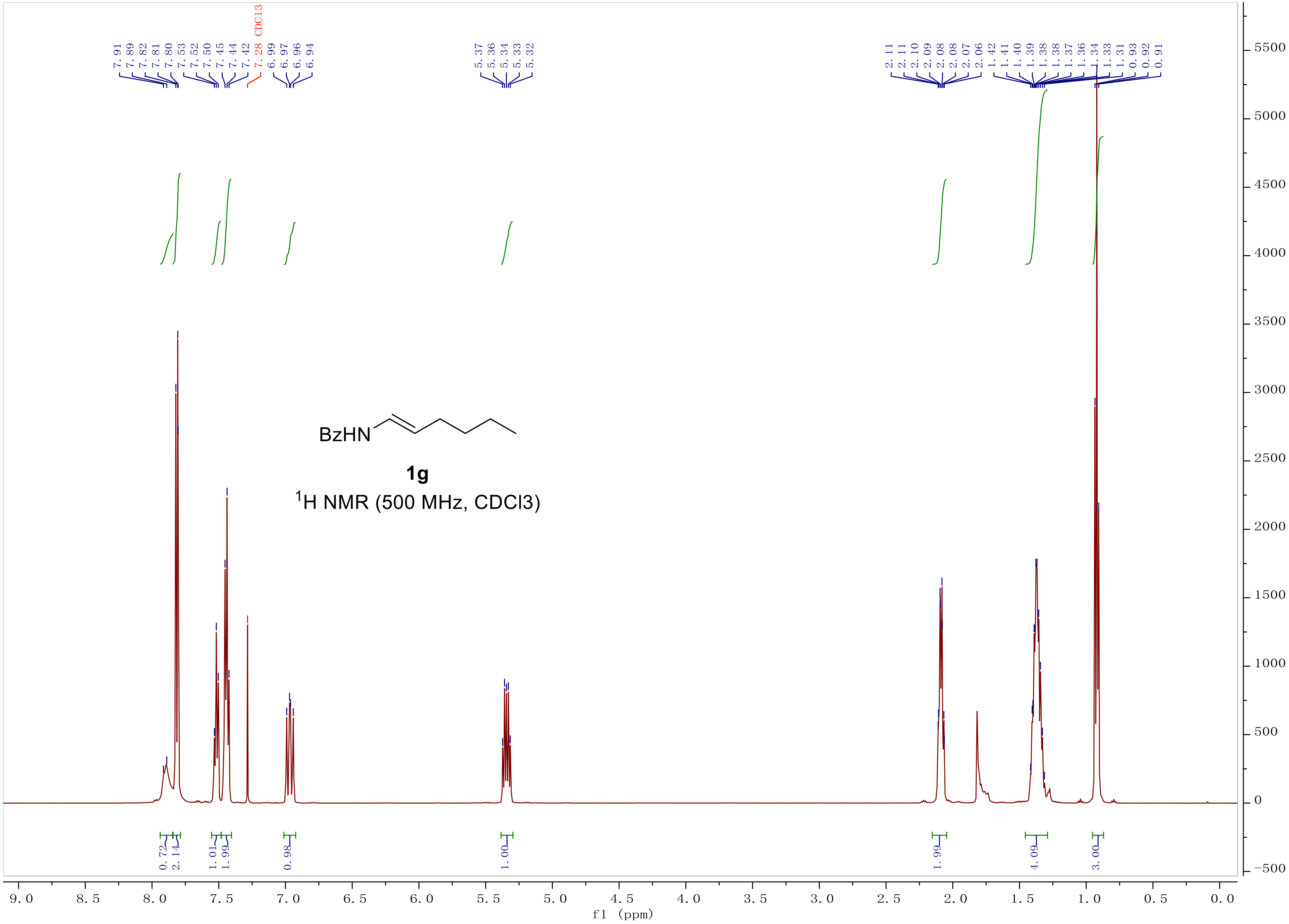
7.91 7.89 7.82 7.81 7.80 7.53 7.52 7.50 7.45 7.44 7.42 7.28 CDCl3 6.99 6.97 6.96 6.94
5.37 5.36 5.34 5.33 5.32
2.11 2.11 2.10 2.09 2.08 2.08 2.07 2.06 1.42 1.41 1.40 1.39 1.38 1.38 1.37 1.36 1.34 1.33 1.31 0.93 0.92 0.91
BzHN
1g
1H NMR (500 MHz, CDCl3)
0.72 2.14 1.01 1.99 0.98 1.00 1.99 4.09 3.00
9.0 8.5 8.0 7.5 7.0 6.5 6.0 5.5 5.0 4.5 4.0 3.5 3.0 2.5 2.0 1.5 1.0 0.5 0.0
f1 (ppm)
5500 5000 4500 4000 3500 3000 2500 2000 1500 1000 500 0 -500

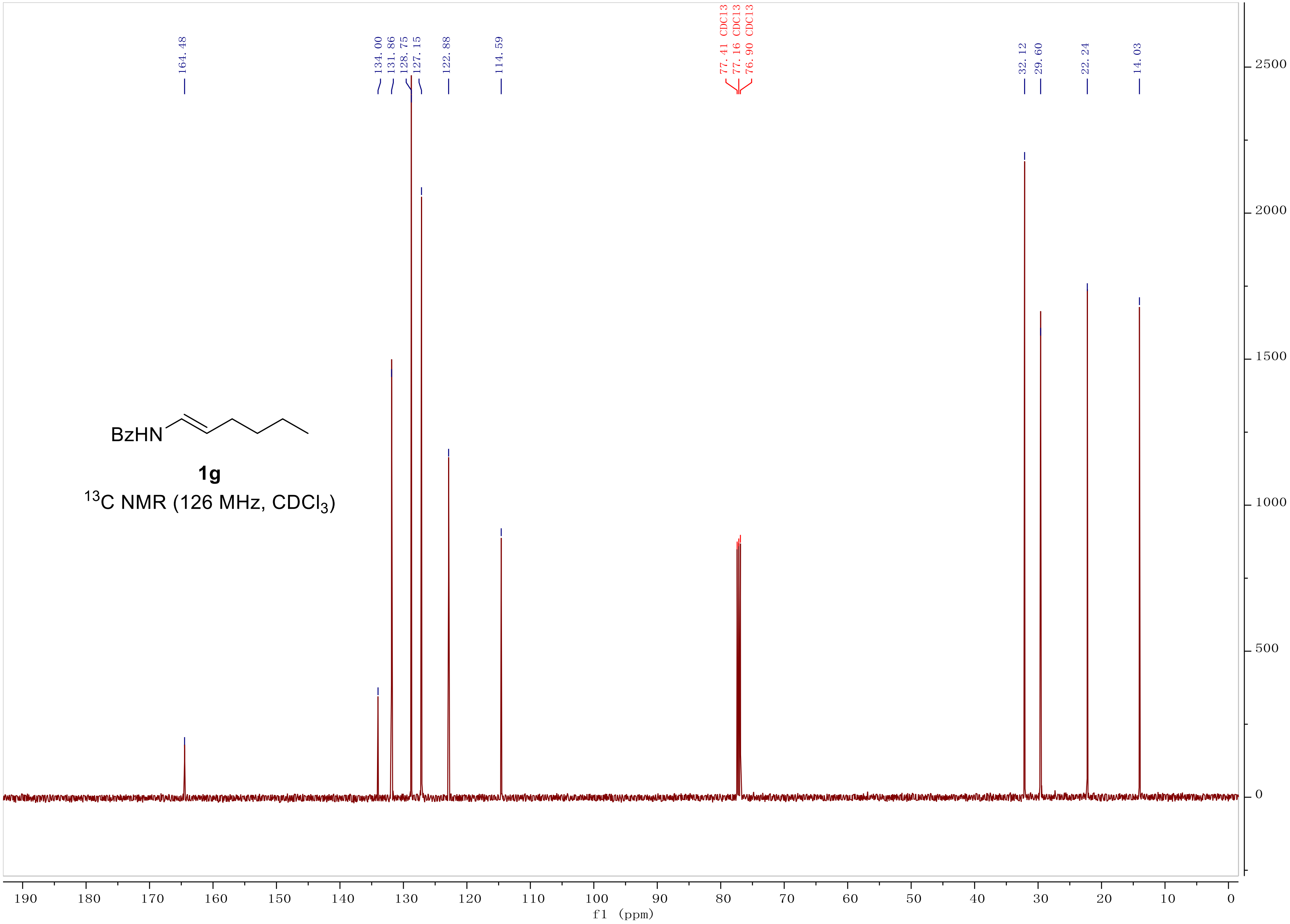
164.48
134.00
131.86
128.75
127.15
122.88
114.59
77.41 CDCl3
77.16 CDCl3
76.90 CDCl3
32.12
29.60
22.24
14.03
BzHN
1g
13C NMR (126 MHz, CDCl3)
f1 (ppm)
190
180
170
160
150
140
130
120
110
100
90
80
70
60
50
40
30
20
10
0
2500
2000
1500
1000
500
0

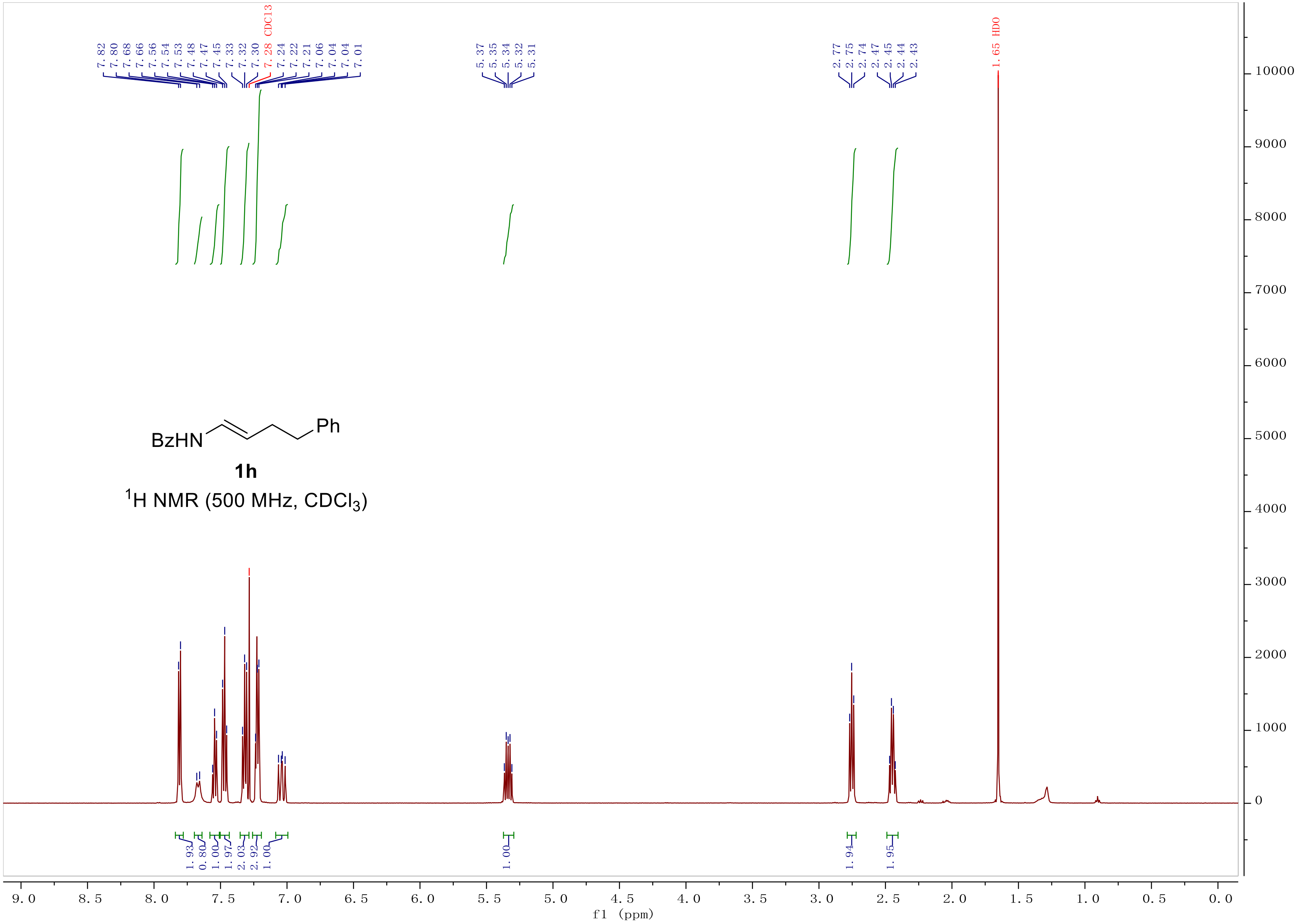
7.82
7.80
7.68
7.66
7.56
7.54
7.53
7.48
7.47
7.45
7.33
7.32
7.30
7.28 CDCl3
7.24
7.22
7.21
7.06
7.04
7.04
7.01
5.37
5.35
5.34
5.32
5.31
2.77
2.75
2.74
2.47
2.45
2.44
2.43
1.65 HDO
BzHN
Ph
1h
1H NMR (500 MHz, CDCl3)
1.93
0.80
1.00
1.97
2.03
2.92
1.00
1.00
1.94
1.95
f1 (ppm)

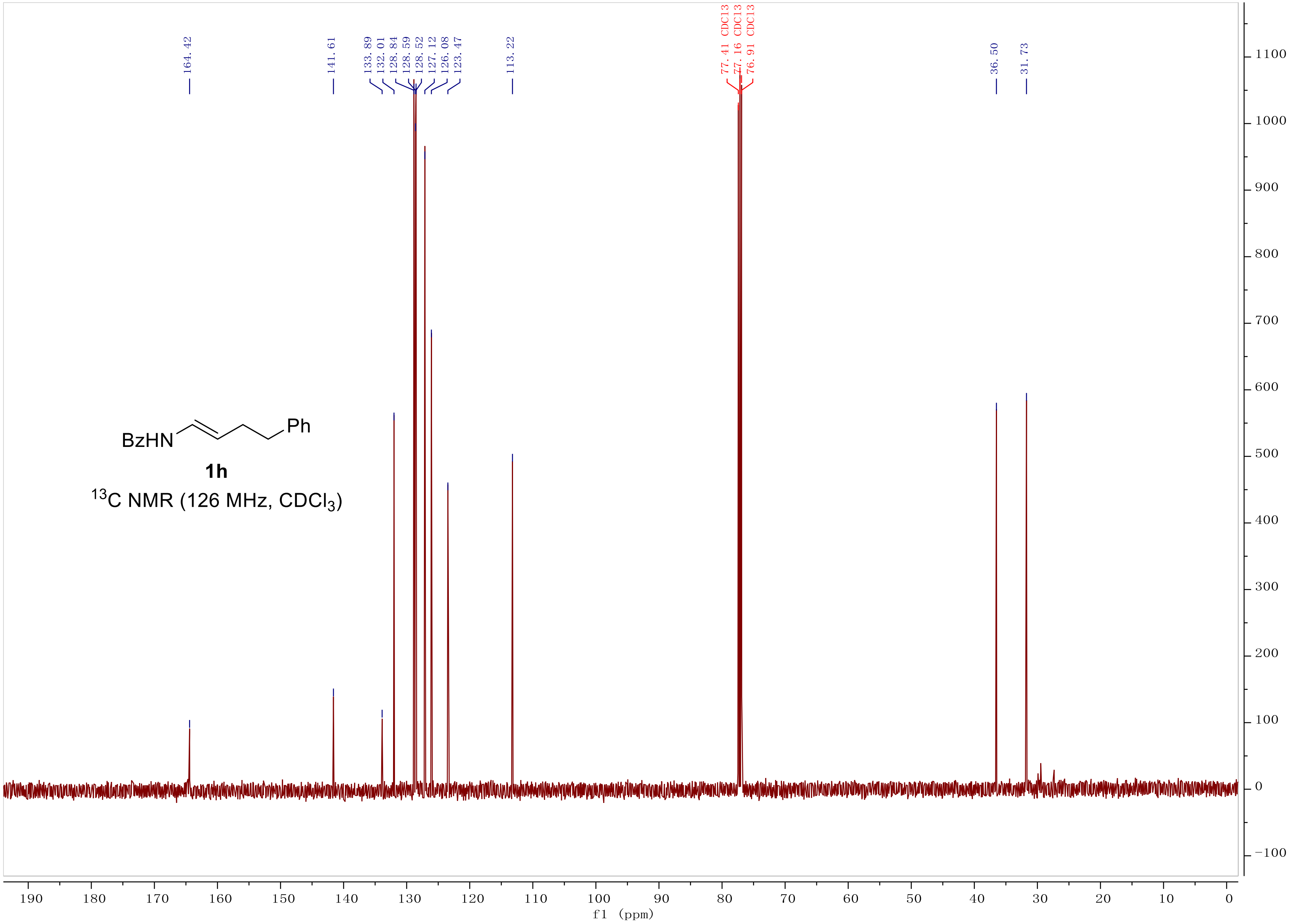
164.42
141.61
133.89
132.01
128.84
128.59
128.52
127.12
126.08
123.47
113.22
77.41 CDCl3
77.16 CDCl3
76.91 CDCl3
36.50
31.73
BzHN
Ph
1h
^{13}C NMR (126 MHz, $CDCl_3$)
f1 (ppm)

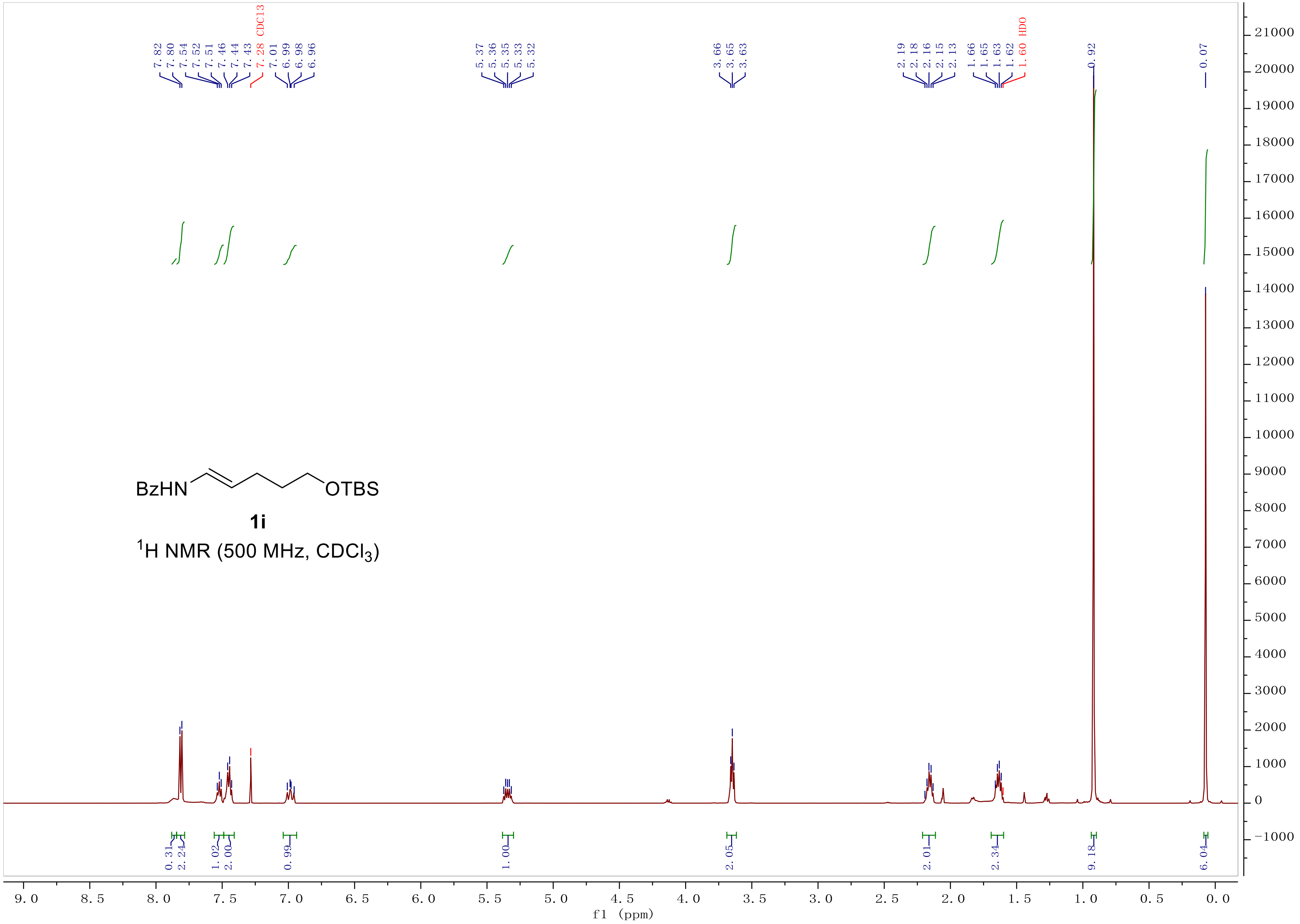

7.82
7.80
7.54
7.52
7.51
7.46
7.44
7.43
7.28 CDCl3
7.01
6.99
6.98
6.96
5.37
5.36
5.35
5.33
5.32
3.66
3.65
3.63
2.19
2.18
2.16
2.15
2.13
1.66
1.65
1.63
1.62
1.60 HDO
0.92
0.07
BzHN
OTBS
1i
^{1}H NMR (500 MHz, $CDCl_3$)
0.31
2.24
1.02
2.00
0.99
1.00
2.05
2.01
2.34
9.18
6.04
f1 (ppm)

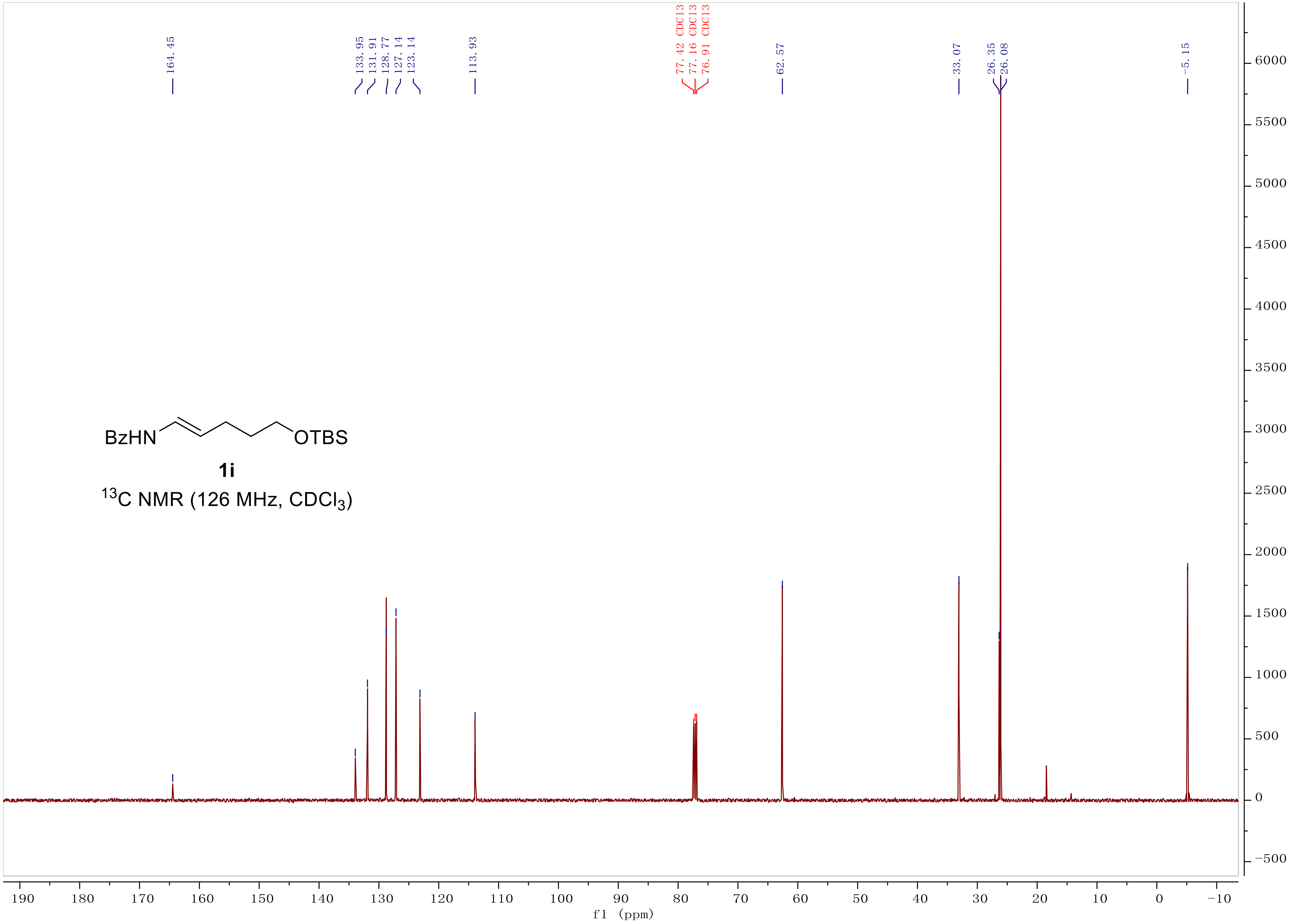

1i

^{13}C NMR (126 MHz, $CDCl_3$)

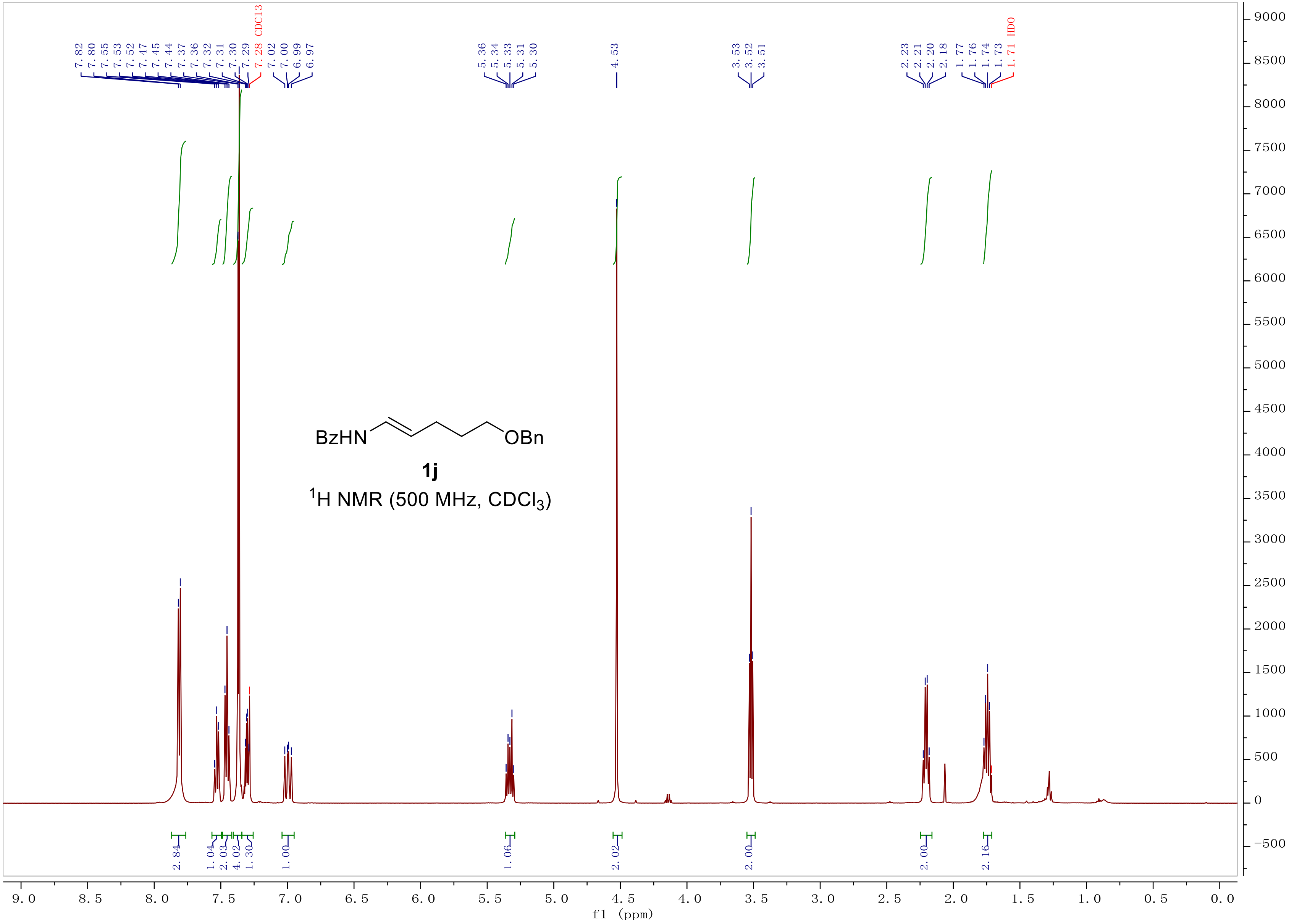

1j

^{1}H NMR (500 MHz, $CDCl_3$)

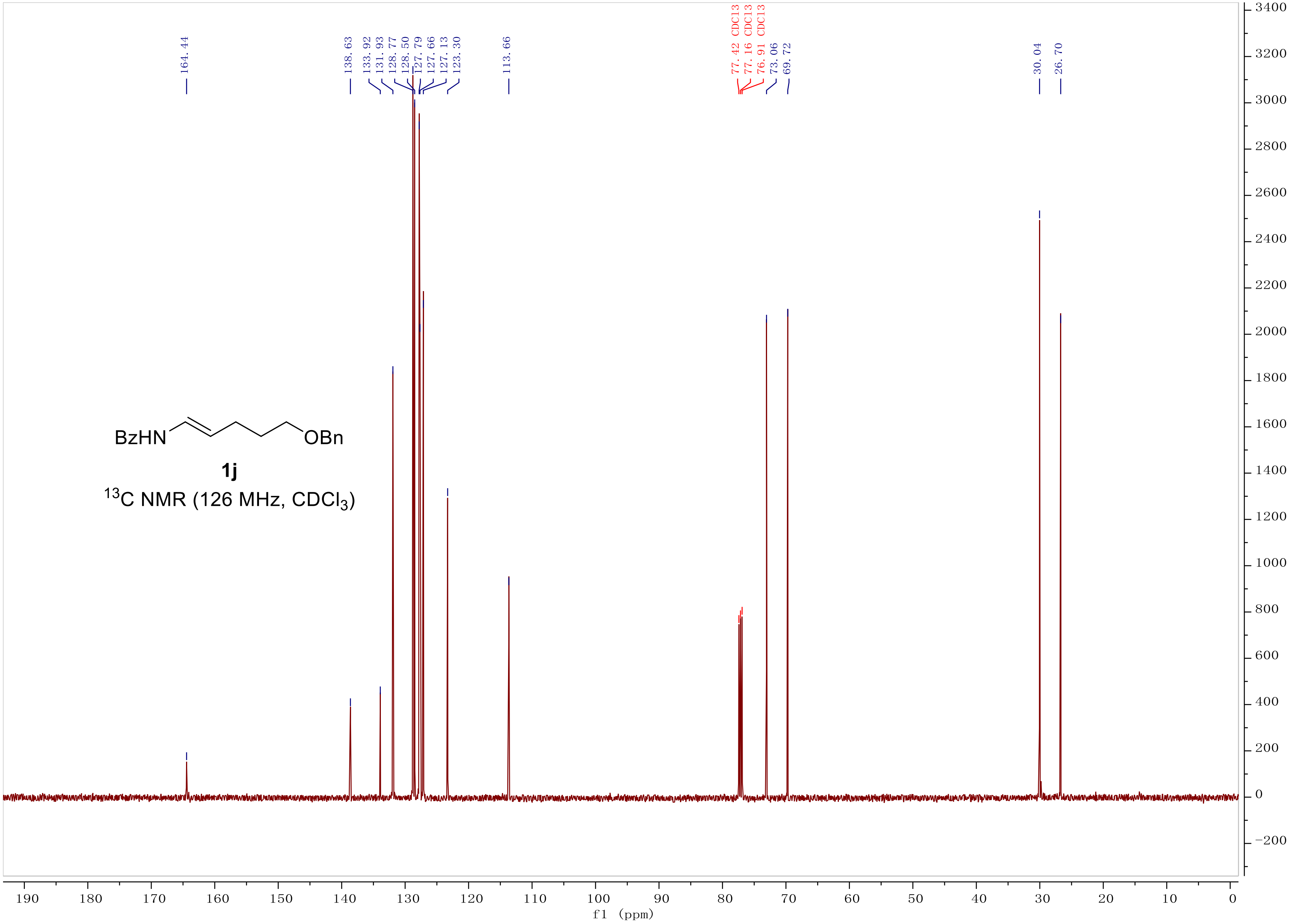
164.44
138.63
133.92
131.93
128.77
128.50
127.79
127.66
127.13
123.30
113.66
77.42 CDCl3
77.16 CDCl3
76.91 CDCl3
73.06
69.72
30.04
26.70
BzHN
OBn
1j
13C NMR (126 MHz, CDCl3)
f1 (ppm)

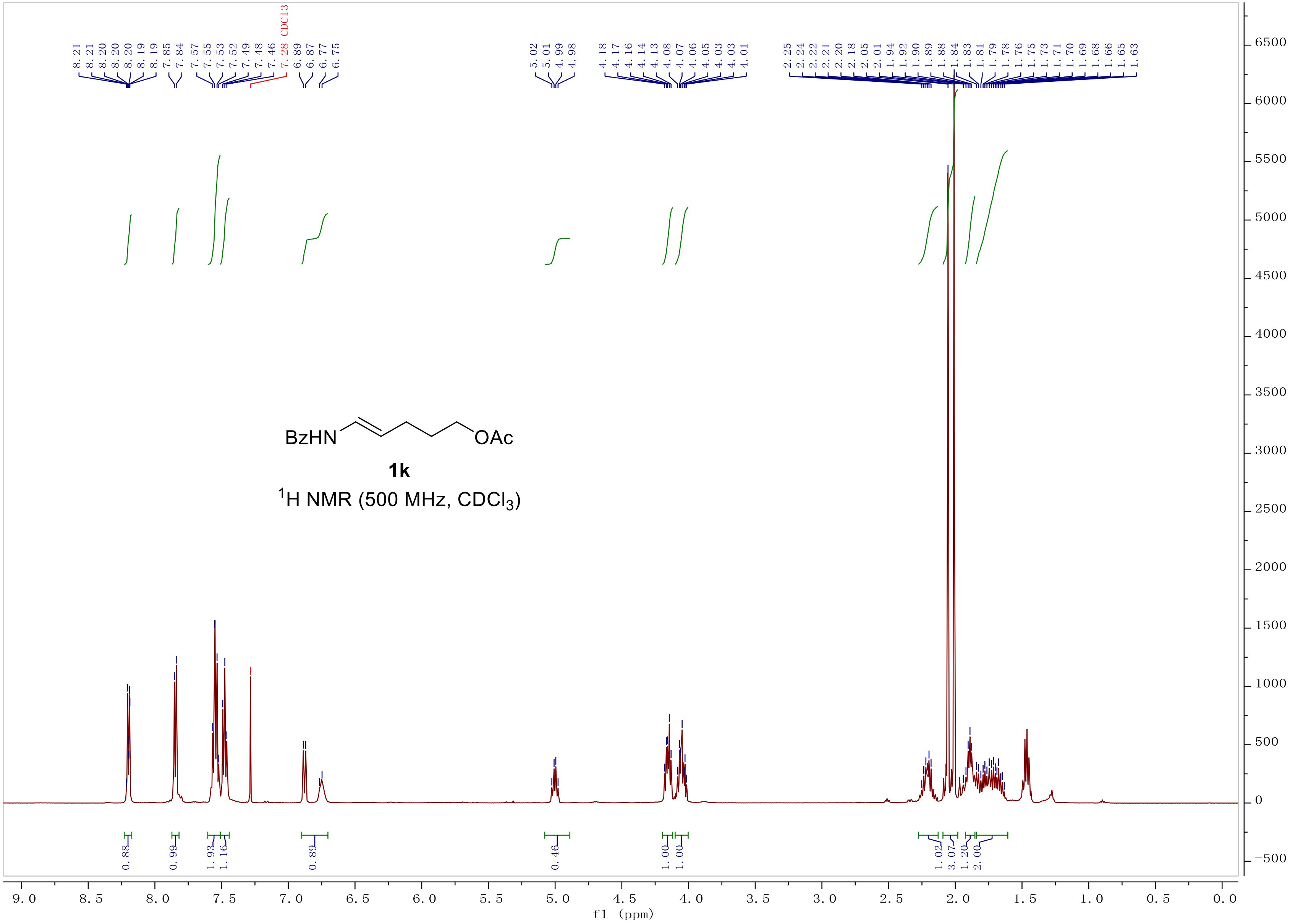

BzHN
OAc
1k
^{1}H NMR (500 MHz, $CDCl_3$)
f1 (ppm)

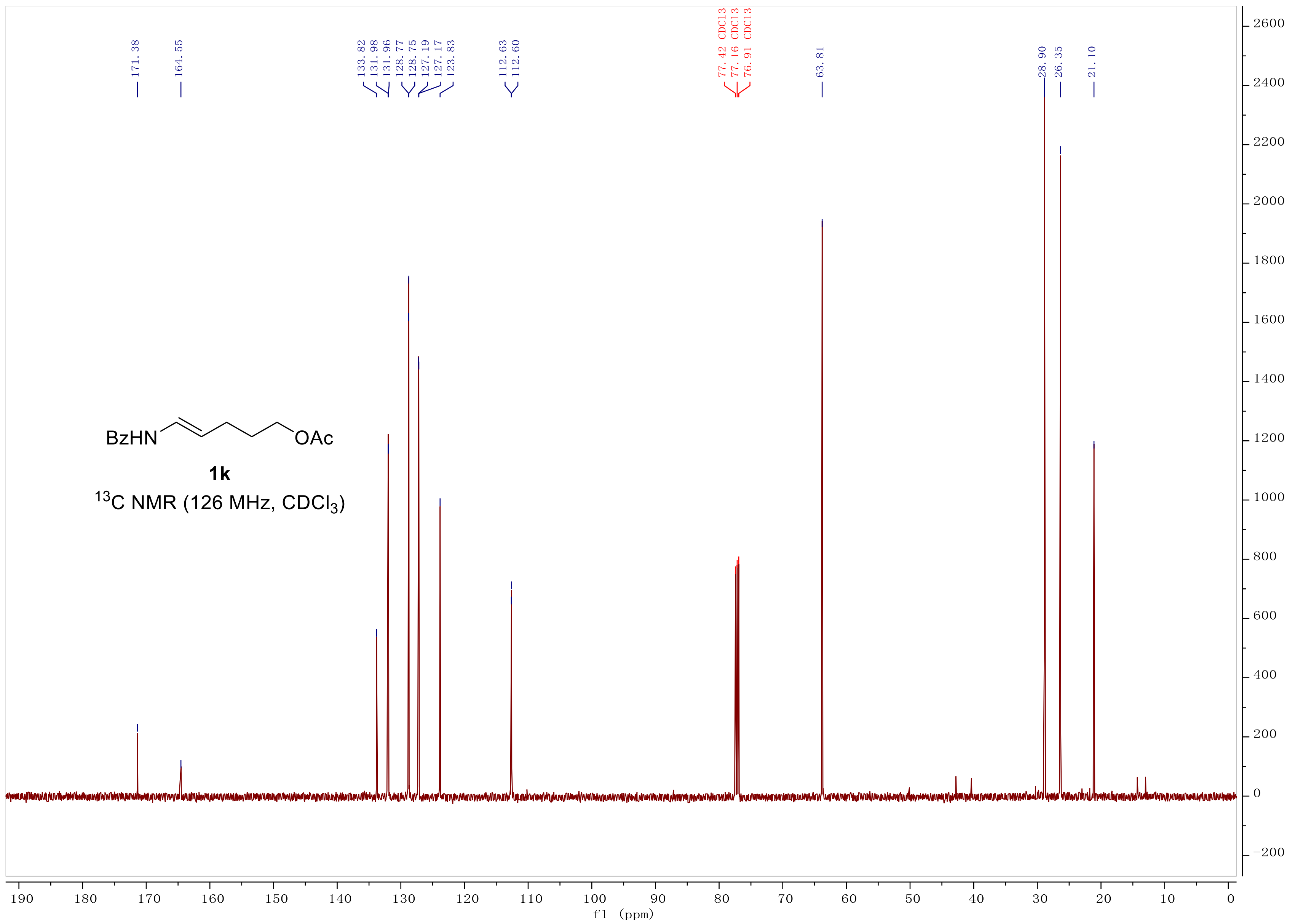
171.38
164.55
133.82
131.98
131.96
128.77
128.75
127.19
127.17
123.83
112.63
112.60
77.42 CDCl3
77.16 CDCl3
76.91 CDCl3
63.81
28.90
26.35
21.10
BzHN
OAc
1k
13C NMR (126 MHz, CDCl3)
f1 (ppm)

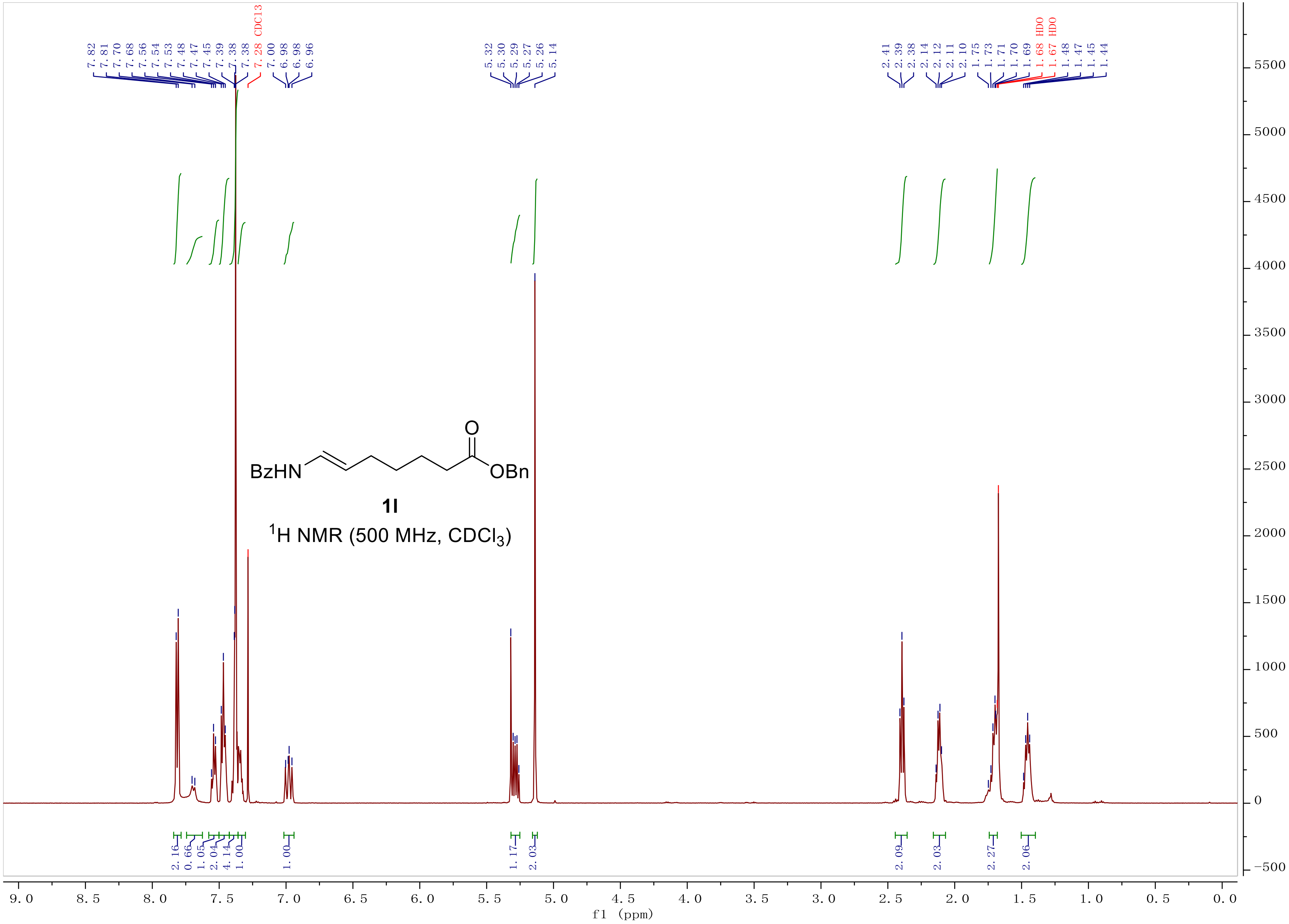
7.82 7.81 7.70 7.68 7.56 7.54 7.53 7.48 7.47 7.45 7.39 7.38 7.38 7.28 CDCl3 7.00 6.98 6.98 6.96
5.32 5.30 5.29 5.27 5.26 5.14
2.41 2.39 2.38 2.14 2.12 2.11 2.10 1.75 1.73 1.71 1.70 1.69 1.68 HDO 1.67 HDO 1.48 1.47 1.45 1.44
BzHN
OBn
O
1l
1H NMR (500 MHz, CDCl3)
2.16 0.66 1.05 2.04 4.14 1.00 1.00 1.17 2.03 2.09 2.03 2.27 2.06
f1 (ppm)

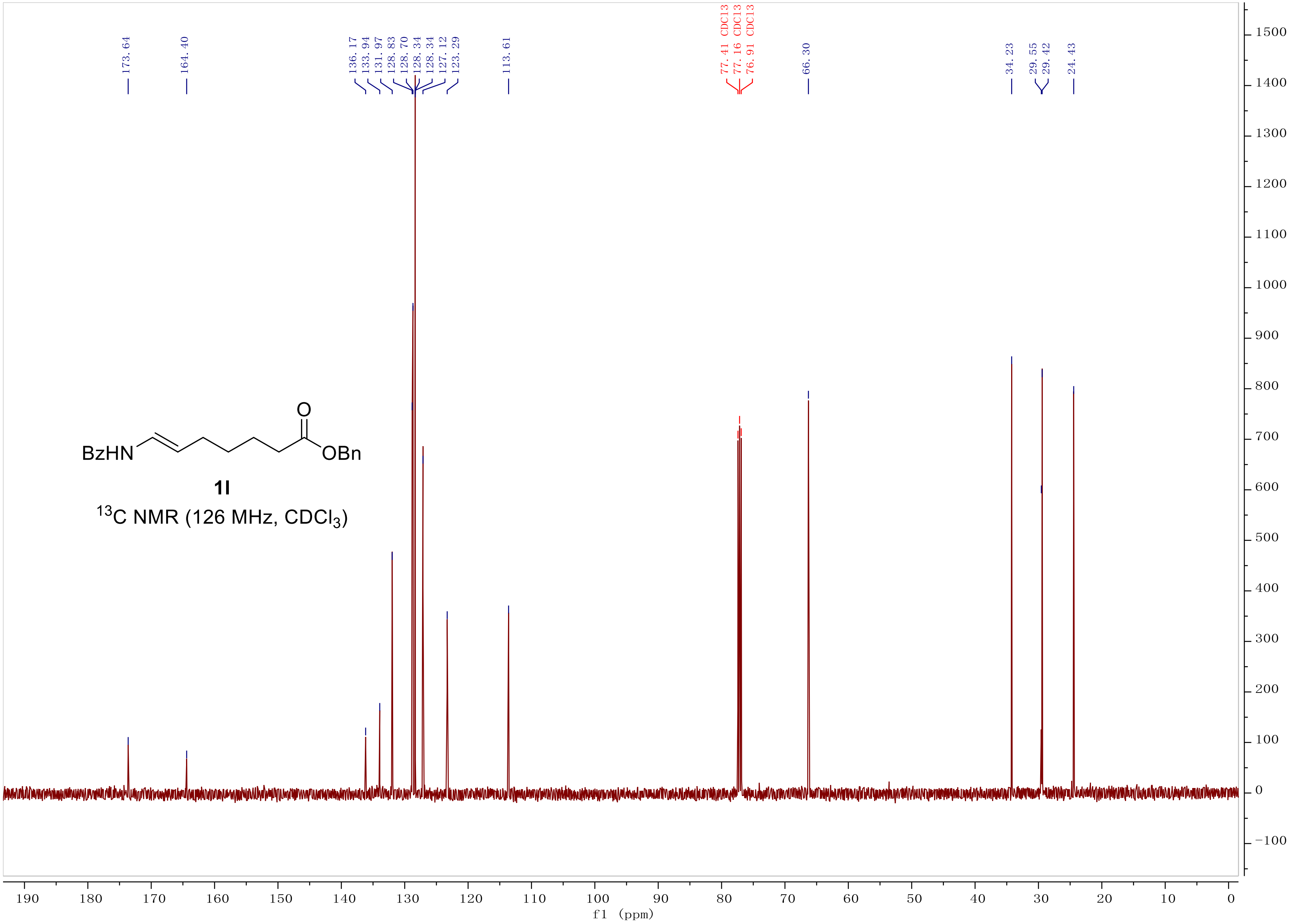

173.64
164.40
136.17
133.94
131.97
128.83
128.70
128.34
128.34
127.12
123.29
113.61
77.41 CDCl3
77.16 CDCl3
76.91 CDCl3
66.30
34.23
29.55
29.42
24.43
BzHN
O
OBn
1l
13C NMR (126 MHz, CDCl3)
f1 (ppm)

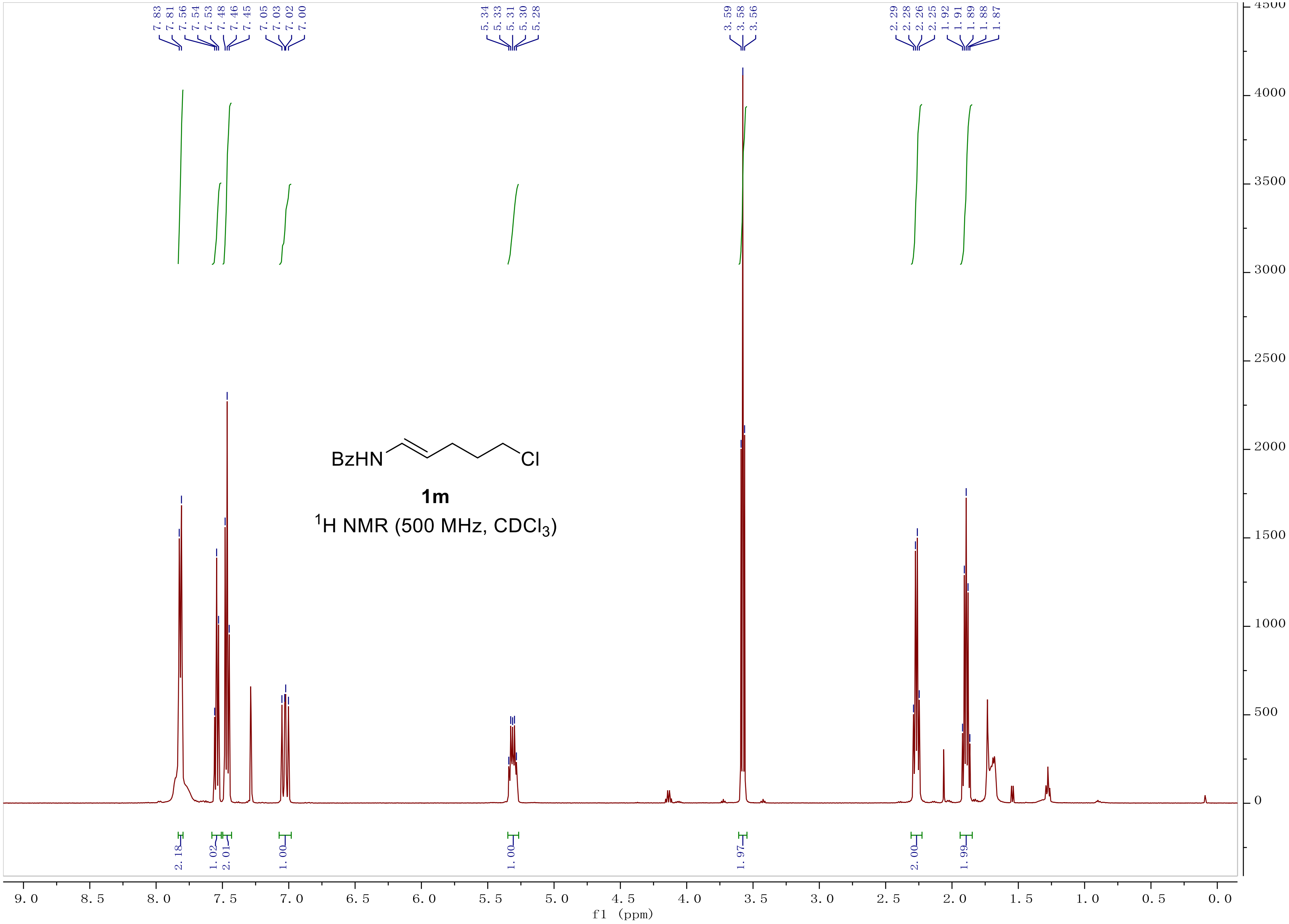
BzHN
Cl
1m
^{1}H NMR (500 MHz, CDCl$_3$)
f1 (ppm)

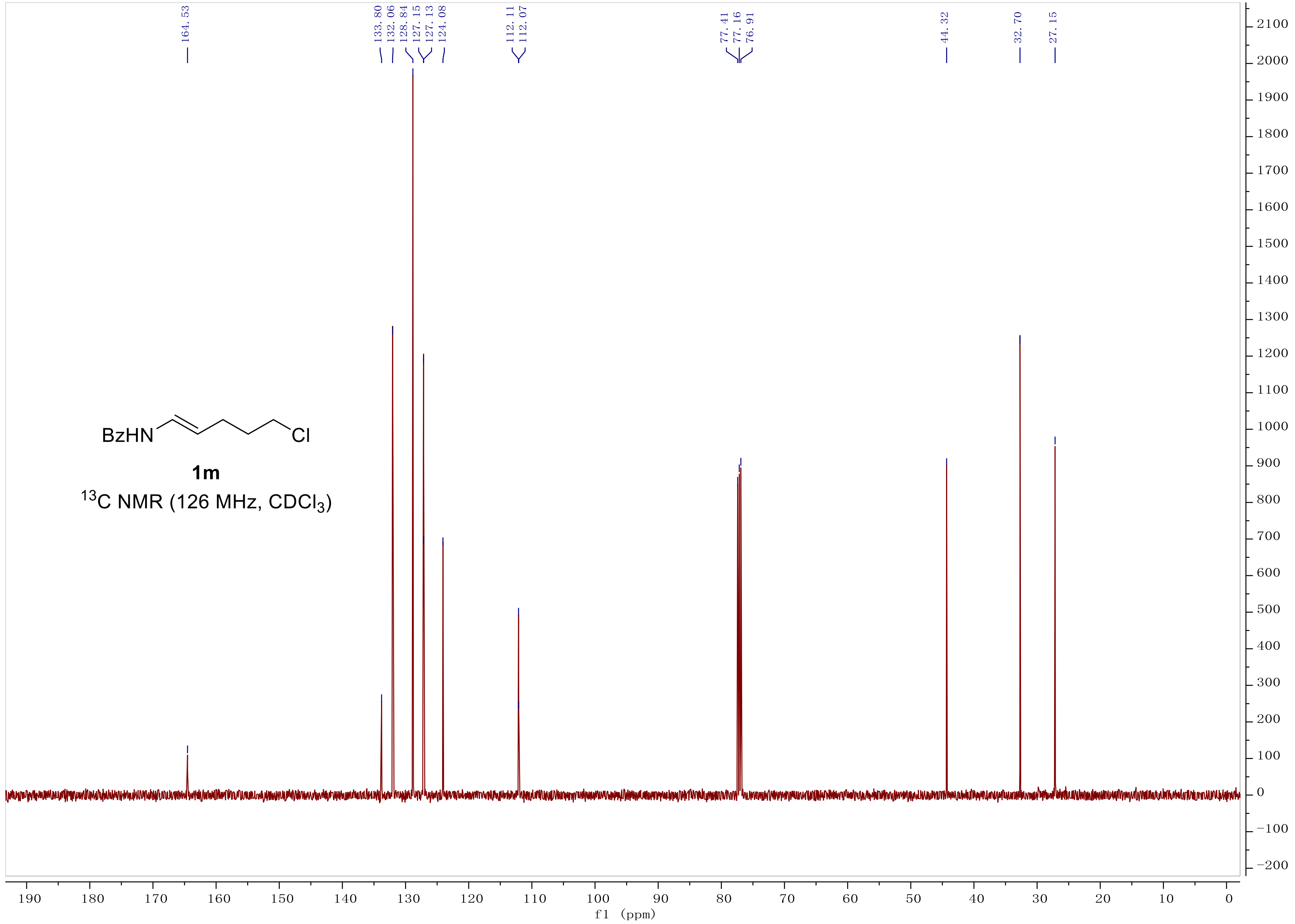

1m

^{13}C NMR (126 MHz, $CDCl_3$)

XI. Spectroscopic Data (HPLC Trace)

Data File E:\DATA\20200921\LC 2020-09-21 09-03-41\OnlineEdited--053.D
Sample Name: YH-17-182-RAC

===

Acq. Operator : SYSTEM Seq. Line : 53
Acq. Instrument : HPLC1260 Location : P1-B1
Injection Date : 9/22/2020 11:02:00 AM Inj : 1
Inj Volume : 3.000 µl
Different Inj Volume from Sample Entry! Actual Inj Volume : 1.000 µl
Acq. Method : E:\DATA\20200921\LC 2020-09-21 09-03-41\10EtOH_35_10_3.M
Last changed : 9/21/2020 9:22:41 PM by SYSTEM
Analysis Method : E:\DATA\20200921\LC 2020-09-21 09-03-41\10EtOH_35_10_3.M (Sequence Method)
Last changed : 9/22/2020 1:58:15 PM by SYSTEM
(modified after loading)
Additional Info : Peak(s) manually integrated

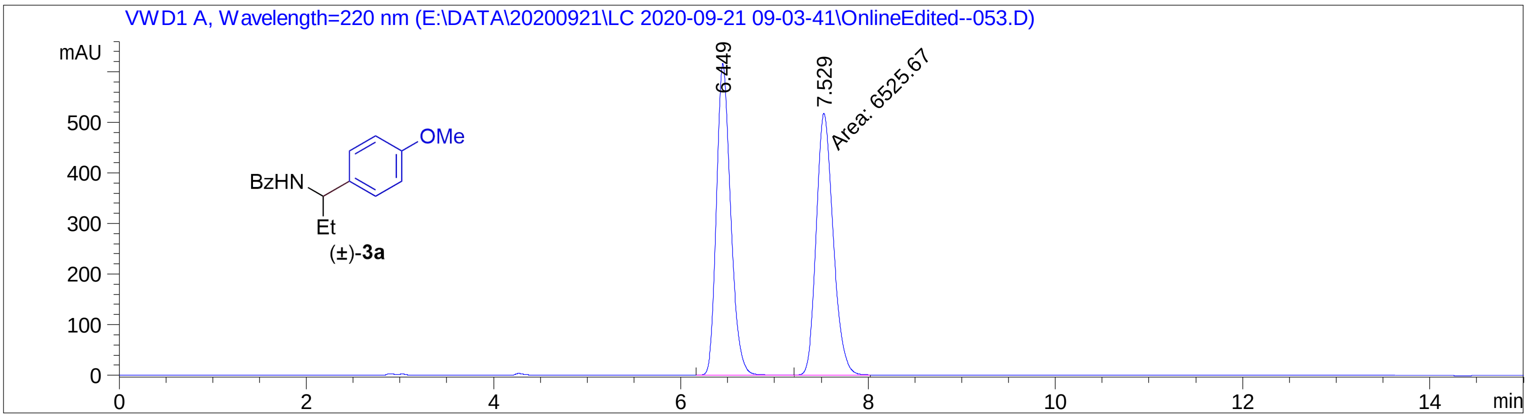

===

Area Percent Report

===

Sorted By : Signal
Multiplier : 1.0000
Dilution : 1.0000
Do not use Multiplier & Dilution Factor with ISTDs

Signal 1: VWD1 A, Wavelength=220 nm

Peak #	RetTime [min]	Type	Width [min]	Area [mAU*s]	Height [mAU]	Area %
1	6.449	BB	0.1623	6512.91113	616.05304	49.9511
2	7.529	MF	0.2102	6525.67432	517.46857	50.0489

Totals : 1.30386e4 1133.52161

===

*** End of Report ***

Data File E:\DATA\20200921\LC 2020-09-21 09-03-41\OnlineEdited--054.D
Sample Name: YH-17-182-EE

===

Acq. Operator : SYSTEM Seq. Line : 54
Acq. Instrument : HPLC1260 Location : P1-B2
Injection Date : 9/22/2020 11:37:44 AM Inj : 1
Inj Volume : 3.000 µl
Different Inj Volume from Sample Entry! Actual Inj Volume : 2.000 µl
Acq. Method : E:\DATA\20200921\LC 2020-09-21 09-03-41\10EtOH_35_10_3.M
Last changed : 9/21/2020 9:22:41 PM by SYSTEM
Analysis Method : E:\DATA\20200921\LC 2020-09-21 09-03-41\10EtOH_35_10_3.M (Sequence Method)
Last changed : 9/22/2020 1:57:44 PM by SYSTEM
(modified after loading)
Additional Info : Peak(s) manually integrated

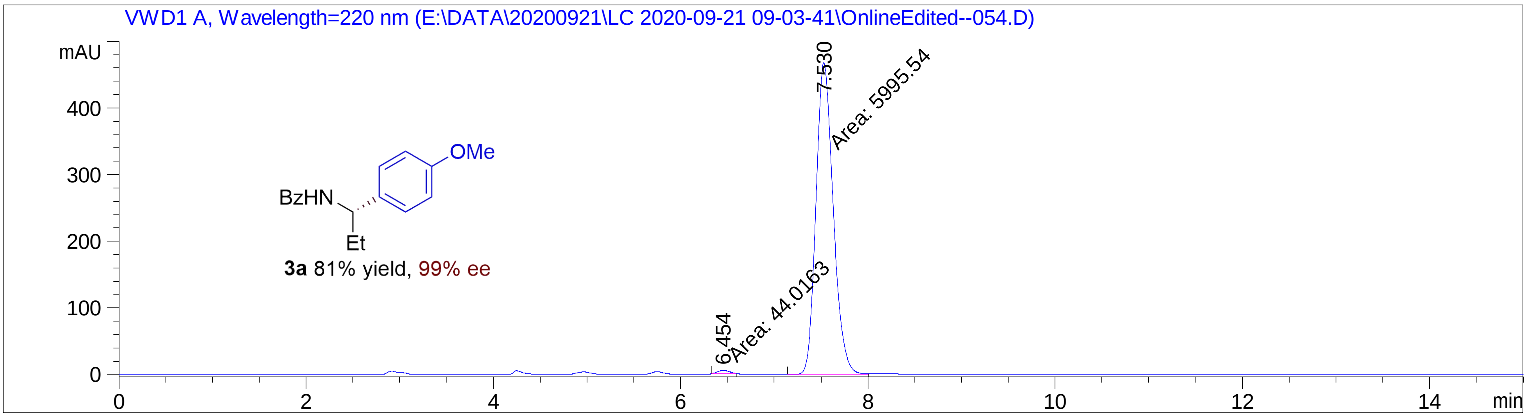

===

Area Percent Report

===

Sorted By : Signal
Multiplier : 1.0000
Dilution : 1.0000
Do not use Multiplier & Dilution Factor with ISTDs

Signal 1: VWD1 A, Wavelength=220 nm

Peak #	RetTime [min]	Type	Width [min]	Area [mAU*s]	Height [mAU]	Area %
1	6.454	MM	0.1428	44.01631	5.13735	0.7288
2	7.530	MF	0.2137	5995.54150	467.51907	99.2712

Totals : 6039.55781 472.65643

===

*** End of Report ***

===

Acq. Operator : SYSTEM Seq. Line : 2
Acq. Instrument : HPLC1260 Location : P1-B1
Injection Date : 10/7/2020 12:52:46 AM Inj : 1
Inj Volume : 3.000 µl
Different Inj Volume from Sample Entry! Actual Inj Volume : 1.000 µl
Acq. Method : E:\DATA\20201003\LC 2020-10-07 00-19-54\20IPA_30_10_2.M
Last changed : 10/7/2020 12:19:54 AM by SYSTEM
Analysis Method : E:\DATA\20201003\LC 2020-10-07 00-19-54\20IPA_30_10_2.M (Sequence Method)
Last changed : 10/7/2020 8:13:51 AM by SYSTEM
(modified after loading)
Additional Info : Peak(s) manually integrated

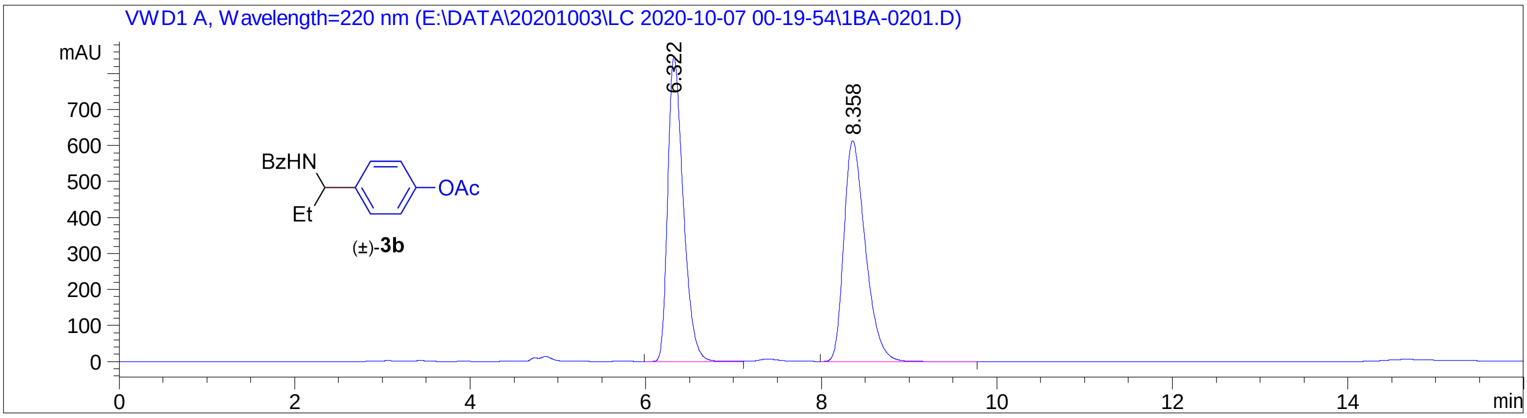

===

Area Percent Report

===

Sorted By : Signal
Multiplier : 1.0000
Dilution : 1.0000
Do not use Multiplier & Dilution Factor with ISTDs

Signal 1: VWD1 A, Wavelength=220 nm

Peak #	RetTime [min]	Type	Width [min]	Area [mAU*s]	Height [mAU]	Area %
1	6.322	BV	0.1876	1.04230e4	847.06049	49.9286
2	8.358	VB	0.2598	1.04529e4	613.51941	50.0714

Totals : 2.08759e4 1460.57990

===

*** End of Report ***

Data File E:\DATA\20201003\LC 2020-10-07 00-19-54\1BB-0301.D
Sample Name: YH-18-29-EE

===

Acq. Operator : SYSTEM Seq. Line : 3
Acq. Instrument : HPLC1260 Location : P1-B2
Injection Date : 10/7/2020 1:23:31 AM Inj : 1
Inj Volume : 3.000 µl
Different Inj Volume from Sample Entry! Actual Inj Volume : 2.000 µl
Acq. Method : E:\DATA\20201003\LC 2020-10-07 00-19-54\20IPA_30_10_2.M
Last changed : 10/7/2020 12:19:54 AM by SYSTEM
Analysis Method : E:\DATA\20201003\LC 2020-10-07 00-19-54\20IPA_30_10_2.M (Sequence Method)
Last changed : 10/7/2020 8:13:51 AM by SYSTEM
(modified after loading)
Additional Info : Peak(s) manually integrated

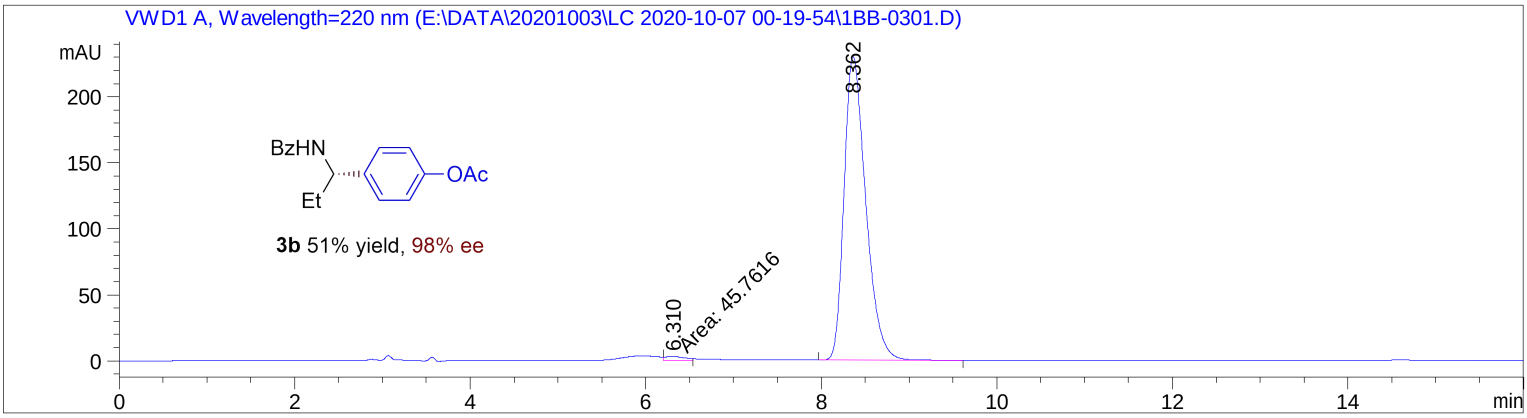

===
Area Percent Report
===

Sorted By : Signal
Multiplier : 1.0000
Dilution : 1.0000
Do not use Multiplier & Dilution Factor with ISTDs

Signal 1: VWD1 A, Wavelength=220 nm

Peak #	RetTime [min]	Type	Width [min]	Area [mAU*s]	Height [mAU]	Area %
1	6.310	MF	0.2637	45.76163	2.89189	1.1468
2	8.362	BB	0.2622	3944.70435	229.87079	98.8532

Totals : 3990.46598 232.76268

===
*** End of Report ***

Data File E:\DATA\20201003\LC 2020-10-06 07-01-40\OnlineEdited--019.D
Sample Name: YH-18-23-RAC

===

Acq. Operator : SYSTEM Seq. Line : 19
Acq. Instrument : HPLC1260 Location : P1-C5
Injection Date : 10/6/2020 4:15:51 PM Inj : 1
Inj Volume : 3.000 µl
Different Inj Volume from Sample Entry! Actual Inj Volume : 1.000 µl
Acq. Method : E:\DATA\20201003\LC 2020-10-06 07-01-40\20IPA_30_10_2.M
Last changed : 10/6/2020 11:07:22 AM by SYSTEM
Analysis Method : E:\DATA\20201003\LC 2020-10-06 07-01-40\20IPA_30_10_2.M (Sequence Method)
Last changed : 10/6/2020 5:39:05 PM by SYSTEM
(modified after loading)
Additional Info : Peak(s) manually integrated

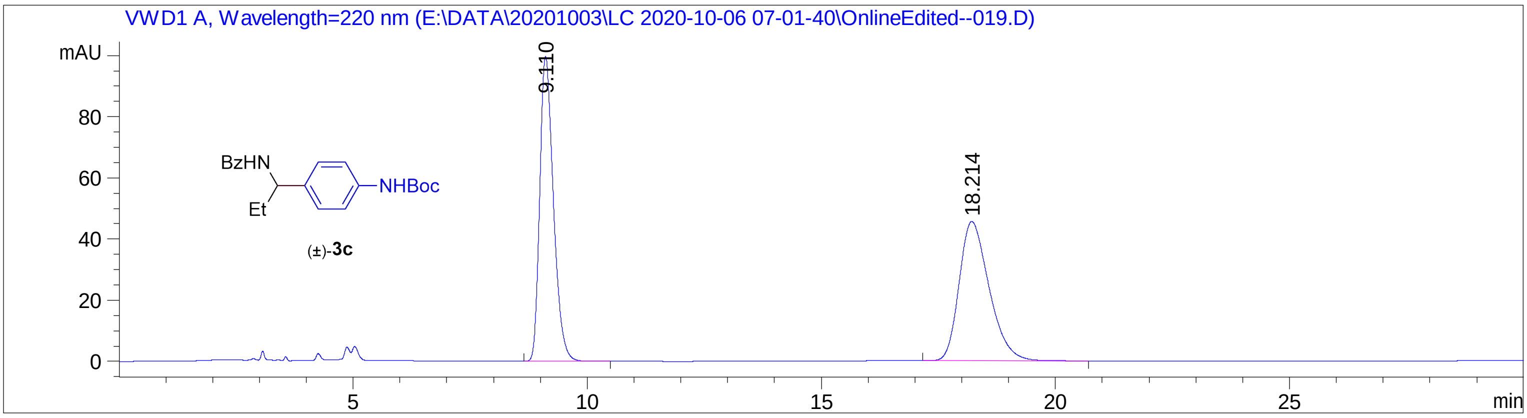

===

Area Percent Report

===

Sorted By : Signal
Multiplier : 1.0000
Dilution : 1.0000
Do not use Multiplier & Dilution Factor with ISTDs

Signal 1: VWD1 A, Wavelength=220 nm

Peak #	RetTime [min]	Type	Width [min]	Area [mAU*s]	Height [mAU]	Area %
1	9.110	BB	0.3111	2024.86633	99.61646	49.9601
2	18.214	BB	0.6826	2028.10364	45.53419	50.0399

Totals : 4052.96997 145.15065

===

*** End of Report ***

Data File E:\DATA\20201003\LC 2020-10-06 07-01-40\OnlineEdited--020.D
Sample Name: YH-18-23-EE

===

Acq. Operator : SYSTEM Seq. Line : 20
Acq. Instrument : HPLC1260 Location : P1-C6
Injection Date : 10/6/2020 4:46:37 PM Inj : 1
Inj Volume : 3.000 µl
Different Inj Volume from Sample Entry! Actual Inj Volume : 2.000 µl
Acq. Method : E:\DATA\20201003\LC 2020-10-06 07-01-40\20IPA_30_10_2.M
Last changed : 10/6/2020 11:07:22 AM by SYSTEM
Analysis Method : E:\DATA\20201003\LC 2020-10-06 07-01-40\20IPA_30_10_2.M (Sequence Method)
Last changed : 10/6/2020 5:39:05 PM by SYSTEM
(modified after loading)
Additional Info : Peak(s) manually integrated

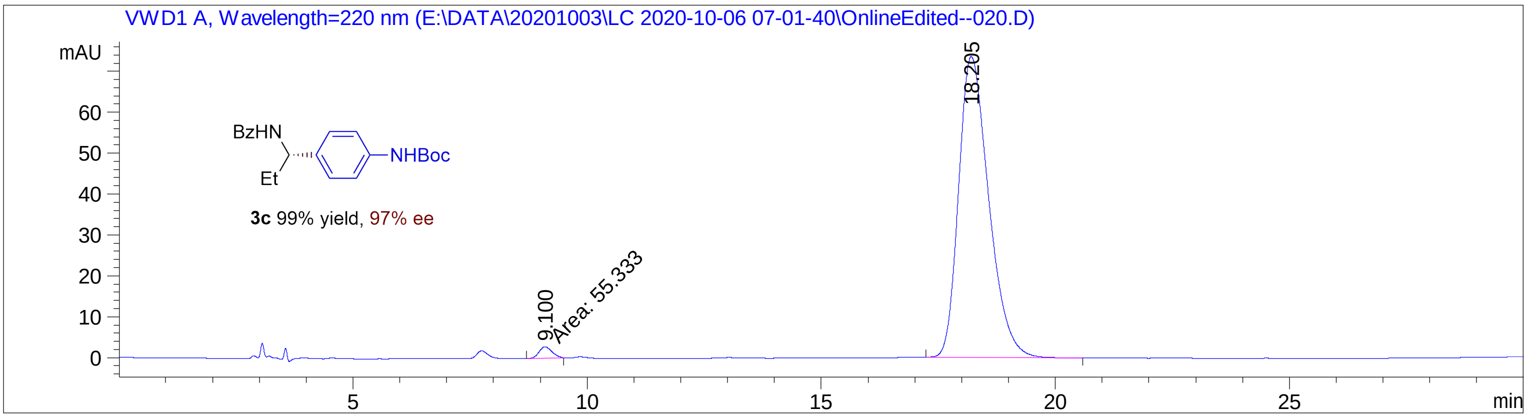

===

Area Percent Report

===

Sorted By : Signal
Multiplier : 1.0000
Dilution : 1.0000
Do not use Multiplier & Dilution Factor with ISTDs

Signal 1: VWD1 A, Wavelength=220 nm

Peak #	RetTime [min]	Type	Width [min]	Area [mAU*s]	Height [mAU]	Area %
1	9.100	MF	0.3303	55.33301	2.79198	1.6558
2	18.205	BB	0.6877	3286.36572	73.49329	98.3442

Totals : 3341.69873 76.28528

===

*** End of Report ***

Data File E:\DATA\20201003\LC 2020-10-06 07-01-40\OnlineEdited--011.D
Sample Name: YH-18-21-RAC

===

Acq. Operator : SYSTEM Seq. Line : 11
Acq. Instrument : HPLC1260 Location : P1-B1
Injection Date : 10/6/2020 12:09:39 PM Inj : 1
Inj Volume : 3.000 µl
Different Inj Volume from Sample Entry! Actual Inj Volume : 10.000 µl
Acq. Method : E:\DATA\20201003\LC 2020-10-06 07-01-40\15IPA_30_10_2.M
Last changed : 10/6/2020 7:03:24 AM by SYSTEM
Analysis Method : E:\DATA\20201003\LC 2020-10-06 07-01-40\15IPA_30_10_2.M (Sequence Method)
Last changed : 10/6/2020 1:14:49 PM by SYSTEM
(modified after loading)
Additional Info : Peak(s) manually integrated

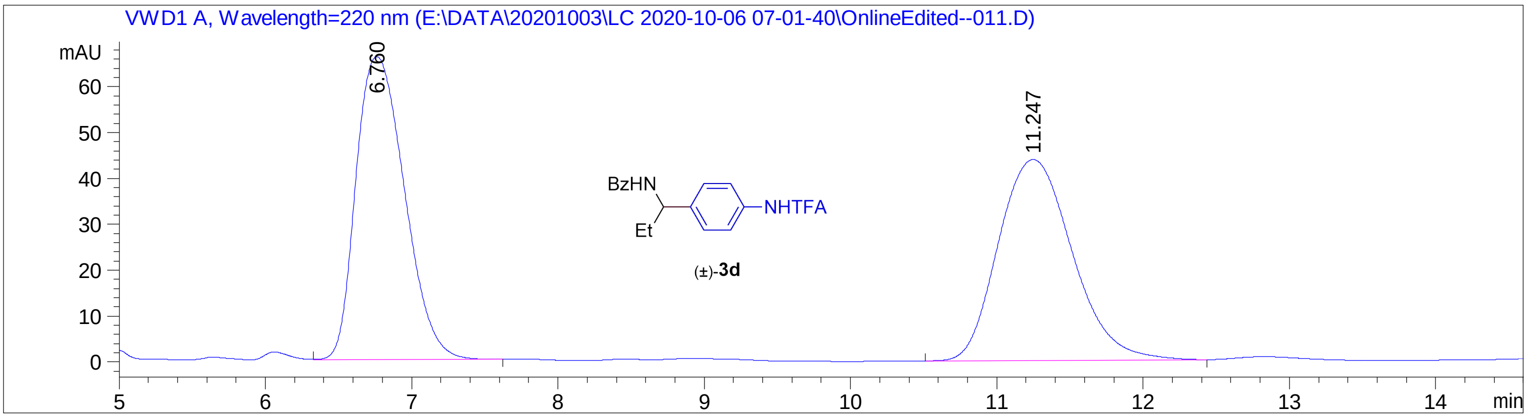

===

Area Percent Report

===

Sorted By : Signal
Multiplier : 1.0000
Dilution : 1.0000
Do not use Multiplier & Dilution Factor with ISTDs

Signal 1: VWD1 A, Wavelength=220 nm

Peak #	RetTime [min]	Type	Width [min]	Area [mAU*s]	Height [mAU]	Area %
1	6.760	VB	0.3702	1524.37830	66.06023	49.8085
2	11.247	BB	0.5573	1536.10059	43.78412	50.1915

Totals : 3060.47888 109.84435

===

*** End of Report ***

Data File E:\DATA\20201003\LC 2020-10-06 07-01-40\OnlineEdited--012.D
Sample Name: YH-18-21-EE

===

Acq. Operator : SYSTEM Seq. Line : 12
Acq. Instrument : HPLC1260 Location : P1-B2
Injection Date : 10/6/2020 12:40:29 PM Inj : 1
Inj Volume : 3.000 µl
Different Inj Volume from Sample Entry! Actual Inj Volume : 2.000 µl
Acq. Method : E:\DATA\20201003\LC 2020-10-06 07-01-40\15IPA_30_10_2.M
Last changed : 10/6/2020 7:03:24 AM by SYSTEM
Analysis Method : E:\DATA\20201003\LC 2020-10-06 07-01-40\15IPA_30_10_2.M (Sequence Method)
Last changed : 10/6/2020 1:14:49 PM by SYSTEM
(modified after loading)
Additional Info : Peak(s) manually integrated

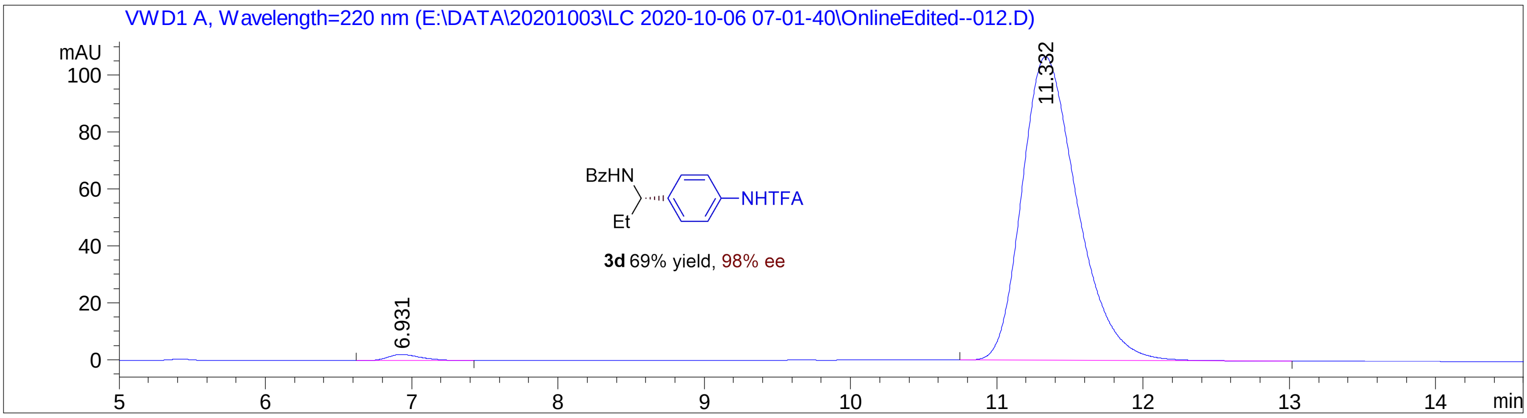

===

Area Percent Report

===

Sorted By : Signal
Multiplier : 1.0000
Dilution : 1.0000
Do not use Multiplier & Dilution Factor with ISTDs

Signal 1: VWD1 A, Wavelength=220 nm

Peak #	RetTime [min]	Type	Width [min]	Area [mAU*s]	Height [mAU]	Area %
1	6.931	BB	0.2385	34.70490	2.18254	1.2210
2	11.332	BB	0.4020	2807.67822	106.70854	98.7790

Totals : 2842.38312 108.89109

===

*** End of Report ***

Data File E:\DATA\20201027\LC 2020-11-17 15-04-56\OnlineEdited--003.D
Sample Name: YH-18-22-RAC

===

Acq. Operator : SYSTEM   Seq. Line : 3
Acq. Instrument : HPLC1260   Location : P1-F1
Injection Date : 11/17/2020 3:38:29 PM   Inj : 1
                                          Inj Volume : 3.000 µl
Acq. Method : E:\DATA\20201027\LC 2020-11-17 15-04-56\20IPA_30_10_1.M
Last changed : 11/17/2020 4:06:41 PM by SYSTEM
(modified after loading)
Analysis Method : E:\DATA\20201027\LC 2020-11-17 15-04-56\20IPA_30_10_1.M (Sequence Method)
Last changed : 11/17/2020 5:19:17 PM by SYSTEM
(modified after loading)
Additional Info : Peak(s) manually integrated

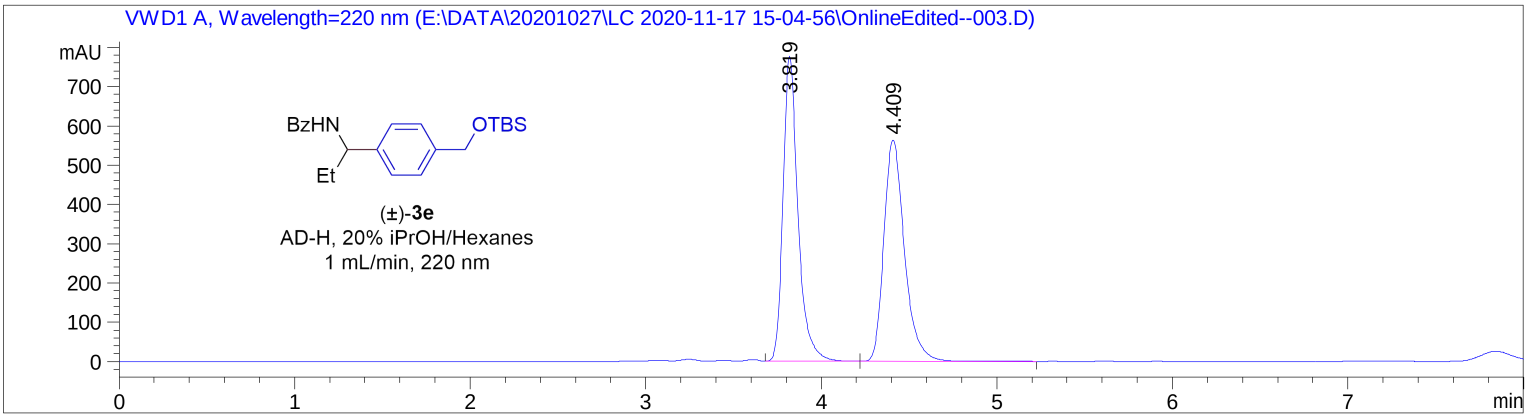

===

Area Percent Report

===

Sorted By : Signal
Multiplier : 1.0000
Dilution : 1.0000
Do not use Multiplier & Dilution Factor with ISTDs

Signal 1: VWD1 A, Wavelength=220 nm

Peak #	RetTime [min]	Type	Width [min]	Area [mAU*s]	Height [mAU]	Area %
1	3.819	BB	0.0892	4599.26611	775.93445	50.0864
2	4.409	BV R	0.1249	4583.39111	563.34161	49.9136

Totals : 9182.65723 1339.27606

===

*** End of Report ***

Data File E:\DATA\20201027\LC 2020-11-17 15-04-56\OnlineEdited--004.D
Sample Name: YH-18-22-EE

===

Acq. Operator : SYSTEM Seq. Line : 4
Acq. Instrument : HPLC1260 Location : P1-F2
Injection Date : 11/17/2020 4:07:30 PM Inj : 1
Inj Volume : 3.000 µl
Acq. Method : E:\DATA\20201027\LC 2020-11-17 15-04-56\20IPA_30_10_1.M
Last changed : 11/17/2020 4:06:41 PM by SYSTEM
Analysis Method : E:\DATA\20201027\LC 2020-11-17 15-04-56\20IPA_30_10_1.M (Sequence Method)
Last changed : 11/17/2020 5:19:17 PM by SYSTEM
(modified after loading)
Additional Info : Peak(s) manually integrated

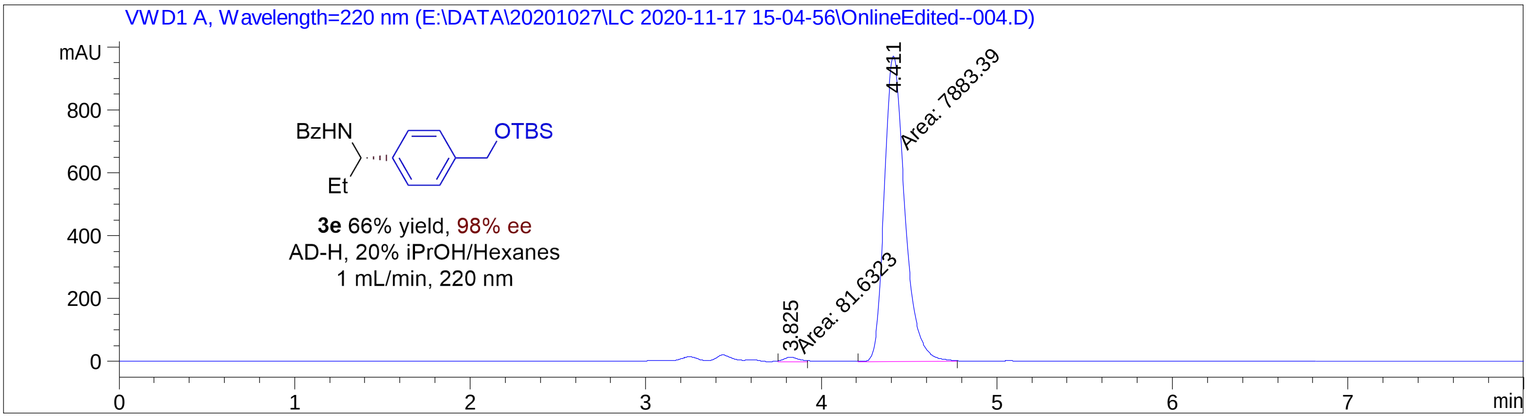

===

Area Percent Report

===

Sorted By : Signal
Multiplier : 1.0000
Dilution : 1.0000
Do not use Multiplier & Dilution Factor with ISTDs

Signal 1: VWD1 A, Wavelength=220 nm

Peak #	RetTime [min]	Type	Width [min]	Area [mAU*s]	Height [mAU]	Area %
1	3.825	FM	0.0989	81.63229	13.76095	1.0249
2	4.411	MF	0.1353	7883.39355	971.01105	98.9751
Totals :				7965.02585	984.77200	

===

*** End of Report ***

===

Acq. Operator : SYSTEM Seq. Line : 10
Acq. Instrument : HPLC1260 Location : P1-A1
Injection Date : 11/24/2020 1:05:10 PM Inj : 1
Inj Volume : 3.000 µl
Different Inj Volume from Sample Entry! Actual Inj Volume : 2.000 µl
Acq. Method : E:\DATA\20201027\LC 2020-11-24 09-35-16\20IPA_20_0.8_1.M
Last changed : 11/24/2020 11:13:48 AM by SYSTEM
Analysis Method : E:\DATA\20201027\LC 2020-11-24 09-35-16\20IPA_20_0.8_1.M (Sequence Method)
Last changed : 11/24/2020 1:33:19 PM by SYSTEM
(modified after loading)
Additional Info : Peak(s) manually integrated

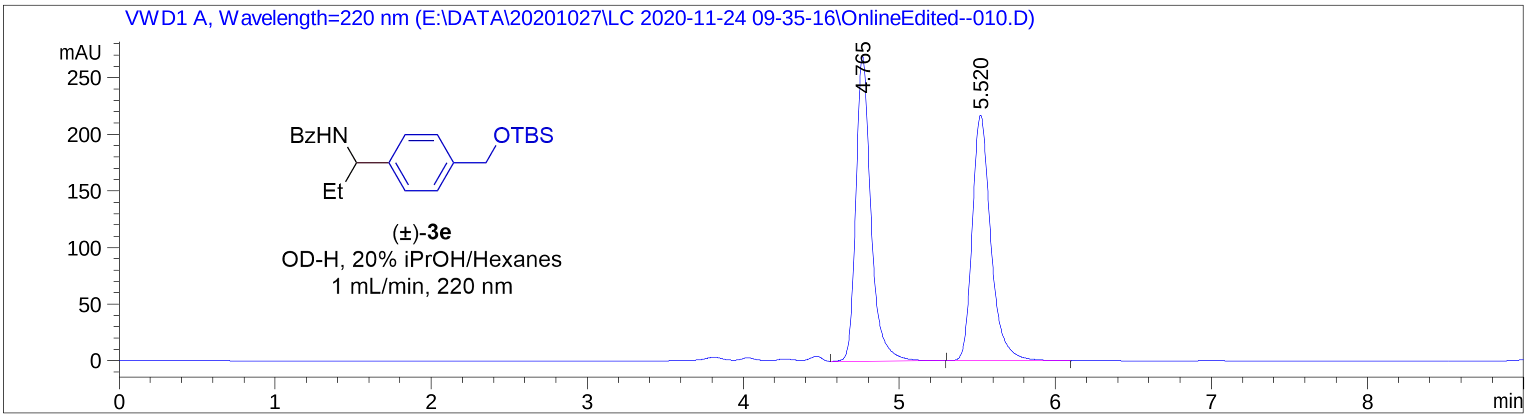

===

Area Percent Report

===

Sorted By : Signal
Multiplier : 1.0000
Dilution : 1.0000
Do not use Multiplier & Dilution Factor with ISTDs

Signal 1: VWD1 A, Wavelength=220 nm

Peak #	RetTime [min]	Type	Width [min]	Area [mAU*s]	Height [mAU]	Area %
1	4.765	BB	0.1017	1816.39221	269.30118	50.3151
2	5.520	BB	0.1254	1793.63892	217.08441	49.6849

Totals : 3610.03113 486.38559

===

*** End of Report ***

Data File E:\DATA\20201027\LC 2020-11-24 09-35-16\OnlineEdited--011.D
Sample Name: YH-18-22-EE-OD

===

Acq. Operator : SYSTEM Seq. Line : 11
Acq. Instrument : HPLC1260 Location : P1-A2
Injection Date : 11/24/2020 1:26:00 PM Inj : 1
Inj Volume : 3.000 µl
Acq. Method : E:\DATA\20201027\LC 2020-11-24 09-35-16\20IPA_20_0.8_1.M
Last changed : 11/24/2020 1:36:08 PM by SYSTEM
(modified after loading)
Analysis Method : E:\DATA\20201027\LC 2020-11-24 09-35-16\20IPA_20_0.8_1.M (Sequence Method)
Last changed : 11/24/2020 1:37:23 PM by SYSTEM
(modified after loading)
Additional Info : Peak(s) manually integrated

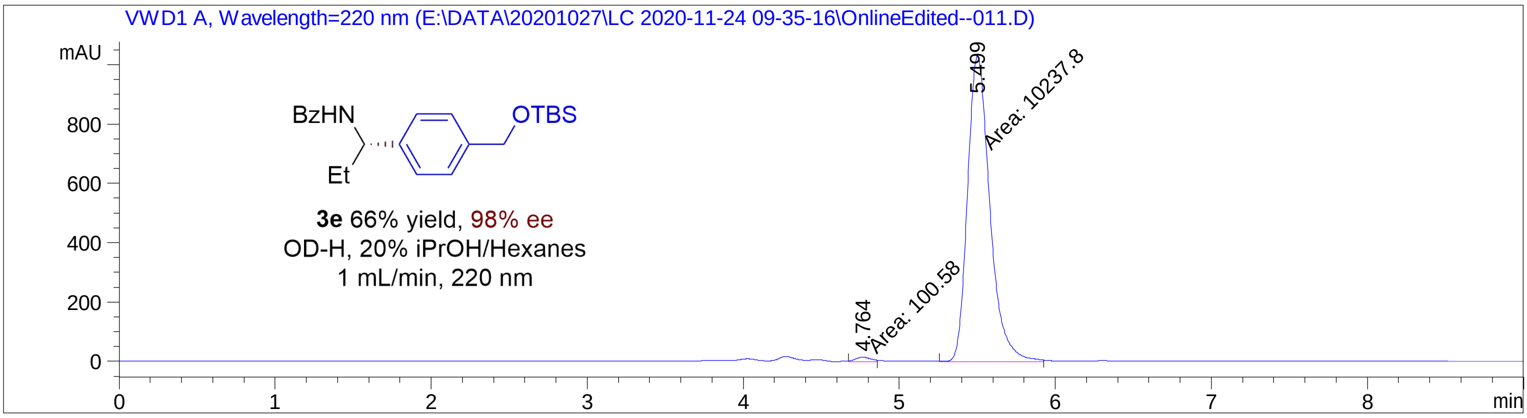

===
Area Percent Report
===

Sorted By : Signal
Multiplier : 1.0000
Dilution : 1.0000
Do not use Multiplier & Dilution Factor with ISTDs

Signal 1: VWD1 A, Wavelength=220 nm

Peak #	RetTime [min]	Type	Width [min]	Area [mAU*s]	Height [mAU]	Area %
1	4.764	FM	0.1136	100.57994	14.75874	0.9729
2	5.499	MF	0.1661	1.02378e4	1027.56250	99.0271

Totals : 1.03383e4 1042.32124

===
*** End of Report ***

===

Acq. Operator : SYSTEM Seq. Line : 21
Acq. Instrument : HPLC1260 Location : P1-C7
Injection Date : 10/6/2020 5:17:22 PM Inj : 1
Inj Volume : 3.000 µl
Different Inj Volume from Sample Entry! Actual Inj Volume : 1.000 µl
Acq. Method : E:\DATA\20201003\LC 2020-10-06 07-01-40\20IPA_30_10_2.M
Last changed : 10/6/2020 11:07:22 AM by SYSTEM
Analysis Method : E:\DATA\20201003\LC 2020-10-06 07-01-40\20IPA_30_10_2.M (Sequence Method)
Last changed : 10/6/2020 6:22:51 PM by SYSTEM
(modified after loading)
Additional Info : Peak(s) manually integrated

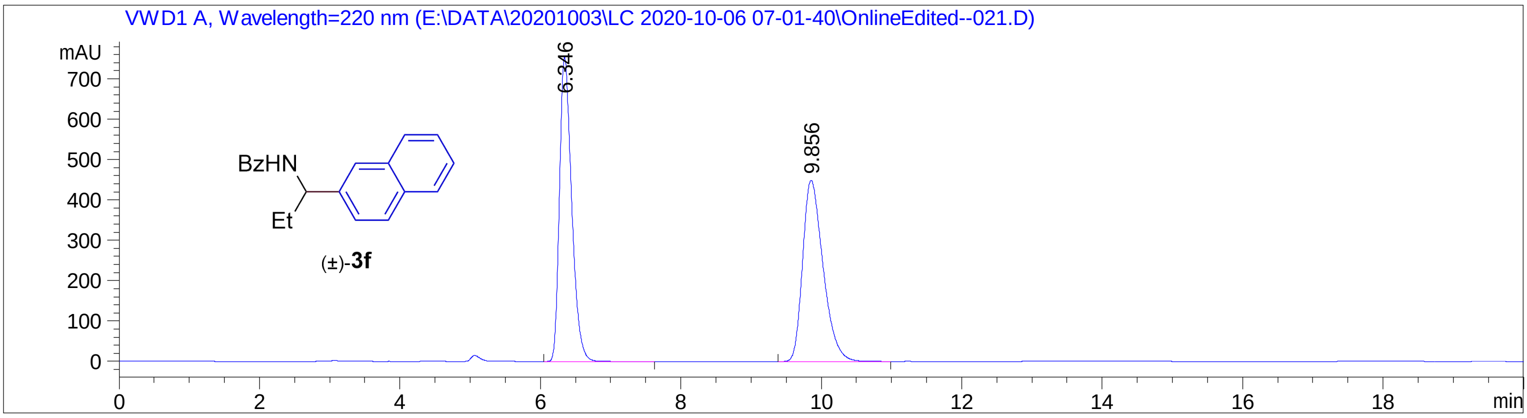

===

Area Percent Report

===

Sorted By : Signal
Multiplier : 1.0000
Dilution : 1.0000
Do not use Multiplier & Dilution Factor with ISTDs

Signal 1: VWD1 A, Wavelength=220 nm

Peak #	RetTime [min]	Type	Width [min]	Area [mAU*s]	Height [mAU]	Area %
1	6.346	BB	0.1844	9091.86328	755.46844	50.0239
2	9.856	BB	0.3090	9083.17676	449.03717	49.9761

Totals : 1.81750e4 1204.50562

===

*** End of Report ***

Data File E:\DATA\20201003\LC 2020-10-06 07-01-40\OnlineEdited--022.D
Sample Name: YH-18-24-EE

===

Acq. Operator : SYSTEM Seq. Line : 22
Acq. Instrument : HPLC1260 Location : P1-C8
Injection Date : 10/6/2020 5:48:08 PM Inj : 1
Inj Volume : 3.000 µl
Different Inj Volume from Sample Entry! Actual Inj Volume : 2.000 µl
Acq. Method : E:\DATA\20201003\LC 2020-10-06 07-01-40\20IPA_30_10_2.M
Last changed : 10/6/2020 11:07:22 AM by SYSTEM
Analysis Method : E:\DATA\20201003\LC 2020-10-06 07-01-40\20IPA_30_10_2.M (Sequence Method)
Last changed : 10/6/2020 6:22:51 PM by SYSTEM
(modified after loading)
Additional Info : Peak(s) manually integrated

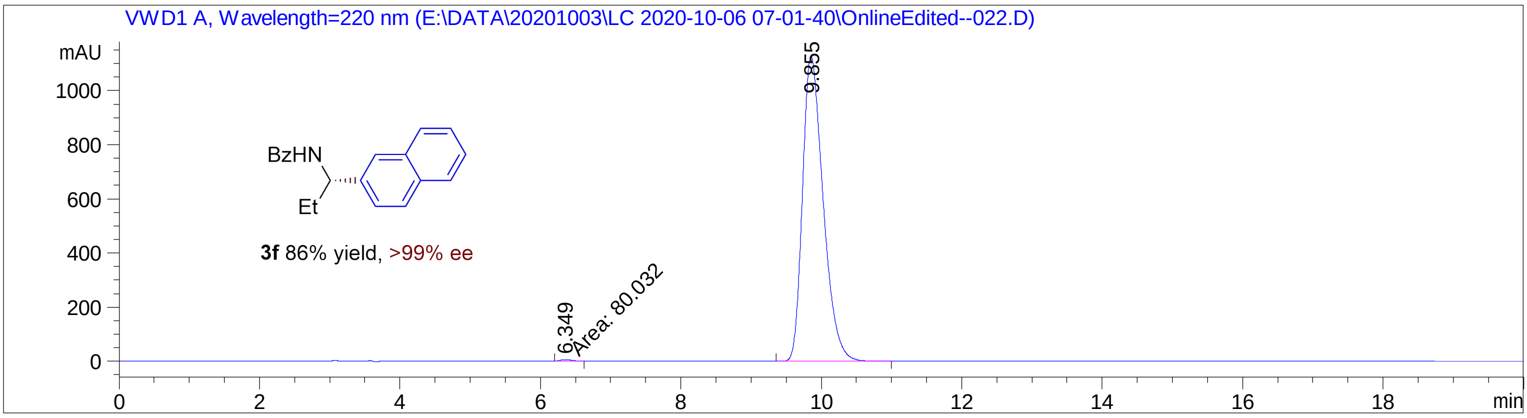

===

Area Percent Report

===

Sorted By : Signal
Multiplier : 1.0000
Dilution : 1.0000
Do not use Multiplier & Dilution Factor with ISTDs

Signal 1: VWD1 A, Wavelength=220 nm

Peak #	RetTime [min]	Type	Width [min]	Area [mAU*s]	Height [mAU]	Area %
1	6.349	FM	0.1989	80.03197	6.70680	0.3464
2	9.855	BB	0.3126	2.30221e4	1125.50293	99.6536

Totals : 2.31021e4 1132.20973

===

*** End of Report ***

Data File E:\DATA\20201003\LC 2020-10-07 22-33-54\OnlineEdited--035.D
Sample Name: YH-18-44-RAC

===

Acq. Operator : SYSTEM Seq. Line : 35
Acq. Instrument : HPLC1260 Location : P1-B4
Injection Date : 10/8/2020 1:43:04 PM Inj : 1
Inj Volume : 3.000 µl
Different Inj Volume from Sample Entry! Actual Inj Volume : 1.000 µl
Acq. Method : E:\DATA\20201003\LC 2020-10-07 22-33-54\20IPA_30_10_2.M
Last changed : 10/8/2020 1:42:37 PM by SYSTEM
(modified after loading)
Analysis Method : E:\DATA\20201003\LC 2020-10-07 22-33-54\20IPA_30_10_2.M (Sequence Method)
Last changed : 10/8/2020 2:26:54 PM by SYSTEM
(modified after loading)
Additional Info : Peak(s) manually integrated

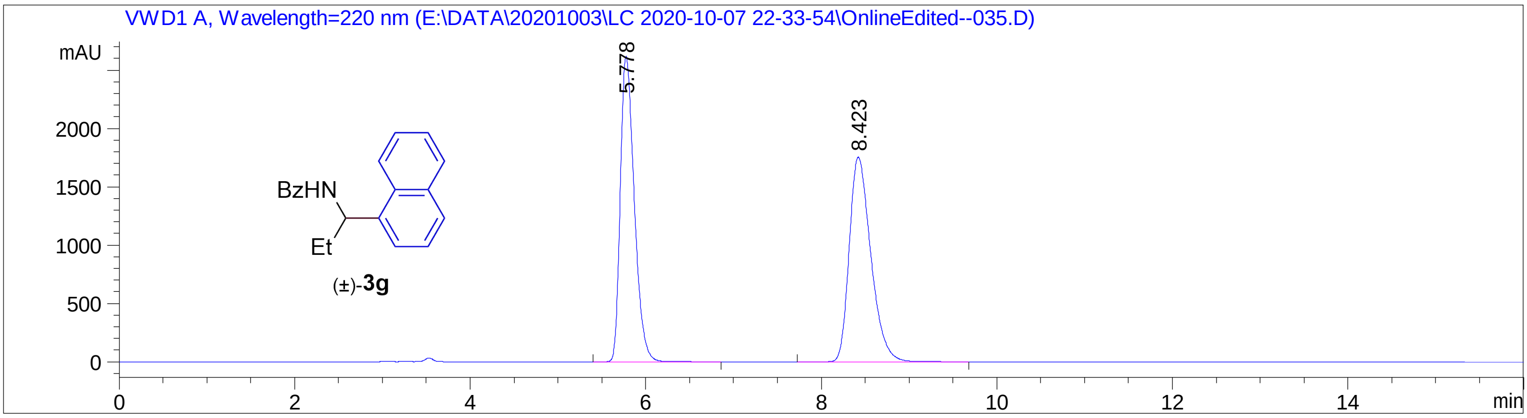

===

Area Percent Report

===

Sorted By : Signal
Multiplier : 1.0000
Dilution : 1.0000
Do not use Multiplier & Dilution Factor with ISTDs

Signal 1: VWD1 A, Wavelength=220 nm

Peak #	RetTime [min]	Type	Width [min]	Area [mAU*s]	Height [mAU]	Area %
1	5.778	BB	0.1700	2.89411e4	2617.80542	49.1562
2	8.423	BB	0.2620	2.99347e4	1755.13647	50.8438

Totals : 5.88758e4 4372.94189

===

*** End of Report ***

Data File E:\DATA\20201003\LC 2020-10-07 22-33-54\OnlineEdited--036.D
Sample Name: YH-18-44-EE

===

Acq. Operator : SYSTEM Seq. Line : 36
Acq. Instrument : HPLC1260 Location : P1-B5
Injection Date : 10/8/2020 2:13:50 PM Inj : 1
Inj Volume : 3.000 µl
Different Inj Volume from Sample Entry! Actual Inj Volume : 2.000 µl
Acq. Method : E:\DATA\20201003\LC 2020-10-07 22-33-54\20IPA_30_10_2.M
Last changed : 10/8/2020 2:27:24 PM by SYSTEM
(modified after loading)
Analysis Method : E:\DATA\20201003\LC 2020-10-07 22-33-54\20IPA_30_10_2.M (Sequence Method)
Last changed : 10/8/2020 2:37:04 PM by SYSTEM
(modified after loading)
Additional Info : Peak(s) manually integrated

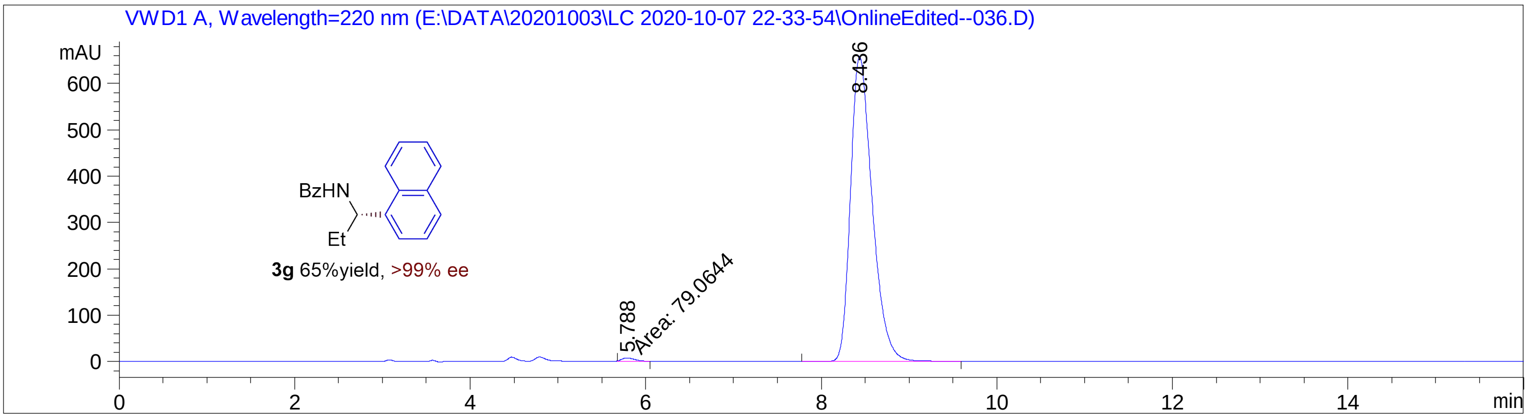

===

Area Percent Report

===

Sorted By : Signal
Multiplier : 1.0000
Dilution : 1.0000
Do not use Multiplier & Dilution Factor with ISTDs

Signal 1: VWD1 A, Wavelength=220 nm

Peak #	RetTime [min]	Type	Width [min]	Area [mAU*s]	Height [mAU]	Area %
1	5.788	FM	0.1733	79.06438	7.60166	0.6969
2	8.436	BB	0.2618	1.12657e4	657.77692	99.3031

Totals : 1.13447e4 665.37857

===

*** End of Report ***

===

Acq. Operator : SYSTEM Seq. Line : 6
Acq. Instrument : HPLC1260 Location : P1-B5
Injection Date : 10/4/2020 1:36:13 AM Inj : 1
Inj Volume : 3.000 µl
Different Inj Volume from Sample Entry! Actual Inj Volume : 1.000 µl
Acq. Method : E:\DATA\20201003\LC 2020-10-03 23-00-22\20IPA_30_10_2.M
Last changed : 10/3/2020 11:00:51 PM by SYSTEM
Analysis Method : E:\DATA\20201003\LC 2020-10-03 23-00-22\20IPA_30_10_2.M (Sequence Method)
Last changed : 10/4/2020 9:36:39 AM by SYSTEM
(modified after loading)
Additional Info : Peak(s) manually integrated

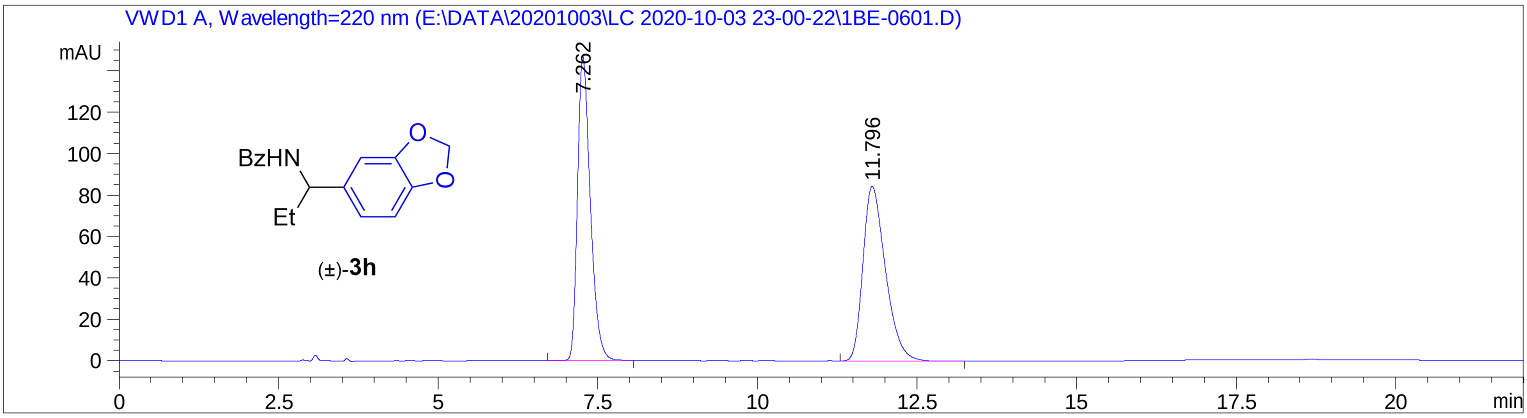

===

Area Percent Report

===

Sorted By : Signal
Multiplier : 1.0000
Dilution : 1.0000
Do not use Multiplier & Dilution Factor with ISTDs

Signal 1: VWD1 A, Wavelength=220 nm

Peak #	RetTime [min]	Type	Width [min]	Area [mAU*s]	Height [mAU]	Area %
1	7.262	BB	0.2155	2069.46729	146.72794	50.0099
2	11.796	BB	0.3742	2068.64893	84.31846	49.9901

Totals : 4138.11621 231.04639

===

*** End of Report ***

===

Acq. Operator : SYSTEM Seq. Line : 7
Acq. Instrument : HPLC1260 Location : P1-B6
Injection Date : 10/4/2020 2:06:59 AM Inj : 1
Inj Volume : 3.000 µl
Different Inj Volume from Sample Entry! Actual Inj Volume : 2.000 µl
Acq. Method : E:\DATA\20201003\LC 2020-10-03 23-00-22\20IPA_30_10_2.M
Last changed : 10/3/2020 11:00:51 PM by SYSTEM
Analysis Method : E:\DATA\20201003\LC 2020-10-03 23-00-22\20IPA_30_10_2.M (Sequence Method)
Last changed : 10/4/2020 9:36:39 AM by SYSTEM
(modified after loading)
Additional Info : Peak(s) manually integrated

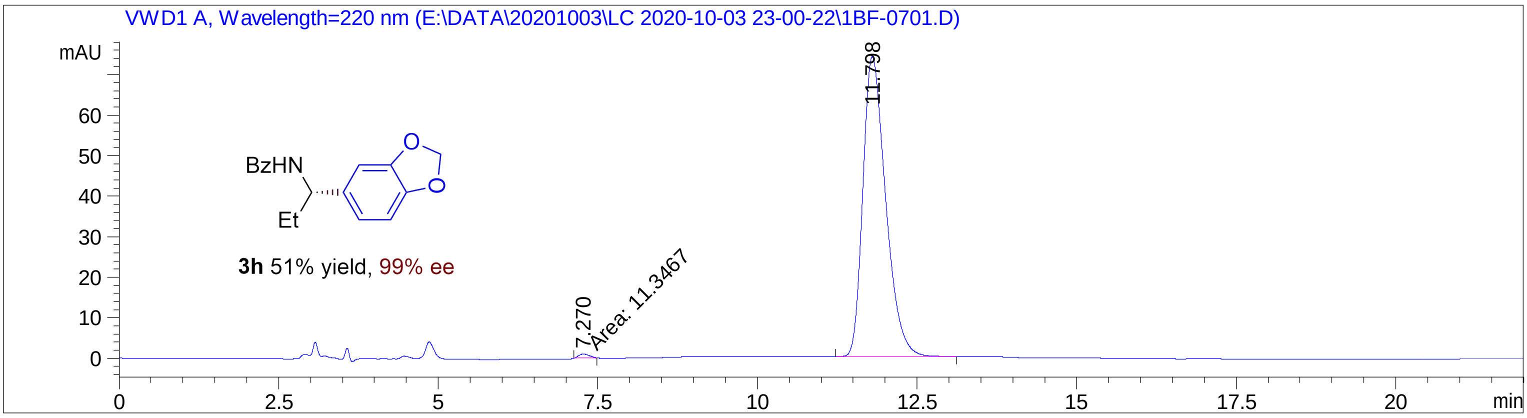

===
Area Percent Report
===

Sorted By : Signal
Multiplier : 1.0000
Dilution : 1.0000
Do not use Multiplier & Dilution Factor with ISTDs

Signal 1: VWD1 A, Wavelength=220 nm

Peak #	RetTime [min]	Type	Width [min]	Area [mAU*s]	Height [mAU]	Area %
1	7.270	MM	0.1934	11.34673	9.77630e-1	0.6153
2	11.798	BB	0.3801	1832.77869	73.96531	99.3847

Totals : 1844.12542 74.94294

===
*** End of Report ***

Data File E:\DATA\20201003\LC 2020-10-05 08-59-28\OnlineEdited--015.D
Sample Name: YH-18-16-RAC

===

Acq. Operator : SYSTEM Seq. Line : 15
Acq. Instrument : HPLC1260 Location : P1-B5
Injection Date : 10/5/2020 2:15:18 PM Inj : 1
Inj Volume : 3.000 µl
Different Inj Volume from Sample Entry! Actual Inj Volume : 1.000 µl
Acq. Method : E:\DATA\20201003\LC 2020-10-05 08-59-28\20IPA_22_10_2.M
Last changed : 10/5/2020 9:52:42 AM by SYSTEM
Analysis Method : E:\DATA\20201003\LC 2020-10-05 08-59-28\20IPA_22_10_2.M (Sequence Method)
Last changed : 10/5/2020 2:56:31 PM by SYSTEM
(modified after loading)
Additional Info : Peak(s) manually integrated

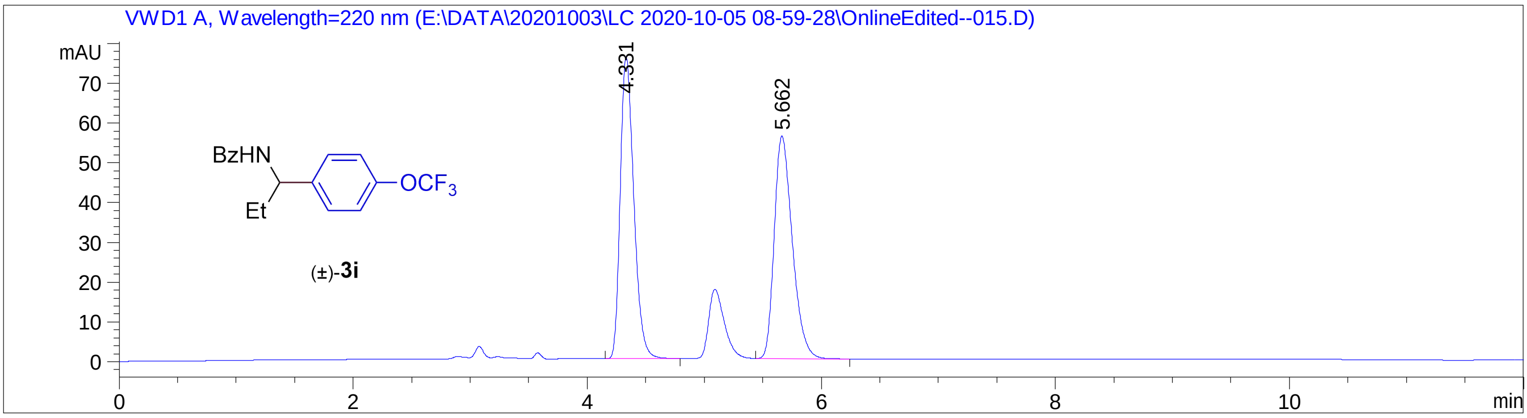

===

Area Percent Report

===

Sorted By : Signal
Multiplier : 1.0000
Dilution : 1.0000
Do not use Multiplier & Dilution Factor with ISTDs

Signal 1: VWD1 A, Wavelength=220 nm

Peak #	RetTime [min]	Type	Width [min]	Area [mAU*s]	Height [mAU]	Area %
1	4.331	BB	0.1250	611.78076	75.94442	50.3870
2	5.662	VB	0.1654	602.38226	56.03320	49.6130

Totals : 1214.16302 131.97762

===

*** End of Report ***

===

Acq. Operator : SYSTEM   Seq. Line : 16
Acq. Instrument : HPLC1260   Location : P1-B6
Injection Date : 10/5/2020 2:29:04 PM   Inj : 1
Inj Volume : 3.000 µl
Different Inj Volume from Sample Entry! Actual Inj Volume : 2.000 µl
Acq. Method : E:\DATA\20201003\LC 2020-10-05 08-59-28\20IPA_22_10_2.M
Last changed : 10/5/2020 9:52:42 AM by SYSTEM
Analysis Method : E:\DATA\20201003\LC 2020-10-05 08-59-28\20IPA_22_10_2.M (Sequence Method)
Last changed : 10/5/2020 2:57:38 PM by SYSTEM
(modified after loading)
Additional Info : Peak(s) manually integrated

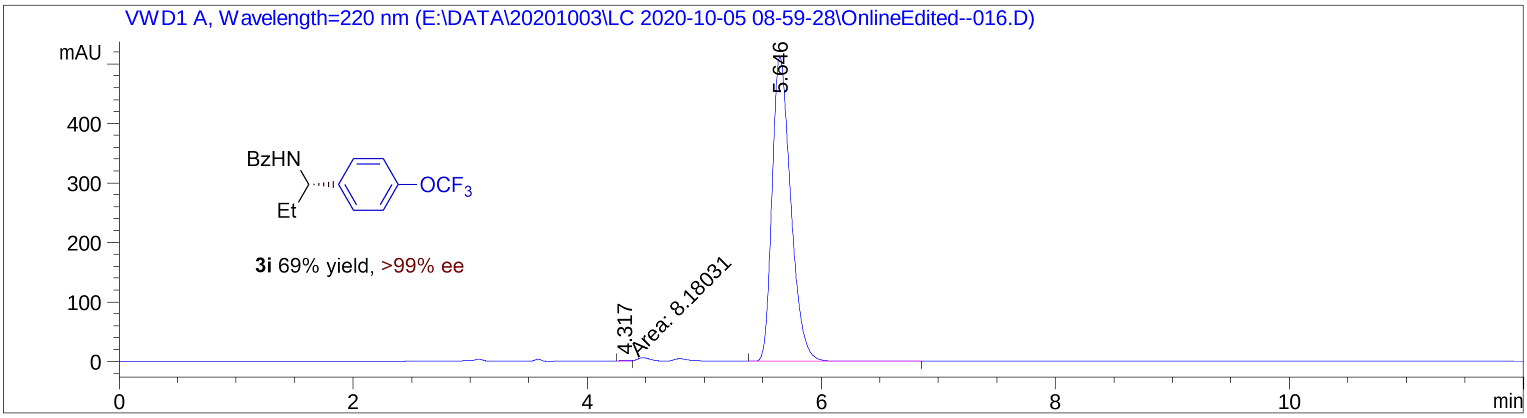

===

Area Percent Report

===

Sorted By : Signal
Multiplier : 1.0000
Dilution : 1.0000
Do not use Multiplier & Dilution Factor with ISTDs

Signal 1: VWD1 A, Wavelength=220 nm

Peak #	RetTime [min]	Type	Width [min]	Area [mAU*s]	Height [mAU]	Area %
1	4.317	FM	0.1117	8.18031	1.22034	0.1441
2	5.646	BB	0.1701	5667.77930	512.14325	99.8559

Totals : 5675.95961 513.36359

===

*** End of Report ***

Data File E:\DATA\20201003\LC 2020-10-06 00-56-07\OnlineEdited--005.D
Sample Name: YH-18-15-RAC

===

Acq. Operator : SYSTEM Seq. Line : 5
Acq. Instrument : HPLC1260 Location : P1-C1
Injection Date : 10/6/2020 2:53:40 AM Inj : 1
Inj Volume : 3.000 µl
Different Inj Volume from Sample Entry! Actual Inj Volume : 1.000 µl
Acq. Method : E:\DATA\20201003\LC 2020-10-06 00-56-07\20IPA_30_10_2.M
Last changed : 10/6/2020 1:19:29 AM by SYSTEM
Analysis Method : E:\DATA\20201003\LC 2020-10-06 00-56-07\20IPA_30_10_2.M (Sequence Method)
Last changed : 10/6/2020 7:04:09 AM by SYSTEM
(modified after loading)
Additional Info : Peak(s) manually integrated

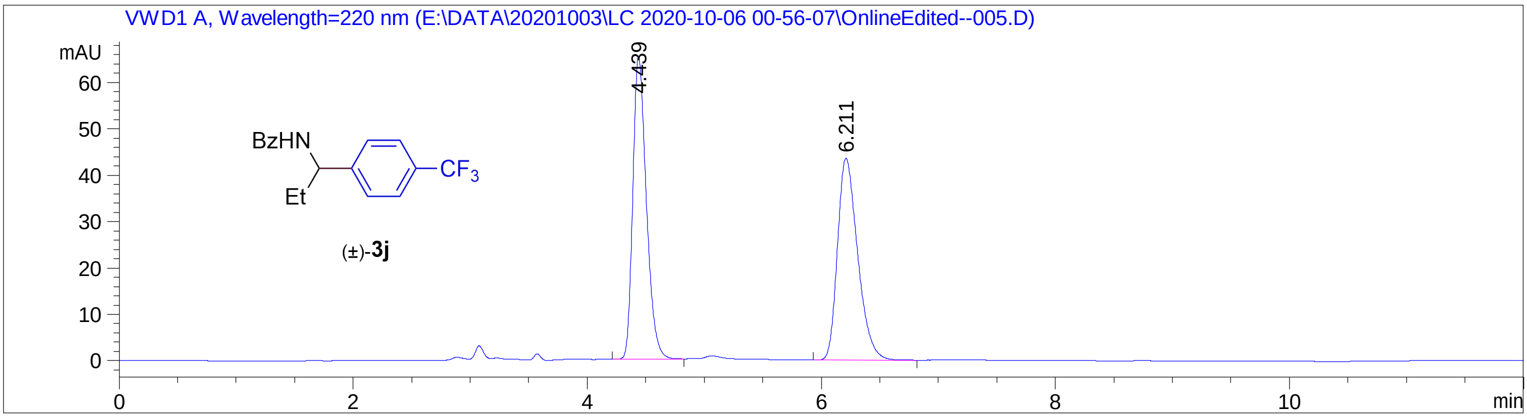

===

Area Percent Report

===

Sorted By : Signal
Multiplier : 1.0000
Dilution : 1.0000
Do not use Multiplier & Dilution Factor with ISTDs

Signal 1: VWD1 A, Wavelength=220 nm

Peak #	RetTime [min]	Type	Width [min]	Area [mAU*s]	Height [mAU]	Area %
1	4.439	BB	0.1229	525.07666	65.23978	50.2056
2	6.211	BB	0.1838	520.77570	43.44968	49.7944

Totals : 1045.85236 108.68946

===

*** End of Report ***

Data File E:\DATA\20201003\LC 2020-10-06 00-56-07\OnlineEdited--006.D
Sample Name: YH-18-15-EE

===

Acq. Operator : SYSTEM Seq. Line : 6
Acq. Instrument : HPLC1260 Location : P1-C2
Injection Date : 10/6/2020 3:24:26 AM Inj : 1
Inj Volume : 3.000 µl
Different Inj Volume from Sample Entry! Actual Inj Volume : 2.000 µl
Acq. Method : E:\DATA\20201003\LC 2020-10-06 00-56-07\20IPA_30_10_2.M
Last changed : 10/6/2020 1:19:29 AM by SYSTEM
Analysis Method : E:\DATA\20201003\LC 2020-10-06 00-56-07\20IPA_30_10_2.M (Sequence Method)
Last changed : 10/6/2020 7:04:09 AM by SYSTEM
(modified after loading)
Additional Info : Peak(s) manually integrated

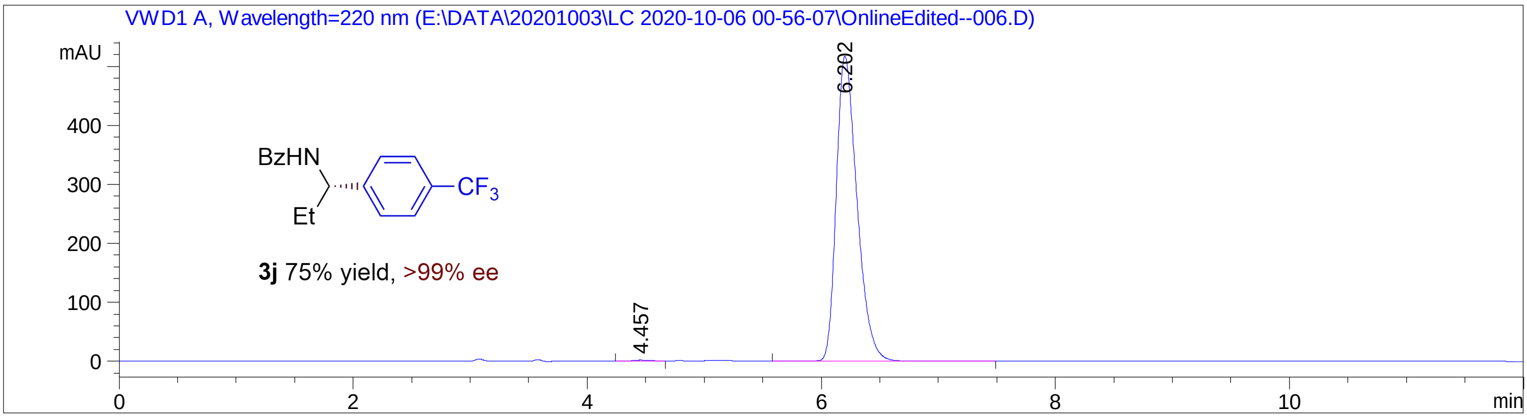

===

Area Percent Report

===

Sorted By : Signal
Multiplier : 1.0000
Dilution : 1.0000
Do not use Multiplier & Dilution Factor with ISTDs

Signal 1: VWD1 A, Wavelength=220 nm

Peak #	RetTime [min]	Type	Width [min]	Area [mAU*s]	Height [mAU]	Area %
1	4.457	BV	0.1571	18.36158	1.72682	0.2880
2	6.202	BB	0.1896	6357.44873	516.38190	99.7120

Totals : 6375.81031 518.10872

===

*** End of Report ***

Data File E:\DATA\20200921\LC 2020-09-29 09-48-51\1BA-0201.D
Sample Name: YH-18-3-RAC

===

Acq. Operator : SYSTEM Seq. Line : 2
Acq. Instrument : HPLC1260 Location : P1-B1
Injection Date : 9/29/2020 10:21:40 AM Inj : 1
Inj Volume : 3.000 µl
Different Inj Volume from Sample Entry! Actual Inj Volume : 1.000 µl
Acq. Method : E:\DATA\20200921\LC 2020-09-29 09-48-51\20IPA_30_10_4.M
Last changed : 9/29/2020 9:48:51 AM by SYSTEM
Analysis Method : E:\DATA\20200921\LC 2020-09-29 09-48-51\20IPA_30_10_4.M (Sequence Method)
Last changed : 9/29/2020 11:23:39 AM by SYSTEM
(modified after loading)
Additional Info : Peak(s) manually integrated

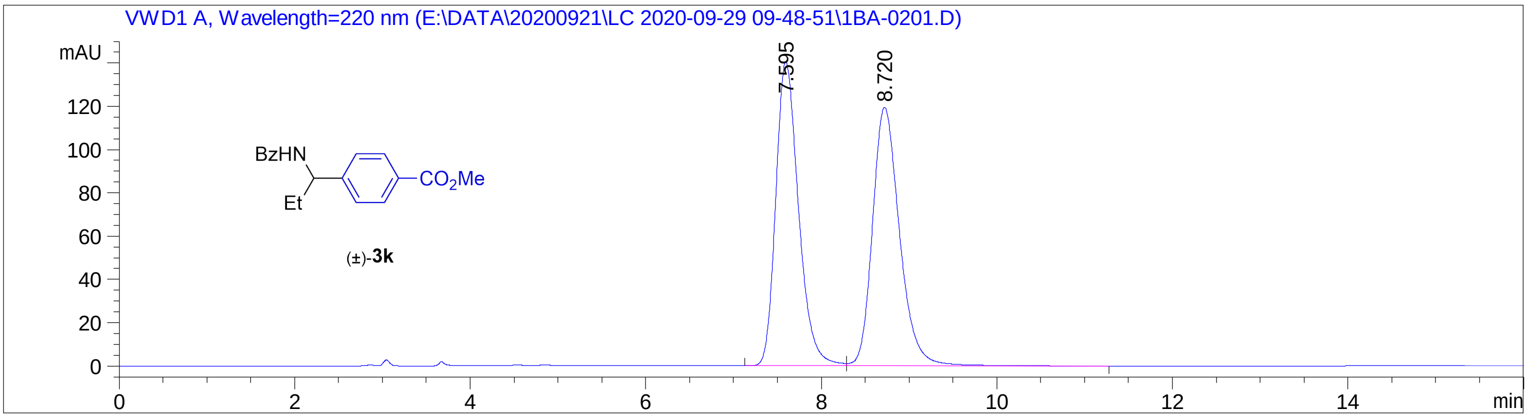

===

Area Percent Report

===

Sorted By : Signal
Multiplier : 1.0000
Dilution : 1.0000
Do not use Multiplier & Dilution Factor with ISTDs

Signal 1: VWD1 A, Wavelength=220 nm

Peak #	RetTime [min]	Type	Width [min]	Area [mAU*s]	Height [mAU]	Area %
1	7.595	BV	0.2764	2532.02734	140.41141	49.7439
2	8.720	VB	0.3280	2558.09717	119.39333	50.2561

Totals : 5090.12451 259.80473

===

*** End of Report ***

Data File E:\DATA\20200921\LC 2020-09-29 09-48-51\1BB-0301.D
Sample Name: YH-18-3-EE

===

Acq. Operator : SYSTEM Seq. Line : 3
Acq. Instrument : HPLC1260 Location : P1-B2
Injection Date : 9/29/2020 10:52:25 AM Inj : 1
Inj Volume : 3.000 µl
Different Inj Volume from Sample Entry! Actual Inj Volume : 2.000 µl
Acq. Method : E:\DATA\20200921\LC 2020-09-29 09-48-51\20IPA_30_10_4.M
Last changed : 9/29/2020 11:20:53 AM by SYSTEM
(modified after loading)
Analysis Method : E:\DATA\20200921\LC 2020-09-29 09-48-51\20IPA_30_10_4.M (Sequence Method)
Last changed : 9/29/2020 11:22:57 AM by SYSTEM
(modified after loading)
Additional Info : Peak(s) manually integrated

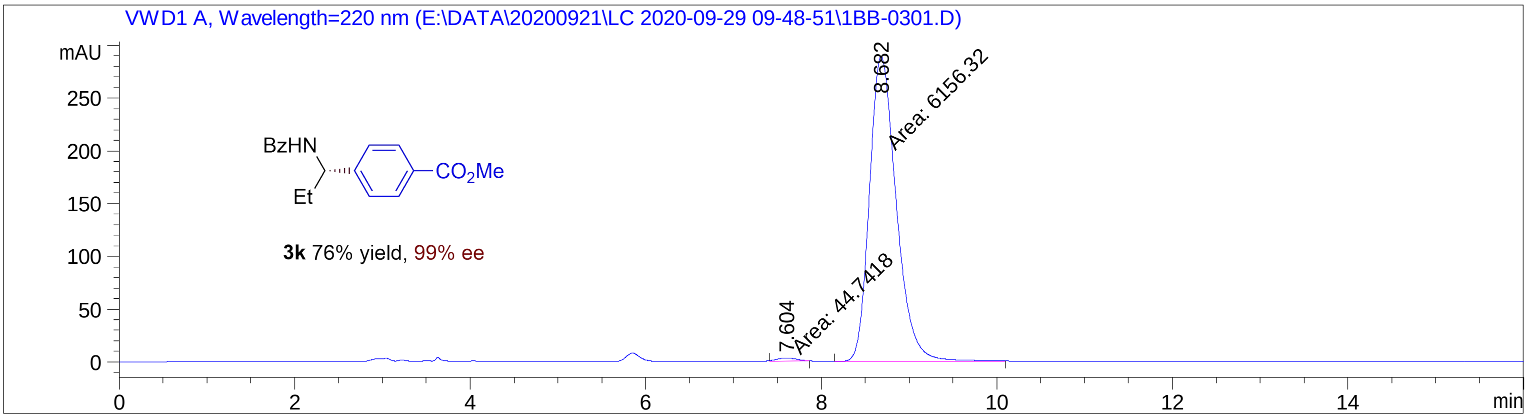

===

Area Percent Report

===

Sorted By : Signal
Multiplier : 1.0000
Dilution : 1.0000
Do not use Multiplier & Dilution Factor with ISTDs

Signal 1: VWD1 A, Wavelength=220 nm

Peak #	RetTime [min]	Type	Width [min]	Area [mAU*s]	Height [mAU]	Area %
1	7.604	FM	0.2578	44.74183	2.89252	0.7215
2	8.682	MF	0.3551	6156.31836	288.94373	99.2785

Totals : 6201.06018 291.83624

===

*** End of Report ***

Data File E:\DATA\20201003\LC 2020-10-06 07-01-40\OnlineEdited--004.D
Sample Name: YH-18-18-RAC

===
Acq. Operator : SYSTEM Seq. Line : 4
Acq. Instrument : HPLC1260 Location : P1-C5
Injection Date : 10/6/2020 8:36:00 AM Inj : 1
Inj Volume : 3.000 µl
Different Inj Volume from Sample Entry! Actual Inj Volume : 1.000 µl
Acq. Method : E:\DATA\20201003\LC 2020-10-06 07-01-40\20IPA_30_10_2.M
Last changed : 10/6/2020 7:03:10 AM by SYSTEM
Analysis Method : E:\DATA\20201003\LC 2020-10-06 07-01-40\20IPA_30_10_2.M (Sequence Method)
Last changed : 10/6/2020 9:09:59 AM by SYSTEM
(modified after loading)
Additional Info : Peak(s) manually integrated

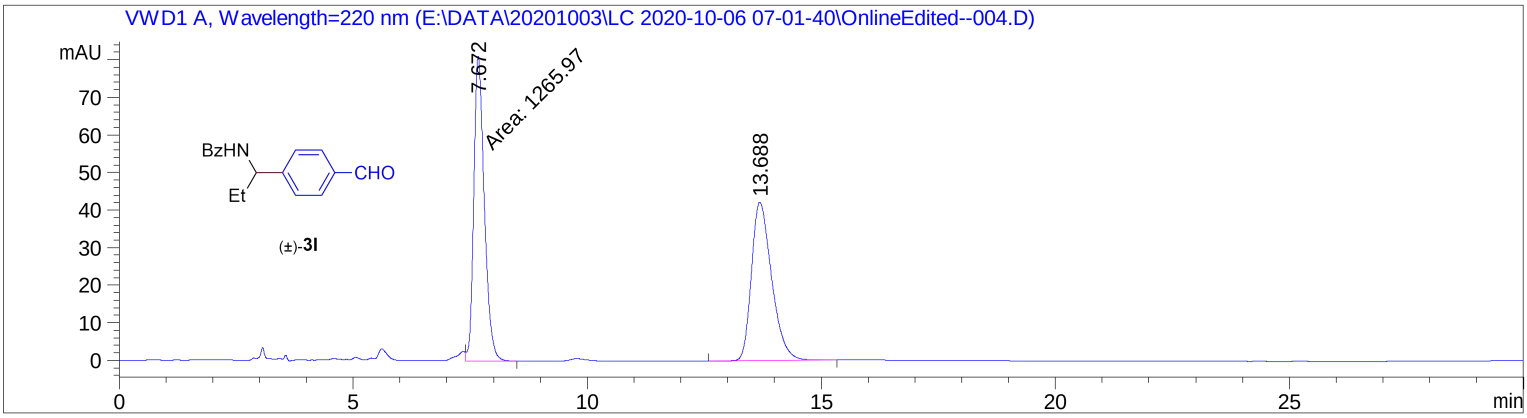

===
Area Percent Report
===

Sorted By : Signal
Multiplier : 1.0000
Dilution : 1.0000
Do not use Multiplier & Dilution Factor with ISTDs

Signal 1: VWD1 A, Wavelength=220 nm

Peak #	RetTime [min]	Type	Width [min]	Area [mAU*s]	Height [mAU]	Area %
1	7.672	FM	0.2606	1265.97180	80.97002	49.9981
2	13.688	BB	0.4591	1266.06970	42.19156	50.0019

Totals : 2532.04150 123.16158

===
*** End of Report ***

Data File E:\DATA\20201003\LC 2020-10-06 07-01-40\OnlineEdited--005.D
Sample Name: YH-18-18-EE

===

Acq. Operator : SYSTEM Seq. Line : 5
Acq. Instrument : HPLC1260 Location : P1-C6
Injection Date : 10/6/2020 9:06:47 AM Inj : 1
Inj Volume : 3.000 µl
Different Inj Volume from Sample Entry! Actual Inj Volume : 2.000 µl
Acq. Method : E:\DATA\20201003\LC 2020-10-06 07-01-40\20IPA_30_10_2.M
Last changed : 10/6/2020 7:03:10 AM by SYSTEM
Analysis Method : E:\DATA\20201003\LC 2020-10-06 07-01-40\20IPA_30_10_2.M (Sequence Method)
Last changed : 10/6/2020 10:35:27 AM by SYSTEM
(modified after loading)
Additional Info : Peak(s) manually integrated

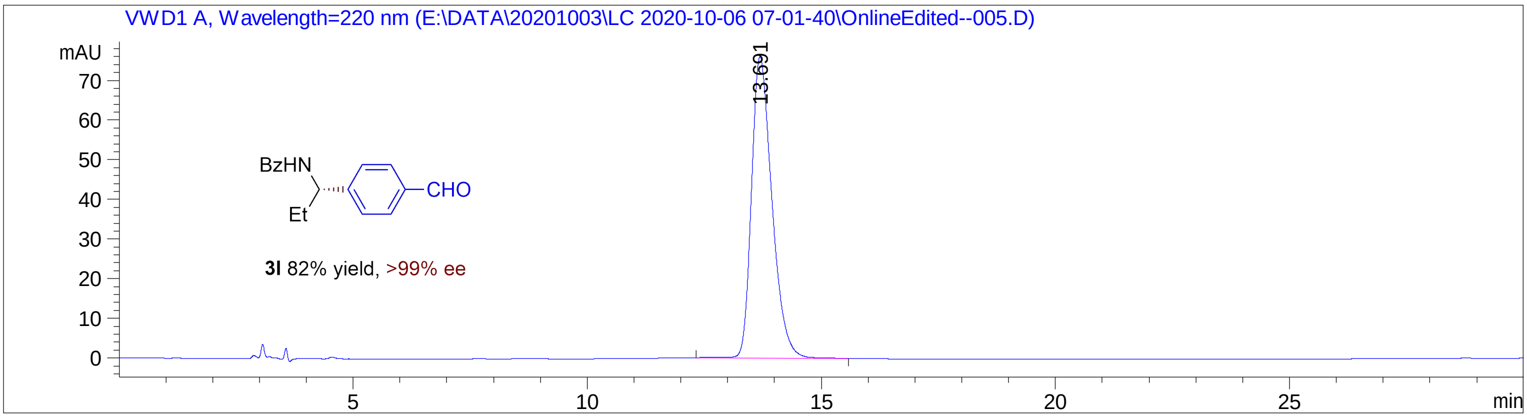

===

Area Percent Report

===

Sorted By : Signal
Multiplier : 1.0000
Dilution : 1.0000
Do not use Multiplier & Dilution Factor with ISTDs

Signal 1: VWD1 A, Wavelength=220 nm

Peak #	RetTime [min]	Type	Width [min]	Area [mAU*s]	Height [mAU]	Area %
1	13.691	BB	0.4641	2306.76611	76.00164	100.0000

Totals : 2306.76611 76.00164

===

*** End of Report ***

Data File E:\DATA\20201003\LC 2020-10-03 23-00-22\1BA-0201.D
Sample Name: YH-18-11-RAC

===

Acq. Operator : SYSTEM Seq. Line : 2
Acq. Instrument : HPLC1260 Location : P1-B1
Injection Date : 10/3/2020 11:33:12 PM Inj : 1
Inj Volume : 3.000 µl
Different Inj Volume from Sample Entry! Actual Inj Volume : 2.000 µl
Acq. Method : E:\DATA\20201003\LC 2020-10-03 23-00-22\20IPA_30_10_2.M
Last changed : 10/3/2020 11:00:51 PM by SYSTEM
Analysis Method : E:\DATA\20201003\LC 2020-10-03 23-00-22\20IPA_30_10_2.M (Sequence Method)
Last changed : 10/4/2020 9:34:03 AM by SYSTEM
(modified after loading)
Additional Info : Peak(s) manually integrated

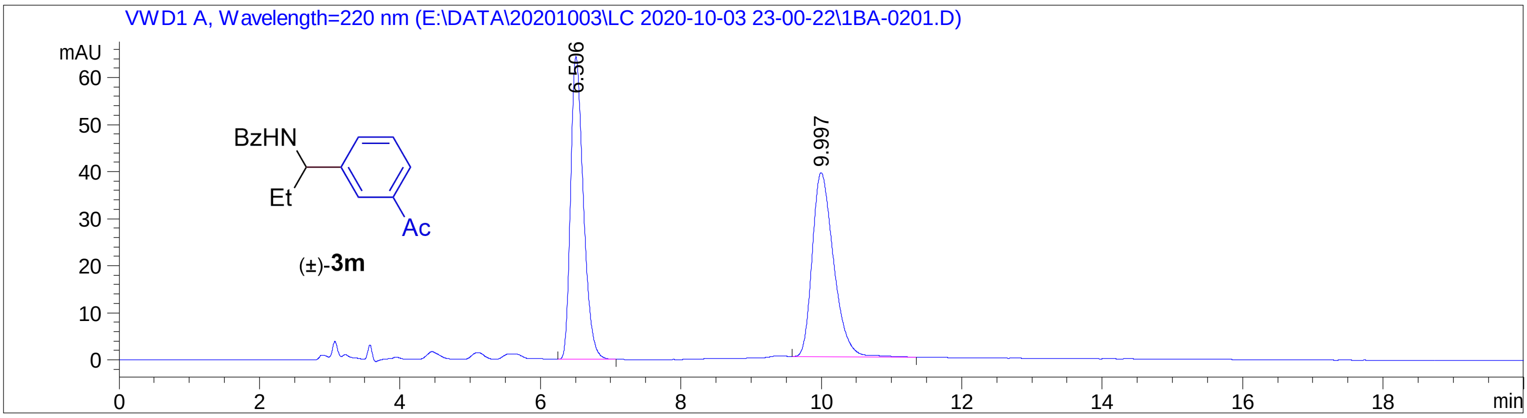

===

Area Percent Report

===

Sorted By : Signal
Multiplier : 1.0000
Dilution : 1.0000
Do not use Multiplier & Dilution Factor with ISTDs

Signal 1: VWD1 A, Wavelength=220 nm

Peak #	RetTime [min]	Type	Width [min]	Area [mAU*s]	Height [mAU]	Area %
1	6.506	BB	0.1938	815.14502	64.35378	49.6515
2	9.997	BB	0.3236	826.58911	39.10279	50.3485

Totals : 1641.73413 103.45656

===

*** End of Report ***

Data File E:\DATA\20201003\LC 2020-10-03 23-00-22\1BB-0301.D
Sample Name: YH-18-11-EE

===

Acq. Operator : SYSTEM Seq. Line : 3
Acq. Instrument : HPLC1260 Location : P1-B2
Injection Date : 10/4/2020 12:03:58 AM Inj : 1
Inj Volume : 3.000 µl
Different Inj Volume from Sample Entry! Actual Inj Volume : 2.000 µl
Acq. Method : E:\DATA\20201003\LC 2020-10-03 23-00-22\20IPA_30_10_2.M
Last changed : 10/3/2020 11:00:51 PM by SYSTEM
Analysis Method : E:\DATA\20201003\LC 2020-10-03 23-00-22\20IPA_30_10_2.M (Sequence Method)
Last changed : 10/4/2020 9:34:03 AM by SYSTEM
(modified after loading)
Additional Info : Peak(s) manually integrated

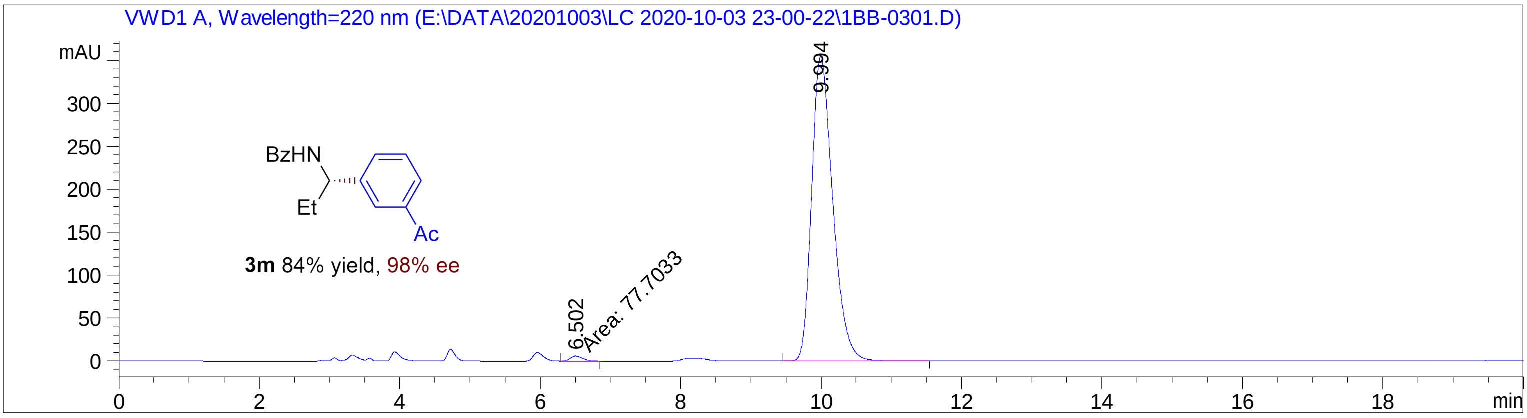

===

Area Percent Report

===

Sorted By : Signal
Multiplier : 1.0000
Dilution : 1.0000
Do not use Multiplier & Dilution Factor with ISTDs

Signal 1: VWD1 A, Wavelength=220 nm

Peak #	RetTime [min]	Type	Width [min]	Area [mAU*s]	Height [mAU]	Area %
1	6.502	MF	0.2115	77.70329	6.12265	1.0378
2	9.994	BB	0.3208	7409.55078	354.52158	98.9622

Totals : 7487.25407 360.64422

===

*** End of Report ***

Data File E:\DATA\20201003\LC 2020-10-05 08-59-28\OnlineEdited--017.D
Sample Name: YH-18-14-RAC

===

Acq. Operator : SYSTEM Seq. Line : 17
Acq. Instrument : HPLC1260 Location : P1-B1
Injection Date : 10/5/2020 2:42:51 PM Inj : 1
Inj Volume : 3.000 µl
Different Inj Volume from Sample Entry! Actual Inj Volume : 1.000 µl
Acq. Method : E:\DATA\20201003\LC 2020-10-05 08-59-28\20IPA_22_10_2.M
Last changed : 10/5/2020 9:52:42 AM by SYSTEM
Analysis Method : E:\DATA\20201003\LC 2020-10-05 08-59-28\20IPA_22_10_2.M (Sequence Method)
Last changed : 10/5/2020 3:11:14 PM by SYSTEM
(modified after loading)
Additional Info : Peak(s) manually integrated

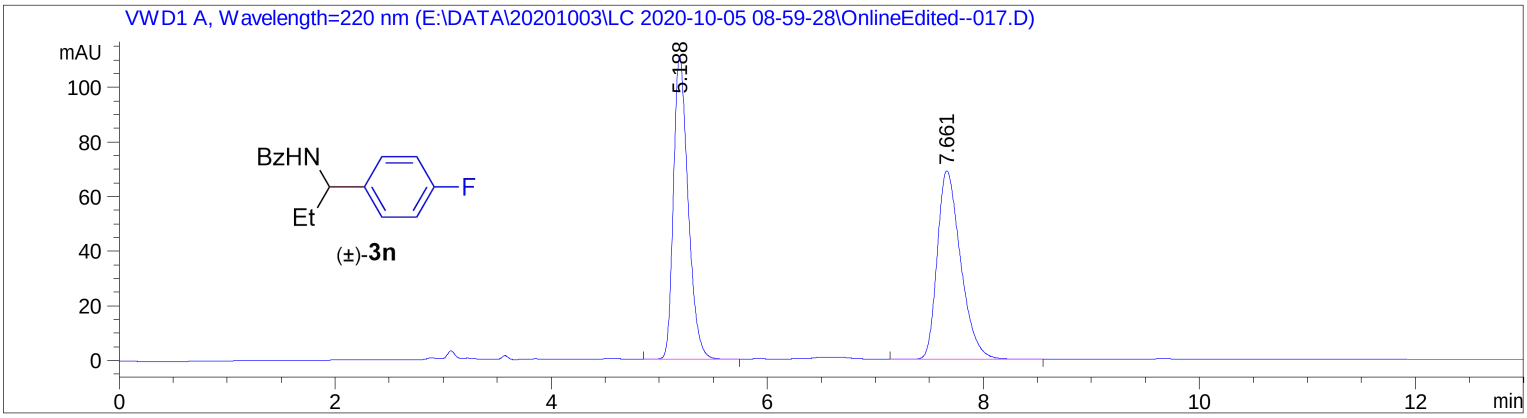

===

Area Percent Report

===

Sorted By : Signal
Multiplier : 1.0000
Dilution : 1.0000
Do not use Multiplier & Dilution Factor with ISTDs

Signal 1: VWD1 A, Wavelength=220 nm

Peak #	RetTime [min]	Type	Width [min]	Area [mAU*s]	Height [mAU]	Area %
1	5.188	BB	0.1450	1044.87891	110.81509	50.1408
2	7.661	BB	0.2296	1039.01001	69.02943	49.8592

Totals : 2083.88892 179.84452

===

*** End of Report ***

===

Acq. Operator : SYSTEM Seq. Line : 18
Acq. Instrument : HPLC1260 Location : P1-B2
Injection Date : 10/5/2020 2:56:35 PM Inj : 1
Inj Volume : 3.000 µl
Different Inj Volume from Sample Entry! Actual Inj Volume : 2.000 µl
Acq. Method : E:\DATA\20201003\LC 2020-10-05 08-59-28\20IPA_22_10_2.M
Last changed : 10/5/2020 9:52:42 AM by SYSTEM
Analysis Method : E:\DATA\20201003\LC 2020-10-05 08-59-28\20IPA_22_10_2.M (Sequence Method)
Last changed : 10/5/2020 3:11:14 PM by SYSTEM
(modified after loading)
Additional Info : Peak(s) manually integrated

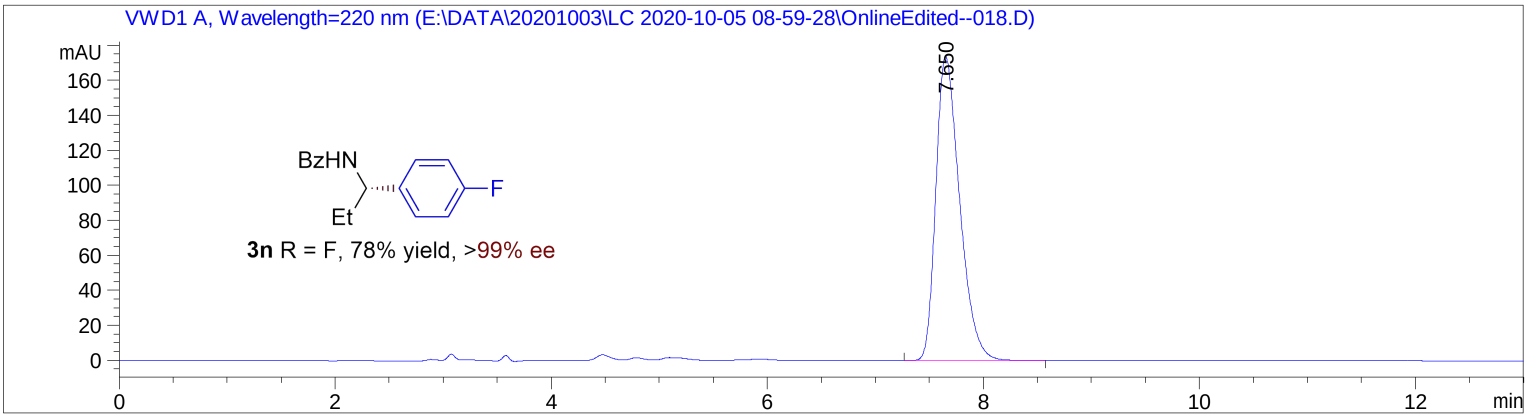

===

Area Percent Report

===

Sorted By : Signal
Multiplier : 1.0000
Dilution : 1.0000
Do not use Multiplier & Dilution Factor with ISTDs

Signal 1: VWD1 A, Wavelength=220 nm

Peak #	RetTime [min]	Type	Width [min]	Area [mAU*s]	Height [mAU]	Area %
1	7.650	BB	0.2347	2660.50854	173.66829	100.0000

Totals : 2660.50854 173.66829

===

*** End of Report ***

Data File E:\DATA\20201003\LC 2020-10-06 07-01-40\OnlineEdited--008.D
Sample Name: YH-18-20-RAC

===

Acq. Operator : SYSTEM Seq. Line : 8
Acq. Instrument : HPLC1260 Location : P1-C9
Injection Date : 10/6/2020 10:37:14 AM Inj : 1
Inj Volume : 3.000 µl
Different Inj Volume from Sample Entry! Actual Inj Volume : 1.000 µl
Acq. Method : E:\DATA\20201003\LC 2020-10-06 07-01-40\20IPA_30_10_2.M
Last changed : 10/6/2020 10:36:50 AM by SYSTEM
(modified after loading)
Analysis Method : E:\DATA\20201003\LC 2020-10-06 07-01-40\20IPA_30_10_2.M (Sequence Method)
Last changed : 10/6/2020 12:26:50 PM by SYSTEM
(modified after loading)
Additional Info : Peak(s) manually integrated

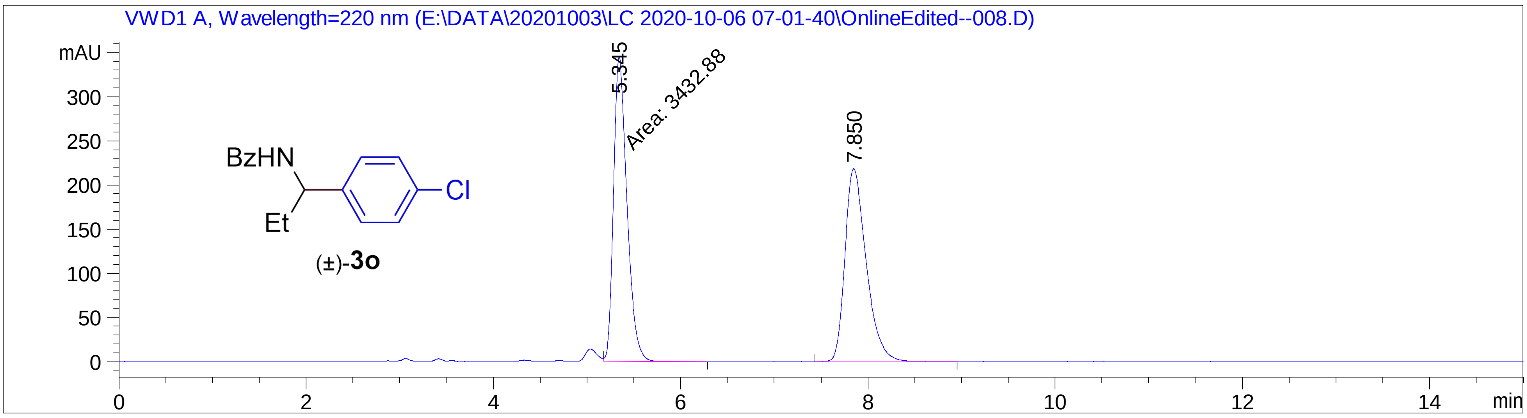

===

Area Percent Report

===

Sorted By : Signal
Multiplier : 1.0000
Dilution : 1.0000
Do not use Multiplier & Dilution Factor with ISTDs

Signal 1: VWD1 A, Wavelength=220 nm

Peak #	RetTime [min]	Type	Width [min]	Area [mAU*s]	Height [mAU]	Area %
1	5.345	FM	0.1657	3432.87842	345.21921	49.9048
2	7.850	BB	0.2401	3445.97168	218.40947	50.0952

Totals : 6878.85010 563.62868

===

*** End of Report ***

===

Acq. Operator : SYSTEM Seq. Line : 9
Acq. Instrument : HPLC1260 Location : P1-C10
Injection Date : 10/6/2020 11:07:59 AM Inj : 1
Inj Volume : 3.000 µl
Different Inj Volume from Sample Entry! Actual Inj Volume : 2.000 µl
Acq. Method : E:\DATA\20201003\LC 2020-10-06 07-01-40\20IPA_30_10_2.M
Last changed : 10/6/2020 10:36:50 AM by SYSTEM
Analysis Method : E:\DATA\20201003\LC 2020-10-06 07-01-40\20IPA_30_10_2.M (Sequence Method)
Last changed : 10/6/2020 12:26:50 PM by SYSTEM
(modified after loading)
Additional Info : Peak(s) manually integrated

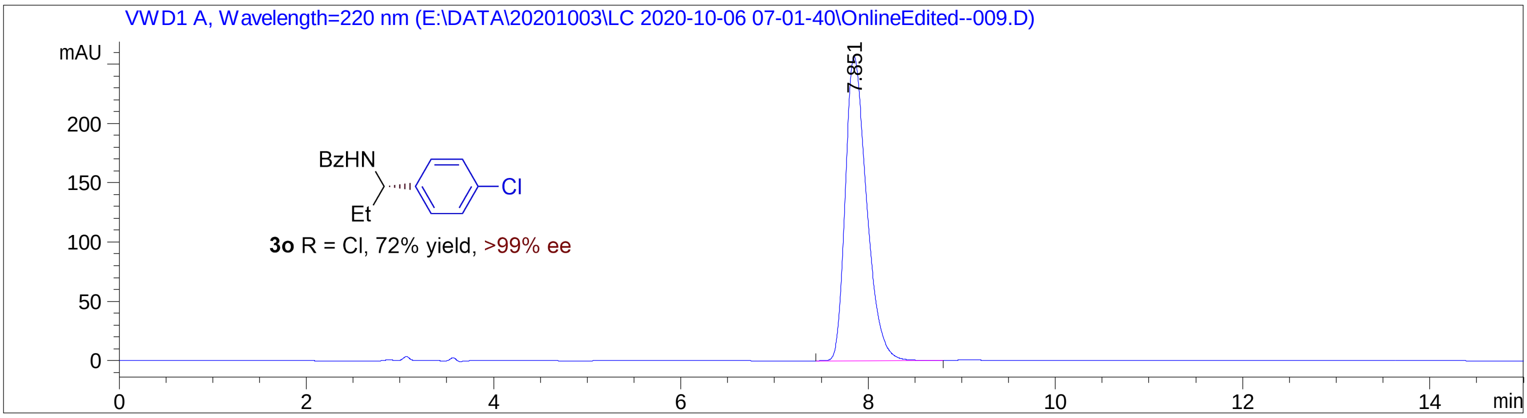

===

Area Percent Report

===

Sorted By : Signal
Multiplier : 1.0000
Dilution : 1.0000
Do not use Multiplier & Dilution Factor with ISTDs

Signal 1: VWD1 A, Wavelength=220 nm

Peak #	RetTime [min]	Type	Width [min]	Area [mAU*s]	Height [mAU]	Area %
1	7.851	BB	0.2418	4063.69604	256.49301	100.0000

Totals : 4063.69604 256.49301

===

*** End of Report ***

Data File E:\DATA\20201003\LC 2020-10-07 22-33-54\OnlineEdited--008.D
Sample Name: YH-18-33-RAC

===

Acq. Operator : SYSTEM   Seq. Line : 8
Acq. Instrument : HPLC1260   Location : P1-A3
Injection Date : 10/8/2020 2:11:34 AM   Inj : 1
Inj Volume : 3.000 µl
Different Inj Volume from Sample Entry! Actual Inj Volume : 1.000 µl
Acq. Method : E:\DATA\20201003\LC 2020-10-07 22-33-54\20IPA_30_10_2.M
Last changed : 10/7/2020 10:36:40 PM by SYSTEM
Analysis Method : E:\DATA\20201003\LC 2020-10-07 22-33-54\20IPA_30_10_2.M (Sequence Method)
Last changed : 10/8/2020 8:09:05 AM by SYSTEM
(modified after loading)
Additional Info : Peak(s) manually integrated

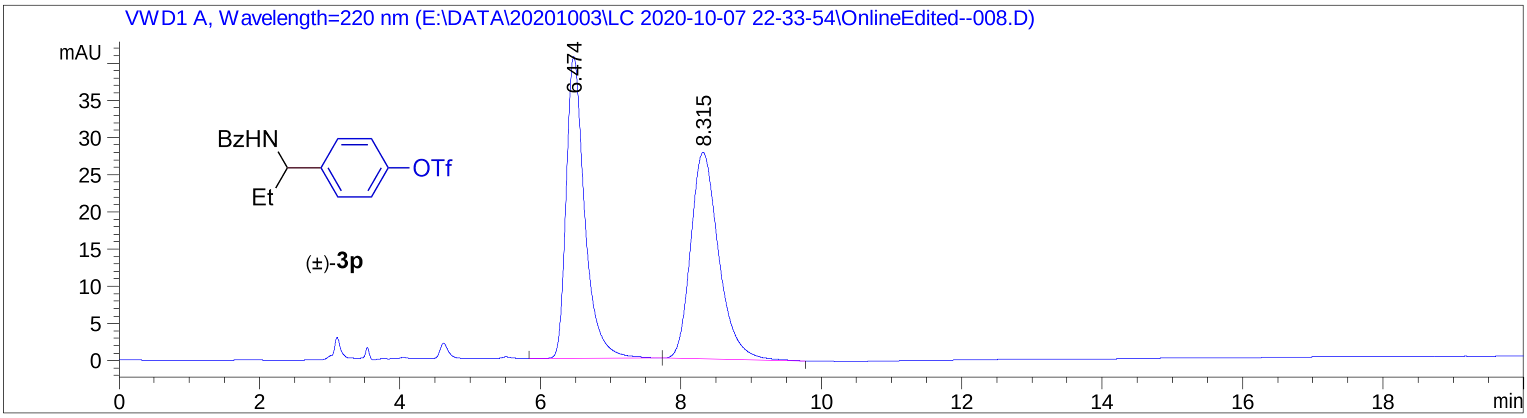

===

Area Percent Report

===

Sorted By : Signal
Multiplier : 1.0000
Dilution : 1.0000
Do not use Multiplier & Dilution Factor with ISTDs

Signal 1: VWD1 A, Wavelength=220 nm

Peak #	RetTime [min]	Type	Width [min]	Area [mAU*s]	Height [mAU]	Area %
1	6.474	BB	0.2755	746.43915	40.59575	49.2161
2	8.315	BB	0.4213	770.21588	27.79106	50.7839

Totals : 1516.65503 68.38681

===

*** End of Report ***

Data File E:\DATA\20201003\LC 2020-10-07 22-33-54\1AD-0501--007.D
Sample Name: YH-18-33-EE

===

Acq. Operator : SYSTEM   Seq. Line : 7
Acq. Instrument : HPLC1260   Location : P1-A4
Injection Date : 10/8/2020 1:40:48 AM   Inj : 1
Inj Volume : 3.000 µl
Different Inj Volume from Sample Entry! Actual Inj Volume : 2.000 µl
Acq. Method : E:\DATA\20201003\LC 2020-10-07 22-33-54\20IPA_30_10_2.M
Last changed : 10/7/2020 10:36:40 PM by SYSTEM
Analysis Method : E:\DATA\20201003\LC 2020-10-07 22-33-54\20IPA_30_10_2.M (Sequence Method)
Last changed : 10/8/2020 8:09:52 AM by SYSTEM
(modified after loading)
Additional Info : Peak(s) manually integrated

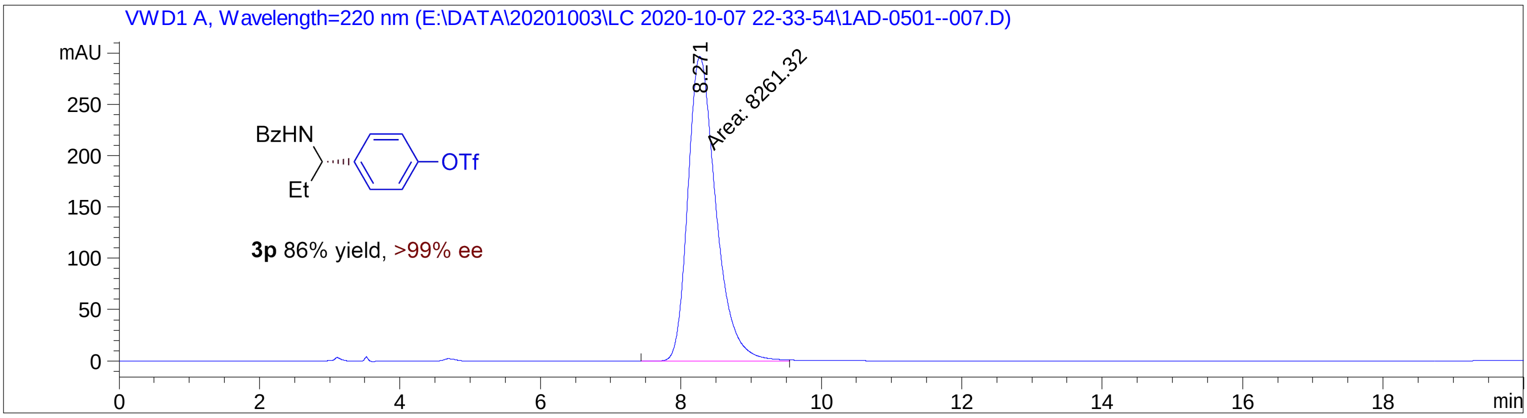

===

Area Percent Report

===

Sorted By : Signal
Multiplier : 1.0000
Dilution : 1.0000
Do not use Multiplier & Dilution Factor with ISTDs

Signal 1: VWD1 A, Wavelength=220 nm

Peak #	RetTime [min]	Type	Width [min]	Area [mAU*s]	Height [mAU]	Area %
1	8.271	MF	0.4639	8261.32031	296.78357	100.0000

Totals :	8261.32031	296.78357

===

*** End of Report ***

Data File E:\DATA\20201003\LC 2020-10-03 23-00-22\1BC-0401.D
Sample Name: YH-18-12-RAC

===

Acq. Operator : SYSTEM Seq. Line : 4
Acq. Instrument : HPLC1260 Location : P1-B3
Injection Date : 10/4/2020 12:34:43 AM Inj : 1
Inj Volume : 3.000 µl
Different Inj Volume from Sample Entry! Actual Inj Volume : 1.000 µl
Acq. Method : E:\DATA\20201003\LC 2020-10-03 23-00-22\20IPA_30_10_2.M
Last changed : 10/3/2020 11:00:51 PM by SYSTEM
Analysis Method : E:\DATA\20201003\LC 2020-10-03 23-00-22\20IPA_30_10_2.M (Sequence Method)
Last changed : 10/4/2020 9:35:24 AM by SYSTEM
(modified after loading)
Additional Info : Peak(s) manually integrated

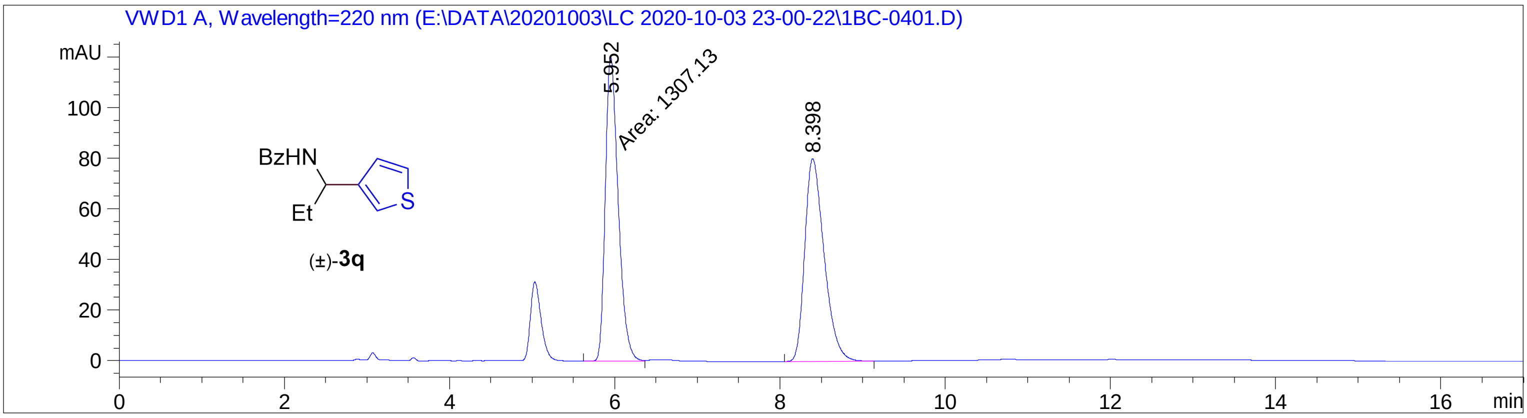

===

Area Percent Report

===

Sorted By : Signal
Multiplier : 1.0000
Dilution : 1.0000
Do not use Multiplier & Dilution Factor with ISTDs

Signal 1: VWD1 A, Wavelength=220 nm

Peak #	RetTime [min]	Type	Width [min]	Area [mAU*s]	Height [mAU]	Area %
1	5.952	MF	0.1811	1307.12964	120.26933	50.0582
2	8.398	BB	0.2491	1304.08838	80.03413	49.9418

Totals : 2611.21802 200.30347

===

*** End of Report ***

Data File E:\DATA\20201003\LC 2020-10-03 23-00-22\1BD-0501.D
Sample Name: YH-18-12-EE

===

Acq. Operator : SYSTEM Seq. Line : 5
Acq. Instrument : HPLC1260 Location : P1-B4
Injection Date : 10/4/2020 1:05:28 AM Inj : 1
Inj Volume : 3.000 µl
Different Inj Volume from Sample Entry! Actual Inj Volume : 2.000 µl
Acq. Method : E:\DATA\20201003\LC 2020-10-03 23-00-22\20IPA_30_10_2.M
Last changed : 10/3/2020 11:00:51 PM by SYSTEM
Analysis Method : E:\DATA\20201003\LC 2020-10-03 23-00-22\20IPA_30_10_2.M (Sequence Method)
Last changed : 10/4/2020 9:35:24 AM by SYSTEM
(modified after loading)
Additional Info : Peak(s) manually integrated

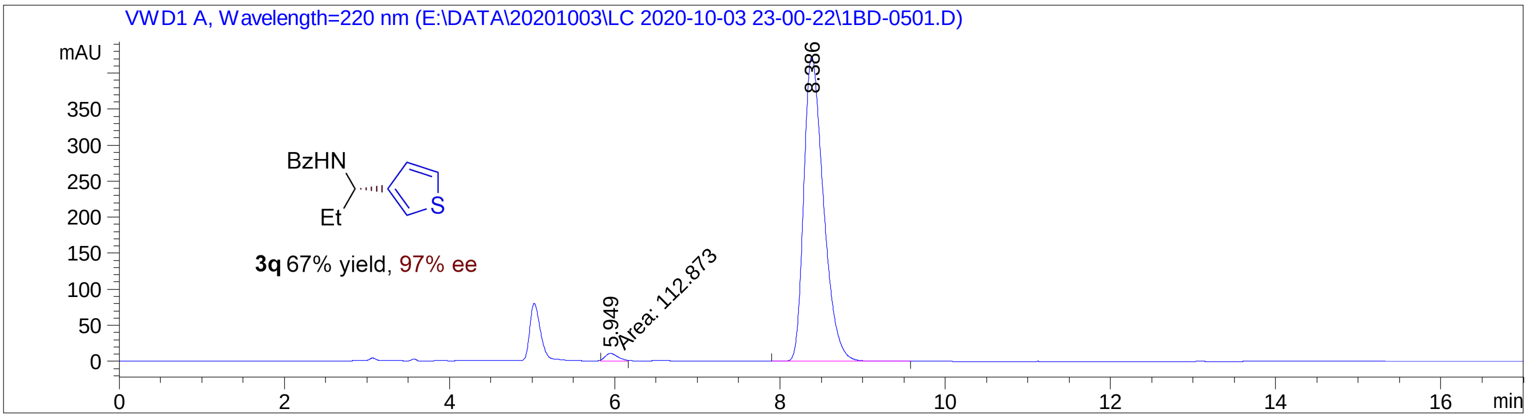

===

Area Percent Report

===

Sorted By : Signal
Multiplier : 1.0000
Dilution : 1.0000
Do not use Multiplier & Dilution Factor with ISTDs

Signal 1: VWD1 A, Wavelength=220 nm

Peak #	RetTime [min]	Type	Width [min]	Area [mAU*s]	Height [mAU]	Area %
1	5.949	FM	0.1789	112.87334	10.51379	1.5848
2	8.386	BB	0.2534	7009.30908	422.81552	98.4152

Totals : 7122.18242 433.32931

===

*** End of Report ***

Data File E:\DATA\20201003\LC 2020-10-07 00-19-54\1BC-0401.D
Sample Name: YH-18-30-RAC

===

Acq. Operator : SYSTEM Seq. Line : 4
Acq. Instrument : HPLC1260 Location : P1-B3
Injection Date : 10/7/2020 1:54:16 AM Inj : 1
Inj Volume : 3.000 µl
Different Inj Volume from Sample Entry! Actual Inj Volume : 1.000 µl
Acq. Method : E:\DATA\20201003\LC 2020-10-07 00-19-54\20IPA_30_10_2.M
Last changed : 10/7/2020 12:19:54 AM by SYSTEM
Analysis Method : E:\DATA\20201003\LC 2020-10-07 00-19-54\20IPA_30_10_2.M (Sequence Method)
Last changed : 10/7/2020 8:15:00 AM by SYSTEM
(modified after loading)
Additional Info : Peak(s) manually integrated

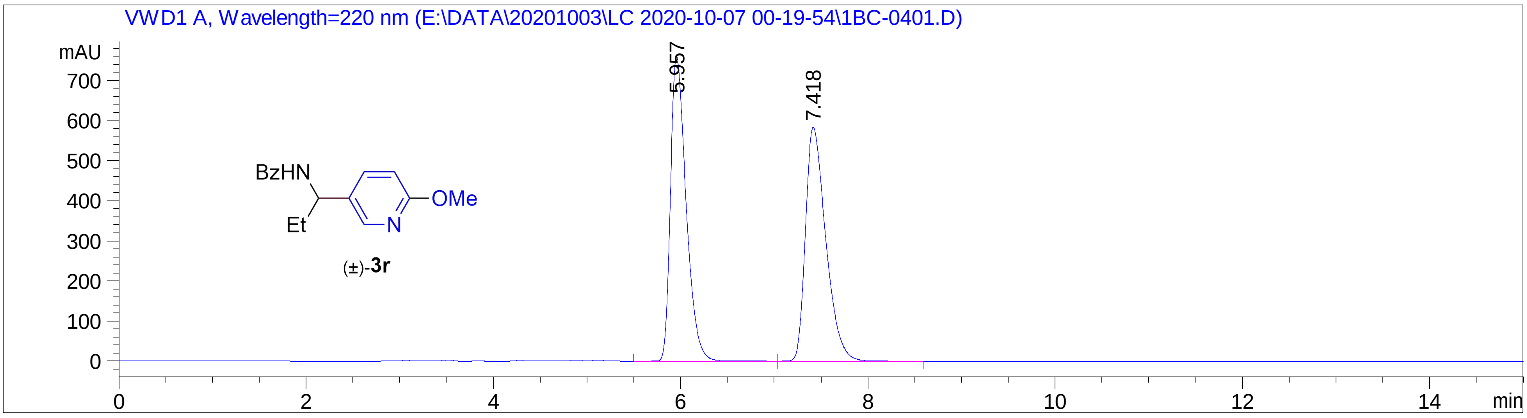

===

Area Percent Report

===

Sorted By : Signal
Multiplier : 1.0000
Dilution : 1.0000
Do not use Multiplier & Dilution Factor with ISTDs

Signal 1: VWD1 A, Wavelength=220 nm

Peak #	RetTime [min]	Type	Width [min]	Area [mAU*s]	Height [mAU]	Area %
1	5.957	BB	0.1772	8819.35352	760.82288	50.0365
2	7.418	BB	0.2310	8806.48535	583.85815	49.9635

Totals : 1.76258e4 1344.68103

===

*** End of Report ***

Data File E:\DATA\20201003\LC 2020-10-07 00-19-54\1BD-0501.D
Sample Name: YH-18-30-EE

===

Acq. Operator : SYSTEM   Seq. Line : 5
Acq. Instrument : HPLC1260   Location : P1-B4
Injection Date : 10/7/2020 2:25:01 AM   Inj : 1
Inj Volume : 3.000 µl
Different Inj Volume from Sample Entry! Actual Inj Volume : 2.000 µl
Acq. Method : E:\DATA\20201003\LC 2020-10-07 00-19-54\20IPA_30_10_2.M
Last changed : 10/7/2020 12:19:54 AM by SYSTEM
Analysis Method : E:\DATA\20201003\LC 2020-10-07 00-19-54\20IPA_30_10_2.M (Sequence Method)
Last changed : 10/7/2020 8:15:39 AM by SYSTEM
(modified after loading)
Additional Info : Peak(s) manually integrated

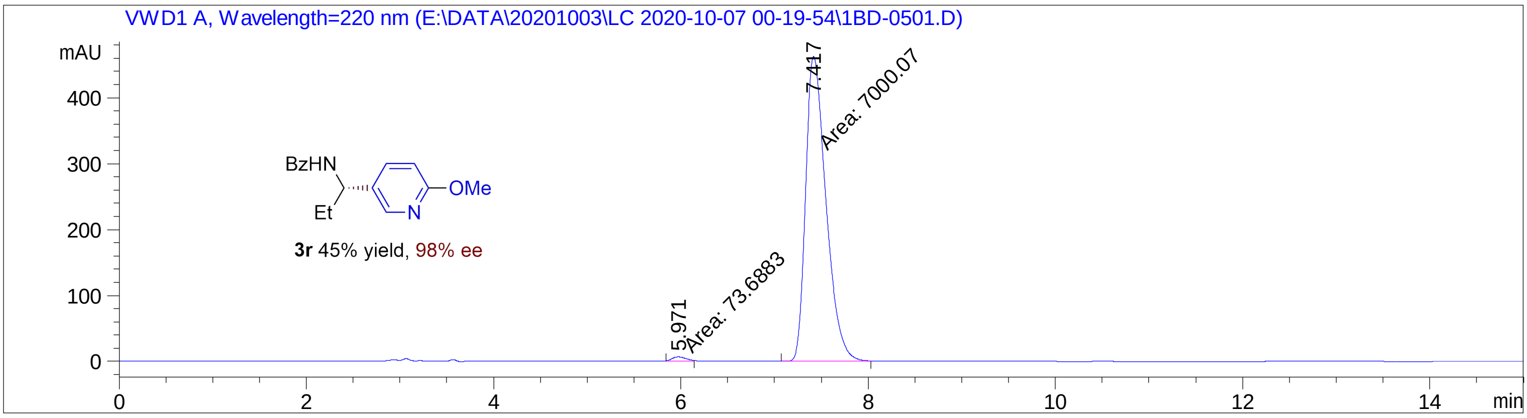

===

Area Percent Report

===

Sorted By : Signal
Multiplier : 1.0000
Dilution : 1.0000
Do not use Multiplier & Dilution Factor with ISTDs

Signal 1: VWD1 A, Wavelength=220 nm

Peak #	RetTime [min]	Type	Width [min]	Area [mAU*s]	Height [mAU]	Area %
1	5.971	FM	0.1803	73.68832	6.81036	1.0417
2	7.417	MF	0.2523	7000.07080	462.46750	98.9583

Totals : 7073.75912 469.27786

===

*** End of Report ***

Data File E:\DATA\20201003\LC 2020-10-06 07-01-40\OnlineEdited--002.D
Sample Name: YH-18-17-RAC

===

Acq. Operator : SYSTEM Seq. Line : 2
Acq. Instrument : HPLC1260 Location : P1-C3
Injection Date : 10/6/2020 7:34:30 AM Inj : 1
Inj Volume : 3.000 µl
Different Inj Volume from Sample Entry! Actual Inj Volume : 1.000 µl
Acq. Method : E:\DATA\20201003\LC 2020-10-06 07-01-40\20IPA_30_10_2.M
Last changed : 10/6/2020 7:03:10 AM by SYSTEM
Analysis Method : E:\DATA\20201003\LC 2020-10-06 07-01-40\20IPA_30_10_2.M (Sequence Method)
Last changed : 10/6/2020 9:08:57 AM by SYSTEM
(modified after loading)
Additional Info : Peak(s) manually integrated

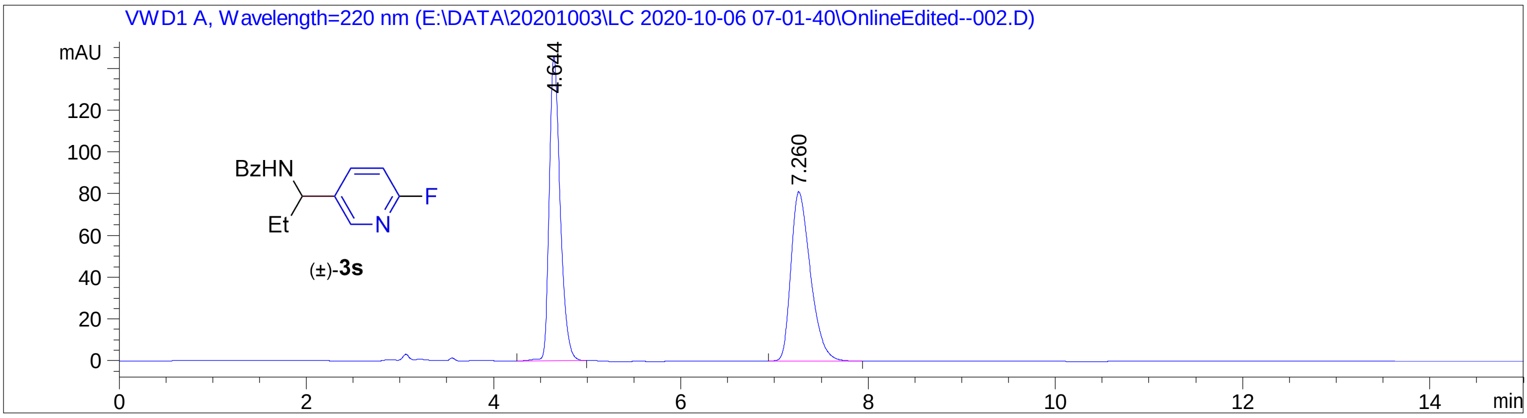

===

Area Percent Report

===

Sorted By : Signal
Multiplier : 1.0000
Dilution : 1.0000
Do not use Multiplier & Dilution Factor with ISTDs

Signal 1: VWD1 A, Wavelength=220 nm

Peak #	RetTime [min]	Type	Width [min]	Area [mAU*s]	Height [mAU]	Area %
1	4.644	BB	0.1221	1175.74829	145.74675	50.0679
2	7.260	BB	0.2218	1172.56042	81.05724	49.9321

Totals : 2348.30872 226.80399

===

*** End of Report ***

Data File E:\DATA\20201003\LC 2020-10-06 07-01-40\OnlineEdited--003.D
Sample Name: YH-18-17-EE

===

Acq. Operator : SYSTEM Seq. Line : 3
Acq. Instrument : HPLC1260 Location : P1-C4
Injection Date : 10/6/2020 8:05:15 AM Inj : 1
Inj Volume : 3.000 µl
Different Inj Volume from Sample Entry! Actual Inj Volume : 2.000 µl
Acq. Method : E:\DATA\20201003\LC 2020-10-06 07-01-40\20IPA_30_10_2.M
Last changed : 10/6/2020 7:03:10 AM by SYSTEM
Analysis Method : E:\DATA\20201003\LC 2020-10-06 07-01-40\20IPA_30_10_2.M (Sequence Method)
Last changed : 10/6/2020 9:08:57 AM by SYSTEM
(modified after loading)
Additional Info : Peak(s) manually integrated

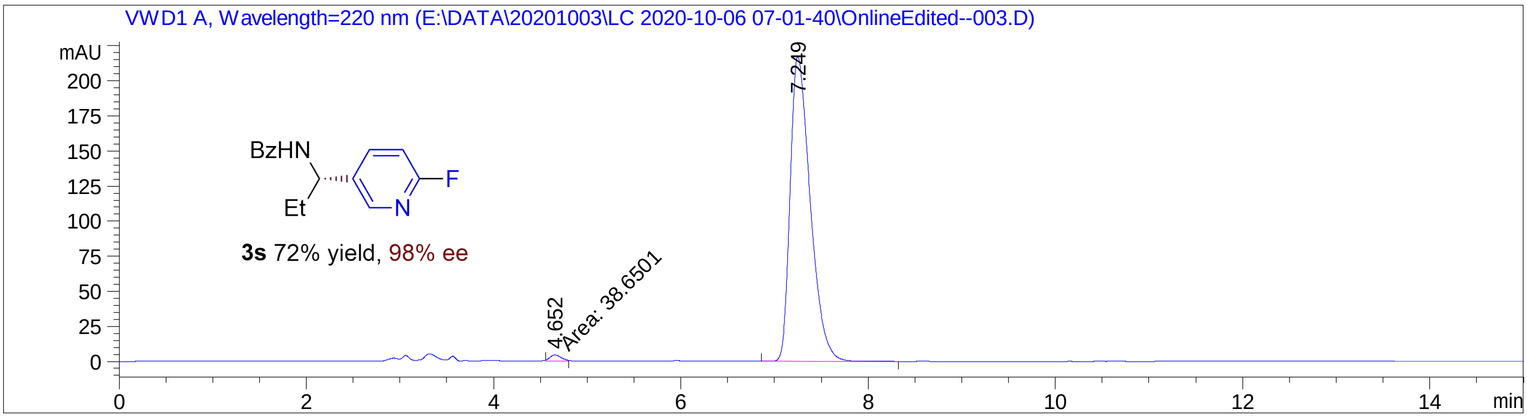

===

Area Percent Report

===

Sorted By : Signal
Multiplier : 1.0000
Dilution : 1.0000
Do not use Multiplier & Dilution Factor with ISTDs

Signal 1: VWD1 A, Wavelength=220 nm

Peak #	RetTime [min]	Type	Width [min]	Area [mAU*s]	Height [mAU]	Area %
1	4.652	FM	0.1441	38.65009	4.46888	1.1793
2	7.249	BB	0.2289	3238.75659	217.25542	98.8207

Totals : 3277.40668 221.72430

===

*** End of Report ***

Data File E:\DATA\20201003\LC 2020-10-07 22-33-54\OnlineEdited--024.D
Sample Name: YH-18-35-RAC

===

Acq. Operator : SYSTEM Seq. Line : 24
Acq. Instrument : HPLC1260 Location : P1-A5
Injection Date : 10/8/2020 10:13:43 AM Inj : 1
Inj Volume : 3.000 µl
Different Inj Volume from Sample Entry! Actual Inj Volume : 1.000 µl
Acq. Method : E:\DATA\20201003\LC 2020-10-07 22-33-54\20IPA_30_10_2.M
Last changed : 10/8/2020 10:24:28 AM by SYSTEM
(modified after loading)
Analysis Method : E:\DATA\20201003\LC 2020-10-07 22-33-54\20IPA_30_10_2.M (Sequence Method)
Last changed : 10/8/2020 10:32:35 AM by SYSTEM
(modified after loading)
Additional Info : Peak(s) manually integrated

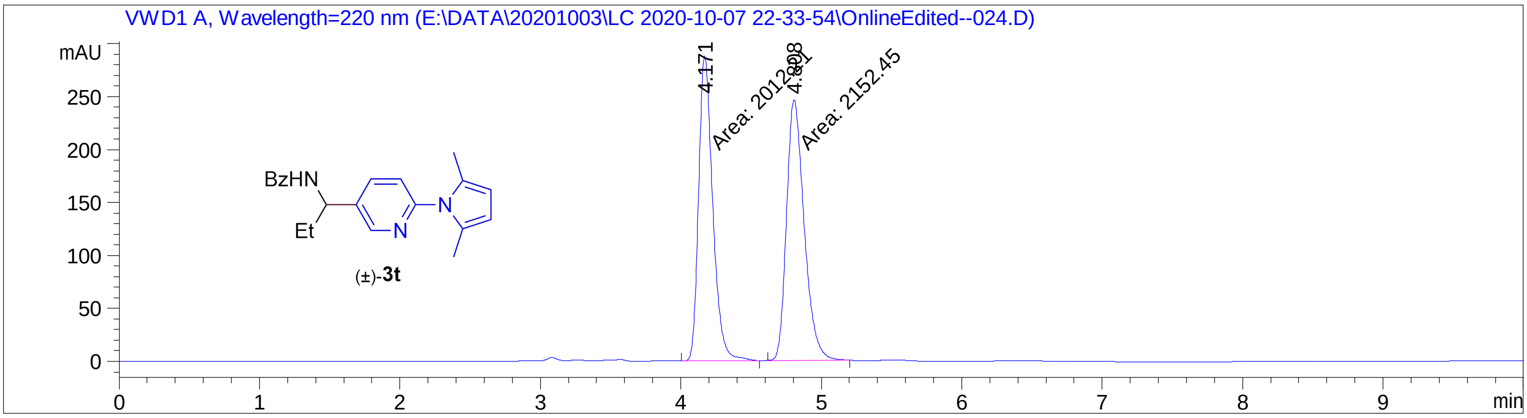

===
Area Percent Report
===

Sorted By : Signal
Multiplier : 1.0000
Dilution : 1.0000
Do not use Multiplier & Dilution Factor with ISTDs

Signal 1: VWD1 A, Wavelength=220 nm

Peak #	RetTime [min]	Type	Width [min]	Area [mAU*s]	Height [mAU]	Area %
1	4.171	FM	0.1167	2012.20569	287.47464	48.3163
2	4.808	MM	0.1457	2152.44873	246.18887	51.6837

Totals : 4164.65442 533.66351

===
*** End of Report ***

===

Acq. Operator : SYSTEM Seq. Line : 25
Acq. Instrument : HPLC1260 Location : P1-A6
Injection Date : 10/8/2020 10:25:12 AM Inj : 1
Inj Volume : 3.000 µl
Different Inj Volume from Sample Entry! Actual Inj Volume : 2.000 µl
Acq. Method : E:\DATA\20201003\LC 2020-10-07 22-33-54\20IPA_30_10_2.M
Last changed : 10/8/2020 10:24:28 AM by SYSTEM
Analysis Method : E:\DATA\20201003\LC 2020-10-07 22-33-54\20IPA_30_10_2.M (Sequence Method)
Last changed : 10/8/2020 10:24:35 AM by SYSTEM
Additional Info : Peak(s) manually integrated

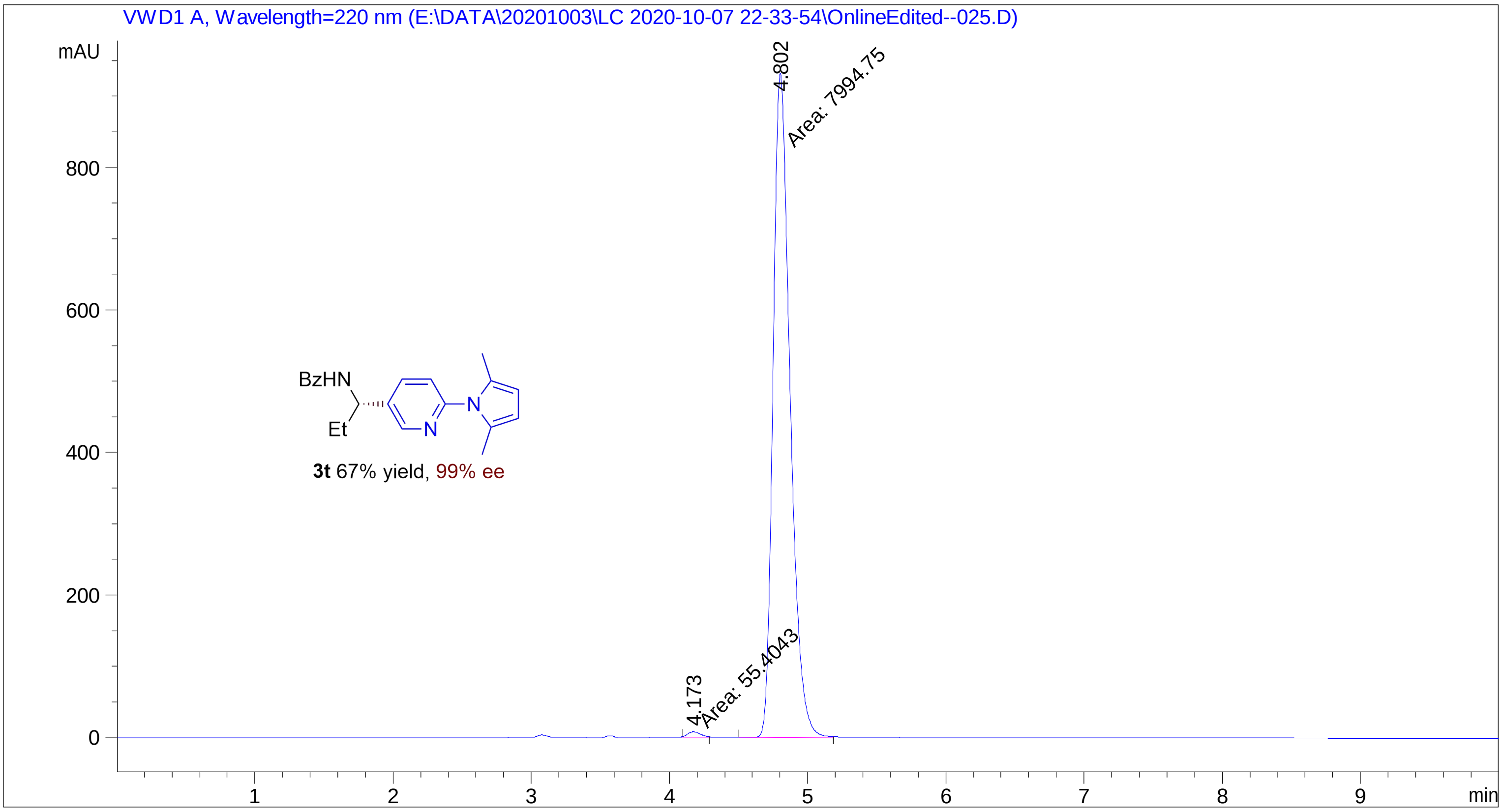

===
Area Percent Report
===

Sorted By : Signal
Multiplier : 1.0000
Dilution : 1.0000
Use Multiplier & Dilution Factor with ISTDs

Signal 1: VWD1 A, Wavelength=220 nm

Peak #	RetTime [min]	Type	Width [min]	Area [mAU*s]	Height [mAU]	Area %
1	4.173	MF	0.1107	55.40433	8.34066	0.6882
2	4.802	MF	0.1430	7994.75293	932.11047	99.3118

Totals : 8050.15726 940.45114

===

Data File E:\DATA\20201003\LC 2020-10-07 22-33-54\1AG-0801--011.D
Sample Name: YH-18-36-RAC

```
=====================================================================
Acq. Operator   : SYSTEM                           Seq. Line :  11
Acq. Instrument : HPLC1260                          Location :   P1-A7
Injection Date  : 10/8/2020 3:43:48 AM                   Inj :   1
                                                  Inj Volume : 3.000 µl
Different Inj Volume from Sample Entry!  Actual Inj Volume : 1.000 µl
Acq. Method     : E:\DATA\20201003\LC 2020-10-07 22-33-54\20IPA_30_10_2.M
Last changed    : 10/7/2020 10:36:40 PM by SYSTEM
Analysis Method : E:\DATA\20201003\LC 2020-10-07 22-33-54\20IPA_30_10_2.M (Sequence Method)
Last changed    : 10/8/2020 8:10:53 AM by SYSTEM
                  (modified after loading)
Additional Info : Peak(s) manually integrated
```

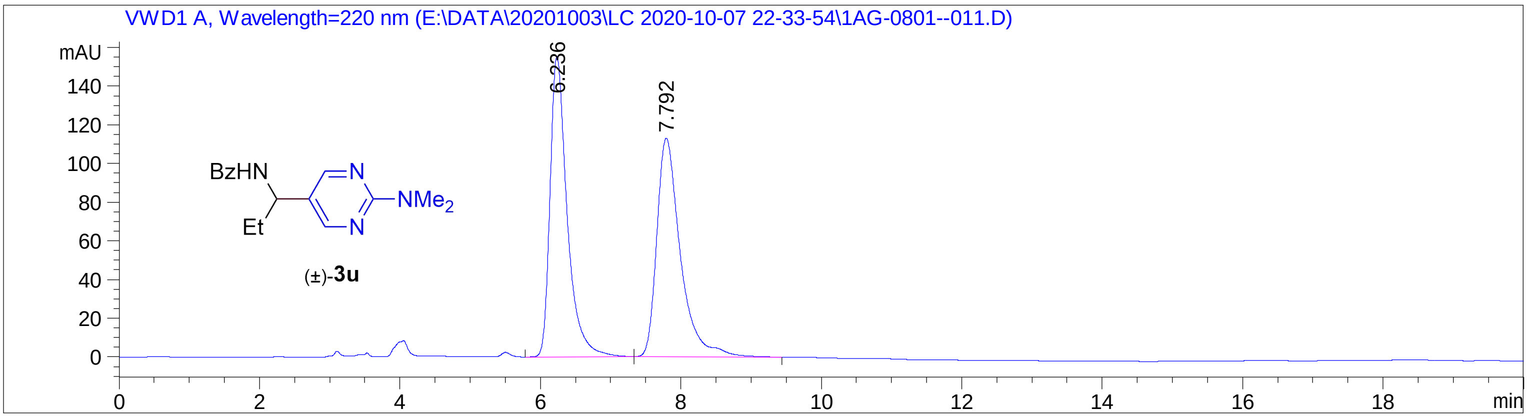


```
=====================================================================
                        Area Percent Report
=====================================================================

Sorted By             :      Signal
Multiplier            :      1.0000
Dilution              :      1.0000
Do not use Multiplier & Dilution Factor with ISTDs


Signal 1: VWD1 A, Wavelength=220 nm
```

Peak #	RetTime [min]	Type	Width [min]	Area [mAU*s]	Height [mAU]	Area %
1	6.236	BB	0.2536	2630.12305	155.27097	49.8803
2	7.792	BB	0.3497	2642.74268	113.05434	50.1197

Totals : 5272.86572 268.32530

```
=====================================================================
                         *** End of Report ***
```

Data File E:\DATA\20201003\LC 2020-10-07 22-33-54\1AH-0901--012.D
Sample Name: YH-18-36-EE

===

Acq. Operator : SYSTEM Seq. Line : 12
Acq. Instrument : HPLC1260 Location : P1-A8
Injection Date : 10/8/2020 4:14:34 AM Inj : 1
Inj Volume : 3.000 µl
Different Inj Volume from Sample Entry! Actual Inj Volume : 2.000 µl
Acq. Method : E:\DATA\20201003\LC 2020-10-07 22-33-54\20IPA_30_10_2.M
Last changed : 10/7/2020 10:36:40 PM by SYSTEM
Analysis Method : E:\DATA\20201003\LC 2020-10-07 22-33-54\20IPA_30_10_2.M (Sequence Method)
Last changed : 10/8/2020 8:11:39 AM by SYSTEM
(modified after loading)
Additional Info : Peak(s) manually integrated

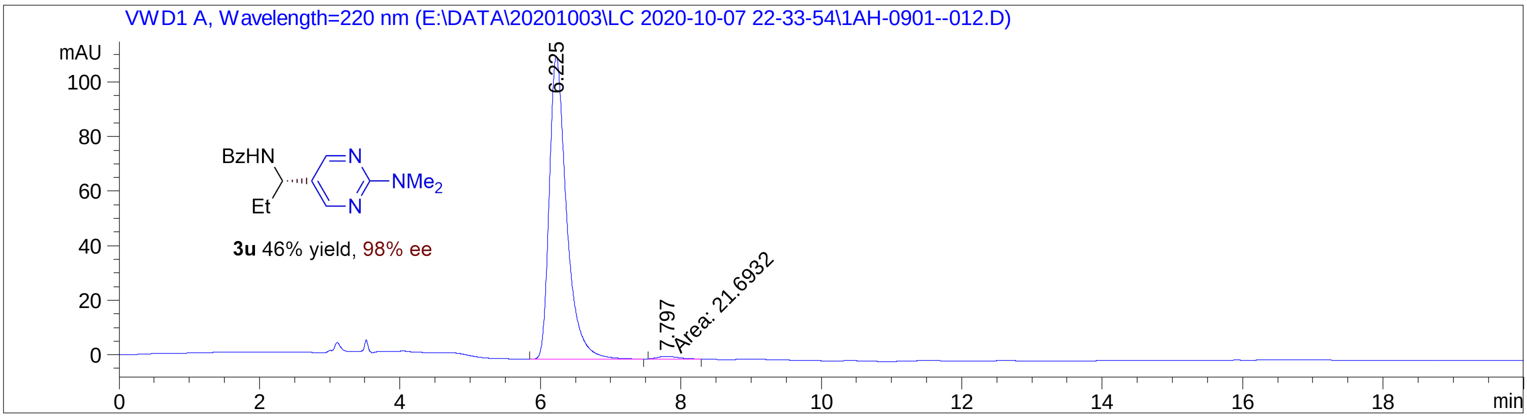

===

Area Percent Report

===

Sorted By : Signal
Multiplier : 1.0000
Dilution : 1.0000
Do not use Multiplier & Dilution Factor with ISTDs

Signal 1: VWD1 A, Wavelength=220 nm

Peak #	RetTime [min]	Type	Width [min]	Area [mAU*s]	Height [mAU]	Area %
1	6.225	BB	0.2580	1901.68115	110.95705	98.8721
2	7.797	MM	0.3506	21.69323	1.03134	1.1279

Totals : 1923.37438 111.98839

===

*** End of Report ***

Data File E:\DATA\20201003\LC 2020-10-07 22-33-54\OnlineEdited--026.D
Sample Name: YH-18-37-RAC

===
Acq. Operator : SYSTEM Seq. Line : 26
Acq. Instrument : HPLC1260 Location : P1-A9
Injection Date : 10/8/2020 10:35:57 AM Inj : 1
Inj Volume : 3.000 µl
Different Inj Volume from Sample Entry! Actual Inj Volume : 1.000 µl
Acq. Method : E:\DATA\20201003\LC 2020-10-07 22-33-54\20IPA_30_10_2.M
Last changed : 10/8/2020 10:55:23 AM by SYSTEM
(modified after loading)
Analysis Method : E:\DATA\20201003\LC 2020-10-07 22-33-54\20IPA_30_10_2.M (Sequence Method)
Last changed : 10/8/2020 11:19:07 AM by SYSTEM
(modified after loading)
Additional Info : Peak(s) manually integrated

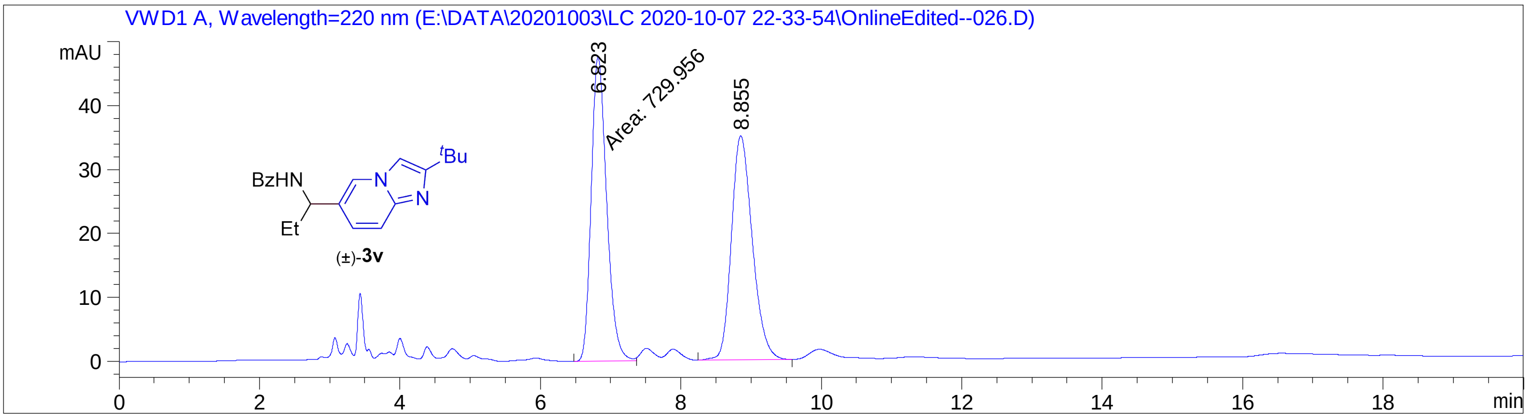

===
Area Percent Report
===

Sorted By : Signal
Multiplier : 1.0000
Dilution : 1.0000
Do not use Multiplier & Dilution Factor with ISTDs

Signal 1: VWD1 A, Wavelength=220 nm

Peak #	RetTime [min]	Type	Width [min]	Area [mAU*s]	Height [mAU]	Area %
1	6.823	MF	0.2550	729.95618	47.71666	50.0698
2	8.855	BB	0.3189	727.92230	35.10399	49.9302
Totals :				1457.87848	82.82064	

===
*** End of Report ***

Data File E:\DATA\20201003\LC 2020-10-07 22-33-54\OnlineEdited--027.D
Sample Name: YH-18-37-EE

===

Acq. Operator : SYSTEM Seq. Line : 27
Acq. Instrument : HPLC1260 Location : P1-A10
Injection Date : 10/8/2020 10:56:43 AM Inj : 1
Inj Volume : 3.000 µl
Different Inj Volume from Sample Entry! Actual Inj Volume : 2.000 µl
Acq. Method : E:\DATA\20201003\LC 2020-10-07 22-33-54\20IPA_30_10_2.M
Last changed : 10/8/2020 10:55:23 AM by SYSTEM
Analysis Method : E:\DATA\20201003\LC 2020-10-07 22-33-54\20IPA_30_10_2.M (Sequence Method)
Last changed : 10/8/2020 11:19:07 AM by SYSTEM
(modified after loading)
Additional Info : Peak(s) manually integrated

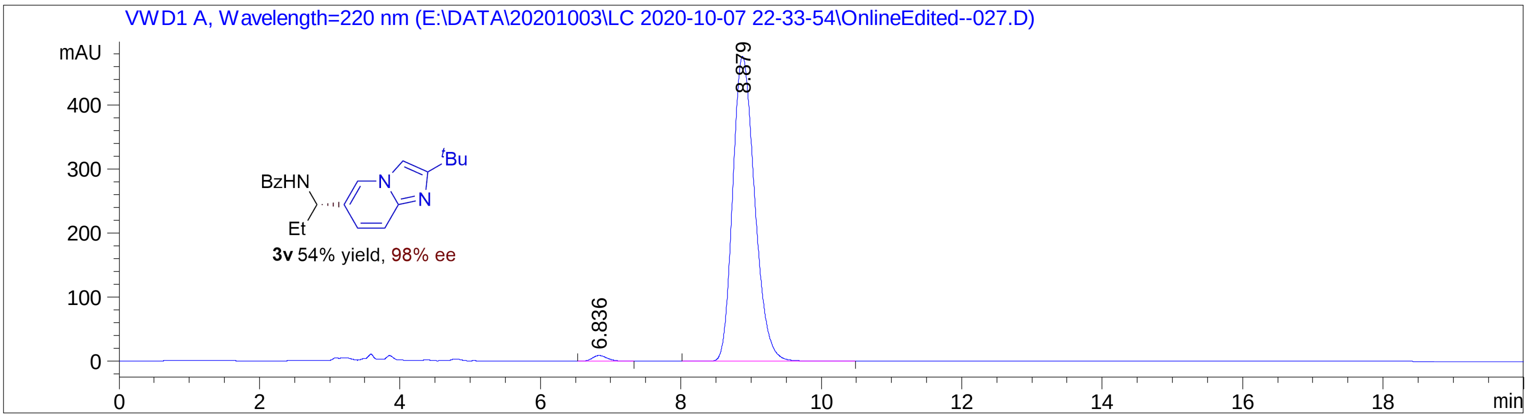

===

Area Percent Report

===

Sorted By : Signal
Multiplier : 1.0000
Dilution : 1.0000
Do not use Multiplier & Dilution Factor with ISTDs

Signal 1: VWD1 A, Wavelength=220 nm

Peak #	RetTime [min]	Type	Width [min]	Area [mAU*s]	Height [mAU]	Area %
1	6.836	BB	0.2317	134.62772	8.99253	1.2870
2	8.879	BB	0.3383	1.03259e4	475.59143	98.7130

Totals : 1.04605e4 484.58396

===

*** End of Report ***

Data File E:\DATA\20201003\LC 2020-10-07 22-33-54\OnlineEdited--039.D
Sample Name: YH-18-40-RAC

===
Acq. Operator : SYSTEM Seq. Line : 39
Acq. Instrument : HPLC1260 Location : P1-A11
Injection Date : 10/8/2020 3:18:18 PM Inj : 1
Inj Volume : 3.000 µl
Different Inj Volume from Sample Entry! Actual Inj Volume : 1.000 µl
Acq. Method : E:\DATA\20201003\LC 2020-10-07 22-33-54\30IPA_30_10_2.M
Last changed : 10/8/2020 11:54:16 AM by SYSTEM
Analysis Method : E:\DATA\20201003\LC 2020-10-07 22-33-54\30IPA_30_10_2.M (Sequence Method)
Last changed : 10/9/2020 12:37:32 AM by SYSTEM
(modified after loading)
Additional Info : Peak(s) manually integrated

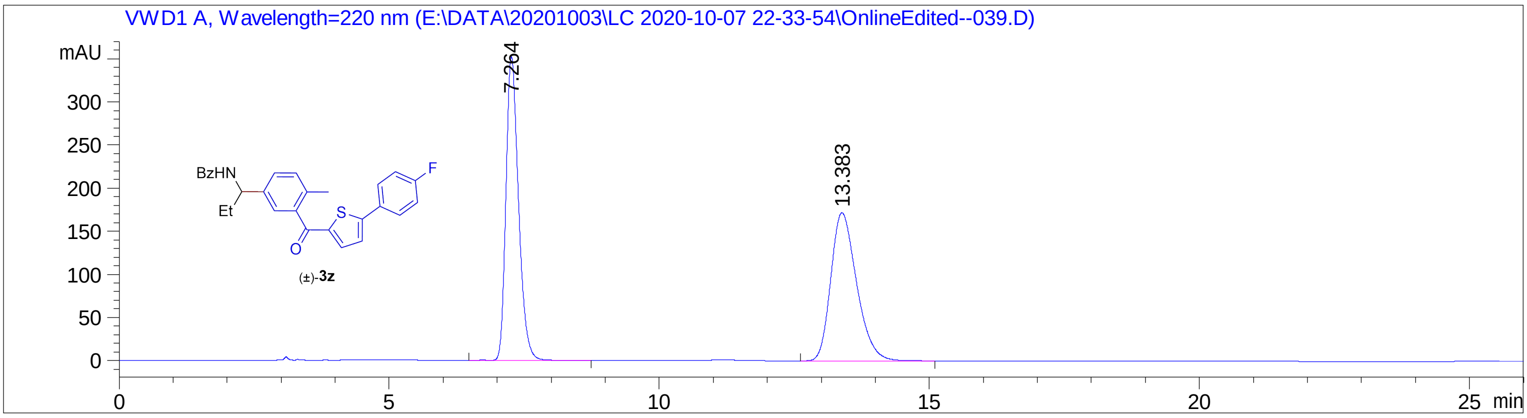

===
Area Percent Report
===

Sorted By : Signal
Multiplier : 1.0000
Dilution : 1.0000
Do not use Multiplier & Dilution Factor with ISTDs

Signal 1: VWD1 A, Wavelength=220 nm

Peak #	RetTime [min]	Type	Width [min]	Area [mAU*s]	Height [mAU]	Area %
1	7.264	VB R	0.2476	5697.72070	352.36447	49.9718
2	13.383	BB	0.5093	5704.16016	171.80746	50.0282

Totals : 1.14019e4 524.17194

===
*** End of Report ***

Data File E:\DATA\20201003\LC 2020-10-07 22-33-54\OnlineEdited--040.D
Sample Name: YH-18-40-EE

===

Acq. Operator : SYSTEM Seq. Line : 40
Acq. Instrument : HPLC1260 Location : P1-B1
Injection Date : 10/8/2020 3:49:06 PM Inj : 1
Inj Volume : 3.000 µl
Different Inj Volume from Sample Entry! Actual Inj Volume : 2.000 µl
Acq. Method : E:\DATA\20201003\LC 2020-10-07 22-33-54\30IPA_30_10_2.M
Last changed : 10/8/2020 11:54:16 AM by SYSTEM
Analysis Method : E:\DATA\20201003\LC 2020-10-07 22-33-54\30IPA_30_10_2.M (Sequence Method)
Last changed : 10/9/2020 12:37:32 AM by SYSTEM
(modified after loading)
Additional Info : Peak(s) manually integrated

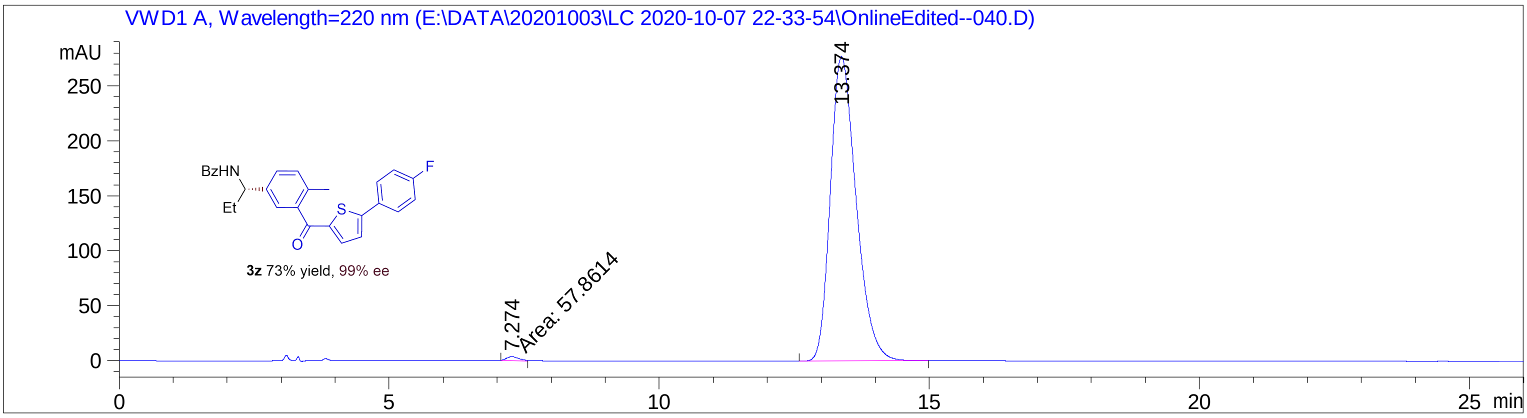

===

Area Percent Report

===

Sorted By : Signal
Multiplier : 1.0000
Dilution : 1.0000
Do not use Multiplier & Dilution Factor with ISTDs

Signal 1: VWD1 A, Wavelength=220 nm

Peak #	RetTime [min]	Type	Width [min]	Area [mAU*s]	Height [mAU]	Area %
1	7.274	FM	0.2624	57.86140	3.67541	0.6248
2	13.374	BB	0.5104	9203.03125	277.15189	99.3752

Totals : 9260.89265 280.82729

===

*** End of Report ***

Data File E:\DATA\20200921\LC 2020-09-29 16-47-38\1AC-0501.D
Sample Name: YH-18-4-RAC

===

Acq. Operator : SYSTEM Seq. Line : 5
Acq. Instrument : HPLC1260 Location : P1-A3
Injection Date : 9/29/2020 6:30:56 PM Inj : 1
Inj Volume : 3.000 µl
Different Inj Volume from Sample Entry! Actual Inj Volume : 1.000 µl
Acq. Method : E:\DATA\20200921\LC 2020-09-29 16-47-38\20ETOH_15_10_4.M
Last changed : 9/29/2020 4:47:38 PM by SYSTEM
Analysis Method : E:\DATA\20200921\LC 2020-09-29 16-47-38\20ETOH_15_10_4.M (Sequence Method)
Last changed : 9/29/2020 7:25:54 PM by SYSTEM
(modified after loading)
Additional Info : Peak(s) manually integrated

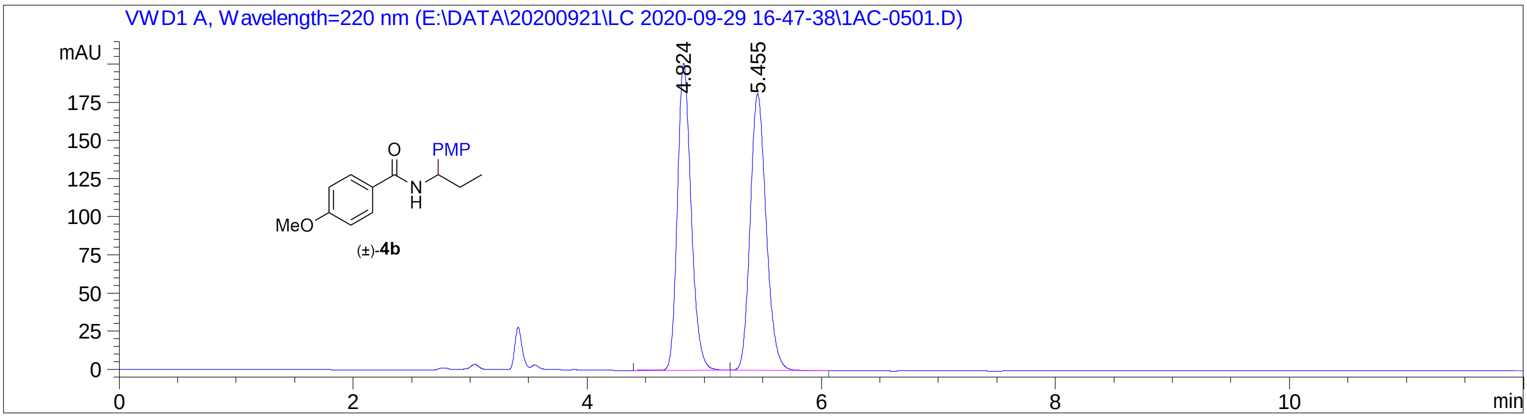

===

Area Percent Report

===

Sorted By : Signal
Multiplier : 1.0000
Dilution : 1.0000
Do not use Multiplier & Dilution Factor with ISTDs

Signal 1: VWD1 A, Wavelength=220 nm

Peak #	RetTime [min]	Type	Width [min]	Area [mAU*s]	Height [mAU]	Area %
1	4.824	VB R	0.1251	1641.40686	201.40259	48.7719
2	5.455	BB	0.1468	1724.07007	181.45563	51.2281

Totals : 3365.47693 382.85822

===

*** End of Report ***

Data File E:\DATA\20200921\LC 2020-09-29 16-47-38\1AD-0601.D
Sample Name: YH-18-4-EE

===

Acq. Operator : SYSTEM Seq. Line : 6
Acq. Instrument : HPLC1260 Location : P1-A4
Injection Date : 9/29/2020 6:46:41 PM Inj : 1
Inj Volume : 3.000 µl
Different Inj Volume from Sample Entry! Actual Inj Volume : 2.000 µl
Acq. Method : E:\DATA\20200921\LC 2020-09-29 16-47-38\20ETOH_15_10_4.M
Last changed : 9/29/2020 4:47:38 PM by SYSTEM
Analysis Method : E:\DATA\20200921\LC 2020-09-29 16-47-38\20ETOH_15_10_4.M (Sequence Method)
Last changed : 9/29/2020 7:25:10 PM by SYSTEM
(modified after loading)
Additional Info : Peak(s) manually integrated

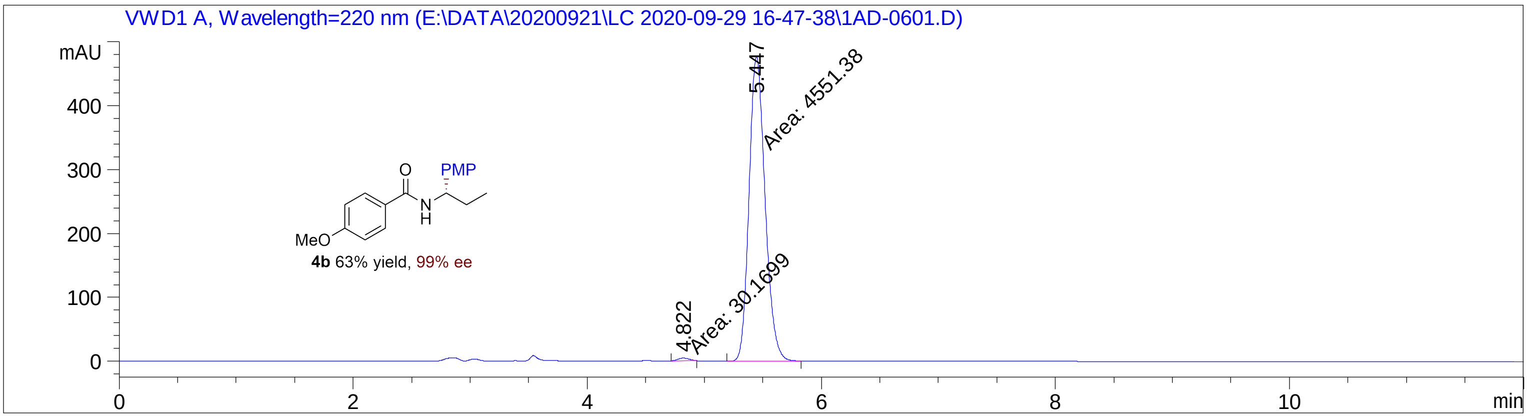

===

Area Percent Report

===

Sorted By : Signal
Multiplier : 1.0000
Dilution : 1.0000
Do not use Multiplier & Dilution Factor with ISTDs

Signal 1: VWD1 A, Wavelength=220 nm

Peak #	RetTime [min]	Type	Width [min]	Area [mAU*s]	Height [mAU]	Area %
1	4.822	MM	0.1158	30.16987	4.34245	0.6585
2	5.447	MF	0.1590	4551.37842	477.19043	99.3415

Totals : 4581.54828 481.53288

===

*** End of Report ***

Data File E:\DATA\20200921\LC 2020-09-29 16-47-38\1AE-0801.D
Sample Name: YH-18-5-RAC

===

Acq. Operator : SYSTEM Seq. Line : 8
Acq. Instrument : HPLC1260 Location : P1-A5
Injection Date : 9/29/2020 7:33:13 PM Inj : 1
Inj Volume : 3.000 µl
Different Inj Volume from Sample Entry! Actual Inj Volume : 1.000 µl
Acq. Method : E:\DATA\20200921\LC 2020-09-29 16-47-38\10ETOH_30_10_2.M
Last changed : 9/29/2020 4:47:38 PM by SYSTEM
Analysis Method : E:\DATA\20200921\LC 2020-09-29 16-47-38\10ETOH_30_10_2.M (Sequence Method)
Last changed : 9/29/2020 9:00:59 PM by SYSTEM
(modified after loading)
Additional Info : Peak(s) manually integrated

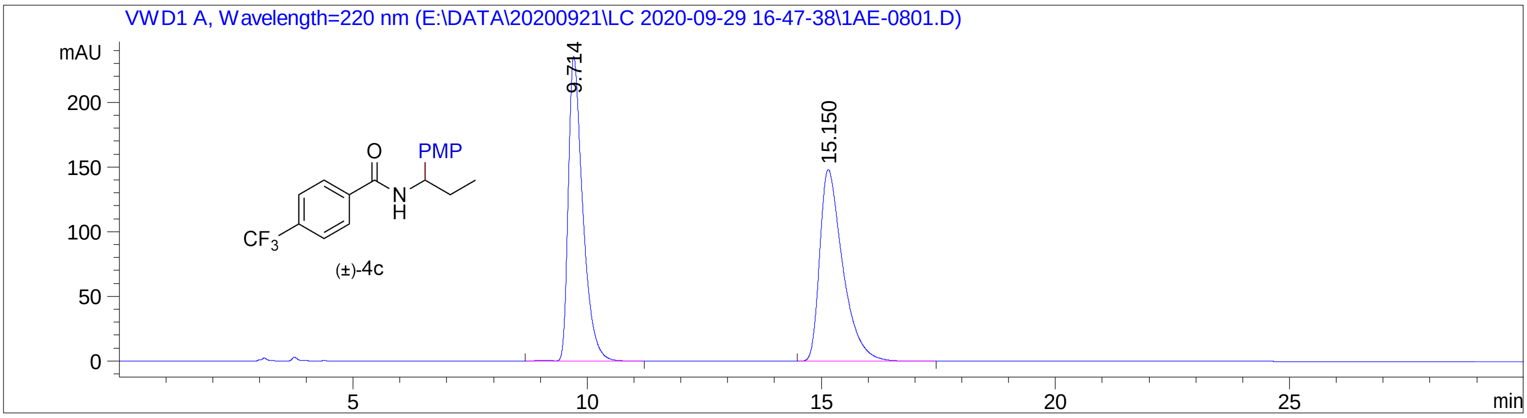

===

Area Percent Report

===

Sorted By : Signal
Multiplier : 1.0000
Dilution : 1.0000
Do not use Multiplier & Dilution Factor with ISTDs

Signal 1: VWD1 A, Wavelength=220 nm

Peak #	RetTime [min]	Type	Width [min]	Area [mAU*s]	Height [mAU]	Area %
1	9.714	VB R	0.3221	5069.50391	235.52547	49.9451
2	15.150	BB	0.5108	5080.65430	148.27206	50.0549

Totals : 1.01502e4 383.79753

===

*** End of Report ***

Data File E:\DATA\20200921\LC 2020-09-29 16-47-38\1AF-0901.D
Sample Name: YH-18-5-EE

===
Acq. Operator : SYSTEM Seq. Line : 9
Acq. Instrument : HPLC1260 Location : P1-A6
Injection Date : 9/29/2020 8:03:59 PM Inj : 1
Inj Volume : 3.000 µl
Different Inj Volume from Sample Entry! Actual Inj Volume : 2.000 µl
Acq. Method : E:\DATA\20200921\LC 2020-09-29 16-47-38\10ETOH_30_10_2.M
Last changed : 9/29/2020 4:47:38 PM by SYSTEM
Analysis Method : E:\DATA\20200921\LC 2020-09-29 16-47-38\10ETOH_30_10_2.M (Sequence Method)
Last changed : 9/29/2020 9:00:59 PM by SYSTEM
(modified after loading)
Additional Info : Peak(s) manually integrated

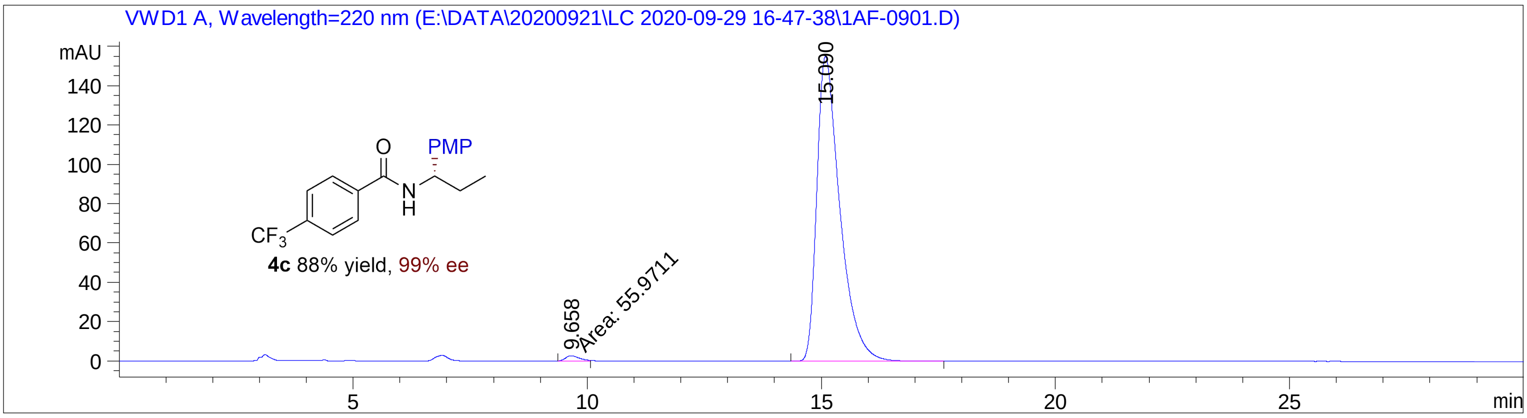

===
Area Percent Report
===

Sorted By : Signal
Multiplier : 1.0000
Dilution : 1.0000
Do not use Multiplier & Dilution Factor with ISTDs

Signal 1: VWD1 A, Wavelength=220 nm

Peak #	RetTime [min]	Type	Width [min]	Area [mAU*s]	Height [mAU]	Area %
1	9.658	FM	0.3463	55.97114	2.69383	1.0405
2	15.090	BB	0.5129	5323.53467	154.93483	98.9595

Totals : 5379.50581 157.62866

===
*** End of Report ***

Data File E:\DATA\20200921\LC 2020-09-24 18-54-40\1BA-0201.D
Sample Name: YH-17-193-RAC

===
Acq. Operator   : SYSTEM   Seq. Line :   2
Acq. Instrument : HPLC1260   Location :   P1-B1
Injection Date : 9/24/2020 7:12:41 PM   Inj :   1
                                               Inj Volume : 3.000 µl
Different Inj Volume from Sample Entry! Actual Inj Volume : 1.000 µl
Acq. Method   : E:\DATA\20200921\LC 2020-09-24 18-54-40\10IPA_15_10_2.M
Last changed   : 9/24/2020 6:54:40 PM by SYSTEM
Analysis Method : E:\DATA\20200921\LC 2020-09-24 18-54-40\10IPA_15_10_2.M (Sequence Method)
Last changed   : 9/24/2020 7:54:21 PM by SYSTEM
                  (modified after loading)
Additional Info : Peak(s) manually integrated

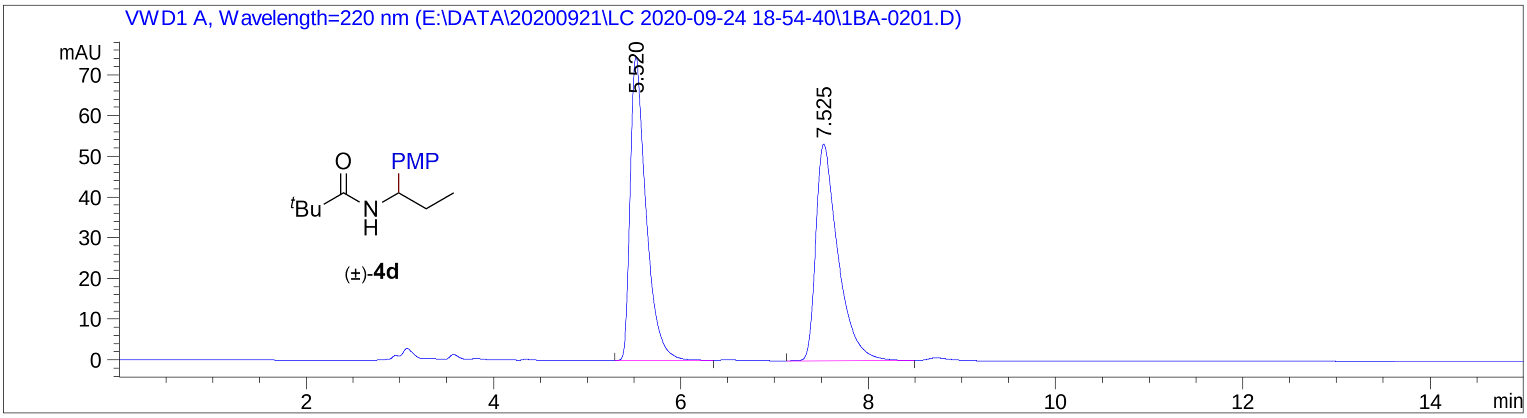

===
Area Percent Report
===

Sorted By : Signal
Multiplier : 1.0000
Dilution : 1.0000
Do not use Multiplier & Dilution Factor with ISTDs

Signal 1: VWD1 A, Wavelength=220 nm

Peak #	RetTime [min]	Type	Width [min]	Area [mAU*s]	Height [mAU]	Area %
1	5.520	BB	0.1732	877.20465	74.57279	50.0406
2	7.525	BB	0.2421	875.78021	53.18159	49.9594

Totals : 1752.98486 127.75439

===
*** End of Report ***

Data File E:\DATA\20200921\LC 2020-09-24 18-54-40\1BB-0301.D
Sample Name: YH-17-193-EE

===

Acq. Operator : SYSTEM   Seq. Line : 3
Acq. Instrument : HPLC1260   Location : P1-B2
Injection Date : 9/24/2020 7:28:25 PM   Inj : 1
Inj Volume : 3.000 µl
Different Inj Volume from Sample Entry! Actual Inj Volume : 1.000 µl
Acq. Method : E:\DATA\20200921\LC 2020-09-24 18-54-40\10IPA_15_10_2.M
Last changed : 9/24/2020 6:54:40 PM by SYSTEM
Analysis Method : E:\DATA\20200921\LC 2020-09-24 18-54-40\10IPA_15_10_2.M (Sequence Method)
Last changed : 9/24/2020 7:54:21 PM by SYSTEM
(modified after loading)
Additional Info : Peak(s) manually integrated

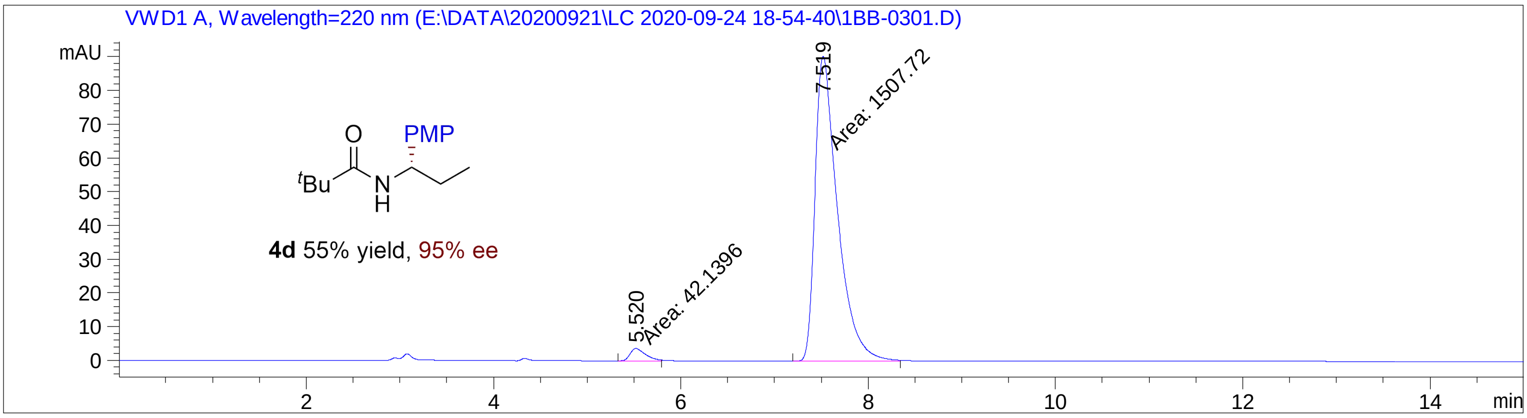

===

Area Percent Report

===

Sorted By : Signal
Multiplier : 1.0000
Dilution : 1.0000
Do not use Multiplier & Dilution Factor with ISTDs

Signal 1: VWD1 A, Wavelength=220 nm

Peak #	RetTime [min]	Type	Width [min]	Area [mAU*s]	Height [mAU]	Area %
1	5.520	MF	0.1911	42.13963	3.67431	2.7189
2	7.519	MF	0.2785	1507.71631	90.22732	97.2811

Totals : 1549.85594 93.90163

===

*** End of Report ***

Data File E:\DATA\20201001\LC 2020-10-02 21-48-39\1AA-0201.D
Sample Name: YH-18-9-RAC

===

Acq. Operator   : SYSTEM   Seq. Line :   2
Acq. Instrument : HPLC1260   Location :   P1-A1
Injection Date : 10/2/2020 10:16:45 PM   Inj :   1
                                                 Inj Volume : 3.000 µl
Different Inj Volume from Sample Entry! Actual Inj Volume : 1.000 µl
Acq. Method   : E:\DATA\20201001\LC 2020-10-02 21-48-39\10IPA_25_8_2.M
Last changed   : 10/2/2020 9:48:39 PM by SYSTEM
Analysis Method : E:\DATA\20201001\LC 2020-10-02 21-48-39\10IPA_25_8_2.M (Sequence Method)
Last changed   : 10/2/2020 11:05:53 PM by SYSTEM
                  (modified after loading)
Additional Info : Peak(s) manually integrated

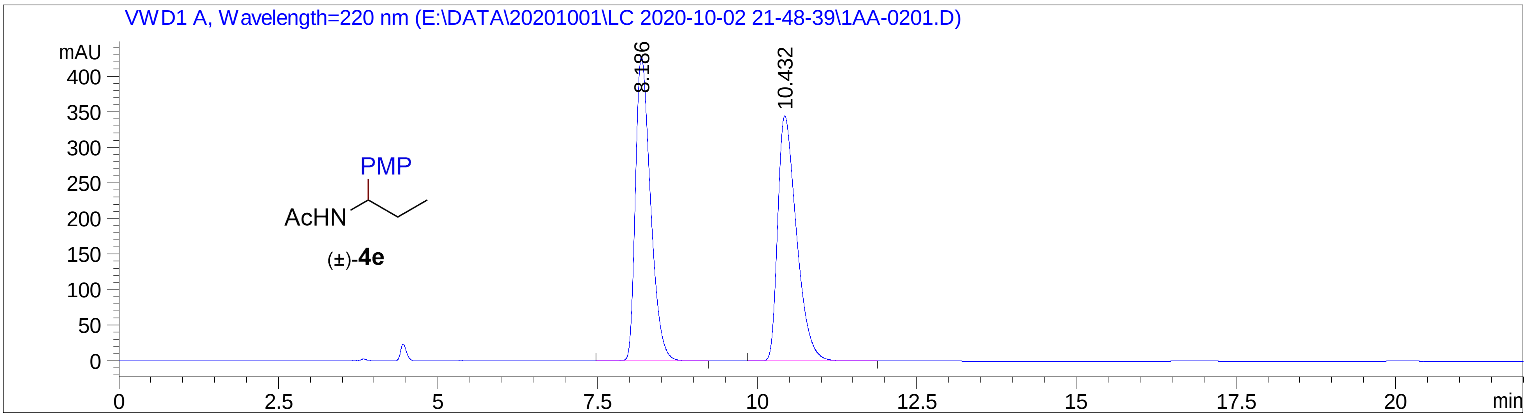

===

Area Percent Report

===

Sorted By : Signal
Multiplier : 1.0000
Dilution : 1.0000
Do not use Multiplier & Dilution Factor with ISTDs

Signal 1: VWD1 A, Wavelength=220 nm

Peak #	RetTime [min]	Type	Width [min]	Area [mAU*s]	Height [mAU]	Area %
1	8.186	BB	0.2523	7023.92188	428.44958	49.9784
2	10.432	BB	0.3119	7029.98486	344.80441	50.0216

Totals : 1.40539e4 773.25400

===

*** End of Report ***

Data File E:\DATA\20201001\LC 2020-10-02 21-48-39\1AB-0301.D
Sample Name: YH-18-9-EE

===

Acq. Operator : SYSTEM Seq. Line : 3
Acq. Instrument : HPLC1260 Location : P1-A2
Injection Date : 10/2/2020 10:42:32 PM Inj : 1
Inj Volume : 3.000 µl
Different Inj Volume from Sample Entry! Actual Inj Volume : 1.000 µl
Acq. Method : E:\DATA\20201001\LC 2020-10-02 21-48-39\10IPA_25_8_2.M
Last changed : 10/2/2020 11:04:40 PM by SYSTEM
(modified after loading)
Analysis Method : E:\DATA\20201001\LC 2020-10-02 21-48-39\10IPA_25_8_2.M (Sequence Method)
Last changed : 10/2/2020 11:06:47 PM by SYSTEM
(modified after loading)
Additional Info : Peak(s) manually integrated

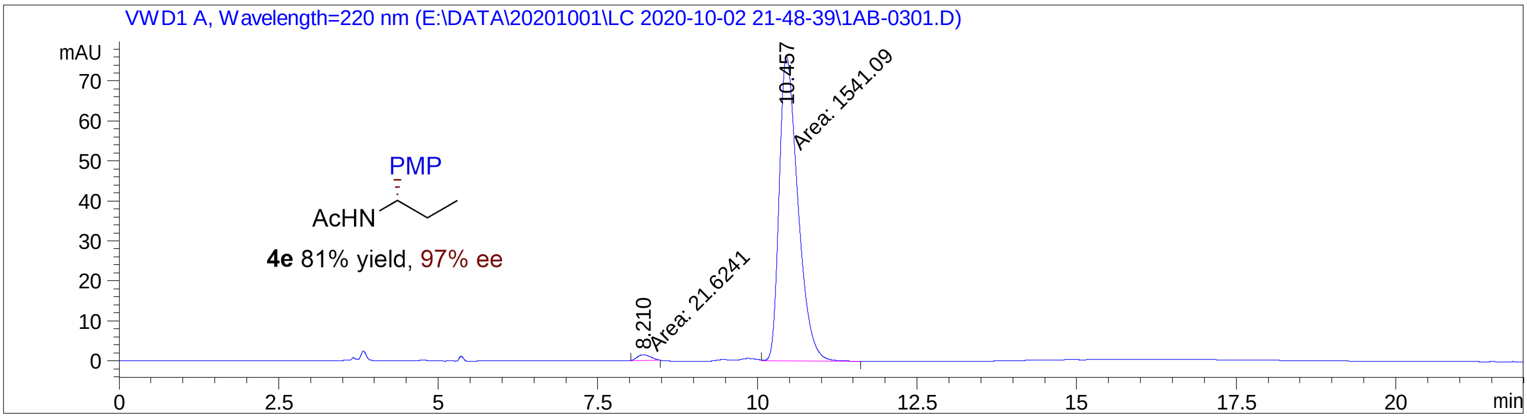

===

Area Percent Report

===

Sorted By : Signal
Multiplier : 1.0000
Dilution : 1.0000
Do not use Multiplier & Dilution Factor with ISTDs

Signal 1: VWD1 A, Wavelength=220 nm

Peak #	RetTime [min]	Type	Width [min]	Area [mAU*s]	Height [mAU]	Area %
1	8.210	MM	0.2443	21.62413	1.47499	1.3838
2	10.457	FM	0.3375	1541.08984	76.09840	98.6162

Totals : 1562.71397 77.57339

===

*** End of Report ***

Data File E:\DATA\20200921\LC 2020-09-25 17-40-35\1BA-0201.D
Sample Name: YH-17-198-RAC

===

Acq. Operator : SYSTEM Seq. Line : 2
Acq. Instrument : HPLC1260 Location : P1-B1
Injection Date : 9/25/2020 6:08:45 PM Inj : 1
Inj Volume : 3.000 µl
Different Inj Volume from Sample Entry! Actual Inj Volume : 1.000 µl
Acq. Method : E:\DATA\20200921\LC 2020-09-25 17-40-35\10ETOH_25_10_4.M
Last changed : 9/25/2020 5:40:35 PM by SYSTEM
Analysis Method : E:\DATA\20200921\LC 2020-09-25 17-40-35\10ETOH_25_10_4.M (Sequence Method)
Last changed : 9/25/2020 6:47:12 PM by SYSTEM
(modified after loading)
Additional Info : Peak(s) manually integrated

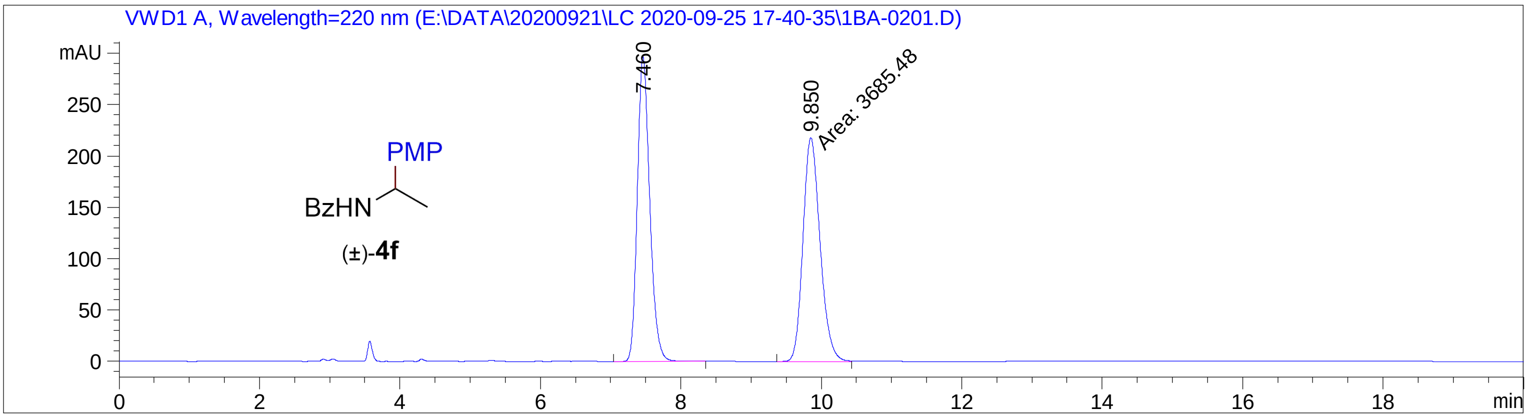

===

Area Percent Report

===

Sorted By : Signal
Multiplier : 1.0000
Dilution : 1.0000
Do not use Multiplier & Dilution Factor with ISTDs

Signal 1: VWD1 A, Wavelength=220 nm

Peak #	RetTime [min]	Type	Width [min]	Area [mAU*s]	Height [mAU]	Area %
1	7.460	BB	0.1883	3646.62793	296.84613	49.7350
2	9.850	MF	0.2820	3685.48389	217.78793	50.2650

Totals : 7332.11182 514.63406

===

*** End of Report ***

Data File E:\DATA\20200921\LC 2020-09-25 17-40-35\1BB-0301.D
Sample Name: YH-17-198-EE

===

Acq. Operator : SYSTEM Seq. Line : 3
Acq. Instrument : HPLC1260 Location : P1-B2
Injection Date : 9/25/2020 6:34:30 PM Inj : 1
Inj Volume : 3.000 µl
Different Inj Volume from Sample Entry! Actual Inj Volume : 2.000 µl
Acq. Method : E:\DATA\20200921\LC 2020-09-25 17-40-35\10ETOH_25_10_4.M
Last changed : 9/25/2020 5:40:35 PM by SYSTEM
Analysis Method : E:\DATA\20200921\LC 2020-09-25 17-40-35\10ETOH_25_10_4.M (Sequence Method)
Last changed : 9/25/2020 7:05:48 PM by SYSTEM
(modified after loading)
Additional Info : Peak(s) manually integrated

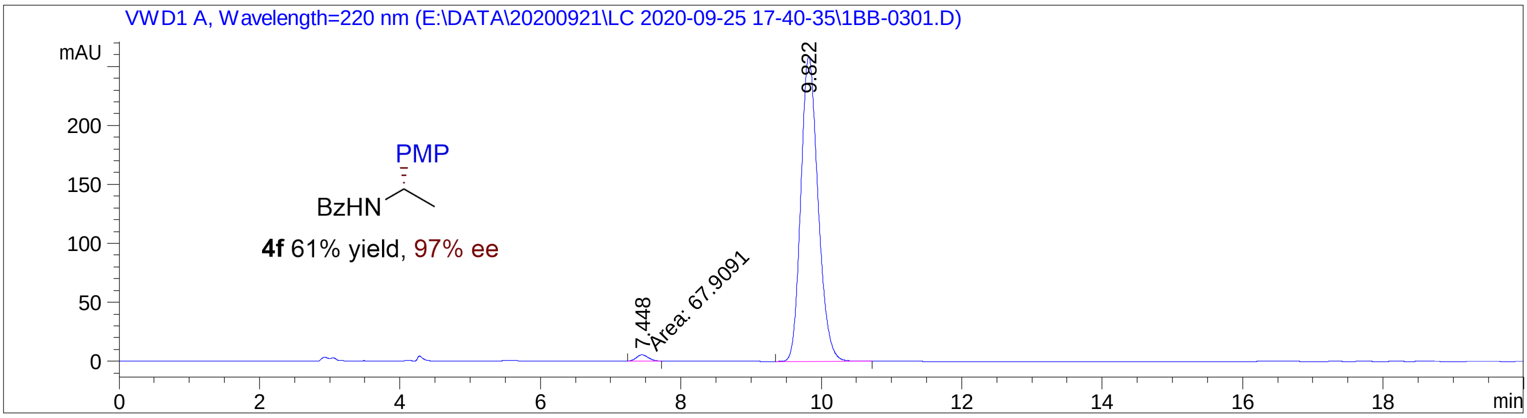

===

Area Percent Report

===

Sorted By : Signal
Multiplier : 1.0000
Dilution : 1.0000
Do not use Multiplier & Dilution Factor with ISTDs

Signal 1: VWD1 A, Wavelength=220 nm

Peak #	RetTime [min]	Type	Width [min]	Area [mAU*s]	Height [mAU]	Area %
1	7.448	MF	0.2057	67.90908	5.50341	1.5192
2	9.822	BB	0.2618	4402.19971	258.39865	98.4808

Totals : 4470.10879 263.90206

===

*** End of Report ***

Data File E:\DATA\20200921\LC 2020-09-26 15-59-13\1BA-0201.D
Sample Name: YH-17-200-RAC

===

Acq. Operator : SYSTEM Seq. Line : 2
Acq. Instrument : HPLC1260 Location : P1-B1
Injection Date : 9/26/2020 4:42:08 PM Inj : 1
Inj Volume : 3.000 µl
Different Inj Volume from Sample Entry! Actual Inj Volume : 1.000 µl
Acq. Method : E:\DATA\20200921\LC 2020-09-26 15-59-13\8EtOH_40_10_2.M
Last changed : 9/26/2020 3:59:13 PM by SYSTEM
Analysis Method : E:\DATA\20200921\LC 2020-09-26 15-59-13\8EtOH_40_10_2.M (Sequence Method)
Last changed : 9/26/2020 6:40:56 PM by SYSTEM
(modified after loading)
Additional Info : Peak(s) manually integrated

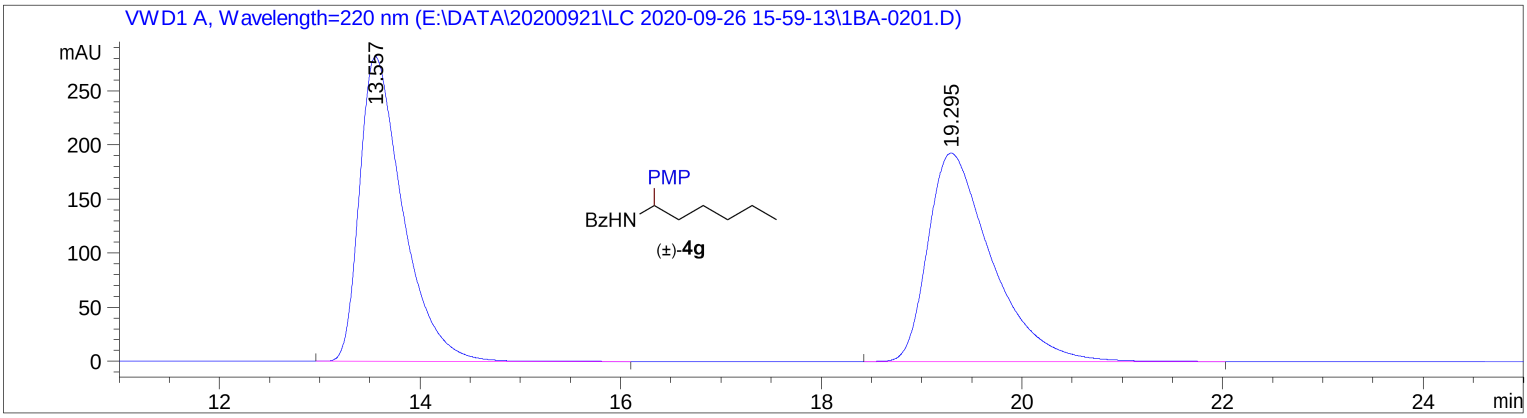

===

Area Percent Report

===

Sorted By : Signal
Multiplier : 1.0000
Dilution : 1.0000
Do not use Multiplier & Dilution Factor with ISTDs

Signal 1: VWD1 A, Wavelength=220 nm

Peak #	RetTime [min]	Type	Width [min]	Area [mAU*s]	Height [mAU]	Area %
1	13.557	BB	0.4489	8422.97559	281.62848	49.8676
2	19.295	BB	0.6560	8467.69922	192.56302	50.1324

Totals : 1.68907e4 474.19150

===

*** End of Report ***

Data File E:\DATA\20200921\LC 2020-09-26 15-59-13\1BB-0301.D
Sample Name: YH-17-200-EE

===

Acq. Operator	: SYSTEM	Seq. Line :	3
Acq. Instrument	: HPLC1260	Location :	P1-B2
Injection Date	: 9/26/2020 5:22:53 PM	Inj :	1
		Inj Volume :	3.000 µl

Different Inj Volume from Sample Entry! Actual Inj Volume : 2.000 µl
Acq. Method : E:\DATA\20200921\LC 2020-09-26 15-59-13\8EtOH_40_10_2.M
Last changed : 9/26/2020 3:59:13 PM by SYSTEM
Analysis Method : E:\DATA\20200921\LC 2020-09-26 15-59-13\8EtOH_40_10_2.M (Sequence Method)
Last changed : 9/26/2020 6:40:56 PM by SYSTEM
(modified after loading)
Additional Info : Peak(s) manually integrated

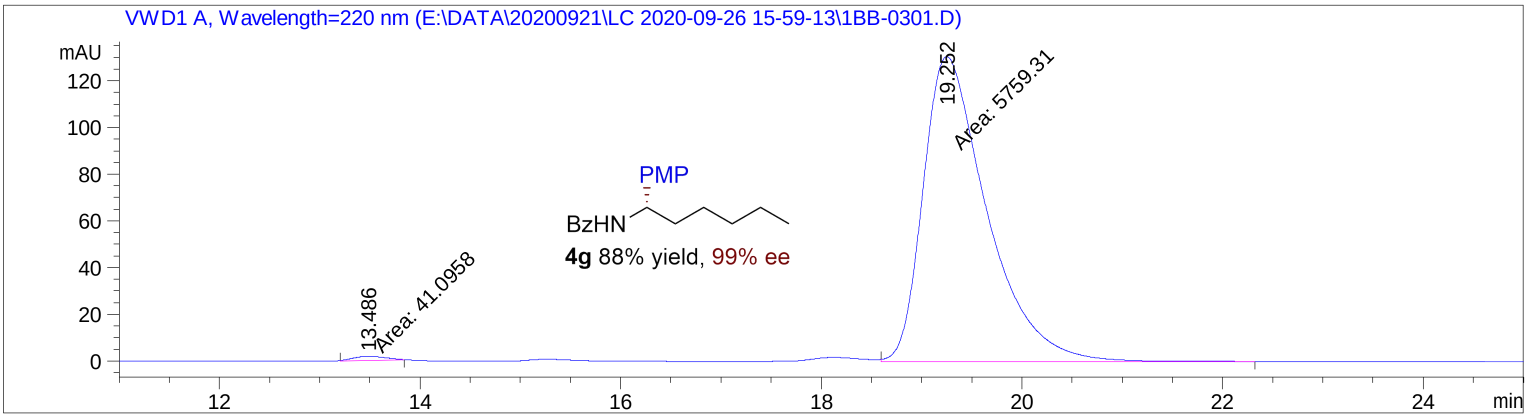

===

Area Percent Report

===

Sorted By : Signal
Multiplier : 1.0000
Dilution : 1.0000
Do not use Multiplier & Dilution Factor with ISTDs

Signal 1: VWD1 A, Wavelength=220 nm

Peak #	RetTime [min]	Type	Width [min]	Area [mAU*s]	Height [mAU]	Area %
1	13.486	MM	0.3812	41.09576	1.79668	0.7085
2	19.252	FM	0.7356	5759.31348	130.48843	99.2915

Totals : 5800.40924 132.28512

===

*** End of Report ***

Data File E:\DATA\20200927\LC 2020-09-27 10-29-43\OnlineEdited--013.D
Sample Name: YH-17-202-RAC

===

Acq. Operator : SYSTEM   Seq. Line : 13
Acq. Instrument : HPLC1260   Location : P1-B3
Injection Date : 9/27/2020 4:04:47 PM   Inj : 2
Inj Volume : 3.000 µl
Different Inj Volume from Sample Entry! Actual Inj Volume : 1.000 µl
Acq. Method : E:\DATA\20200927\LC 2020-09-27 10-29-43\20ETOH_30_10_2.M
Last changed : 9/27/2020 3:44:30 PM by SYSTEM
Analysis Method : E:\DATA\20200927\LC 2020-09-27 10-29-43\20ETOH_30_10_2.M (Sequence Method)
Last changed : 9/27/2020 7:03:19 PM by SYSTEM
(modified after loading)
Additional Info : Peak(s) manually integrated

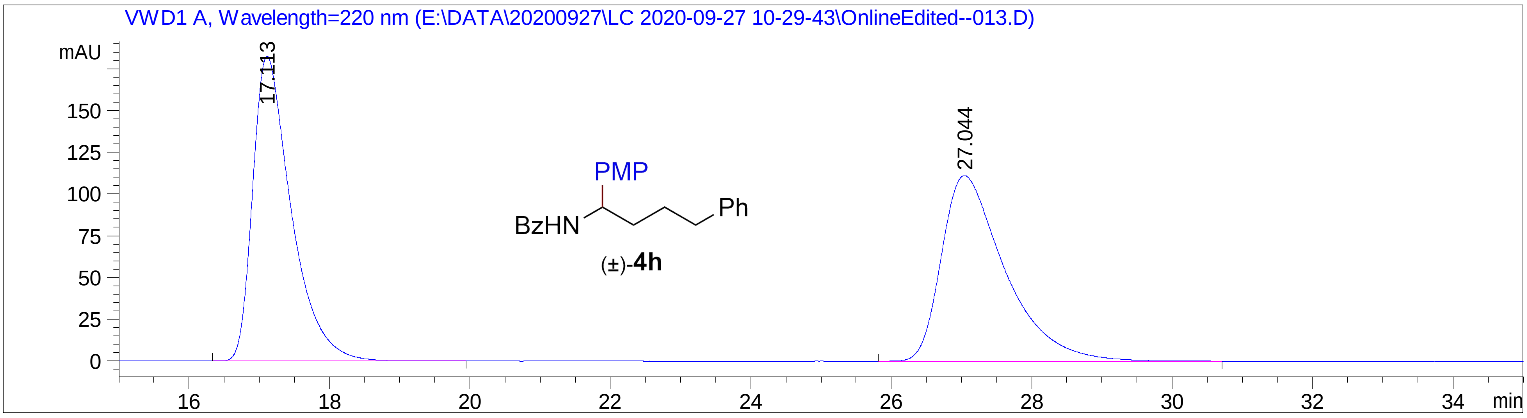

===

Area Percent Report

===

Sorted By : Signal
Multiplier : 1.0000
Dilution : 1.0000
Do not use Multiplier & Dilution Factor with ISTDs

Signal 1: VWD1 A, Wavelength=220 nm

Peak #	RetTime [min]	Type	Width [min]	Area [mAU*s]	Height [mAU]	Area %
1	17.113	BB	0.5727	6970.66895	182.48018	49.9333
2	27.044	BB	0.9367	6989.28320	111.13666	50.0667

Totals : 1.39600e4 293.61684

===

*** End of Report ***

===

Acq. Operator : SYSTEM Seq. Line : 14
Acq. Instrument : HPLC1260 Location : P1-B4
Injection Date : 9/27/2020 4:45:32 PM Inj : 1
Inj Volume : 3.000 µl
Different Inj Volume from Sample Entry! Actual Inj Volume : 2.000 µl
Acq. Method : E:\DATA\20200927\LC 2020-09-27 10-29-43\20ETOH_30_10_2.M
Last changed : 9/27/2020 3:44:30 PM by SYSTEM
Analysis Method : E:\DATA\20200927\LC 2020-09-27 10-29-43\20ETOH_30_10_2.M (Sequence Method)
Last changed : 9/27/2020 7:03:19 PM by SYSTEM
(modified after loading)
Additional Info : Peak(s) manually integrated

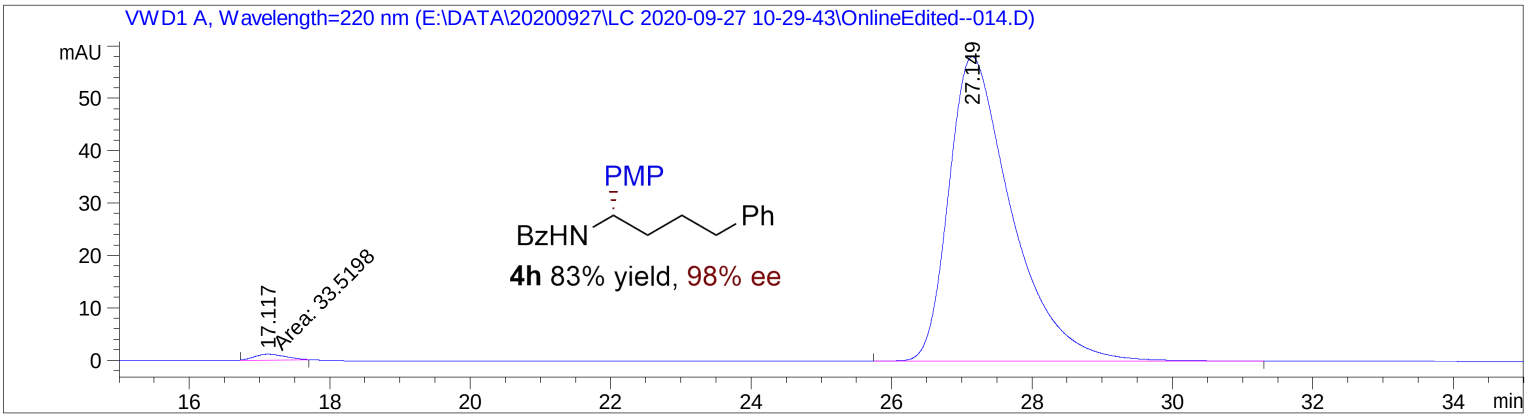

===

Area Percent Report

===

Sorted By : Signal
Multiplier : 1.0000
Dilution : 1.0000
Do not use Multiplier & Dilution Factor with ISTDs

Signal 1: VWD1 A, Wavelength=220 nm

Peak #	RetTime [min]	Type	Width [min]	Area [mAU*s]	Height [mAU]	Area %
1	17.117	MM	0.5076	33.51980	1.10059	0.9187
2	27.149	BB	0.9217	3614.92407	58.11379	99.0813

Totals : 3648.44387 59.21438

===

*** End of Report ***

Data File E:\DATA\20200921\LC 2020-09-24 18-54-40\OnlineEdited--007.D
Sample Name: YH-17-193-RAC

```
=====================================================================
Acq. Operator   : SYSTEM                          Seq. Line :    7
Acq. Instrument : HPLC1260                         Location :   P1-B1
Injection Date  : 9/24/2020 9:01:50 PM                  Inj :    1
                                                 Inj Volume : 3.000 µl
Different Inj Volume from Sample Entry!  Actual Inj Volume : 1.000 µl
Acq. Method     : E:\DATA\20200921\LC 2020-09-24 18-54-40\10IPA_30_10_2.M
Last changed    : 9/24/2020 8:44:09 PM by SYSTEM
Analysis Method : E:\DATA\20200921\LC 2020-09-24 18-54-40\10IPA_30_10_2.M (Sequence Method)
Last changed    : 9/24/2020 10:01:25 PM by SYSTEM
                  (modified after loading)
Additional Info : Peak(s) manually integrated
```

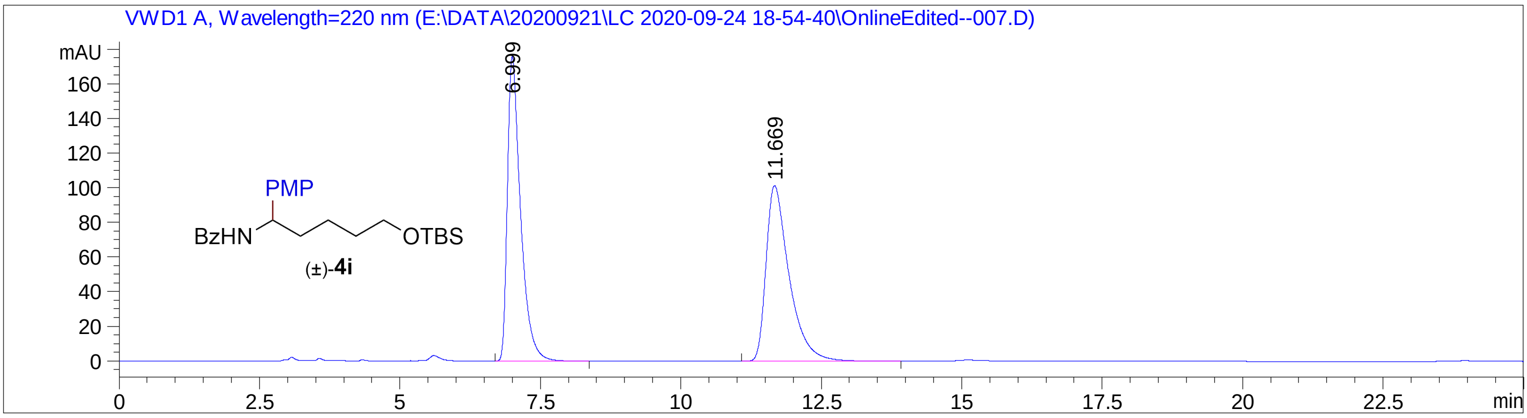

===
Area Percent Report
===

Sorted By : Signal
Multiplier : 1.0000
Dilution : 1.0000
Do not use Multiplier & Dilution Factor with ISTDs

Signal 1: VWD1 A, Wavelength=220 nm

Peak #	RetTime [min]	Type	Width [min]	Area [mAU*s]	Height [mAU]	Area %
1	6.999	BB	0.2411	2864.67676	175.83029	49.7185
2	11.669	BB	0.4222	2897.10986	101.43122	50.2815

Totals : 5761.78662 277.26151

===
*** End of Report ***

Data File E:\DATA\20200921\LC 2020-09-24 18-54-40\OnlineEdited--008.D
Sample Name: YH-17-193-EE

===

Acq. Operator : SYSTEM Seq. Line : 8
Acq. Instrument : HPLC1260 Location : P1-B2
Injection Date : 9/24/2020 9:32:35 PM Inj : 1
Inj Volume : 3.000 µl
Different Inj Volume from Sample Entry! Actual Inj Volume : 1.000 µl
Acq. Method : E:\DATA\20200921\LC 2020-09-24 18-54-40\10IPA_30_10_2.M
Last changed : 9/24/2020 10:01:49 PM by SYSTEM
(modified after loading)
Analysis Method : E:\DATA\20200921\LC 2020-09-24 18-54-40\10IPA_30_10_2.M (Sequence Method)
Last changed : 9/24/2020 10:03:41 PM by SYSTEM
(modified after loading)
Additional Info : Peak(s) manually integrated

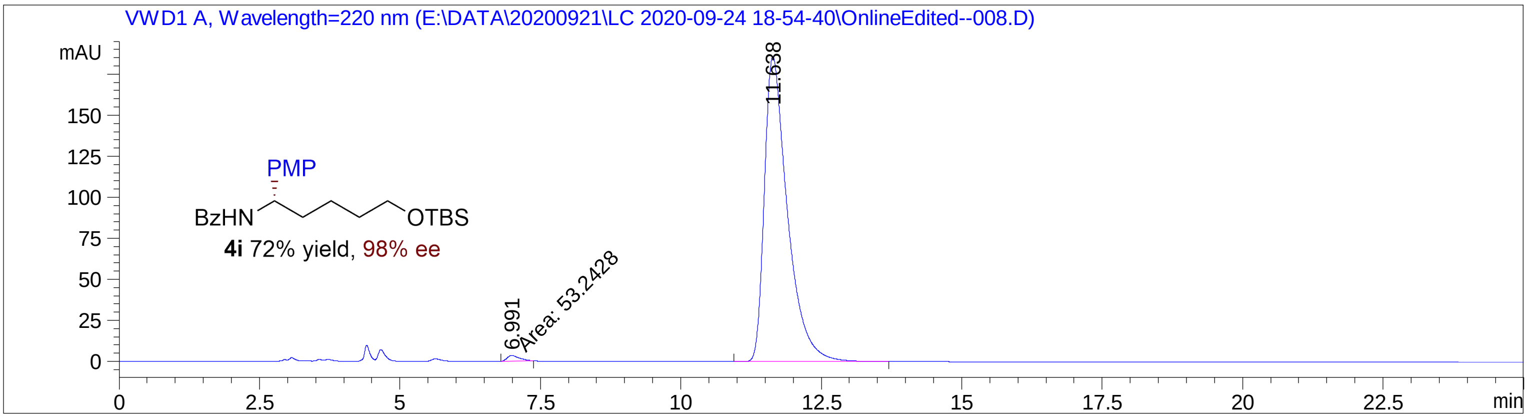

===

Area Percent Report

===

Sorted By : Signal
Multiplier : 1.0000
Dilution : 1.0000
Do not use Multiplier & Dilution Factor with ISTDs

Signal 1: VWD1 A, Wavelength=220 nm

Peak #	RetTime [min]	Type	Width [min]	Area [mAU*s]	Height [mAU]	Area %
1	6.991	MM	0.2540	53.24284	3.49425	0.9907
2	11.638	BB	0.4250	5321.20215	185.89029	99.0093

Totals : 5374.44498 189.38454

===

*** End of Report ***

Data File E:\DATA\20200927\LC 2020-09-27 10-29-43\OnlineEdited--009.D
Sample Name: YH-17-201-RAC

===

Acq. Operator : SYSTEM   Seq. Line : 9
Acq. Instrument : HPLC1260   Location : P1-B1
Injection Date : 9/27/2020 1:51:47 PM   Inj : 1
Inj Volume : 3.000 µl
Different Inj Volume from Sample Entry! Actual Inj Volume : 1.000 µl
Acq. Method : E:\DATA\20200927\LC 2020-09-27 10-29-43\20ETOH_30_10_2.M
Last changed : 9/27/2020 1:18:50 PM by SYSTEM
Analysis Method : E:\DATA\20200927\LC 2020-09-27 10-29-43\20ETOH_30_10_2.M (Sequence Method)
Last changed : 9/27/2020 3:24:43 PM by SYSTEM
(modified after loading)
Additional Info : Peak(s) manually integrated

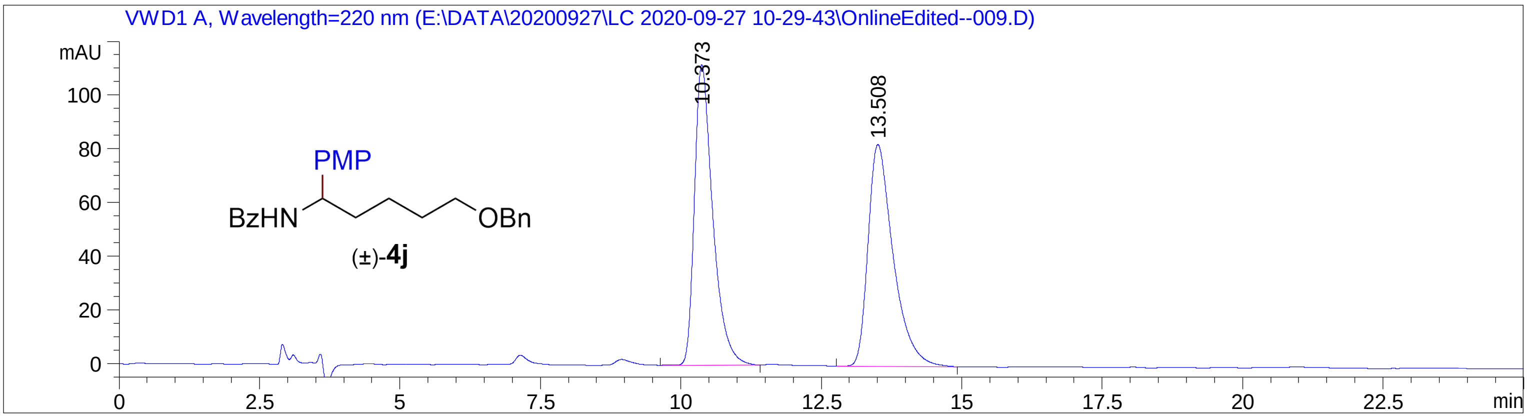

===

Area Percent Report

===

Sorted By : Signal
Multiplier : 1.0000
Dilution : 1.0000
Do not use Multiplier & Dilution Factor with ISTDs

Signal 1: VWD1 A, Wavelength=220 nm

Peak #	RetTime [min]	Type	Width [min]	Area [mAU*s]	Height [mAU]	Area %
1	10.373	BB	0.3411	2546.71680	112.07801	49.7002
2	13.508	BB	0.4655	2577.43799	82.71635	50.2998

Totals : 5124.15479 194.79436

===

*** End of Report ***

Data File E:\DATA\20200927\LC 2020-09-27 10-29-43\OnlineEdited--010.D
Sample Name: YH-17-201-EE

===

Acq. Operator : SYSTEM   Seq. Line : 10
Acq. Instrument : HPLC1260   Location : P1-B2
Injection Date : 9/27/2020 2:22:33 PM   Inj : 1
Inj Volume : 3.000 µl
Different Inj Volume from Sample Entry! Actual Inj Volume : 2.000 µl
Acq. Method : E:\DATA\20200927\LC 2020-09-27 10-29-43\20ETOH_30_10_2.M
Last changed : 9/27/2020 1:18:50 PM by SYSTEM
Analysis Method : E:\DATA\20200927\LC 2020-09-27 10-29-43\20ETOH_30_10_2.M (Sequence Method)
Last changed : 9/27/2020 3:24:43 PM by SYSTEM
(modified after loading)
Additional Info : Peak(s) manually integrated

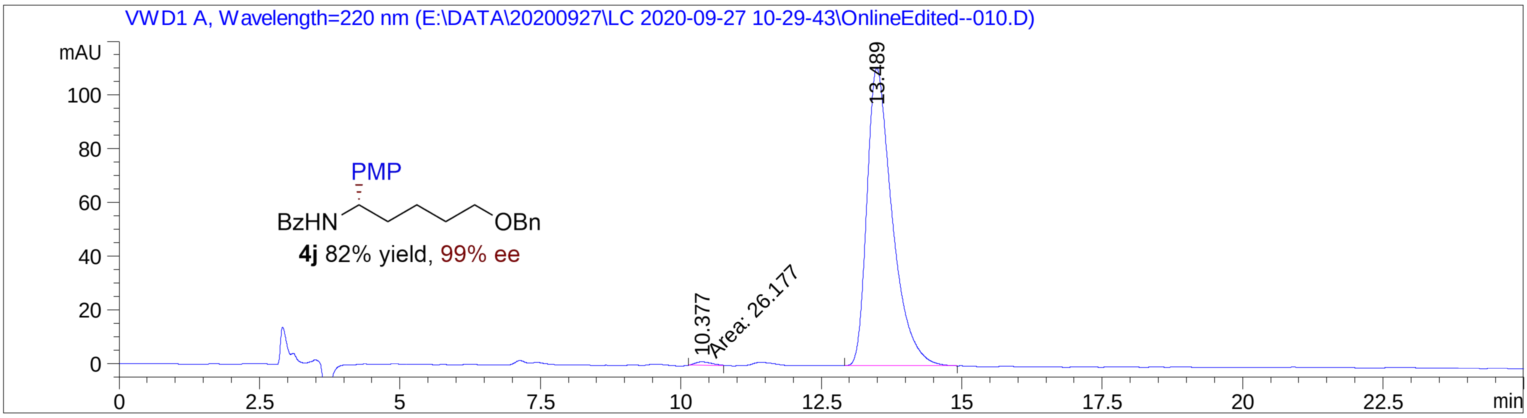

===

Area Percent Report

===

Sorted By : Signal
Multiplier : 1.0000
Dilution : 1.0000
Do not use Multiplier & Dilution Factor with ISTDs

Signal 1: VWD1 A, Wavelength=220 nm

Peak #	RetTime [min]	Type	Width [min]	Area [mAU*s]	Height [mAU]	Area %
1	10.377	MM	0.3386	26.17703	1.28860	0.7478
2	13.489	BB	0.4697	3474.51636	110.84788	99.2522

Totals : 3500.69339 112.13647

===

*** End of Report ***

Data File E:\DATA\20200927\LC 2020-09-27 10-29-43\OnlineEdited--036.D
Sample Name: YH-17-203-RAC

===

Acq. Operator : SYSTEM Seq. Line : 36
Acq. Instrument : HPLC1260 Location : P1-B1
Injection Date : 9/28/2020 2:33:12 AM Inj : 1
Inj Volume : 3.000 µl
Different Inj Volume from Sample Entry! Actual Inj Volume : 1.000 µl
Acq. Method : E:\DATA\20200927\LC 2020-09-27 10-29-43\20ETOH_30_10_4.M
Last changed : 9/27/2020 7:51:03 PM by SYSTEM
Analysis Method : E:\DATA\20200927\LC 2020-09-27 10-29-43\20ETOH_30_10_4.M (Sequence Method)
Last changed : 9/28/2020 9:20:43 AM by SYSTEM
(modified after loading)
Additional Info : Peak(s) manually integrated

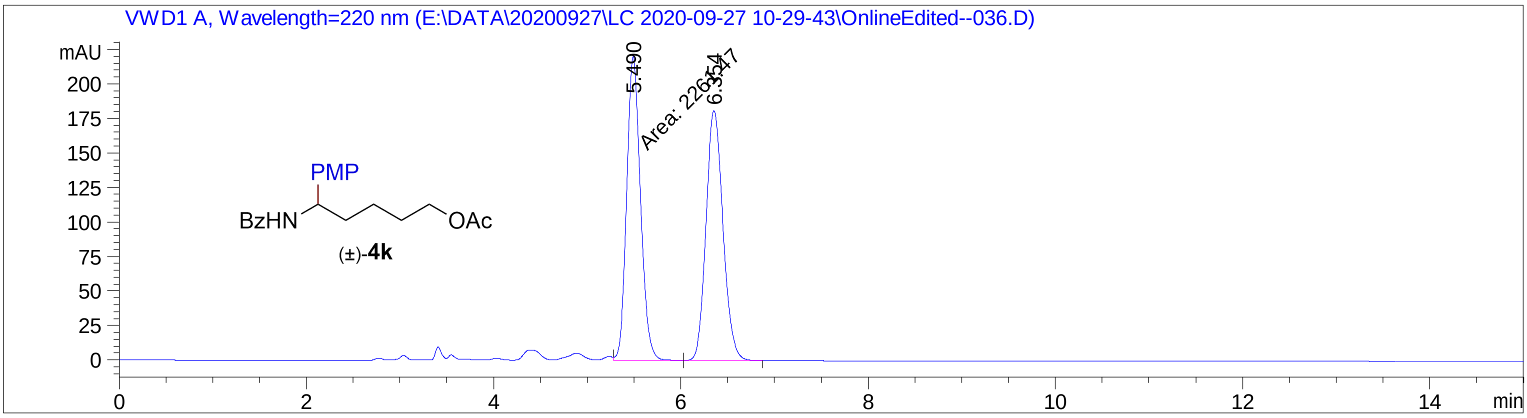

===

Area Percent Report

===

Sorted By : Signal
Multiplier : 1.0000
Dilution : 1.0000
Do not use Multiplier & Dilution Factor with ISTDs

Signal 1: VWD1 A, Wavelength=220 nm

Peak #	RetTime [min]	Type	Width [min]	Area [mAU*s]	Height [mAU]	Area %
1	5.490	FM	0.1713	2261.47437	220.06464	49.7877
2	6.354	BB	0.1950	2280.76050	181.00043	50.2123

Totals : 4542.23486 401.06506

===

*** End of Report ***

===

Acq. Operator : SYSTEM Seq. Line : 37
Acq. Instrument : HPLC1260 Location : P1-B2
Injection Date : 9/28/2020 3:03:58 AM Inj : 1
Inj Volume : 3.000 µl
Different Inj Volume from Sample Entry! Actual Inj Volume : 2.000 µl
Acq. Method : E:\DATA\20200927\LC 2020-09-27 10-29-43\20ETOH_30_10_4.M
Last changed : 9/27/2020 7:51:03 PM by SYSTEM
Analysis Method : E:\DATA\20200927\LC 2020-09-27 10-29-43\20ETOH_30_10_4.M (Sequence Method)
Last changed : 9/28/2020 9:23:00 AM by SYSTEM
(modified after loading)
Additional Info : Peak(s) manually integrated

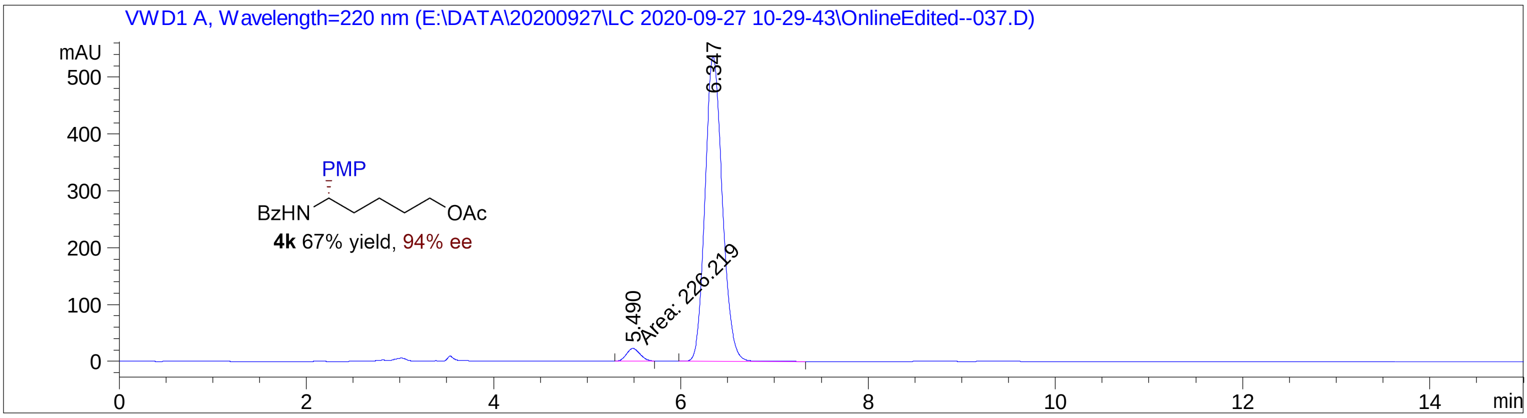

===

Area Percent Report

===

Sorted By : Signal
Multiplier : 1.0000
Dilution : 1.0000
Do not use Multiplier & Dilution Factor with ISTDs

Signal 1: VWD1 A, Wavelength=220 nm

Peak #	RetTime [min]	Type	Width [min]	Area [mAU*s]	Height [mAU]	Area %
1	5.490	MM	0.1675	226.21860	22.51526	3.1813
2	6.347	BB	0.1987	6884.65332	536.38910	96.8187

Totals : 7110.87192 558.90436

===

*** End of Report ***

Data File E:\DATA\20200921\LC 2020-09-25 17-40-35\OnlineEdited--012.D
Sample Name: YH-17-196-RAC

===

Acq. Operator : SYSTEM Seq. Line : 12
Acq. Instrument : HPLC1260 Location : P1-B1
Injection Date : 9/25/2020 10:27:33 PM Inj : 1
Inj Volume : 3.000 µl
Different Inj Volume from Sample Entry! Actual Inj Volume : 1.000 µl
Acq. Method : E:\DATA\20200921\LC 2020-09-25 17-40-35\10ETOH_25_10_4.M
Last changed : 9/25/2020 9:47:14 PM by SYSTEM
Analysis Method : E:\DATA\20200921\LC 2020-09-25 17-40-35\10ETOH_25_10_4.M (Sequence Method)
Last changed : 9/26/2020 8:45:27 AM by SYSTEM
(modified after loading)
Additional Info : Peak(s) manually integrated

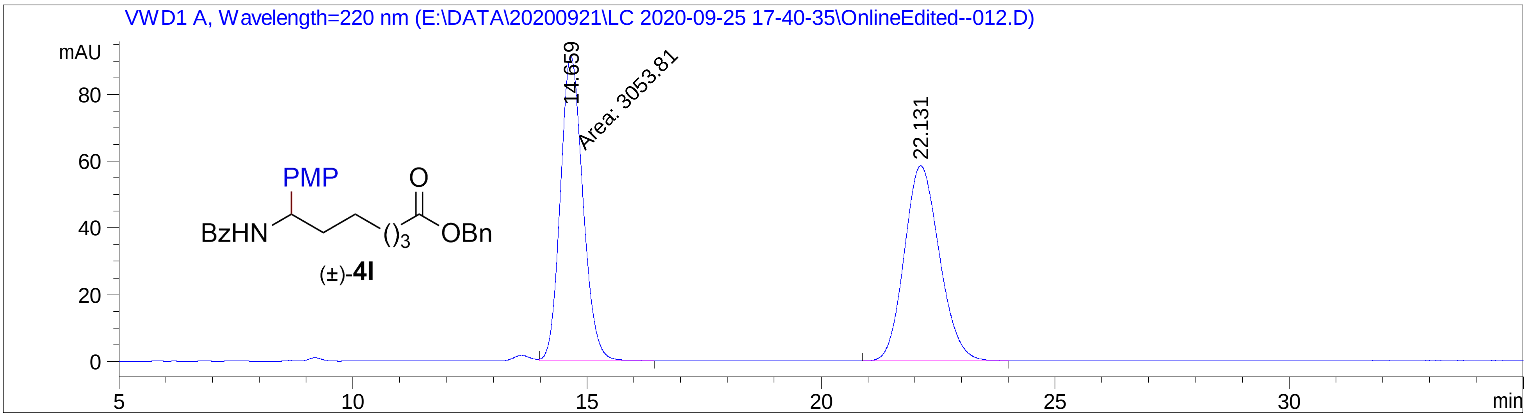

===

Area Percent Report

===

Sorted By : Signal
Multiplier : 1.0000
Dilution : 1.0000
Do not use Multiplier & Dilution Factor with ISTDs

Signal 1: VWD1 A, Wavelength=220 nm

Peak #	RetTime [min]	Type	Width [min]	Area [mAU*s]	Height [mAU]	Area %
1	14.659	FM	0.5577	3053.80542	91.25372	50.0352
2	22.131	BB	0.8105	3049.50684	58.46655	49.9648

Totals : 6103.31226 149.72026

===

*** End of Report ***

===

Acq. Operator : SYSTEM Seq. Line : 13
Acq. Instrument : HPLC1260 Location : P1-B2
Injection Date : 9/25/2020 11:08:19 PM Inj : 1
Inj Volume : 3.000 µl
Different Inj Volume from Sample Entry! Actual Inj Volume : 2.000 µl
Acq. Method : E:\DATA\20200921\LC 2020-09-25 17-40-35\10ETOH_25_10_4.M
Last changed : 9/25/2020 9:47:14 PM by SYSTEM
Analysis Method : E:\DATA\20200921\LC 2020-09-25 17-40-35\10ETOH_25_10_4.M (Sequence Method)
Last changed : 9/26/2020 8:46:05 AM by SYSTEM
(modified after loading)
Additional Info : Peak(s) manually integrated

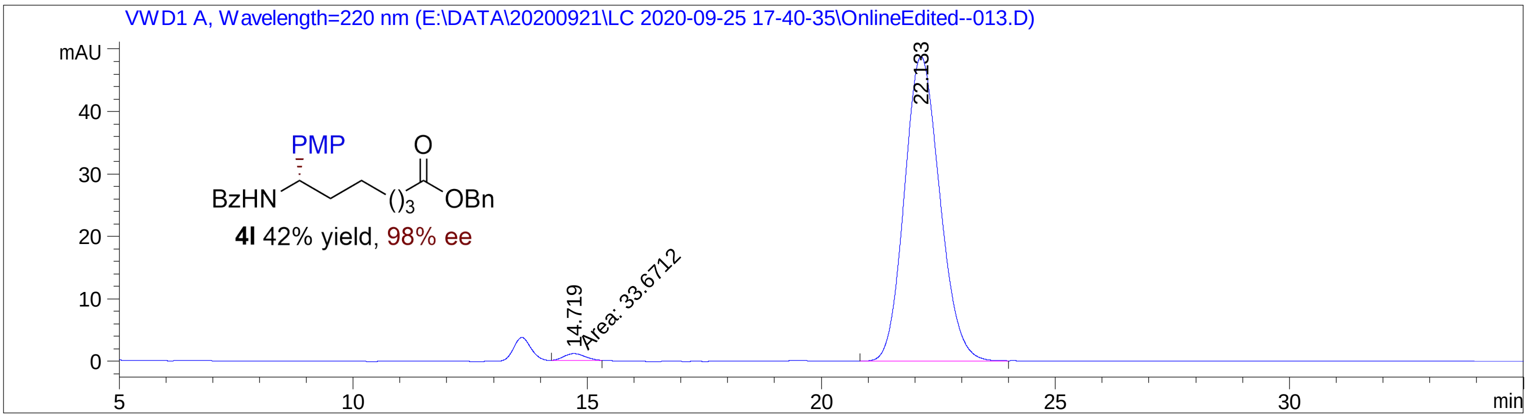

===

Area Percent Report

===

Sorted By : Signal
Multiplier : 1.0000
Dilution : 1.0000
Do not use Multiplier & Dilution Factor with ISTDs

Signal 1: VWD1 A, Wavelength=220 nm

Peak #	RetTime [min]	Type	Width [min]	Area [mAU*s]	Height [mAU]	Area %
1	14.719	MM	0.5185	33.67116	1.08240	1.2957
2	22.133	BB	0.8149	2565.07397	48.74711	98.7043

Totals : 2598.74513 49.82951

===

*** End of Report ***

Data File E:\DATA\20200921\LC 2020-09-26 14-39-28\1BA-0201.D
Sample Name: YH-17-199-RAC

===

Acq. Operator : SYSTEM Seq. Line : 2
Acq. Instrument : HPLC1260 Location : P1-B1
Injection Date : 9/26/2020 3:07:01 PM Inj : 1
Inj Volume : 3.000 µl
Different Inj Volume from Sample Entry! Actual Inj Volume : 1.000 µl
Acq. Method : E:\DATA\20200921\LC 2020-09-26 14-39-28\20ETOH_30_10_2.M
Last changed : 9/26/2020 2:51:24 PM by SYSTEM
Analysis Method : E:\DATA\20200921\LC 2020-09-26 14-39-28\20ETOH_30_10_2.M (Sequence Method)
Last changed : 9/26/2020 3:55:30 PM by SYSTEM
(modified after loading)
Additional Info : Peak(s) manually integrated

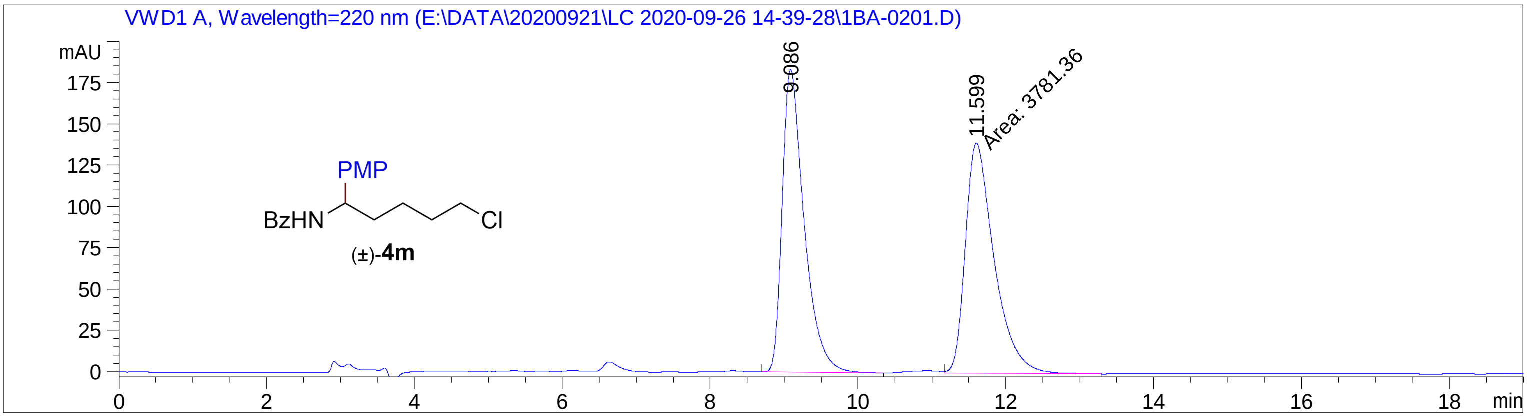

===

Area Percent Report

===

Sorted By : Signal
Multiplier : 1.0000
Dilution : 1.0000
Do not use Multiplier & Dilution Factor with ISTDs

Signal 1: VWD1 A, Wavelength=220 nm

Peak #	RetTime [min]	Type	Width [min]	Area [mAU*s]	Height [mAU]	Area %
1	9.086	BB	0.3065	3774.72949	183.12154	49.9561
2	11.599	FM	0.4523	3781.35938	139.32381	50.0439

Totals : 7556.08887 322.44534

===

*** End of Report ***

Data File E:\DATA\20200921\LC 2020-09-26 14-39-28\1BB-0301.D
Sample Name: YH-17-199-EE

===

Acq. Operator : SYSTEM   Seq. Line : 3
Location : P1-B2
Injection Date : 9/26/2020 3:32:45 PM   Inj : 1
Acq. Method : 20ETOH_30_10_2.M
Analysis Method : E:\DATA\20200921\LC 2020-09-26 14-39-28\20ETOH_30_10_2.M (Sequence Method)
Last changed : 9/26/2020 3:55:50 PM by SYSTEM
(modified after loading)
Additional Info : Peak(s) manually integrated

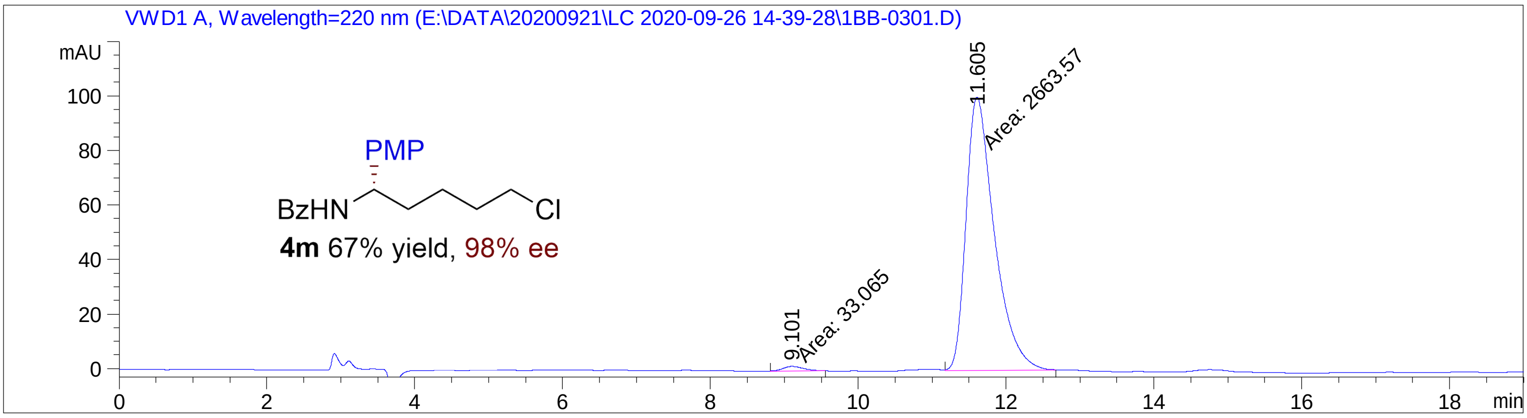

===

Area Percent Report

===

Sorted By : Signal
Multiplier : 1.0000
Dilution : 1.0000
Do not use Multiplier & Dilution Factor with ISTDs

Signal 1: VWD1 A, Wavelength=220 nm

Peak #	RetTime [min]	Type	Width [min]	Area [mAU*s]	Height [mAU]	Area %
1	9.101	MM	0.3246	33.06498	1.69750	1.2262
2	11.605	FM	0.4430	2663.57129	100.22038	98.7738

Totals : 2696.63626 101.91788

===

*** End of Report ***

Data File E:\DATA\20201009\LC 2020-10-10 15-52-29\1AA-0201.D
Sample Name: YH-18-52-RAC

===

Acq. Operator : SYSTEM Seq. Line : 2
Acq. Instrument : HPLC1260 Location : P1-A1
Injection Date : 10/10/2020 4:10:52 PM Inj : 1
Inj Volume : 3.000 µl
Different Inj Volume from Sample Entry! Actual Inj Volume : 1.000 µl
Acq. Method : E:\DATA\20201009\LC 2020-10-10 15-52-29\10IPA_15_10_2.M
Last changed : 10/10/2020 4:18:25 PM by SYSTEM
(modified after loading)
Analysis Method : E:\DATA\20201009\LC 2020-10-10 15-52-29\10IPA_15_10_2.M (Sequence Method)
Last changed : 10/10/2020 9:54:10 PM by SYSTEM
(modified after loading)
Additional Info : Peak(s) manually integrated

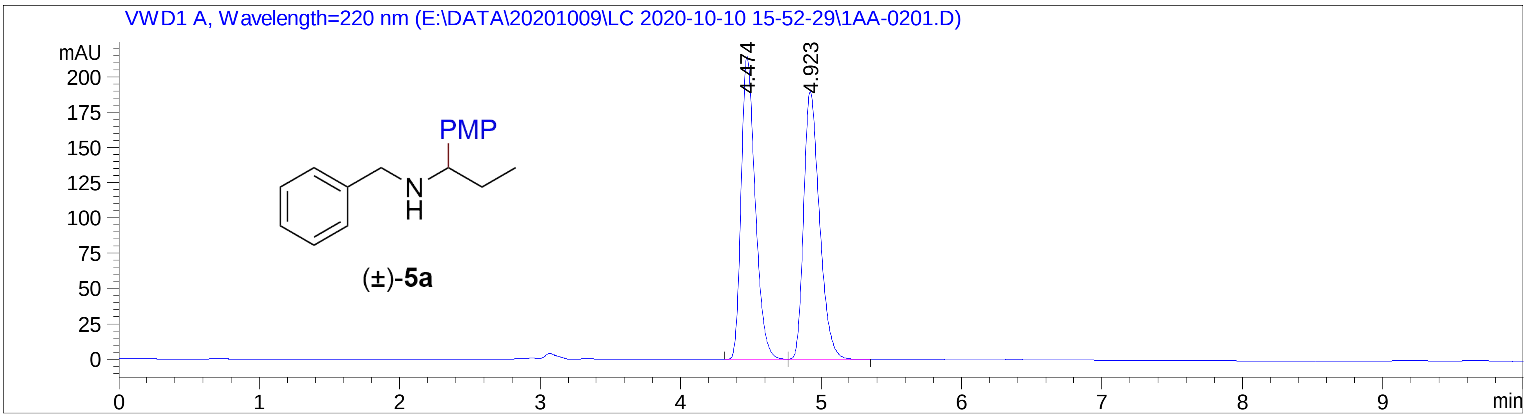

===

Area Percent Report

===

Sorted By : Signal
Multiplier : 1.0000
Dilution : 1.0000
Do not use Multiplier & Dilution Factor with ISTDs

Signal 1: VWD1 A, Wavelength=220 nm

Peak #	RetTime [min]	Type	Width [min]	Area [mAU*s]	Height [mAU]	Area %
1	4.474	BB	0.1073	1493.57813	214.37967	50.1009
2	4.923	BB	0.1195	1487.56238	189.71567	49.8991

Totals : 2981.14050 404.09534

===

*** End of Report ***

Data File E:\DATA\20201009\LC 2020-10-10 20-21-48\OnlineEdited--004.D
Sample Name: YH-18-52-EE

===

Acq. Operator	: SYSTEM	Seq. Line :	4
Acq. Instrument	: HPLC1260	Location :	P1-B2
Injection Date	: 10/10/2020 9:33:42 PM	Inj :	1
		Inj Volume :	3.000 µl

Acq. Method : E:\DATA\20201009\LC 2020-10-10 20-21-48\10IPA_10_10_2.M
Last changed : 10/10/2020 9:18:38 PM by SYSTEM
Analysis Method : E:\DATA\20201009\LC 2020-10-10 20-21-48\10IPA_10_10_2.M (Sequence Method)
Last changed : 10/10/2020 9:53:34 PM by SYSTEM
(modified after loading)
Additional Info : Peak(s) manually integrated

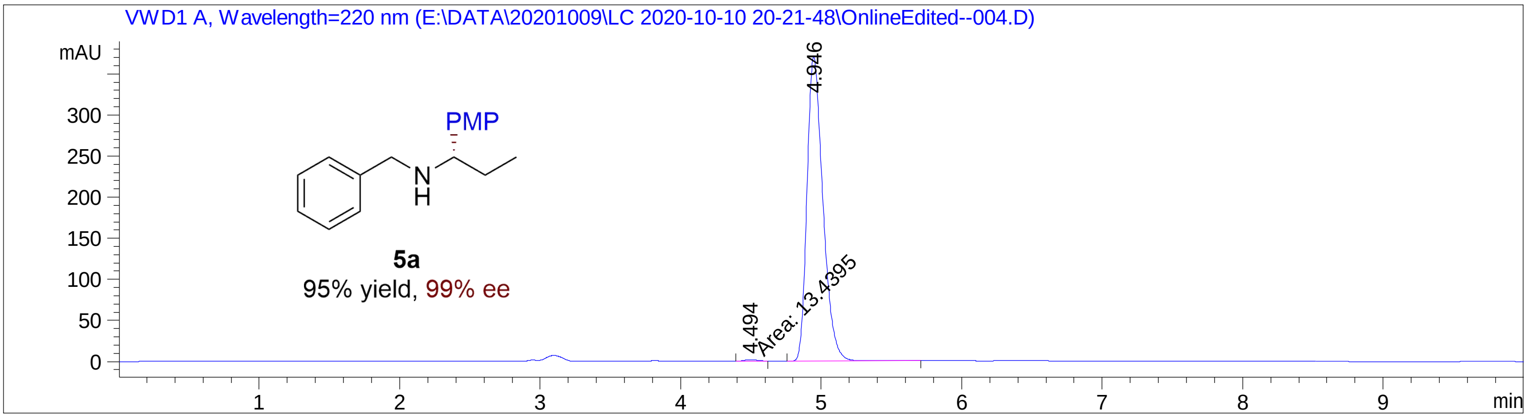

===

Area Percent Report

===

Sorted By : Signal
Multiplier : 1.0000
Dilution : 1.0000
Do not use Multiplier & Dilution Factor with ISTDs

Signal 1: VWD1 A, Wavelength=220 nm

Peak #	RetTime [min]	Type	Width [min]	Area [mAU*s]	Height [mAU]	Area %
1	4.494	FM	0.1125	13.43952	1.99146	0.4577
2	4.946	BB	0.1199	2923.08179	370.98889	99.5423

Totals : 2936.52131 372.98035

===

*** End of Report ***

Data File E:\DATA\20201009\LC 2020-10-10 16-56-12\1AA-0201.D
Sample Name: YH-18-55-RAC

===

Acq. Operator : SYSTEM Seq. Line : 2
Acq. Instrument : HPLC1260 Location : P1-A1
Injection Date : 10/10/2020 5:29:21 PM Inj : 1
Inj Volume : 3.000 µl
Different Inj Volume from Sample Entry! Actual Inj Volume : 1.000 µl
Acq. Method : E:\DATA\20201009\LC 2020-10-10 16-56-12\20IPA_30_10_2.M
Last changed : 10/10/2020 5:48:04 PM by SYSTEM
(modified after loading)
Analysis Method : E:\DATA\20201009\LC 2020-10-10 16-56-12\20IPA_30_10_2.M (Sequence Method)
Last changed : 10/10/2020 6:18:53 PM by SYSTEM
(modified after loading)
Additional Info : Peak(s) manually integrated

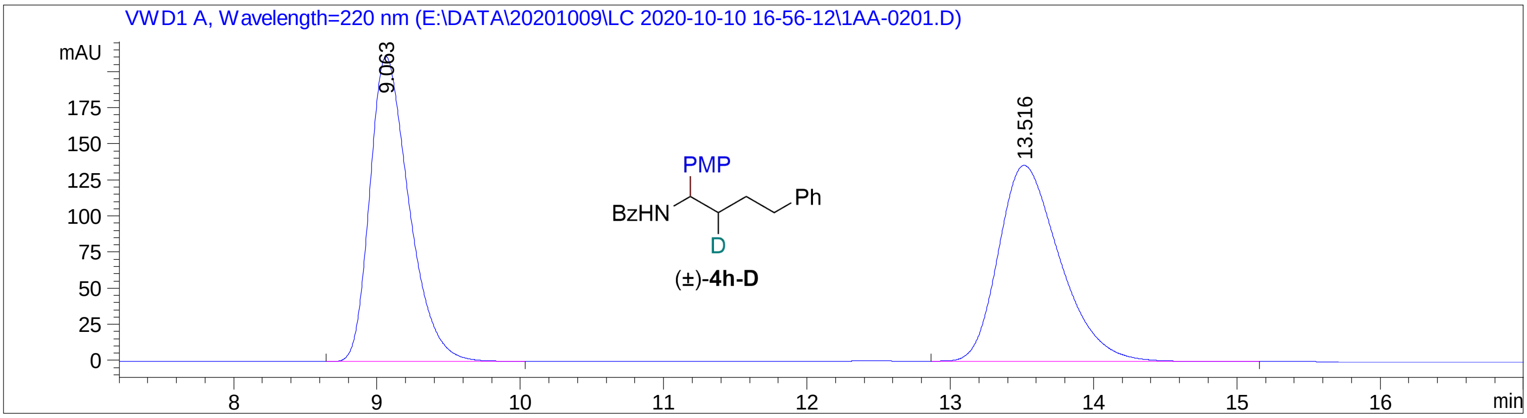

===

Area Percent Report

===

Sorted By : Signal
Multiplier : 1.0000
Dilution : 1.0000
Do not use Multiplier & Dilution Factor with ISTDs

Signal 1: VWD1 A, Wavelength=220 nm

Peak #	RetTime [min]	Type	Width [min]	Area [mAU*s]	Height [mAU]	Area %
1	9.063	BB	0.2897	4014.35278	211.20630	49.9040
2	13.516	BB	0.4557	4029.79126	135.59634	50.0960

Totals : 8044.14404 346.80264

===

*** End of Report ***

Data File E:\DATA\20201009\LC 2020-10-10 16-56-12\1AB-0301.D
Sample Name: YH-18-52-1

===
Acq. Operator : SYSTEM Seq. Line : 3
Acq. Instrument : HPLC1260 Location : P1-A2
Injection Date : 10/10/2020 5:50:13 PM Inj : 1
Inj Volume : 3.000 µl
Different Inj Volume from Sample Entry! Actual Inj Volume : 10.000 µl
Acq. Method : E:\DATA\20201009\LC 2020-10-10 16-56-12\20IPA_30_10_2.M
Last changed : 10/10/2020 5:48:04 PM by SYSTEM
Analysis Method : E:\DATA\20201009\LC 2020-10-10 16-56-12\20IPA_30_10_2.M (Sequence Method)
Last changed : 10/12/2020 10:44:41 AM by SYSTEM
(modified after loading)
Additional Info : Peak(s) manually integrated

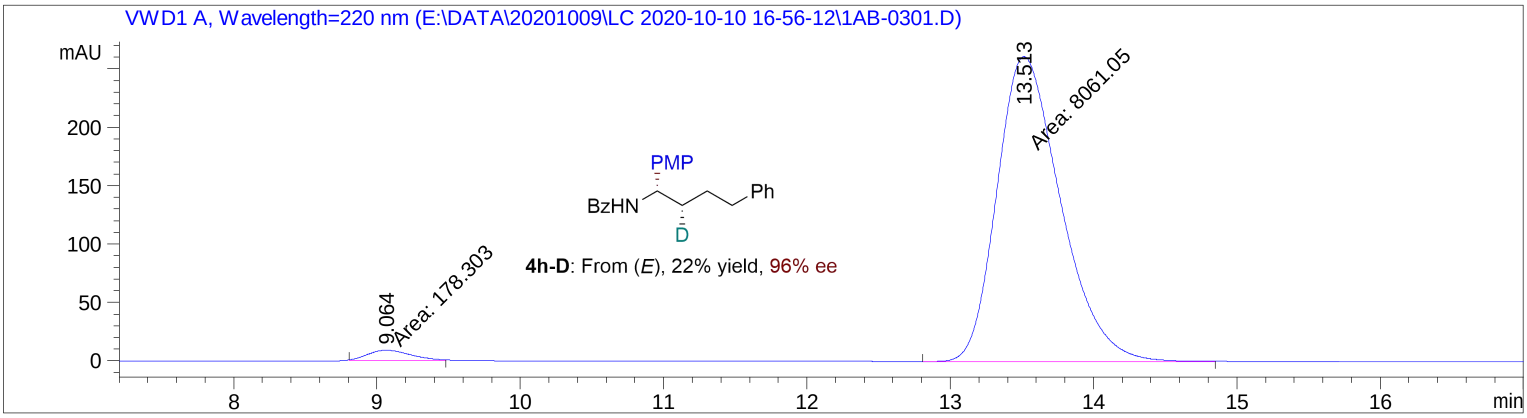

===
Area Percent Report
===

Sorted By : Signal
Multiplier : 1.0000
Dilution : 1.0000
Do not use Multiplier & Dilution Factor with ISTDs

Signal 1: VWD1 A, Wavelength=220 nm

Peak #	RetTime [min]	Type	Width [min]	Area [mAU*s]	Height [mAU]	Area %
1	9.064	MF	0.3428	178.30272	8.66791	2.1640
2	13.513	MF	0.5145	8061.05273	261.12848	97.8360

Totals : 8239.35545 269.79639

===
*** End of Report ***

Data File E:\DATA\20201009\LC 2020-10-10 16-56-12\1AC-0401.D
Sample Name: YH-18-52-2

===

Acq. Operator : SYSTEM   Seq. Line : 4
Acq. Instrument : HPLC1260   Location : P1-A3
Injection Date : 10/10/2020 6:11:07 PM   Inj : 1
Inj Volume : 3.000 µl
Different Inj Volume from Sample Entry! Actual Inj Volume : 10.000 µl
Acq. Method : E:\DATA\20201009\LC 2020-10-10 16-56-12\20IPA_30_10_2.M
Last changed : 10/10/2020 6:30:23 PM by SYSTEM
(modified after loading)
Analysis Method : E:\DATA\20201009\LC 2020-10-10 16-56-12\20IPA_30_10_2.M (Sequence Method)
Last changed : 10/10/2020 6:31:34 PM by SYSTEM
(modified after loading)
Additional Info : Peak(s) manually integrated

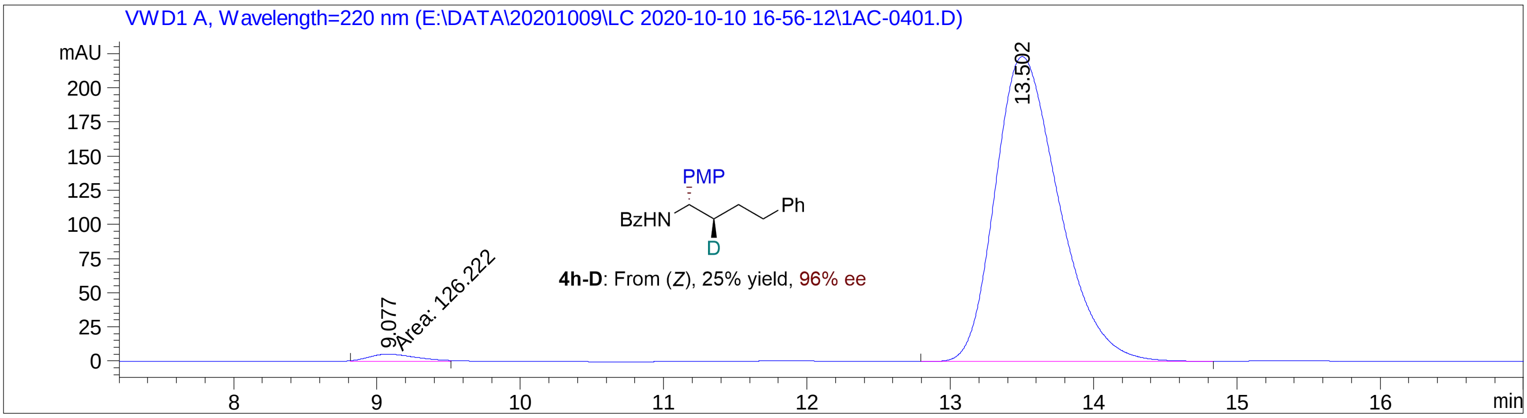

===
Area Percent Report
===

Sorted By : Signal
Multiplier : 1.0000
Dilution : 1.0000
Do not use Multiplier & Dilution Factor with ISTDs

Signal 1: VWD1 A, Wavelength=220 nm

Peak #	RetTime [min]	Type	Width [min]	Area [mAU*s]	Height [mAU]	Area %
1	9.077	FM	0.3919	126.22247	5.36766	1.7963
2	13.502	BB	0.4747	6900.64941	223.22180	98.2037

Totals : 7026.87188 228.58946

===
*** End of Report ***

XII. Supplementary References

1. Xin, S., Aikten, C., Harrod, J. F., Mu, Y. & Samuel, E. Redistribution reactions of alkoxy- and siloxysilanes, catalyzed by dimethyltitanocene. *Can. J. Chem.* **68**, 471–476 (1990).

2. Berk, S. C., Kreutzer, K. A. & Buchwald, S. L. A catalytic method for the reduction of esters to alcohols. *J. Am. Chem. Soc*. **113**, 5093–5095 (1991).

3. Berk, S. C. & Buchwald, S. L. An air-stable catalyst system for the conversion of esters to alcohols. *J. Org. Chem*. **57**, 3751–3753 (1992).

4. Jiang, L., Job, G. E., Klapars, A. & Buchwald, S. L. Copper-catalyzed coupling of amides and carbamates with vinyl halides. *Org. Lett.* **5**, 3667–3669 (2003).
